# Aziridination via Nitrogen-Atom Transfer to Olefins from Photoexcited Azoxy-Triazenes

**DOI:** 10.1021/jacs.3c14713

**Published:** 2024-03-24

**Authors:** Joshua K. Mitchell, Waseem A. Hussain, Ajay H. Bansode, Ryan M. O’Connor, Marvin Parasram

**Affiliations:** Department of Chemistry, New York University, New York, New York 10003, United States

## Abstract

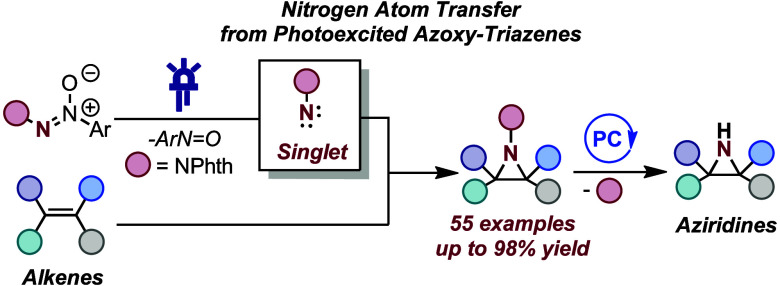

Herein, we report that readily accessible azoxy-triazenes can serve as nitrogen atom sources under visible light excitation for the phthalimido-protected aziridination of alkenes. This approach eliminates the need for external oxidants, precious transition metals, and photocatalysts, marking a departure from conventional methods. The versatility of this transformation extends to the selective aziridination of both activated and unactivated multisubstituted alkenes of varying electronic profiles. Notably, this process avoids the formation of competing C–H insertion products. The described protocol is operationally simple, scalable, and adaptable to photoflow conditions. Mechanistic studies support the idea that the photofragmentation of azoxy-triazenes results in the generation of a free singlet nitrene. Furthermore, a mild photoredox-catalyzed N–N cleavage of the protecting group to furnish the free aziridines is reported. Our findings contribute to the advancement of sustainable and practical methodologies for the synthesis of nitrogen-containing compounds, showcasing the potential for broader applications in synthetic chemistry.

Aziridines, with their inherent ring strain of 27 kcal mol^–1^, allow them to be potent synthetic handles to access valuable 1,2-aminofunctionalization products, which are featured in natural products and pharmaceutically relevant compounds.^1−8^ The aziridine core itself plays a significant role in the antitumor activity of certain small therapeutics and natural products, like mitomycin.^9^ Therefore, innovative strategies to access aziridine motifs continue to be of active interest in the synthetic community. Common strategies include the [2 + 1] cycloaddition of reactive nitrene intermediates with olefins.^10,11^ In the early 1990s, Atkinson and co-workers illustrated that the thermal formation of phthalimidonitrenes is capable of aziridination (Scheme 1A, Top). However, these approaches suffer from low reaction efficiency.^12,13^ Over the past few decades, it has been shown that the use of transition metals (TMs) can stabilize nitrene intermediates from precursors, such as haloamines, iminoiodinanes, and organic azides, or from amines under oxidative conditions, to effectuate efficient reactivity (Scheme 1A, Left).^14−18^ While each approach offers unique advantages, these methods are conducted under harsh conditions and have a limited substrate scope. Moreover, notable milder methods currently still require precious metals like Rh.^19^

**Scheme 1:**
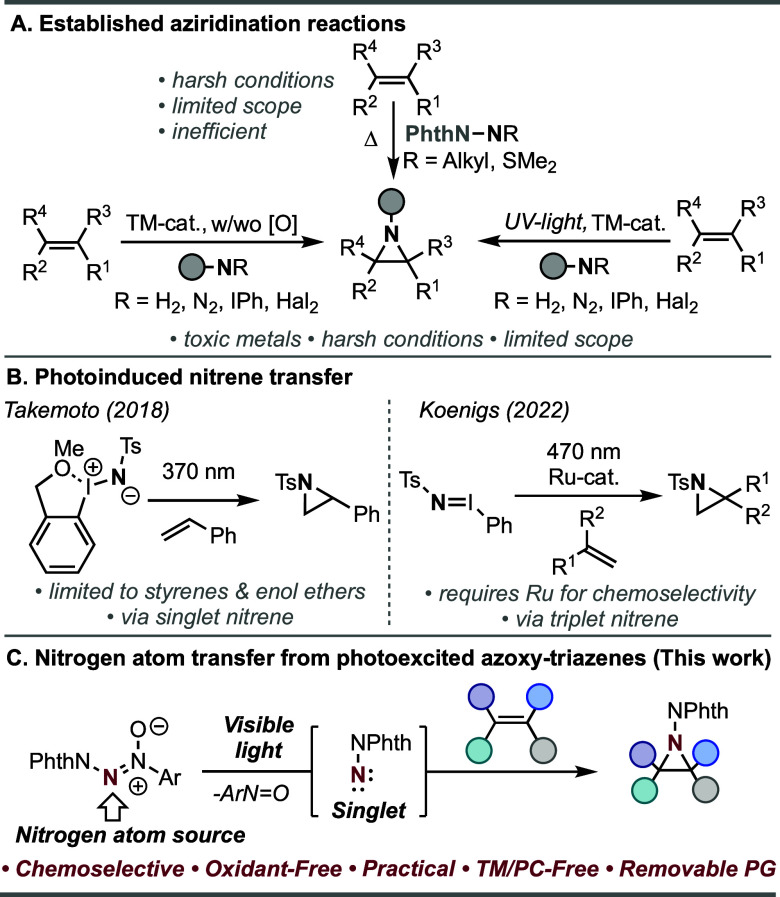
Aziridination of Alkenes

Throughout the years, approaches for the photogeneration of nitrenes have evolved, presenting complementary advantages over conventional thermal methods.^20,21^ Previously constricted to UV-light and transition metals for intermolecular nitrene transfer (Scheme 1A, Right), recent progress encompasses direct photolysis or the utilization of photocatalysts under mild visible-light conditions for free nitrene formation.^22^ In 2018, the Takemoto group demonstrated that photoexcitation of specialized *ortho*-substituted iminoiodinanes can effectively produce a free singlet nitrene (Scheme 1B, Left);^23^ however, this method was restricted to silyl enol ethers and styrenes.

In 2022, Koenigs reported that blue light excitation of iminoiodinanes can engender triplet nitrene formation, leading to allylic C–H insertion products. With the addition of a Ru-based photoredox catalyst, the reaction mechanism can be redirected to generate a nitrogen radical anion intermediate that can react with alkenes to produce aziridines, albeit with low stereospecificity (Scheme 1B, Right).^24^ Unfortunately, the reliance on precious metals like Ru^25^ for chemoselectivity can be seen as a limitation from a cost perspective. Thus, the development of a metal- and oxidant-free aziridination method is highly warranted. Herein, we report that readily synthesized azoxy-triazenes can lead to the formation of free nitrenes under direct visible-light irradiation to enable the stereospecific and chemoselective aziridination of alkenes (Scheme 1C). Previously, our group and others have reported the use of photoexcited nitroarenes as oxygen-atom-transfer agents to access alcohols from hydrocarbons^26^ and carbonyl derivatives from alkenes, aldehydes, and imines.^27−29^ Hence, we hypothesized that the use of isoelectronic azoxyarenes may trigger a nitrogen-atom-transfer event under visible-light irradiation with alkenes to give aziridines. In 1981, Hoesch and Köppel reported a single example of using azoxyarenes as nitrene precursors under harsh UV light.^30^ Recently, the Koenigs group illustrated that tosyl-protected azoxyarenes can undergo direct visible-light excitation leading to N–S bond homolysis to achieve group transfer of the azoxy to alkenes.^31^ Conversely, we postulated that the use of a phthalimide-protected azoxy-triazene, featuring a stronger N–N over an N–S bond, may lead to a nitrogen-atom transfer of a phthalimide-protected amine under visible-light irradiation for the functionalization of alkenes.

To test our hypothesis, we subjected 4-fluorostyrene (**1a**) and readily synthesized 1-phenyl-2-phthalimidodiazene-1-oxide (**AT1**)^30,32,33^ in dichloromethane to 390 nm light irradiation, which resulted in the desired aziridine product (**2a**) in 70% ^1^H NMR yield. Once the optimized reaction conditions were obtained (see Supporting Information), the electronic effect of the aziridination reaction was investigated with styrene derivatives (Table 1, **1a–1l**, **1p**). It was found that the transformation was not impacted by the electronic pattern, as substrates possessing both electron-rich and -deficient groups resulted in good to high yields (**2a–2l**, **2p**, 60–95%). Furthermore, substituents such as Me (**1c**, **1p**), *t*-Bu (**1d**), OH (**1i**), and CHO (**1l**) that are prone to C–H nitrene insertion or hydrogen atom transfer were tolerated in high yields. Unfortunately, the isolation of **2i** was difficult (see Supporting Information). Substrates **1n** and **1o** failed to react. Disubstituted alkenes gave moderate to excellent yields (**2q**–**2x**; 45–90%) of the desired aziridination products. Notably, aziridination of electron-deficient styrene **1s** is challenging under TM-free conditions;^34^ however, aziridine **2s** was obtained in 45% isolated yield under our conditions. Among the β-substituted styrenes, *cis*- and *trans*-stilbene (**1y**, **1z**) yielded no reaction. This is likely due to the strong fluorescence quenching of the starting material. Challenging trisubstituted (**1aa**) and tetrasubstituted (**1ab**) styrenes yielded **2aa**–**2ab** in moderate to good yields under the reaction conditions.

**Table 1:**
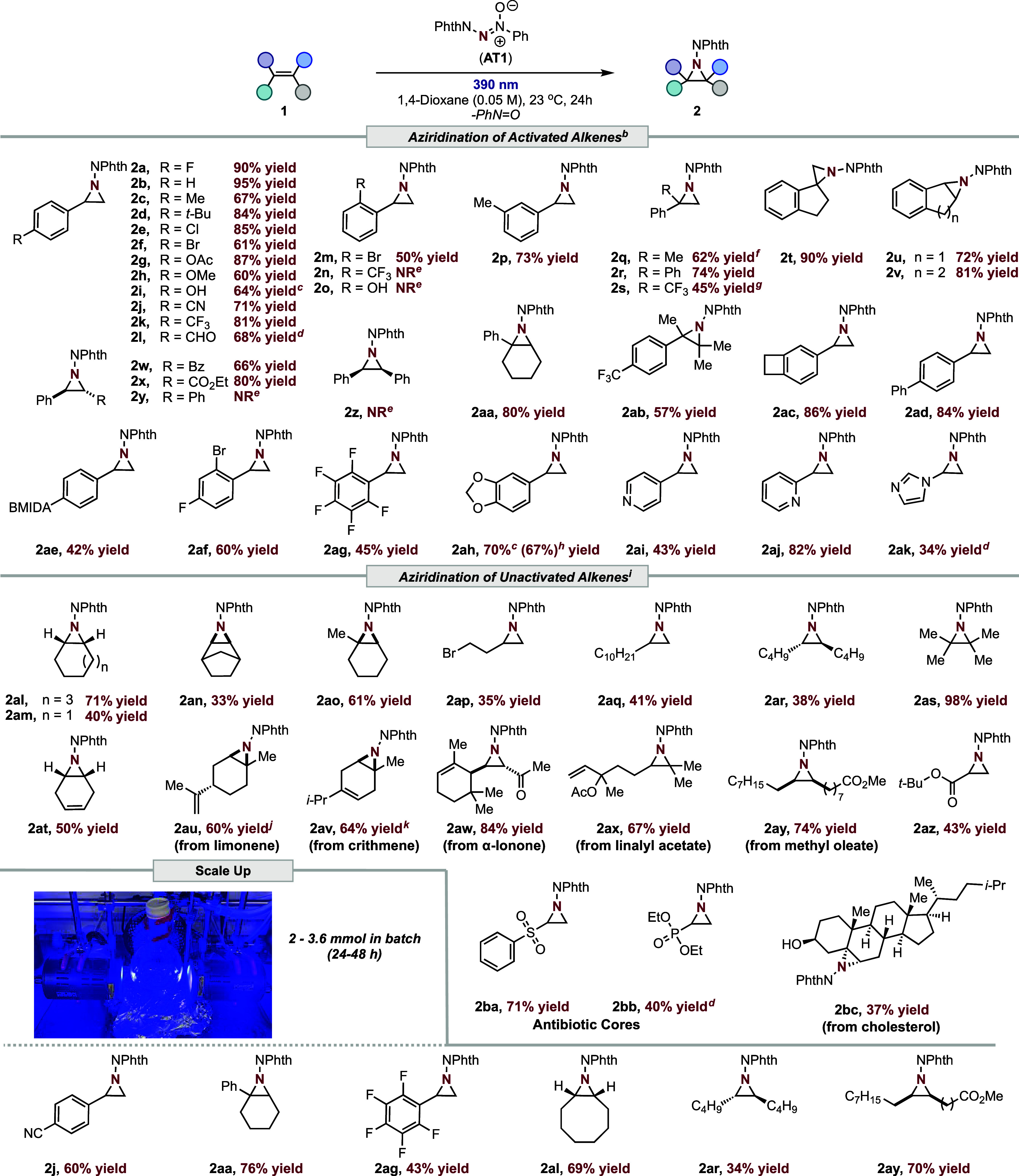
Scope of the Photoinduced Azoxy-Triazene Promoted Aziridination Reactions^a^^,^^b^

a Isolated yields.

b Conditions: 1-phenyl-2-phthalimidodiazene-1-oxide (1 equiv), 1.2 equiv of alkene, 390 nm, 1,4-dioxane (0.05 M), 23 °C, 24 h, rt.

c Denotes ^1^H NMR yield using CH_2_Br_2_ as an external standard.

d Using **AT4** (see Supporting Information).

e No reaction.

f 5.6:1 trans/cis.

g 10:1 stereoisomers.

h Isolated as 2-((2-(benzo[d][1,3]dioxol-5-yl)-2-ethoxyethyl)amino)isoindoline-1,3-dione (**2ah1**, see Supporting Information).

i Using 2.0 equiv of alkene; 0.025 M.

j Major product (d.r. 50:50); 4% ^1^H NMR yield of minor product (**2au1**, see Supporting Information) was detected.

k Major product; 14% ^1^H NMR yield of minor product (**2av1**, see Supporting Information).

Bicyclic-substituted styrene **1ac** generated **2ac** in a good yield. Other styrenes like *p*-biphenyl (**1ad**) resulted in 84% of **2ad**. Substrate **1ae**, possessing a BMIDA functional handle, was tolerated under the reaction conditions (**2ae**, 42%).^35^ Highly electron-deficient styrenes, such as **1af–1ag**, resulted in a moderate yield of aziridination product (**2af–2ag**). The sensitive acetal group of **1ah**, with a C–H bond that is prone to nitrene insertion, led to the aziridination product **2ah** selectively in a good ^1^H NMR yield (70%). Owing to the sensitivity of **2ah**, the product was subjected to ethanolysis (**2ah1**) and isolated. Other substrates prone to fluorescence quenching of **AT1**, such as heterocyclic amines (**1ai–1aj**), yielded aziridine products **2ai**–**2aj** in moderate to good yields (43–82%). However, imidazole (**1ak**) produced a low yield (**2ak**, 34%).

Next, unactivated olefins were studied under these conditions (see Supporting Information). Subjecting cycloalkenes to the reaction conditions resulted in good yields of the aziridination products (**2al–2am**, 40–71%), whereas bicyclic norbornene gave **2an** in 33% yield. Cyclic trisubstituted olefins possessing a methyl (**1ao**) substituent generated the corresponding aziridine **2ao** in a moderate yield (61%). For noncyclic substrates, terminal and internal alkenes led to moderate to excellent yields of the aziridine products (**2ap–2as**, 35–98%).

The regioselectivity of the transformation was examined on unactivated alkenes. 1,4-Cyclohexadiene (**1at**) yielded only **2at** (50%) with no diaziridination detected. Limonene (**1au**) produced aziridination product **2au** with a 15:1 ratio of internal (dr 50:50) to terminal alkene. To investigate the impact of sterics on the reactivity toward alkenes, we examined crithmene (**1av**). Aziridination (**2av**) occurred at the less hindered alkene in a 4.7:1 regioisomeric ratio. Next, odorant α-ionone (**1aw**),^36^ possessing a trisubstituted cyclic and disubstituted linear alkene, was investigated. Aziridination of the disubstituted linear alkene was the sole product detected (**2aw**). When linalyl acetate (**1ax**) was tested, boasting both noncyclic internal and terminal alkenes, regioselective aziridination of the internal alkene was obtained in 67% yield (**2ax**). These regioselectivity studies indicate that the aziridination event is sensitive to the steric profile of the alkenes. *Cis*-fatty ester, methyl oleate (**1ay**), gave 74% **2ay** under the reaction conditions. Antibiotic cores **2ba** and **2bb** were synthesized in good to excellent yields. Finally, cholesterol (**1bc**), with an unprotected alcohol, gave a moderate yield of **2bc**. This outcome complements existing aziridination protocols, where protection of oxidatively sensitive groups is not required due to the anaerobic nature of our protocol.^37−39^ Notably, in all cases, allylic C–H amination products were not detected, illustrating that this aziridination approach is highly chemoselective.

To evaluate the scalability of the method, activated and unactivated alkenes (**1j**, **1aa**, **1ag**, **1al**, **1ar**, and **1ay**) were employed in a gramscale batch setup, yielding comparable results to our isolation scale (Table 1). Employing a photoflow reactor^40,41^ (see Supporting Information) for substrates with lower yields (**1m**, **1ai**, **1ak**, and **1ap**) led to a 3-to-5-fold increase in productivity. In terms of the synthetic utility of the transformation, we report the first protocol for the N–N bond cleavage of phthalimidoaziridines (**2b**, **2x**, **2ay**, and **2al**) to furnish unprotected or derivatized aziridines (**2b1**, **2x1**, **2ay1**, and **2al2**) under mild photoredox catalysis (Scheme 2A).^42,43^ Other derivatizations of the reaction products, such as reductive (**2a1**, a phenelzine derivative)^44^ and nucleophilic (**2q1** and **2m1**) ring-opening^45−48^ as well as hydrazine workup of **Z-6c** to afford amino-aziridine (**Z**-**6c1**), are also possible (Scheme 2B).

**Scheme 2:**
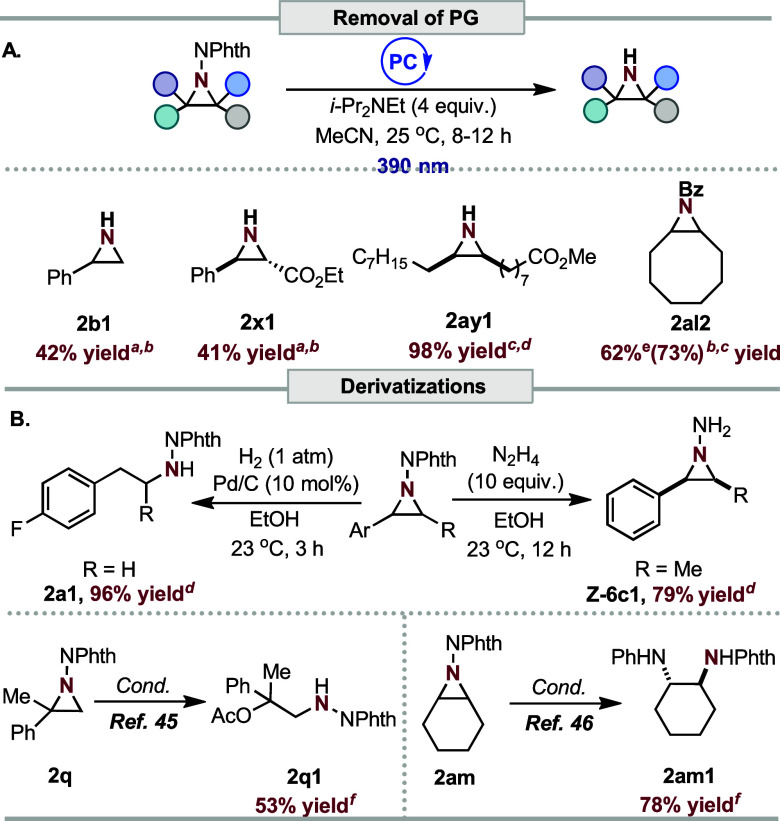
Synthetic Utility. (A) Deprotection of Aziridine. (B) Derivatization of Aziridine Products 8 mol % 4-CzIPN. Denotes ^1^H NMR yield of the aziridine using CH_2_Br_2_ as an external standard. 0.5 mol % [Ir(dtbbpy)(ppy)_2_]PF_6_. Isolated yield. Isolated as Bz-protected aziridine. Literature-reported yield.

The mechanism of the transformation was then interrogated. UV–vis studies indicated that azoxy-triazene was the sole absorbing species under the reaction conditions (Figure S3). Control experiments (Table S4; Figure S4) established that sustained light exposure was crucial for both aziridine formation and fragmentation of the azoxy-triazene. Moreover, experiments involving various triplet-state and singlet-state quenchers indicated that the azoxy-triazene predominantly enters the singlet state upon excitation (Table S6), similar to other azoxyarenes systems.^49−51^ Since our method results in chemoselective aziridination, singlet nitrene intermediates are likely formed during the reaction progress. To support this, singlet nitrene traps^52,53^ such as dimethyl sulfide (DMS, **3a**) and dimethyl sulfoxide (DMSO, **3b**) were independently used and resulted in trapped products **4a** and **4b** in 20% and >99% ^1^H NMR yield, respectively (Table 2A), strongly supporting the formation of a singlet nitrene species.

**Table 2:**
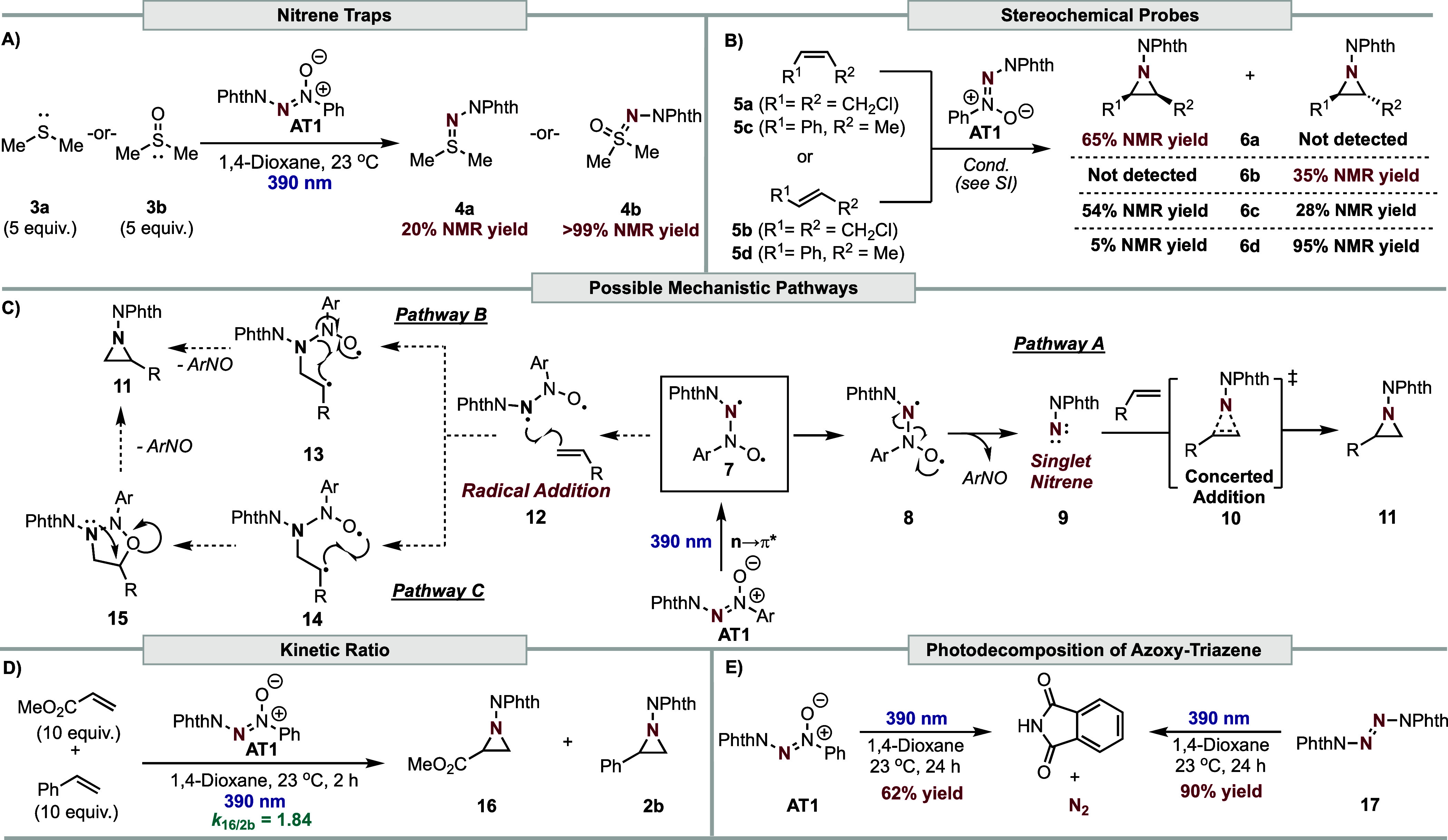
Mechanistic Studies and Proposed Mechanisms^a^

a (A) Nitrene trapping studies. (B) Stereochemical probe study. (C) Possible mechanisms. (D) Kinetic ratio study. (E) Control studies for the photodecomposition of azoxy-triazene.

Further support for singlet nitrene formation can be ascertained by the employment of stereochemical probes (Table 2B),^54,55^ where retention of the initial geometry indicates a concerted mechanism via a singlet nitrene, and ablation supports a stepwise mechanism via a triplet nitrene. Unactivated alkenes, (*Z*)-1,4-dichlorobut-2-ene (**5a**) and (*E*)-1,4-dichlorobut-2-ene (**5b**), were tested under the reaction conditions, and a concentration dependence study was conducted.^56,57^ Both of which resulted in stereospecific aziridination, thus supporting singlet nitrene formation (Table 2C, Pathway A). Notably, (*Z*)- and (*E*)-β-methylstyrene (**5c**–**5d**) led to stereoablation. This phenomenon could be explained by the propensity of styrenyl aziridine products to undergo photoisomerization, rendering **5c**–**5d** ineffective as probes (see Supporting Information).^58^ Despite this, nonconcerted reaction pathways via stepwise radical addition (**12**) of the photoexcited diradical intermediate **7** to the alkene leading to either intermediate **13** (Pathway B) or **15** (Pathway C), followed by intramolecular fragmentation to generate the aziridine product (**11**), were considered.

To determine if aziridination occurs via Pathway A rather than Pathway B or C, a kinetic competition study was conducted with styrene and methyl acrylate (Table 2D). A value of *k*_16/2a_ = 1.84 was obtained, which is identical to prior reports on free phthalimidonitrene formation.^13^ Further evidence for Pathway A was provided by the photoirradiation of the starting azoxy-triazene material in the absence of alkene, which resulted in significant detection of phthalimide, presumably via photofragmentation of nitrene dimer 1,4-bis-phthaloyltetrazene (**17**) (Table 2E, Left).^59^ This was verified by subjecting **17** to the reaction conditions, resulting in the formation of the corresponding phthalimide product in 90% yield (Table 2E, Right). The possibility of carbon-centered radical intermediates (**13** or **14**) was ruled out from the employment of radical quenchers, radical clocks, and Hammett studies (See Supporting Information).^60^ Based on the results of our mechanistic studies, Pathway A, featuring the photogeneration of a singlet free nitrene, is most probable.

In conclusion, we have illustrated that photoinduced azoxy-triazenes can promote a nitrogen atom transfer event for the chemoselective aziridination of activated and unactivated alkenes. Our method leverages the singlet-excited state of the azoxy-system that is accessed upon visible-light excitation and subsequently fragments to generate free singlet nitrenes. A wide range of functional groups were tolerated owing to the mild conditions of the transformation, and a protocol for N–N cleavage/deprotection has been provided. This relatively benign, metal-free method to attain reactive nitrene intermediates at the expense of readily accessible azoxy-triazenes is a distinct feature of the methodology that opens avenues for sustainable aziridination events and related nitrogen atom transfer reactions.

## Data Availability

The data underlying this study are available in the published article and its Supporting Information.

## Supporting Information Available

Experimental details, optimization studies, characterization data, and NMR spectra (PDF)

## Abbreviations

UV: ultraviolet
NMR: nuclear magnetic resonance
PhotoNMR: photochemical nuclear magnetic resonance
Bz: benzoyl
PC: photocatalyst
