# Supplementary material for: Aziridination via Nitrogen-Atom Transfer to Olefins from Photoexcited Azoxy-Triazenes
Source: J Am Chem Soc. 2024 Mar 24;146(14):9499–505. doi: 10.1021/jacs.3c14713 (PMC11009954; doi:10.1021/jacs.3c14713)
Supplement: Supplementary file 1 — ja3c14713_si_001.pdf [file ja3c14713_si_001.pdf]

Aziridination via Nitrogen-Atom Transfer to Olefins from Photoexcited Azoxy-Triazenes

Joshua K. Mitchell, Waseem A. Hussain, Ajay H. Bansode, Ryan M. O'Connor, and Marvin Parasram*
Department of Chemistry, New York University, New York, New York 10003, United States.

Supporting Information

Table of Contents

General Information S3
List Azoxy-Triazenes and Nitrosoarenes, and their Identifiers S4
General Procedures S5
Optimization of the reaction parameters for the aziridination of activated alkenes S8
Optimization of the reaction parameters for the aziridination of unactivated alkenes S10
Control Experiments S11
Characterization of Aziridination Products S12
  Characterization of Aziridination Products from Activated Alkenes S12
  Characterization of Aziridination Products from Unactivated Alkenes S27
  Characterization of Other Aziridine Products S34
  Starting Material Characterization S35
  Deprotected and Derivatized Aziridine Characterization S37
  Continuous-Photoflow Reaction Data S40
Possible Reaction Pathways S41
Mechanistic studies S42
  UV-Vis S42
  Light On/Off Studies S43
  Atmosphere Effects - Initial Rate Studies S44
  Hammett Studies S45
  Pinacol Probe S46
  Nitrene Traps S47
  Stereochemical Probes S49
  Stereoablation of Aziridine Product Geometry Under Visible Light S51
  Concentration Dependence Study of Stereochemical Probes S53

Kinetic Ratio Study S54
Intermediate Controls S56
Quenching Experiments S58
Radical Clock Study S59
NMR spectra S67
NMR spectra of Isolated Compounds S67
NMR Yields Spectra S130
Continuous-Flow NMR Yield Spectra S133
References S136

General Information

All requisite chemicals were purchased from Fisher Scientific, Sigma Aldrich (Merck), Oakwood Chemical (Oakwood Products), Ambeed, TCI, and used without further purification unless otherwise stated. ^{1}H NMR spectra were recorded at 400, 500, and 600 MHz; ^{13}C NMR spectra were obtained at 101 MHz, 126 MHz, and 151 MHz; and ^{19}F NMR spectra at 377 MHz and 471 MHz. All NMR were obtained on a Bruker 400, 500, and 600 MHz Advance spectrometer and are referenced to the deuterated solvent resonance. Chemical shifts (δ) are reported in parts per million (ppm), multiplicity (s = singlet, br = broad, d= doublet, t = triplet, q = quartet, qt = quintet, sext = sextet, m = multiplet), and coupling constants (J) are in Hertz (Hz). All reactions were carried out under ambient conditions unless otherwise noted. Thin-layer chromatography (TLC) was performed on 250-μm glass-backed silica gel plates and column chromatography were performed using 200–300 mesh silica gel unless otherwise stated. Deuterated chloroform ($CDCl_3$), dichloromethane (CD_2Cl_2), and acetonitrile (CD_3CN) were purchased from Cambridge Isotopes. GC chromatograms were taken on an Agilent 8890 GC with 5977B MSD, and helium as the carrier gas. High-resolution mass spectra (HRMS) were obtained on an Agilent 6224 TOF LC/MS which was acquired through the support of New York University. We utilized 34 W Kessil Lamps with varying wavelengths for the photochemical setups.

List Azoxy-Triazenes and Nitrosoarenes, and their Identifiers

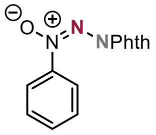

AT1

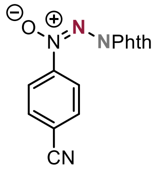

AT2

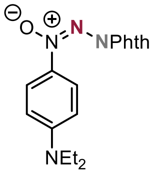

AT3

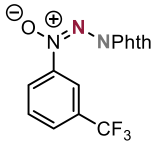

AT4

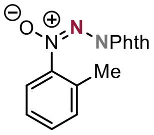

AT5

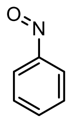

N1

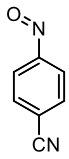

N2

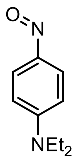

N3

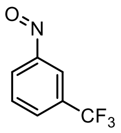

N4

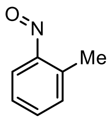

N5

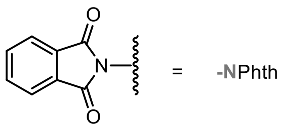

Figure S1. A list of azoxy-triazenes and nitrosoarenes, and their corresponding identifiers.

General Procedures

General Procedure A. Conditions for the Photoinduced Aziridination Reactions of Activated Alkenes.

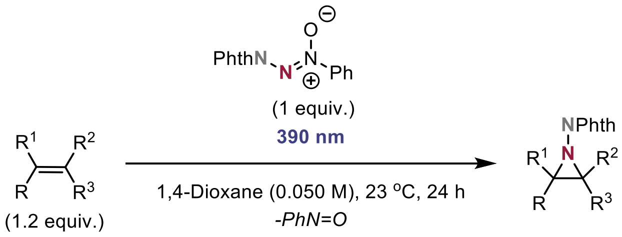

Reaction setup. Except when otherwise stated, in a 4-dram vial equipped with a stir-bar, was charged 1-phenyl-2-phthalimidodiazene 1-oxide (**AT1**, 134 mg, 0.50 mmol, 1.0 equiv.), activated alkene (0.60 mmol, 1.2 equiv.), and 1,4-dioxane (10.0 mL, 0.050 M). The reaction vessel was then capped and left to stir at 1000 rpm and irradiated under 390 nm Kessil lamps with a cooling fan for 24 h. Completion of the reaction was determined by TLC analysis. After the reaction was complete, the solvent was removed. The crude product was purified by column chromatography (0–30% EtOAc/Hexanes) to afford the aziridine products.

General Procedure B. Conditions for the Photoinduced Aziridination Reactions of Unactivated Alkenes.

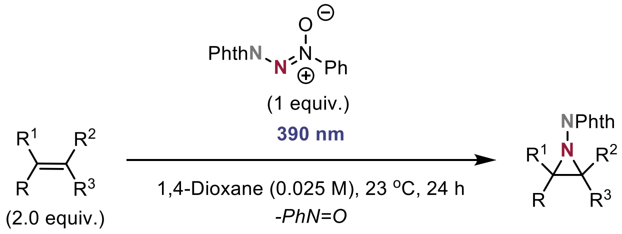

Reaction setup. Except when otherwise stated, in a 4-dram vial equipped with a stir-bar, was charged 1-phenyl-2-phthalimidodiazene 1-oxide (**AT1**, 66.8 mg, 0.25 mmol, 1.0 equiv.), unactivated alkene (0.50 mmol, 2.0 equiv.), and 1,4-dioxane (5.0 mL, 0.025 M). The reaction vessel was then capped and left to stir at 1000 rpm and irradiated under 390 nm Kessil lamps with a cooling fan for 24 h. Completion of the reaction was determined by TLC analysis. After the reaction was complete, the solvent was removed. The crude product was purified by column chromatography (0–30% EtOAc/Hexanes) to afford the aziridine products.

General Procedure C. Gram-Scale Conditions for the Photoinduced Aziridination Reactions.

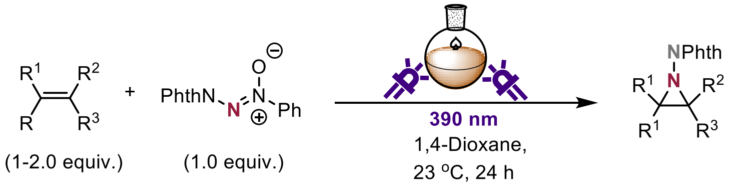

In a 250 mL round-bottom flask equipped with a stir-bar, was charged 1-phenyl-2-phthalimidodiazene 1-oxide (**AT1**, 1.0 equiv.), alkene (1.2 - 2.0 equiv.), and 1,4-dioxane (0.025 M - 0.050 M). The reaction vessel was then capped with a septum and left to stir at 800 rpm and irradiated under two 390 nm Kessil lamps from opposing angles and cooled with a cooling fan for 24 h (Figure S2a). Solvent was removed, once the reaction was complete. The crude product was purified by column chromatography (0–30% EtOAc/Hexanes) to afford the aziridine products.

General Procedure D. Continuous-Flow Conditions for the Photoinduced Aziridination Reactions.

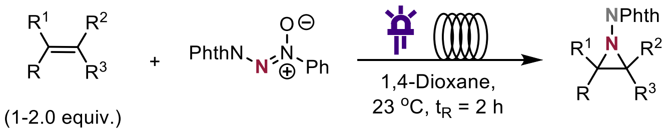

Reaction apparatus. The setup for the flow reactor used is as follows: 10 ft. of 0.03-inch diameter Fluorinated Ethylene Propylene (FEP) tubing was coiled around a 150 mL glass beaker (Figure S2b). Two 390 nm Kessil lamps (34 W each) were placed approximately 3.0 cm from the coil of tubing. An electric fan was placed behind to prevent the tubing from overheating. A syringe pump was connected to control the flow rate of the reaction.

Reaction setup. To a 2-dram vial was added 1-phenyl-2-phthalimidodiazene 1-oxide (**AT1**, 1.0 equiv.), alkene (1.2 - 2.0 equiv.), and 1,4-dioxane (0.025M - 0.05M). Then, the mixture was taken up in a 5.0 mL syringe. The mixture was dispensed through the FEP tubing. The flow rate was set using the syringe pump, the blue LEDs were switched on and the reaction flowed through for 2 h. A 3-dram vial was used to collect the reaction solution. After the given reaction time, the mixture was concentrated down and diluted with $CDCl_3$. Then, CH_2Br_2 (1.0 equiv.) was added as an external ^{1}H NMR standard. The NMR yield of the product was recorded.

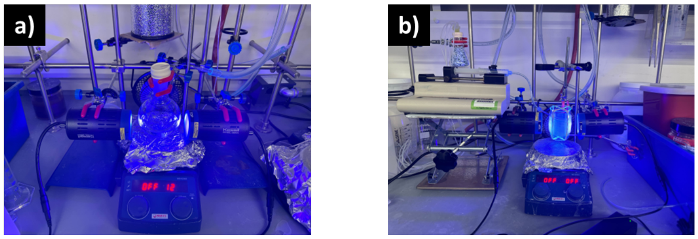

Figure S2. Visual representation of photochemical setup for A) Scaled up and B) Photoflow reactions.

General Procedure E. Synthesis of Azoxy-Triazenes.

While there are several ways of synthesizing azoxy-triazenes which we have tested,[1,2,3] for simplicity and reduced reaction times, we primarily used the following:

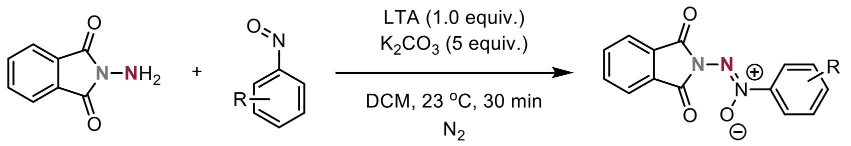

Reaction setup. In a 250 mL Schlenk flask was added nitrosoarene (7.0 mmol, 1.0 equiv.), potassium carbonate (35.0 mmol, 5.0 equiv.), and amino-phthalimide (7.0 mmol, 1.0 equiv.), which was then purged under nitrogen. After which, 100 mL of dichloromethane was added, and the mixture was allowed to stir vigorously. A solution of lead tetraacetate (7.0 mmol, 1.0 equiv.) in 15 mL of dichloromethane under nitrogen was added dropwise to the reaction mixture and the vessel was left to stir for 1 h. When the reaction was complete, the mixture filtered through a thick pad of celite and silica. The filtrate was concentrated down under reduced pressure. The crude was then recrystallized in hot ethanol. During recrystallization, the solid did not need to be completely dissolved in the ethanol to produce spectroscopically pure products.

General Procedure F. Deprotection of phthalimidonitrenes to furnish free aziridines.

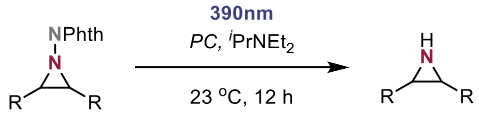

Reaction setup. In a vial was placed a stir bar, phthalimidoaziridine (1.0 equiv.), *N,N*-diisopropylethylamine (4.0 equiv.), photocatalyst (0.005-0.08 equiv.), and MeCN (0.05 M) under nitrogen. The reaction vessel was then irradiated under a 390 nm Kessil lamp, 4 inches away with 2 fans, and stirred at 1000 rpm for 12 h. The reaction was then quenched with a small amount of water, filtered through a pad of sodium sulfate and celite, and concentrated down under a stream of nitrogen. Then, CH_2Br_2 (1.0 equiv.) was added as an external ^{1}H NMR standard. The NMR yield of the product was recorded. *The reactions appears to be very sensitive to time and temperature. Isolation of reaction products is challenging.*

Optimization of the reaction parameters for the aziridination of activated alkenes

Table S1. Solvent screen for activated substrates.

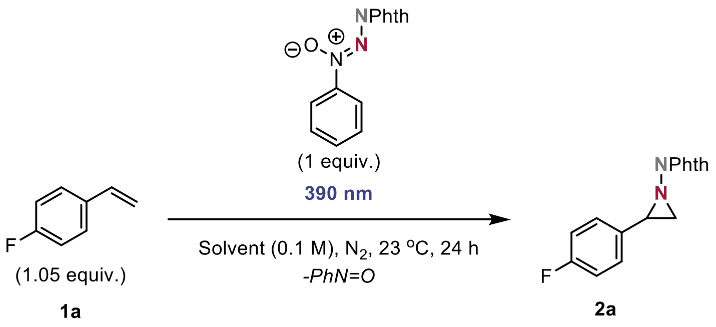

Entry	*Solvent*	*Yield* (**2a**) [a]
1	$PhCF_3$	13%
2	NMP	45%
3	Nitromethane	59%
4	Acetone	63%
5	THF	64%
6	DCM	70%
7	DCE	71%
8	DMA	75%
9	**MeCN**	**80%**
10	**1,4-Dioxane**	**84%**

Reactions were performed on a 0.1 mmol scale. [a] Denotes ^{1}H NMR yields of **2a** using CH_2Br_2 as an external standard. *Solvents found to react with the photoexcited azoxy-triazene: DMSO and MeOH.

Table S2. Concentration screen for activated substrates.

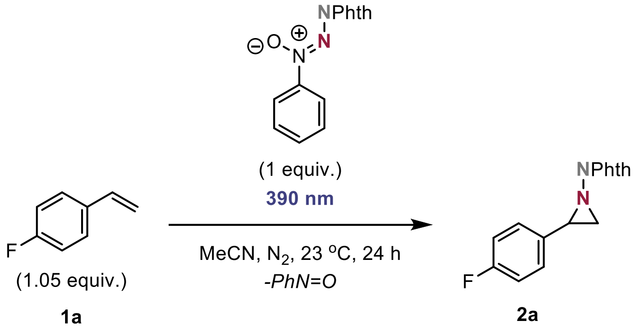

Entry	*Solvent*	***1a*** *(mmol)*	*Concentration (mM)*	*Yield (**2a**)* [a]
1	MeCN	0.1	0.2	39%
2	MeCN	0.1	0.1	80%
3	1,4-Dioxane	0.1	0.05	79%
4	**MeCN**	**0.1**	**0.05**	**>99%**
5	MeCN	0.5	0.05	75%
6	**1,4-Dioxane**	**0.5**	**0.05**	**94%**

[a] Denotes ^{1}H NMR yields of **2a** using CH_2Br_2 as an external standard.

Optimization of the reaction parameters for the aziridination of unactivated alkenes

Table S3. Optimization of unactivated substrates.

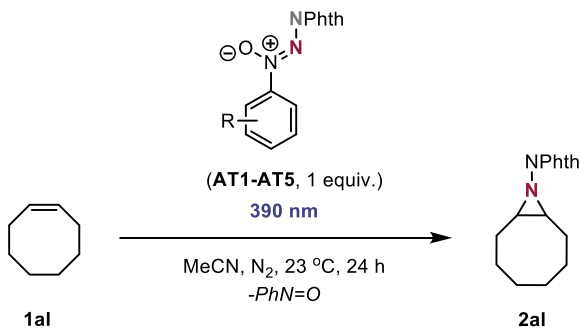

Entry	*Azoxy-Triazene*	*Equiv.* (***1al***)	*Concentration (mM)*	*Yield* (***2al***) [a]
1	**AT1**	1.0	50	59%
2	**AT1**	1.0	25	62%
2	**AT1**	1.5	25	69%
3	**AT1**	2.0	25	71%
4	**AT1**	2.0	25	71%[b]
5	**AT2**	2.0	25	58%
6	**AT3**	2.0	25	36%
7	**AT4**	2.0	25	30%
8	**AT5**	2.0	25	0%
9	**AT1**	2.0 (of **1ao**) [c]	25	81% (of **2ao**)
10	**AT1**	2.0 (of **1aq**) [c]	25	56% (of **2aq**)
11	**AT1**	2.0 (of **1as**) [c]	25	98% (of **2as**)

Reactions were performed on a 0.1 mmol scale. [a] Denotes ^{1}H NMR yields of **2a** using CH_2Br_2 as an external standard. [b] Without fan ~temperature rises to 40 °C. [c] For substrate **1ao**, **1aq**, and **1as**, refer to the characterization section.

Control Experiments

Table S4. Control experiments.

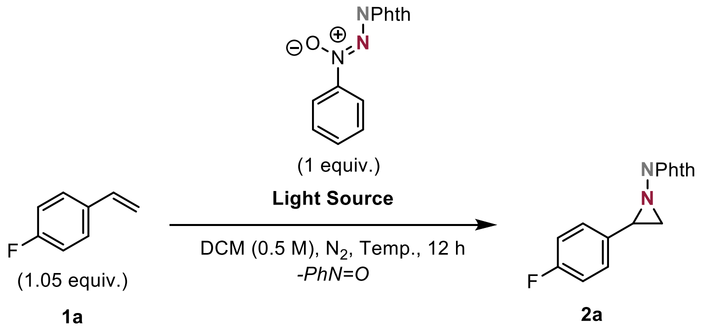

Entry	*Solvent*	*Conditions*	*Yield (**2a**)* [a]
1	DCM	427 nm @ 23°C	39%
**2**	**DCM**	**390 nm @ 23**°C	**72%**
3	DCM	370 nm @ 23°C	94%
4	DCM	Dark @ 40°C	0%
5	MeCN	Dark @ 82°C	0%
6	1,4-Dioxane	Nitrogen @ 23°C	79%
7	1,4-Dioxane	Air @ 23°C	78%
8	1,4-Dioxane	Oxygen @ 23°C	82%

Reactions were performed on a 0.1 mmol scale. [a] Denotes ^{1}H NMR yields of **2a** using CH_2Br_2 as an external standard.

Characterization of Aziridination Products

Characterization of Aziridination Products from Activated Alkenes

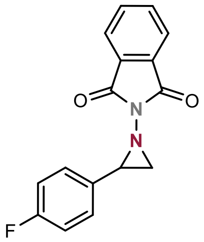

2-(2-(4-fluorophenyl)aziridin-1-yl)isoindoline-1,3-dione (2a)

Following General Procedure A, prepared from 0.50 mmol of **AT1** and 1.0 equiv. of 4-fluorostyrene (**1a**) in 24 h, the title compound was isolated via column chromatography (0–5% EtOAc/Hexanes) as a white solid (122 mg, 90% yield). Analytical data for **2a** was in accordance with literature data.[4]

TLC (SiO_2) R_f = 0.25 in 9:1 Hexanes:EtOAc.

^{1}H NMR (400 MHz, $CDCl_3$) (δ, ppm): 7.82 – 7.79 (m, 2H), 7.73 – 7.69 (m, 2H), 7.50 – 7.41 (m, 2H), 7.12 – 7.03 (m, 2H), 3.59 (dd, *J* = 7.9, 5.9 Hz, 1H), 2.89 (dd, *J* = 7.9, 2.5 Hz, 1H), 2.77 (dd, *J* = 5.8, 2.5 Hz, 1H).

^{13}C NMR (101 MHz, $CDCl_3$) (δ, ppm): 165.5, 134.7, 130.8, 129.6, 129.5, 123.6, 116.1, 115.8, 44.4, 40.0.

HRMS (ESI-TOF): *m/z* calculated for $C_{16}H_{11}FN_2O_2$ $[M]^+$ = 298.0805 , found 298.0800.

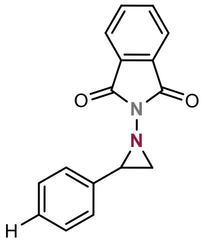

2-(2-phenylaziridin-1-yl)isoindoline-1,3-dione (2b)

Following General Procedure A, prepared from 0.50 mmol of **AT1** and 1.2 equiv. of styrene (**1b**) in 24 h, the title compound was isolated via flash column chromatography (0–5% EtOAc/Hexanes) as an off-white solid (126 mg, 95% yield). Analytical data for **2b** was in accordance with literature data.[4]

TLC (SiO_2) R_f = 0.21 in 9:1 Hexanes:EtOAc.

^{1}H NMR (400 MHz, $CDCl_3$) (δ, ppm): 7.84 – 7.77 (m, 2H), 7.74 – 7.67 (m, 2H), 7.49 – 7.43 (m, 2H), 7.42 – 7.36 (m, 2H), 7.36 – 7.30 (m, 1H), 3.62 (dd, *J* = 8.0, 5.9 Hz, 1H), 2.90 (dd, *J* = 8.0, 2.5 Hz, 1H), 2.80 (dd, *J* = 5.9, 2.5 Hz, 1H).

^{13}C NMR (101 MHz, $CDCl_3$) (δ, ppm): 165.2, 136.5, 134.3, 130.5, 128.9, 128.2, 127.4, 123.3, 44.7, 39.9.

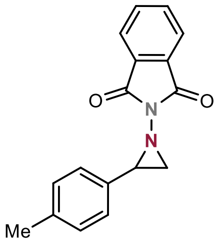

2-(2-(p-tolyl)aziridin-1-yl)isoindoline-1,3-dione (2c)

Following General Procedure A, prepared from 0.50 mmol of **AT1** and 1.2 equiv. of 4-methyl styrene (**2c**) in 24 h. The title compound was isolated via flash column chromatography (9:1:1:0.1, Hexanes:EtOAc:DCM:Et_3N) as an off-white solid (93 mg, 67% yield). Analytical data for **2c** was in accordance with literature data.[5]

TLC (SiO_2) R_f = 0.30 in 9:0.9:0.1 Hexanes:EtOAc:Et_3N.

^{1}H NMR (500 MHz, $CDCl_3$) (δ, ppm): 7.79 (dd, *J* = 5.4, 3.1 Hz, 2H), 7.69 (dd, *J* = 5.4, 3.0 Hz, 2H), 7.35 (d, *J* = 8.2 Hz, 2H), 7.19 (d, *J* = 7.8 Hz, 2H), 3.57 (dd, *J* = 7.9, 5.9 Hz, 1H), 2.87 (dd, *J* = 8.0, 2.5 Hz, 1H), 2.79 (dd, *J* = 5.9, 2.5 Hz, 1H), 2.36 (s, 3H).

^{13}C NMR (126 MHz, $CDCl_3$) (δ, ppm): 165.2, 138.0, 134.2, 133.4, 130.5, 129.4, 127.3, 123.2, 44.6, 39.7, 21.4.

HRMS (ESI-TOF): *m/z* calculated for $C_{17}H_{14}N_2O_2$ $[M]^+$ = 278.1055., found 278.1042.

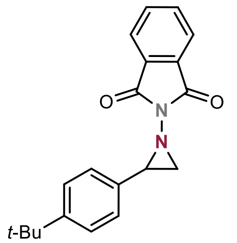

2-(2-(4-(*tert*-butyl)phenyl)aziridin-1-yl)isoindoline-1,3-dione (2d)

Following General Procedure A, prepared from 0.50 mmol of **AT1** and 1.2 equiv. of 4-*tert*-butylstyrene (**1d**) in 24 h, the title compound was isolated via flash column chromatography (0–5% EtOAc/Hexanes) as a white solid (135 mg, 84% yield).

TLC (SiO_2) R_f = 0.20 in 8:2 Hexanes:EtOAc.

^{1}H NMR (400 MHz, $CDCl_3$) (δ, ppm): 7.79 (dd, *J* = 5.4, 3.1 Hz, 2H), 7.69 (dd, *J* = 5.5, 3.0 Hz, 2H), 7.43 – 7.36 (m, 4H), 3.58 (dd, *J* = 7.9, 5.9 Hz, 1H), 2.87 (dd, *J* = 7.9, 2.5 Hz, 1H), 2.81 (dd, *J* = 5.9, 2.5 Hz, 1H), 1.32 (s, 9H).

^{13}C NMR (101 MHz, $CDCl_3$) (δ, ppm): 165.2, 151.2, 134.2, 133.4, 130.5, 127.0, 125.7, 123.2, 44.5, 39.8, 34.7, 31.5.

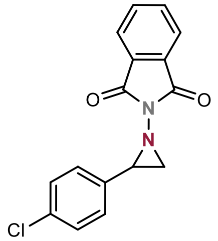

2-(2-(4-chlorophenyl)aziridin-1-yl)isoindoline-1,3-dione (2e)

Following General Procedure A, prepared from 0.50 mmol of **AT1** and 1.2 equiv. of 4-chlorostyrene (**1e**) in 24 h, the title compound was isolated via flash column chromatography (0–10% EtOAc/Hexanes) as an off-white solid (127 mg, 85% yield). Analytical data for **2e** was in accordance with literature data.[4]

TLC (SiO_2) R_f = 0.25 in 9:1 Hexanes:EtOAc.

^{1}H NMR (400 MHz, $CDCl_3$) δ 7.80 (dd, *J* = 5.4, 3.1 Hz, 2H), 7.71 (dd, *J* = 5.5, 3.1 Hz, 2H), 7.42 – 7.33 (m, 4H), 3.57 (dd, *J* = 7.9, 5.8 Hz, 1H), 2.91 (dd, *J* = 7.9, 2.5 Hz, 1H), 2.75 (dd, *J* = 5.8, 2.5 Hz, 1H).

^{13}C NMR (101 MHz, $CDCl_3$) δ 165.2, 135.1, 134.4, 134.1, 130.5, 128.9, 128.8, 123.3, 44.0, 39.9.

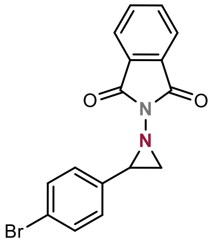

2-(2-(4-bromophenyl)aziridin-1-yl)isoindoline-1,3-dione (2f)

Following General Procedure A, prepared from 0.50 mmol of **AT1** and 1.2 equiv. of 4-bromostyrene (**1f**) in 24 h, the title compound was isolated via flash column chromatography (0–10% EtOAc/Hexanes) as an off-white solid (105 mg, 61% yield). Analytical data for **2f** was in accordance with literature data.[4]

TLC (SiO_2) R_f = 0.22 in 9:1 Hexanes:EtOAc.

^{1}H NMR (400 MHz, $CDCl_3$) (δ, ppm): 7.80 (dd, *J* = 5.3, 3.1 Hz, 2H), 7.70 (dd, *J* = 5.5, 3.1 Hz, 2H), 7.53 – 7.47 (m, 2H), 7.31 (m, 2H), 3.55 (dd, *J* = 7.9, 5.8 Hz, 1H), 2.91 (dd, *J* = 7.9, 2.6 Hz, 1H), 2.74 (dd, *J* = 5.8, 2.4 Hz, 1H).

^{13}C NMR (126 MHz, $CDCl_3$) (δ, ppm): 165.4, 135.9, 134.6, 132.1, 130.7, 129.4, 123.6, 122.5, 44.3, 40.1.

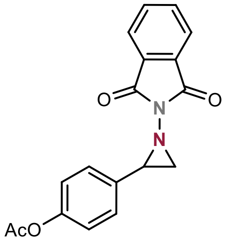

4-(1-(1,3-dioxoisoindolin-2-yl)aziridin-2-yl)phenyl acetate (2g)

Following General Procedure A, prepared from 0.50 mmol of **AT1** and 1.2 equiv. of 4-vinyl phenyl acetate (**1g**) in 24 h, the title compound **2g** was isolated via flash column chromatography (0–8% EtOAc/Hexanes) as a white solid (140 mg, 87% yield).

TLC (SiO_2) R_f = 0.25 in 9:1 Hexanes:EtOAc.

^{1}H NMR (400 MHz, $CDCl_3$) (δ, ppm): 7.80 (dd, *J* = 5.6, 2.9 Hz, 2H), 7.70 (dd, *J* = 5.6, 2.9 Hz, 2H), 7.52 – 7.44 (m, 2H), 7.15 – 7.07 (m, 2H), 3.61 (dd, *J* = 7.9, 5.8 Hz, 1H), 2.90 (dd, *J* = 7.9, 2.5 Hz, 1H), 2.76 (dd, *J* = 5.8, 2.5 Hz, 1H).

^{13}C NMR (101 MHz, $CDCl_3$) (δ, ppm): 169.5, 165.1, 150.6, 134.3, 134.1, 130.4, 128.5, 123.2, 121.8, 44.1, 39.8, 21.2.

HRMS (ESI-TOF): *m/z* calculated for $C_{18}H_{14}N_2O_4$ $[M]^+$ = 322.0954 , found 322.0950.

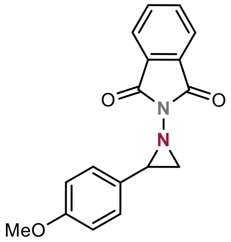

2-(2-(4-methoxyphenyl)aziridin-1-yl)isoindoline-1,3-dione (2h)

Following General Procedure A, prepared from 0.50 mmol of **AT1** and 1.2 equiv. of 4-vinyl anisole (**1h**) in 24 h, the title compound was isolated via flash column chromatography (9:0.9:0.1

Hexanes:EtOAc:Et_3N) as an off-white solid (89 mg, 60% yield). Analytical data for **2h** was in accordance with literature data.[5]
TLC (SiO_2) R_f = 0.07 in 9:0.9:0.1 Hexanes:EtOAc:Et_3N.
^{1}H NMR (400 MHz, $CDCl_3$) (δ, ppm): 7.84 – 7.76 (m, 2H), 7.74 – 7.66 (m, 2H), 7.45 – 7.37 (m, 2H), 6.99 – 6.87 (m, 2H), 3.82 (s, 3H), 3.56 (dd, *J* = 8.0, 5.9 Hz, 1H), 2.86 (dd, *J* = 7.9, 2.5 Hz, 1H), 2.80 (dd, *J* = 6.0, 2.6 Hz, 1H).
^{13}C NMR (101 MHz, $CDCl_3$) (δ, ppm): 165.2, 159.7, 134.2, 130.5, 128.8, 128.5, 123.2, 114.2, 55.5, 44.5, 39.5.

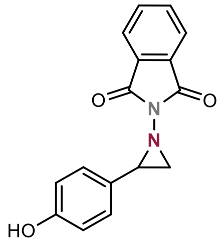

2-(2-(4-hydroxyphenyl)aziridin-1-yl)isoindoline-1,3-dione (2i)
Following General Procedure A, prepared from 0.50 mmol of **AT1** and 1.2 equiv. of 4-hydroxystyrene (**1i**) in 24 h. ^{1}H NMR yield determined using CH_2Br_2 as an external standard 64% NMR yield. The product ring opens when exposed to silica gel. The product is very sensitive and difficult to isolate.
^{1}H NMR (crude) (500 MHz, $CDCl_3$) (δ, ppm): 3.57 (dd, 1H, aziridine proton), 3.34 (dd, 1H, aziridine proton).
HRMS (ESI-TOF): *m/z* calculated for $C_{16}H_{12}N_2O_3$ $[M]^+$ = 280.0848, found 280.0857.

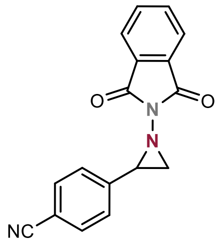

4-(1-(1,3-dioxoisoindolin-2-yl)aziridin-2-yl)benzonitrile (2j)
Following General Procedure A, prepared from 0.50 mmol of **AT1** and 1.2 equiv. of 4-vinyl benzonitrile (**1j**) in 24 h, the title compound was isolated via flash column chromatography (0–5% EtOAc/Hexanes) as an off-white solid (103 mg, 71% yield).
TLC (SiO_2) R_f = 0.20 in 8:2 Hexanes:EtOAc
^{1}H NMR (500 MHz, $CDCl_3$) (δ, ppm): 7.81 (dd, *J* = 5.4, 3.1 Hz, 2H), 7.72 (dd, *J* = 5.5, 3.1 Hz, 2H), 7.67 (d, *J* = 8.3 Hz, 2H), 7.56 (d, *J* = 8.3 Hz, 2H), 3.62 (dd, *J* = 7.9, 5.6 Hz, 1H), 2.99 (dd, *J* = 7.9, 2.4 Hz, 1H), 2.75 (dd, *J* = 5.6, 2.5 Hz, 1H).
^{13}C NMR (126 MHz, $CDCl_3$) (δ, ppm): 165.1, 142.2, 134.5, 132.5, 130.4, 128.0, 123.4, 118.8, 112.0, 43.8, 40.4.
HRMS (ESI-TOF): *m/z* calculated for $C_{17}H_{11}N_3O_2$ $[M]^+$ = 289.0805, found 289.0852.

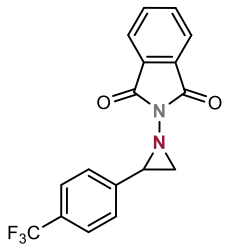

2-(2-(4-(trifluoromethyl)phenyl)aziridin-1-yl)isoindoline-1,3-dione (2k)
Following General Procedure A, prepared from 0.50 mmol of **AT1** and 1.2 equiv. of 4-(Trifluoromethyl) styrene (**1k**) in 24 h, the title compound was isolated via flash column chromatography (0–5% EtOAc/Hexanes) as a white solid (135 mg, 81% yield).
TLC (SiO_2) R_f = 0.20 in 8:2 Hexanes:EtOAc
^{1}H NMR (600 MHz, $CDCl_3$) (δ, ppm): 7.81 (dd, *J* = 5.4, 3.0 Hz, 2H), 7.72 (dd, *J* = 5.5, 3.0 Hz, 2H), 7.64 (d, *J* = 7.9 Hz, 2H), 7.57 (d, *J* = 7.8 Hz, 2H), 3.64 (dd, *J* = 7.9, 5.7 Hz, 1H), 2.97 (dd, *J* = 7.9, 2.5 Hz, 1H), 2.77 (dd, *J* = 5.7, 2.4 Hz, 1H).
^{13}C NMR (151 MHz, $CDCl_3$) (δ, ppm): 164.9, 140.5, 134.2, 130.2, 130.1, 127.5, 125.47, 125.45, 125.42, 125.40, 123.2, 43.7, 40.0.
HRMS (ESI-TOF): *m/z* calculated for $C_{17}H_{11}N_3O_2$ $[M]^+$ = 332.0773, found 332.0785.

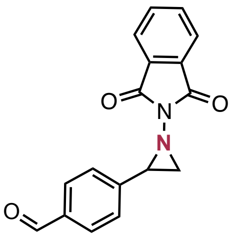

4-(1-(1,3-dioxoisoindolin-2-yl)aziridin-2-yl)benzaldehyde (2l)
Following General Procedure A, prepared from 0.25 mmol of **AT4** and 1.2 equiv. of *p*-vinyl benzaldehyde (**1l**) in 24 h. The title compound was isolated via silica gel column chromatography in (1:10:94 Et_3N/EtOAc/Hexanes) as a pale white solid (50 mg, 68% yield).
TLC (SiO_2) R_f = 0.4 in 9:1 Hexanes:EtOAc
^{1}H NMR (crude) (500 MHz, $CDCl_3$) (δ, ppm): 10.03 (s, 1H), 7.93 – 7.88 (m, 2H), 7.81 (dd, *J* = 5.4, 3.1 Hz, 2H), 7.72 (dd, *J* = 5.4, 3.0 Hz, 2H), 7.62 (s, 2H), 3.66 (dd, *J* = 8.0, 5.7 Hz, 1H), 2.99 (dd, *J* = 8.0, 2.4 Hz, 1H), 2.79 (dd, *J* = 5.7, 2.4 Hz, 1H).
^{13}C NMR (126 MHz, $CDCl_3$) (δ, ppm): 192.0, 165.1, 143.6, 136.2, 134.4, 130.4, 130.1, 127.9, 123.4, 44.1, 40.5.
HRMS (ESI-TOF): *m/z* calculated for $C_{17}H_{12}N_2O_3$ $[M]^+$ = 292.0848, found 292.0858.

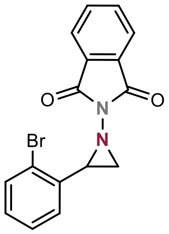

2-(2-(2-bromophenyl)aziridin-1-yl)isoindoline-1,3-dione (2m)
Following General Procedure A, prepared from 0.50 mmol of **AT1** and 1.2 equiv. of 2-bromostyrene (**1m**) in 24 h. The title compound was isolated via flash column chromatography (8.8:1:1,

Hexanes:EtOAc:Et_3N) as a white solid (86 mg, 50% yield). Analytical data for **2m** was in accordance with literature data.[4]

TLC (SiO_2) R_f = 0.25 in 8.8:1:1 Hexanes:EtOAc:Et_3N

^{1}H NMR (500 MHz, $CDCl_3$) (δ, ppm): 7.81 (dd, *J* = 5.4, 3.1 Hz, 2H), 7.71 (dd, *J* = 5.4, 3.1 Hz, 2H), 7.58 (ddd, *J* = 14.7, 7.9, 1.5 Hz, 2H), 7.33 (td, *J* = 7.5, 1.2 Hz, 1H), 7.18 (td, *J* = 7.7, 1.8 Hz, 1H), 3.80 (dd, *J* = 8.1, 5.7 Hz, 1H), 3.05 (dd, *J* = 8.1, 2.1 Hz, 1H), 2.55 (dd, *J* = 5.7, 2.1 Hz, 1H).

^{13}C NMR (126 MHz, $CDCl_3$) (δ, ppm): 165.2, 136.6, 134.3, 132.3, 130.5, 129.3, 128.7, 127.8, 123.5, 123.3, 44.8, 40.7.

HRMS (ESI-TOF): *m/z* calculated for $C_{16}H_{11}BrN_2O_2$ $[M]^+$ = 342.0004, found 342.0002.

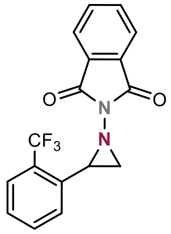

2-(2-(2-(trifluoromethyl)phenyl)aziridin-1-yl)isoindoline-1,3-dione (2n)

Following General Procedure A, prepared from 0.50 mmol of **AT1** and 1.2 equiv. of 2-(trifluoromethyl)styrene (**1n**) in 24 h. The title compound resulted in no reaction.

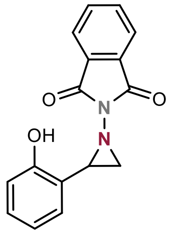

2-(2-(2-hydroxyphenyl)aziridin-1-yl)isoindoline-1,3-dione (2o)

Following General Procedure A, prepared from 0.50 mmol of **AT1** and 1.2 equiv. of 2-hydroxystrene (**1o**) in 24 h. The title compound resulted in no reaction.

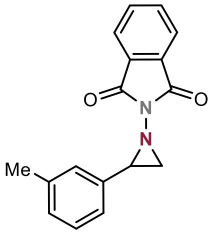

2-(2-(*m*-tolyl)aziridin-1-yl)isoindoline-1,3-dione (2p)

Following General Procedure A, prepared from 0.25 mmol of **AT1** and 1.2 equiv. of 1-methyl-3-vinylbenzene (**1p**) in 24 h, the title compound was isolated via flash column chromatography (0–10% EtOAc/Hexanes) as a yellow solid (51 mg, 73% yield).

TLC (SiO_2) R_f = 0.15 in 9:1 Hexanes:EtOAc.

^{1}H NMR (500 MHz, CD_3CN) (δ, ppm): 7.76 (d, *J* = 1.2 Hz, 4H), 7.28 (t, *J* = 7.6 Hz, 1H), 7.23 (s, 1H), 7.20 (d, *J* = 7.6 Hz, 1H), 7.16 (d, *J* = 7.4 Hz, 1H), 3.53 (dd, *J* = 8.0, 5.8 Hz, 1H), 2.88 (dd, *J* = 8.0, 2.3 Hz, 1H), 2.71 (dd, *J* = 5.8, 2.3 Hz, 1H), 2.36 (s, 3H).

^{13}C NMR (126 MHz, CD_3CN) (δ, ppm): 165.9, 139.1, 138.1, 135.1, 131.5, 129.5, 129.3, 128.5, 125.1, 123.5, 45.2, 40.4, 21.3.
HRMS (ESI-TOF): *m/z* calculated for $C_{17}H_{11}N_3O_2$ $[M]^+$ = 278.1055, found 278.1070.

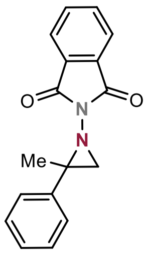

2-(2-methyl-2-phenylaziridin-1-yl)isoindoline-1,3-dione (2q)
Following General Procedure A, prepared from 0.50 mmol of **AT1** and 1.2 equiv. of α-methyl styrene (**1q**) in 24 h, the title compound was isolated via flash column chromatography (0–5% EtOAc/Hexanes) as a white solid (86 mg, 62% yield; cis : trans = 1: 5.6). Analytical data for **2q** was in accordance with literature data.[6]
^{1}H NMR (400 MHz, $CDCl_3$) (δ, ppm): 7.82 (dd, *J* = 5.4, 3.1 Hz, 2H, *trans*), 7.75 – 7.65 (m, 4H, *trans*), 7.61 – 7.52 (m, 4H, *cis*), 7.43 – 7.41 (m, 2H, *cis*), 7.40 – 7.35 (m, 1H, *trans*), 7.33 – 7.27 (m, 1H, *trans*), 7.24 – 7.13 (m, 3H, *cis*), 4.26 (d, *J* = 3.6 Hz, 1H, *cis*), 3.13 (d, *J* = 2.6 Hz, 1H, *trans*), 2.88 (dd, *J* = 2.6, 0.8 Hz, 1H, *trans*), 2.69 (d, *J* = 3.5 Hz, 1H, *cis*), 1.84 (s, 3H, *cis*), 1.64 (s, 3H, *trans*).
^{13}C NMR (101 MHz, $CDCl_3$) (δ, ppm): 166.4, 141.7, 134.2, 133.8, 130.8, 129.3, 128.6, 128.5, 128.1, 127.7, 127.6, 123.2, 122.8, 48.4, 43.1, 40.3, 24.0, 20.3.

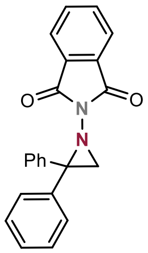

2-(2,2-diphenylaziridin-1-yl)isoindoline-1,3-dione (2r)
Following General Procedure A, prepared from 0.50 mmol of **AT1** and 1.2 equiv. of 1,1-Diphenylethylene (**1r**) in 24 h, the title compound was isolated via flash column chromatography (0–5% EtOAc/Hexanes) as a white solid (126 mg, 74% yield).
^{1}H NMR (400 MHz, $CDCl_3$) (δ, ppm): 7.74 – 7.68 (m, 2H), 7.66 – 7.55 (m, 4H), 7.41 – 7.35 (m, 2H), 7.35 – 7.28 (m, 3H), 7.20 – 7.11 (m, 3H), 4.70 (d, *J* = 3.2 Hz, 1H), 2.90 (d, *J* = 3.2 Hz, 1H).
^{13}C NMR (101 MHz, $CDCl_3$) (δ, ppm): 166.1, 141.4, 134.8, 133.9, 130.6, 128.6, 128.5, 128.1, 127.6, 122.8, 55.1, 40.5.
HRMS (ESI-TOF): *m/z* calculated for $C_{18}H_{14}N_2O_2$ $[M]^+$ = 340.1212, found 340.12.

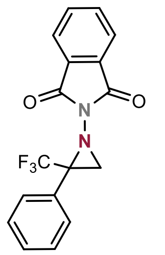

2-(2-phenyl-2-(trifluoromethyl)aziridin-1-yl)isoindoline-1,3-dione (2s)
Following General Procedure A, prepared from 0.50 mmol of **AT1** and 1.2 equiv. of 3,3,3-trifluoro-2-phenylpropene (**1s**) in 24, the title compound was isolated via flash column chromatography (0–20% EtOAc/Hexanes) as a white solid (75 mg, 45% yield) (contains an isomer in a 1:0.1 ratio, isomer 1: isomer 2).
TLC (SiO_2) R_f = 0.28 in 8.7:1:0.2:0.1 Hexanes:EtOAc:Et_3N:DCM
^{1}H NMR (500 MHz, $CDCl_3$) (δ, ppm): 7.91 (d, *J* = 7.8 Hz, 2H, isomer 2), 7.75 (dd, *J* = 5.4, 3.0 Hz, 2H, isomer 2), 7.66 (dd, *J* = 5.6, 3.1 Hz, 2H, isomer 1), 7.64 – 7.60 (m, 2H, isomer 1), 7.59 – 7.50 (m, 2H, isomer 1), 7.45 (d, *J* = 7.1 Hz, 2H, isomer 2), 7.36 – 7.26 (m, 3H, isomer 1), 4.58 (dd, *J* = 3.2, 1.7 Hz, 1H, isomer 1), 4.36 (d, *J* = 3.1 Hz, 1H, isomer 2), 3.20 (d, *J* = 3.1 Hz, 1H, isomer 1), 3.03 – 2.90 (m, 1H, isomer 2).
^{13}C NMR (126 MHz, $CDCl_3$) (δ, ppm): 165.8, 134.5, 134.4, 131.5, 130.5, 128.9, 128.7, 127.1, 124.9, 122.7, 50.8 (d, *J* = 35.4 Hz), 34.6.
^{19}F NMR (471 MHz, $CDCl_3$) (δ, ppm): -65.9, -71.0.
HRMS (ESI-TOF): *m/z* calculated for $C_{17}H_{11}F_3N_2O_2$ $[M]^+$ = 332.0773, found 332.0778.

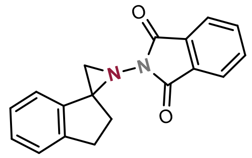

2-(2',3'-dihydrospiro[aziridine-2,1'-inden]-1-yl)isoindoline-1,3-dione (2t)
Following General Procedure A, prepared from 0.50 mmol of **AT1** and 1.2 equiv. of 1-methylene-2,3-dihydro-1*H*-indene (**1t**) in 24 h, the title compound was isolated via column chromatography (1:15:84, Et_3N:EtOAc:Hexanes) as a white solid (131 mg, 90% yield).
TLC (SiO_2) R_f: 0.28 in 1:15:84 Et_3N:EtOAc:Hexanes.
^{1}H NMR (500 MHz, $CDCl_3$) (δ, ppm): 7.86 (dd, *J* = 5.4, 3.1 Hz, 2H), 7.74 (dd, *J* = 5.5, 3.0 Hz, 2H), 7.72 – 7.68 (m, 1H), 7.47 (dt, *J* = 7.5, 0.9 Hz, 1H), 7.35 (td, *J* = 7.5, 1.0 Hz, 1H), 7.23 (td, *J* = 7.5, 1.1 Hz, 1H), 6.60 (t, *J* = 1.7 Hz, 1H), 4.81 (t, *J* = 6.3 Hz, 1H), 4.26 – 4.21 (m, 2H), 3.38 (d, *J* = 2.0 Hz, 2H).
^{13}C NMR (101 MHz, $CDCl_3$) δ 166.2, 143.9, 143.6, 139.0, 134.0, 132.2, 130.1, 126.1, 124.8, 123.6, 123.2, 119.4, 48.6, 37.8.
HRMS (ESI-TOF): *m/z* calculated for $C_{18}H_{14}N_2O_2$ $[M]^+$ = 290.1055, found 290.1048.

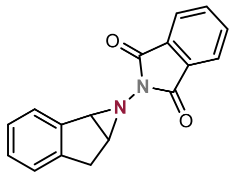

2-(6,6a-dihydroindeno[1,2-*b*]azirin-1(1a*H*)-yl)isoindoline-1,3-dione (2u)

Following General Procedure A, prepared from 0.50 mmol of (*Z*)-2-(1,3-dioxoisoindolin-2-yl)-1-phenyldiazene 1-oxide and 1.2 equiv. of 1*H*-indene (**1u**) in 24 h, the title compound was isolated via flash column chromatography (0–5% EtOAc/Hexanes) as a white solid (100 mg, 72% yield). Analytical data for **2u** was in accordance with literature data.[6]

^{1}H NMR (500 MHz, CD_2Cl_2) (δ, ppm): 7.78 (dd, *J* = 5.4, 3.1 Hz, 2H), 7.71 (dd, *J* = 5.4, 3.1 Hz, 2H), 7.67 – 7.64 (m, 1H), 7.30 – 7.23 (m, 3H), 4.14 (d, *J* = 5.3 Hz, 1H), 3.78 (t, *J* = 5.0 Hz, 1H), 3.42 (d, *J* = 17.7 Hz, 1H), 3.28 (dd, *J* = 17.7, 4.8 Hz, 1H).

^{13}C NMR (101 MHz, $CDCl_3$) (δ, ppm): 165.3, 143.3, 139.0, 134.2, 130.6, 128.8, 127.0, 125.7, 125.5, 123.2, 53.5, 49.0, 35.2.

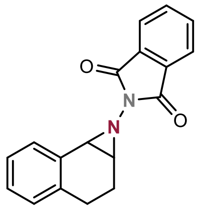

2-(1a,2,3,7b-tetrahydro-1*H*-naphtho[1,2-*b*]azirin-1-yl)isoindoline-1,3-dione (2v)

Following General Procedure A, prepared from 0.50 mmol of **AT1** and 1.2 equiv. of 1,2-dihydronaphthalene (**1v**) in 24 h, the title compound was isolated via flash column chromatography (0–5% EtOAc/Hexanes) as a white solid (118 mg, 81% yield). Analytical data for **2v** was in accordance with literature data.[2]

^{1}H NMR (400 MHz, CD_2Cl_2) (δ, ppm): 7.81 – 7.75 (m, 2H), 7.74 – 7.68 (m, 2H), 7.56 (dd, *J* = 5.3, 3.6 Hz, 1H), 7.32 – 7.22 (m, 2H), 7.19 – 7.07 (m, 1H), 3.52 (d, *J* = 7.6 Hz, 1H), 3.44 – 3.37 (m, 1H), 2.90 – 2.77 (m, 1H), 2.74 – 2.59 (m, 2H), 1.87 – 1.74 (m, 1H).

^{13}C NMR (101 MHz, CD_2Cl_2) (δ, ppm): 165.5, 136.8, 134.4, 132.0, 131.0, 130.2, 128.9, 128.4, 126.7, 123.2, 46.5, 46.1, 25.6, 20.2.

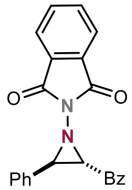

2-((2*S*,3*R*)-2-benzoyl-3-phenylaziridin-1-yl)isoindoline-1,3-dione (2w)

Following General Procedure A, prepared from 0.50 mmol of **AT1** and 1.2 equiv. of *E*-chalcone (**1w**) in 24 h, the title compound was isolated via column chromatography (0–7% EtOAc/Hexanes) as a white solid (122 mg, 66% yield).

TLC (SiO_2) R_f = 0.20 in 9:1 Hexanes:EtOAc.

^{1}H NMR (400 MHz, $CDCl_3$) (δ, ppm): 8.18 – 8.05 (m, 2H), 7.76 – 7.72 (m, 2H), 7.66 (dd, *J* = 5.5, 3.1 Hz, 2H), 7.64 – 7.59 (m, 1H), 7.56 – 7.49 (m, 4H), 7.44 – 7.35 (m, 3H), 4.69 (d, *J* = 4.9 Hz, 1H), 4.39 (d, *J* = 4.8 Hz, 1H).

^{13}C NMR (101 MHz, $CDCl_3$) (δ, ppm): 190.7, 164.8, 137.5, 135.3, 134.1, 133.8, 130.4, 128.94, 128.91, 128.84, 128.75, 127.4, 123.3, 50.8, 48.9.

HRMS (ESI-TOF): *m/z* calculated for $C_{23}H_{16}N_2O_3$ $[M]^+$ = 368.1161, found 368.1159.

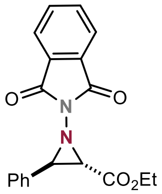

ethyl (2*S*,3*R*)-1-(1,3-dioxoisoindolin-2-yl)-3-phenylaziridine-2-carboxylate (2x)
Following General Procedure A, prepared from 0.50 mmol of **AT1** and 1.2 equiv. of ethyl cinnamate (**1x**) in 24 h, the title compound was isolated via column chromatography (0–8% EtOAc/Hexanes) as a white solid (135 mg, 80% yield).
TLC (SiO_2) R_f = 0.20 in 9:1 Hexanes:EtOAc.
^{1}H NMR (400 MHz, $CDCl_3$) (δ, ppm): 7.78 (dd, *J* = 5.5, 3.0 Hz, 2H), 7.68 (dd, *J* = 5.5, 3.1 Hz, 2H), 7.47 (dd, *J* = 8.1, 1.7 Hz, 2H), 7.42 – 7.34 (m, 3H), 4.39 (d, *J* = 5.0 Hz, 1H), 4.19 (q, *J* = 7.0 Hz, 2H), 3.51 (d, *J* = 5.0 Hz, 1H), 1.28 (t, *J* = 7.2 Hz, 3H).
^{13}C NMR (101 MHz, $CDCl_3$) (δ, ppm): 166.7, 165.1, 135.0, 134.5, 130.6, 129.1, 129.1, 127.7, 123.6, 62.6, 50.0, 46.8, 14.4.
HRMS (ESI-TOF): *m/z* calculated for $C_{19}H_{16}N_2O_4$ $[M]^+$ = 336.1110, found 336.1102.

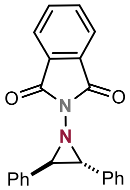

2-((2R,3R)-2,3-diphenylaziridin-1-yl)isoindoline-1,3-dione (2y)
Following General Procedure A, prepared from 0.10 mmol of **AT1** and 1.2 equiv. of *trans*-stilbene (**1y**) in 24 h. The title compound resulted in no reaction.

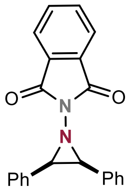

2-((2R,3S)-2,3-diphenylaziridin-1-yl)isoindoline-1,3-dionedione (2z)
Following General Procedure A, prepared from 0.10 mmol of **AT1** and 1.2 equiv. of *cis*-stilbene (**1z**) in 24 h. The title compound resulted in no reaction.

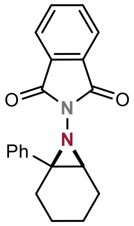

2-(1-phenyl-7-azabicyclo[4.1.0]heptan-7-yl)isoindoline-1,3-dione (2aa)
Following General Procedure A, prepared from 0.50 mmol of **AT1** and 1.2 equiv. of 2,3,4,5-tetrahydro-1,1'-biphenyl (**1aa**) in 24 h, the title compound was isolated via column chromatography (0–7% EtOAc/Hexanes) as a white solid (127 mg, 80% yield).

TLC (SiO_2) R_f = 0.20 in 9:1 Hexanes:EtOAc.
^{1}H NMR (400 MHz, $CDCl_3$) (δ, ppm): 7.62 – 7.49 (m, 4H), 7.41 (dd, J = 7.5, 1.7 Hz, 2H), 7.19 (t, J = 7.4 Hz, 2H), 7.16 – 7.11 (m, 1H), 4.40 (dd, J = 5.5, 1.5 Hz, 1H), 2.87 (ddd, J = 14.3, 7.2, 5.3 Hz, 1H), 2.38 – 2.27 (m, 1H), 2.22 – 2.01 (m, 1H), 1.71 – 1.54 (m, 2H), 1.40 (m, J = 34.3, 13.7, 6.5, 3.2 Hz, 2H)
^{13}C NMR (101 MHz, $CDCl_3$) (δ, ppm): ^{13}C NMR (126 MHz, $CDCl_3$) δ 166.4, 137.8, 134.0, 129.4, 128.4, 128.2, 122.9, 53.6, 44.2, 30.5, 24.2, 21.2, 20.1.
HRMS (ESI-TOF): *m/z* calculated for $C_{20}H_{18}N_2O_2$ $[M]^+$ = 318.1368, found 318.1377.

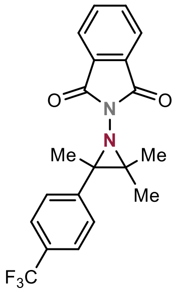

2-(2,2,3-trimethyl-3-(4-(trifluoromethyl)phenyl)aziridin-1-yl)isoindoline-1,3-dione (2ab)
Following General Procedure A, prepared from 0.25 mmol of **AT1** and 1.2 equiv. of 1-(3-methylbut-2-en-2-yl)-4-(trifluoromethyl)benzene (**1ab**) in 24 h, the title compound was isolated via column chromatography (0–10% EtOAc/Hexanes) as a yellow solid (53 mg, 57% yield).
TLC (SiO_2) R_f = 0.25 in 9:1 Hexanes:EtOAc.
^{1}H NMR (500 MHz, $CDCl_3$) (δ, ppm): 7.82 (dd, J = 5.4, 3.1 Hz, 2H), 7.72 (dd, J = 5.4, 3.1 Hz, 2H), 7.67 (d, J = 8.0 Hz, 2H), 7.61 (d, J = 8.2 Hz, 2H), 1.58 (s, 3H), 1.46 (s, 3H), 1.15 (s, 3H).
^{13}C NMR (101 MHz, $CDCl_3$) (δ, ppm): 166.84, 146.57, 134.19, 131.56, 129.16 (d, J = 32.3 Hz), 128.17, 125.34 (q, J = 4.0 Hz), 123.11, 55.41, 50.75, 23.86, 18.52, 17.77. CF_3 quartet buried.
^{19}F NMR (471 MHz, $CDCl_3$) (δ, ppm): -62.36.
HRMS (ESI-TOF): *m/z* calculated for $C_{20}H_{17}F_3N_2O_2$ $[M]^+$ = 374.1242, found 374.1241.

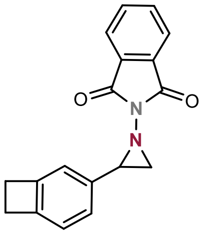

2-(2-(bicyclo[4.2.0]octa-1(6),2,4-trien-3-yl)aziridin-1-yl)isoindoline-1,3-dione (2ac)
Following General Procedure A, prepared from 0.50 mmol of **AT1** and 1.2 equiv. of 4-vinyl benzocyclobutene (**1ac**) in 24 h, the title compound was isolated via flash column chromatography (0–5% EtOAc/Hexanes) as a white solid (125 mg, 86% yield).
TLC (SiO_2) R_f = 0.20 in 8:2 Hexanes:EtOAc.
^{1}H NMR (400 MHz, $CDCl_3$) (δ, ppm): 7.79 (dd, J = 5.4, 3.1 Hz, 2H), 7.69 (dd, J = 5.5, 3.0 Hz, 2H), 7.29 (dd, J = 7.6, 1.5 Hz, 1H), 7.17 (t, J = 1.3 Hz, 1H), 7.05 (dd, J = 7.5, 1.0 Hz, 1H), 3.57 (dd, J = 7.9, 5.9 Hz, 1H), 3.17 (s, 4H), 2.87 (dd, J = 7.9, 2.5 Hz, 1H), 2.79 (dd, J = 5.9, 2.5 Hz, 1H).
^{13}C NMR (101 MHz, $CDCl_3$) (δ, ppm): 165.2, 146.2, 145.9, 135.0, 134.2, 130.6, 126.2, 123.2, 122.7, 121.5, 45.4, 39.9, 29.6, 29.5.
HRMS (ESI-TOF): *m/z* calculated for $C_{18}H_{14}N_2O_2$ $[M]^+$ = 290.1055, found 290.1070.

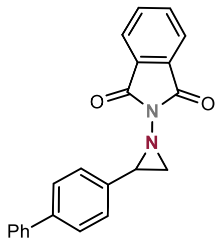

2-(2-([1,1'-biphenyl]-4-yl)aziridin-1-yl)isoindoline-1,3-dione (2ad)

Following General Procedure A, prepared from 0.50 mmol of **AT1** and 1.2 equiv. of 4-vinyl-1,1'-biphenyl (**1ad**) in 24 h, the title compound was isolated via flash column chromatography (0–5% EtOAc/Hexanes) as a white solid (136 mg, 80% yield).

TLC (SiO_2) R_f = 0.20 in 8:2 Hexanes:EtOAc.

^{1}H NMR (500 MHz, $CDCl_3$) (δ, ppm): 7.81 (dd, *J* = 5.4, 3.1 Hz, 2H), 7.71 (dd, *J* = 5.4, 3.0 Hz, 2H), 7.61 (t, *J* = 7.3 Hz, 4H), 7.53 (d, *J* = 8.2 Hz, 2H), 7.45 (t, *J* = 7.7 Hz, 2H), 7.35 (t, *J* = 7.3 Hz, 1H), 3.65 (dd, *J* = 7.9, 5.9 Hz, 1H), 2.94 (dd, *J* = 8.0, 2.5 Hz, 1H), 2.85 (dd, *J* = 5.9, 2.5 Hz, 1H).

^{13}C NMR (101 MHz, $CDCl_3$) (δ, ppm): 165.2, 141.2, 141.0, 135.6, 134.3, 130.5, 128.9, 127.8, 127.49, 127.48, 127.3, 123.3, 44.5, 40.0.

HRMS (ESI-TOF): *m/z* calculated for $C_{20}H_{18}N_2O_2$ $[M]^+$ = 340.1212, found 340.1207.

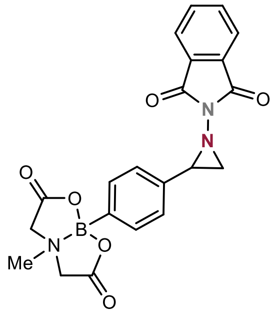

8-(4-(1-(1,3-dioxoisoindolin-2-yl)aziridin-2-yl)phenyl)-4-methyldihydro-4λ4,8λ4-[1,3,2]oxazaborolo[2,3-*b*][1,3,2]oxazaborole-2,6(3*H*,5*H*)-dione (2ae)

Following General Procedure A, prepared from 0.28 mmol of **AT1** and 1.2 equiv. of 4-methyl-8-(4-vinylphenyl)dihydro-4λ4,8λ4-[1,3,2]oxazaborolo[2,3-*b*][1,3,2]oxazaborole-2,6(3*H*,5*H*)-dione (**1ae**) in 24 h, the title compound was isolated via recrystallization (1:1, DCM/MeOH) as a pale white solid (49 mg, 42% yield).

^{1}H NMR (500 MHz, CD_3CN) (δ, ppm): 7.76 (q, *J* = 1.1 Hz, 4H), 7.55 – 7.50 (m, 2H), 7.43 (d, *J* = 8.1 Hz, 2H), 4.07 (d, *J* = 17.0 Hz, 2H), 3.91 (dd, *J* = 17.1, 2.8 Hz, 2H), 3.64 – 3.56 (m, 1H), 2.91 (dd, *J* = 8.0, 2.3 Hz, 1H), 2.75 (dd, *J* = 5.8, 2.3 Hz, 1H), 2.53 (s, 3H).

^{13}C NMR (101 MHz, CD_2Cl_2) (δ, ppm): 167.5, 165.4, 138.9, 134.6, 132.93, 132.89, 130.8, 127.4, 123.3, 62.3, 48.0, 44.8, 40.4.

HRMS (ESI-TOF): *m/z* calculated for $C_{21}H_{18}BN_3O_6$ $[M]^+$ = 418.1325, found 418.1344.

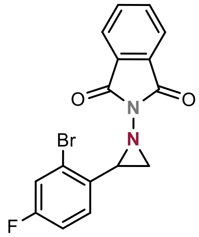

2-(2-(2-bromo-4-fluorophenyl)aziridin-1-yl)isoindoline-1,3-dione (2af)
Following General Procedure A, prepared from 0.50 mmol of **AT1** and 1.2 equiv. of 2-bromo-4-fluoro-1-vinylbenzene (**1af**) in 24 h, the title compound was isolated via crystallization using (1:0.25:0.25 mixture of Hexane/EtOAc/DCM) and recrystallization with hexane as a pale white solid (110 mg, 60% yield).
^{1}H NMR (500 MHz, CD_2Cl_2) (δ, ppm): 7.80 (dd, *J* = 5.5, 3.1 Hz, 2H), 7.73 (dd, *J* = 5.4, 3.1 Hz, 2H), 7.58 (dd, *J* = 8.7, 6.0 Hz, 1H), 7.36 (dd, *J* = 8.3, 2.6 Hz, 1H), 7.10 (td, *J* = 8.4, 2.6 Hz, 1H), 3.71 (dd, *J* = 8.0, 5.7 Hz, 1H), 3.02 (dd, *J* = 8.0, 2.1 Hz, 1H), 2.50 (dd, *J* = 5.7, 2.1 Hz, 1H).
^{13}C NMR (126 MHz, CD_2Cl_2) (δ, ppm): 165.3, 163.2, 161.3, 134.6, 133.3 (d, *J* = 3.5 Hz), 130.7, 130.1 (d, *J* = 8.7 Hz), 123.6 (d, *J* = 9.6 Hz), 123.4, 119.8 (d, *J* = 24.8 Hz), 115.2 (d, *J* = 21.1 Hz), 44.4, 40.88.
^{19}F NMR (471 MHz, CD_2Cl_2) (δ, ppm): -113.23.
HRMS (ESI-TOF): *m/z* calculated for $C_{16}H_{10}BrFN_2O_2$[M] = 359.9910, found 359.9913.

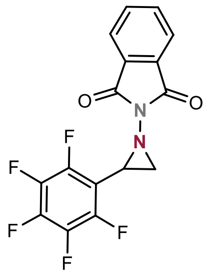

2-(2-(perfluorophenyl)aziridin-1-yl)isoindoline-1,3-dione (2ag)
Following General Procedure A, prepared from 0.50 mmol of **AT1** and 1.2 equiv. of 1,2,3,4,5-pentafluoro-6-vinylbenzene (**1ag**) in 24 h, the title compound was isolated via Davisil column chromatography (0–35% EtOAc/Hexanes) as a yellow solid (80 mg, 45% yield).
TLC (SiO_2) R_f = 0.25 in 7:3 Hexanes:EtOAc.
^{1}H NMR (500 MHz, $CDCl_3$) (δ, ppm): 7.81 (dd, *J* = 5.4, 3.1 Hz, 2H), 7.72 (dd, *J* = 5.5, 3.0 Hz, 2H), 3.84 (dd, *J* = 8.1, 5.7 Hz, 1H), 3.17 (dd, *J* = 5.7, 2.3 Hz, 1H), 3.10 (dd, *J* = 8.2, 2.3 Hz, 1H).
^{13}C NMR (126 MHz, $CDCl_3$) (δ, ppm): 165.3, 163.2, 161.3, 134.6, 133.3 (d, J = 3.5 Hz), 130.7, 130.1 (d, J = 8.7 Hz), 123.4, 119.8 (d, J = 24.8 Hz), 115.2 (d, J = 21.1 Hz), 44.4, 40.9.
^{19}F NMR (471 MHz, $CDCl_3$) (δ, ppm) -143.1 – -143.31 (m), -153.87 (t, *J* = 20.8 Hz), -161.93 (td, *J* = 21.7, 21.2, 7.4 Hz).
HRMS (ESI-TOF): *m/z* calculated for $C_{16}H_7F_5N_2O_2$ $[M]^+$ = 354.0428, found = 354.0416.

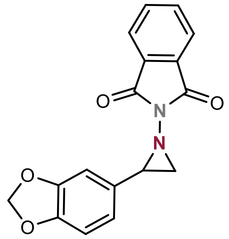

2-(2-(benzo[d][1,3]dioxol-5-yl)aziridin-1-yl)isoindoline-1,3-dione (2ah)
Following General Procedure A, prepared from 0.25 mmol of **AT1** and 1.2 equiv. of (4-methylpent-1-ene-2,4-diyl)dibenzene (**1ah**) in 24 h. ^{1}H NMR yield was determined using CH_2Br_2 as an external standard (70% NMR yield). Isolation was proven difficult due to the sensitivity of the compound to column chromatography.
^{1}H NMR (crude) (500 MHz, $CDCl_3$) (δ, ppm): 2.83 (dd, *J* = 8.0, 2.5 Hz, 1H, aziridine proton), 2.74 (dd, *J* = 5.9, 2.6 Hz, 1H, aziridine proton).
HRMS (ESI-TOF): *m/z* calculated for $C_{17}H_{12}N_3O_4$ $[M]^+$ = 308.0797, found = 308.0795.

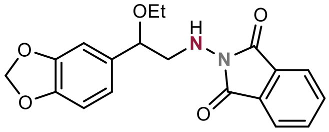

2-((2-(benzo[d][1,3]dioxol-5-yl)-2-ethoxyethyl)amino)isoindoline-1,3-dione (2ah1)
Owing to the rapid aziridine ring opening nature of **2ah** upon exposure to silica gel, *in situ* derivatization was performed by heating the aziridine **2ah** (0.20 mmol, 55 mg) in ethanol (8ml) and silica gel (1eq.) at 100 °C. The title compound was isolated via silica gel chromatography (2:10:88 Et_3N:EtOAc:Hexanes) as a brown solid (55 mg, 67% overall yield).
(70% NMR yield).
TLC (SiO_2) R_f = 0.3 in 2:10:88 Et_3N:EtOAc:Hexanes.
^{1}H NMR (crude) (500 MHz, $CDCl_3$) (δ, ppm): 7.85 (dd, *J* = 5.4, 3.1 Hz, 2H), 7.73 (dd, *J* = 5.5, 3.12Hz, 2H), 6.82 (d, *J* = 1.6 Hz, 1H), 6.80 – 6.71 (m, 2H), 5.96 – 5.90 (m, 2H), 5.12 (dd, *J* = 8.5, 3.3 Hz, 1H), 4.47 (dd, *J* = 9.5, 3.2 Hz, 1H), 3.43 – 3.28 (m, 3H), 3.00 (ddd, *J* = 12.2, 8.4, 3.2 Hz, 1H), 1.09 (t, *J* = 7.0 Hz, 3H).
^{13}C NMR (126 MHz, $CDCl_3$) (δ, ppm): 166.6, 148.0, 147.4, 134.3, 134.1, 130.6, 123.5, 120.4, 108.3, 107.0, 101.2, 80.2, 64.3, 57.3, 15.
HRMS (ESI-TOF): *m/z* calculated for $C_{19}H_{18}N_2O_5$ $[M^+]$ = 354.1216, found 354.1232.

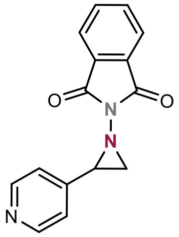

2-(2-(pyridin-4-yl)aziridin-1-yl)isoindoline-1,3-dione (2ai)
Following General Procedure A, prepared from 0.50 mmol of **AT1** and 1.2 equiv. of 4-vinylpyridine (**1ai**) in 24 h, the title compound was isolated via column chromatography (2.5:20:77.5 Et_3N:EtOAc:Hexanes) as a pale white solid (57 mg, 43% yield).
TLC (SiO_2) R_f = 0.28 in 2.5:20:77.5 Et_3N:EtOAc:Hexanes.
^{1}H NMR (500 MHz, $CDCl_3$) (δ, ppm): 8.62 – 8.59 (m, 2H), 7.81 (dd, *J* = 5.4, 3.1 Hz, 2H), 7.72 (dd, *J* = 5.5, 3.1 Hz, 2H), 7.38 – 7.33 (m, 2H), 3.56 (dd, *J* = 8.0, 5.6 Hz, 1H), 2.98 (dd, *J* = 8.0, 2.4 Hz, 1H), 2.74 (dd, *J* = 5.6, 2.4 Hz, 1H).
^{13}C NMR (126 MHz, $CDCl_3$) (δ, ppm): 165.0, 150.1, 145.8, 134.5, 130.3, 123.4, 122.1, 43.1, 40.3.
HRMS (ESI-TOF): *m/z* calculated for $C_{15}H_{11}N_3O_2$ $[M]^+$ = 265.0851, found 265.0862.

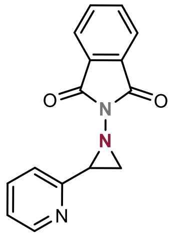

2-(2-(pyridin-2-yl)aziridin-1-yl)isoindoline-1,3-dione (2aj)

Following General Procedure A, prepared from 0.50 mmol of **AT1** 1.2 equiv. of 2-vinylpyridine (**1aj**) in 24 h, the title compound was isolated via column chromatography (2.5:20:77.5 Et_3N:EtOAc:Hexanes) as a white solid (109 mg, 82% yield).

TLC (SiO_2) R_f = 0.28 in 2.5:20:77.5 Et_3N:EtOAc:Hexanes.

^{1}H NMR (500 MHz, $CDCl_3$) (δ, ppm): 8.59 (dt, *J* = 4.9, 1.3 Hz, 1H), 7.85 – 7.77 (m, 2H), 7.75 – 7.68 (m, 3H), 7.61 (dt, *J* = 7.8, 1.1 Hz, 1H), 7.25 – 7.22 (m, 1H), 3.71 (dd, *J* = 7.9, 5.7 Hz, 1H), 3.06 (dd, *J* = 5.8, 2.1 Hz, 1H), 2.95 (dd, *J* = 7.9, 2.1 Hz, 1H).

^{13}C NMR (101 MHz, $CDCl_3$) (δ, ppm): 164.8, 155.5, 149.3, 136.5, 134.0, 130.2, 123.0, 122.8, 122.6, 45.0, 38.9.

HRMS (ESI-TOF): *m/z* calculated for $C_{15}H_{11}N_3O_2$ $[M+H]^+$ = 265.0851, found 265.0857.

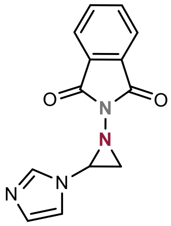

2-(2-(1*H*-imidazol-1-yl)aziridin-1-yl)isoindoline-1,3-dione (2ak)

Following General Procedure A, prepared from 0.50 mmol of **AT4** and 1.2 equiv. of vinyl imidazole (**1ak**) in 24 h, the title compound was isolated via crystallizing the phthalimide impurities at -70 °C and running a quick silica gel preparatory TLC at (1:94:5 Et_3N/DCM/MeOH). The desired compound was collected as an orange solid (22 mg, 34% yield).

TLC (SiO_2) R_f = 0.45 in 1:96:5 Et_3N:DCM:MeOH.

^{1}H NMR (600 MHz, $CDCl_3$) (δ, ppm): 7.88 – 7.80 (m, 3H), 7.75 (dd, *J* = 5.5, 3.1 Hz, 2H), 7.33 (d, *J* = 1.4 Hz, 1H), 7.12 (d, *J* = 1.2 Hz, 1H), 4.78 (dd, *J* = 6.8, 4.6 Hz, 1H), 3.19 – 3.14 (m, 1H), 3.05 (dd, *J* = 6.8, 3.6 Hz, 1H).

^{13}C NMR (126 MHz, $CDCl_3$) (δ, ppm): 164.9, 137.0, 134.7, 130.2, 123.7, 118.7, 90.9, 51.3, 37.3.

HRMS (ESI-TOF): *m/z* calculated for $C_{13}H_{10}N_4O_2$ [M] = 254.0804, found 254.0812.

Characterization of Aziridination Products from Unactivated Alkenes

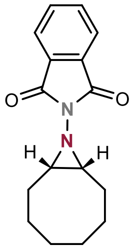

2-(9-azabicyclo[6.1.0]nonan-9-yl)isoindoline-1,3-dione (2al)
Following General Procedure B, prepared from 0.25 mmol of **AT1** and 2.0 equiv. of *cis*-cyclooctene (**1al**) in 24 h, the title compound was isolated via column chromatography (0–15% EtOAc/Hexanes) as a white solid (48 mg, 71% yield). Analytical data for **2al** was in accordance with literature data.[7]
TLC (SiO_2) R_f = 0.21 in 9:1 Hexanes:EtOAc.
^{1}H NMR (500 MHz, $CDCl_3$) (δ, ppm): δ 7.74 (dd, *J* = 5.4, 3.1 Hz, 2H), 7.65 (dd, *J* = 5.5, 3.1 Hz, 2H), 2.64 – 2.42 (m, 4H), 1.71 – 1.62 (m, 2H), 1.60 – 1.52 (m, 2H), 1.50 – 1.42 (m, 4H), 1.40 – 1.33 (m, 2H).
^{13}C NMR (126 MHz, $CDCl_3$) (δ, ppm): 165.5, 134.3, 131.0, 123.2, 48.4, 26.9, 26.8, 25.8.

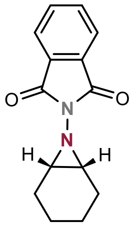

2-(7-azabicyclo[4.1.0]heptan-7-yl)isoindoline-1,3-dione (2am)
Following General Procedure A, prepared from 0.50 mmol of **AT1** and 2 equiv. of cyclohexene (**1am**) in 24 h, the title compound was isolated via column chromatography (0–15% EtOAc/Hexanes) as a white solid (48 mg, 40% yield). Analytical data for **2am** was in accordance with literature data.[4]
TLC (SiO_2) R_f = 0.28 in 8:1.9:0.1 Hexanes:EtOAc:Et_3N
^{1}H NMR (500 MHz, $CDCl_3$) (δ, ppm): 7.74 (dd, *J* = 5.4, 3.1 Hz, 2H), 7.66 (dd, *J* = 5.5, 3.1 Hz, 2H), 2.73 (dd, *J* = 3.2, 1.5 Hz, 2H), 2.30 – 2.21 (m, 2H), 2.02 – 1.92 (m, 2H), 1.48 – 1.37 (m, 2H), 1.35 – 1.23 (m, 2H).
^{13}C NMR (126 MHz, $CDCl_3$) (δ, ppm): 165.4, 134.0, 130.8, 123.0, 44.1, 23.3, 20.2.
HRMS (ESI-TOF): *m/z* calculated for $C_{14}H_{14}N_2O_2$ $[M]^+$ = 242.1055, found 242.1061.

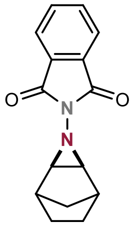

2-(3-azatricyclo[3.2.1.0^{2,4}]octan-3-yl)isoindoline-1,3-dione (2an)
Following General Procedure A, prepared from 0.25 mmol of **AT1** 2.0 equiv. of norbornene (**1an**) in 24 h, the title compound was isolated via column chromatography (0–15% EtOAc/Hexanes) as a white solid (21 mg, 33% yield). Analytical data for **2an** was in accordance with literature data.[7]

TLC (SiO_2) R_f = 0.21 in 9:1 Hexanes:EtOAc.
^{1}H NMR (500 MHz, $CDCl_3$) (δ, ppm): 7.74 (dd, *J* = 5.4, 3.1 Hz, 2H), 7.65 (dd, *J* = 5.5, 3.0 Hz, 2H), 2.79 (d, *J* = 1.2 Hz, 2H), 2.78 – 2.74 (m, 2H), 1.60 (dt, *J* = 10.1, 2.2 Hz, 1H), 1.50 (dq, *J* = 7.6, 1.8 Hz, 2H), 1.31 – 1.23 (m, 2H), 0.80 (dt, *J* = 10.1, 1.2 Hz, 1H).
^{13}C NMR (126 MHz, $CDCl_3$) (δ, ppm): 165.5, 134.0, 130.6, 123.0, 45.3, 36.4, 27.9, 26.3.

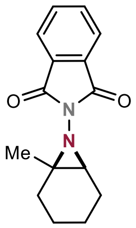

2-(1-methyl-7-azabicyclo[4.1.0]heptan-7-yl)isoindoline-1,3-dione (2ao)
Following General Procedure A, prepared from 0.25 mmol of **AT1** and 2.0 equiv. of methyl cyclohexene (**1ao**) in 24 h, the title compound was isolated via column chromatography (2:20:80 Et_3N:EtOAc:Hexanes) as a white solid (39 mg, 61% yield). Analytical data for **2ao** was in accordance with literature data.[7]
TLC (SiO_2) R_f = 0.28 in 2:20:80 Et_3N:EtOAc:Hexanes.
^{1}H NMR (500 MHz, $CDCl_3$) (δ, ppm): 7.75 (dd, *J* = 5.4, 3.1 Hz, 2H), 7.66 (dd, *J* = 5.5, 3.0 Hz, 2H), 2.90 (dd, *J* = 5.7, 1.4 Hz, 1H), 2.30 – 2.11 (m, 2H), 2.02 (dq, *J* = 14.7, 5.9 Hz, 1H), 1.73 – 1.70 (m, 1H), 1.53 – 1.38 (m, 2H), 1.34 (m, 1H), 1.30 (s, 3H), 1.28 – 1.18 (m, 1H).
^{13}C NMR (126 MHz, $CDCl_3$) (δ, ppm): 166.2, 133.7, 130.8, 122.6, 48.3, 47.2, 30.3, 23.6, 20.3, 20.1, 20.0.

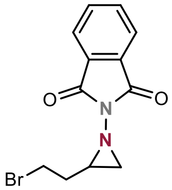

2-(2-(2-bromoethyl)aziridin-1-yl)isoindoline-1,3-dione (2ap)
Following General Procedure A, prepared from 0.25 mmol of **AT1** and 2.0 equiv. of 4-bromo-1-butene (**1ap**) in 24 h, the title compound was isolated via column chromatography (0–5% EtOAc/Hexanes) as an off-white solid (26 mg, 35% yield).
TLC (SiO_2) R_f = 0.11 in 9:1 Hexanes:EtOAc.
^{1}H NMR (400 MHz, $CDCl_3$) (δ, ppm): 7.80 – 7.75 (m, 2H), 7.71 – 7.67 (m, 2H), 3.88 (ddd, *J* = 10.0, 8.1, 6.7 Hz, 1H), 3.73 (ddd, *J* = 10.1, 7.0, 5.5 Hz, 1H), 2.72 (tdd, *J* = 8.0, 5.7, 4.3 Hz, 1H), 2.59 (dd, *J* = 7.7, 2.5 Hz, 1H), 2.39 (dd, *J* = 5.7, 2.5 Hz, 1H), 2.28 (dddd, *J* = 15.0, 8.2, 7.0, 4.2 Hz, 1H), 2.16 – 2.03 (m, 1H).
^{13}C NMR (101 MHz, $CDCl_3$) (δ, ppm): 165.3, 134.3, 130.4, 123.3, 42.1, 37.7, 35.8, 30.1.
HRMS (ESI-TOF): *m/z* calculated for $C_{12}H_{11}BrN_2O_2$ $[M]^+$ = 294.0004, found = 294.0.

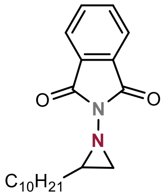

2-(2-decylaziridin-1-yl)isoindoline-1,3-dione (2aq)

Following General Procedure A, prepared from 0.50 mmol of **AT1** and 2.0 equiv. of 1-dodecene (**1aq**) in 24 h, the title compound was isolated via Davisil column chromatography (0–5% EtOAc/Hexanes) as an off-white solid (67 mg, 41% yield).
TLC (SiO_2) R_f = 0.23 in 9:1 Hexanes:EtOAc.
^{1}H NMR (400 MHz, $CDCl_3$) (δ, ppm): 7.78 – 7.75 (m, 2H), 7.69 – 7.65 (m, 2H), 2.64 – 2.55 (m, 1H), 2.44 (dd, *J* = 7.7, 2.3 Hz, 1H), 2.28 (dd, *J* = 5.8, 2.3 Hz, 1H), 1.79 – 1.54 (m, 4H), 1.41–1.22 (m, 14H), 0.94 – 0.83 (m, 3H).
^{13}C NMR (101 MHz, $CDCl_3$) (δ, ppm): 165.4, 134.1, 130.6, 123.1, 43.5, 38.4, 32.2, 32.1, 29.8, 29.7, 29.5, 26.6, 22.9, 14.3.
HRMS (ESI-TOF): *m/z* calculated for $C_{20}H_{28}N_2O_2$ $[M]^+$ = 328.2151, found 328.2158.

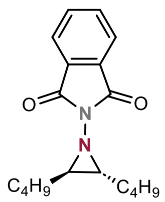

2-((2*S*,3*S*)-2,3-dibutylaziridin-1-yl)isoindoline-1,3-dione (2ar)
Following General Procedure A, prepared from 0.25 mmol of **AT1** and 2 equiv. of *trans*-5-decene (**1ar**) in 24 h, the title compound was isolated via column chromatography (0–10% EtOAc/Hexanes) as a yellow solid (29 mg, 38% yield).
TLC (SiO_2) R_f = 0.24 in 9:1 Hexanes:EtOAc.
^{1}H NMR (400 MHz, $CDCl_3$) (δ, ppm): 7.77 (dd, *J* = 5.4, 3.1 Hz, 2H), 7.68 (dd, *J* = 5.5, 3.1 Hz, 2H), 2.83 (q, *J* = 5.8 Hz, 1H), 2.33 (ddd, *J* = 9.6, 5.6, 4.1 Hz, 1H), 2.01 – 1.92 (m, 1H), 1.85 – 1.75 (m, 1H), 1.65 – 1.23 (m, 9H), 1.05 (dtd, *J* = 13.2, 9.4, 5.8 Hz, 1H), 0.95 (t, *J* = 7.3 Hz, 3H), 0.88 (t, *J* = 7.2 Hz, 3H).
^{13}C NMR (101 MHz, $CDCl_3$) (δ, ppm): 166.51, 134.06, 130.79, 123.02, 49.05, 47.64, 32.26, 29.76, 28.66, 27.63, 22.79, 22.67, 14.16, 14.13.
HRMS (ESI-TOF): *m/z* calculated for $C_{18}H_{24}N_2O_2$ $[M]^+$ = 300.1838, found = 300.1831.

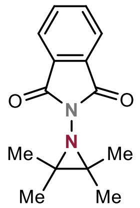

2-(2,2,3,3-tetramethylaziridin-1-yl)isoindoline-1,3-dione (2as)
Following General Procedure A, prepared from 0.25 mmol of **AT1** and 2 equiv. of 2,3-dimethylbutene (**1as**) in 24 h, the title compound was isolated via column chromatography (0–10% EtOAc/Hexanes) as a white solid (60 mg, 98% yield).
TLC (SiO_2) R_f = 0.28 in 9:1 Hexanes:EtOAc.
^{1}H NMR (500 MHz, $CDCl_3$) (δ, ppm): 7.75 (dd, *J* = 5.4, 3.1 Hz, 2H), 7.66 (dd, *J* = 5.5, 3.1 Hz, 2H), 1.44 (s, 3H), 1.27 (s, 3H).
^{13}C NMR (126 MHz, $CDCl_3$) (δ, ppm): 166.6, 133.7, 131.3, 122.6, 49.6, 21.9, 17.8.
HRMS (ESI-TOF): *m/z* calculated for $C_{14}H_{16}N_2O_2$ $[M]^+$ = 244.1212, found = 244.1213.

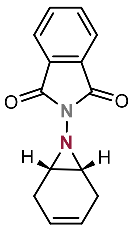

2-(7-azabicyclo[4.1.0]heptan-7-yl)isoindoline-1,3-dione (2at)

Following General Procedure A, prepared from 0.50 mmol of **AT1** and 2.0 equiv. of 1,4-cyclohexadiene (**1at**) in 24 h, the title compound was isolated via column chromatography (0–10% EtOAc/Hexanes) as a yellow solid (60 mg, 50% yield).

TLC (SiO_2) R_f = 0.12 in 9:1 Hexanes:EtOAc.

^{1}H NMR (400 MHz, $CDCl_3$) (δ, ppm): 7.76 (dd, *J* = 5.5, 3.1 Hz, 2H), 7.67 (dd, *J* = 5.4, 3.1 Hz, 2H), 5.58 – 5.54 (m, 2H), 2.88 (tdt, *J* = 7.8, 7.0, 3.1, 1.6 Hz, 4H), 2.56 – 2.46 (m, 2H).

^{13}C NMR (101 MHz, $CDCl_3$) (δ, ppm): 165.3, 134.1, 130.7, 123.1, 122.5, 42.9, 23.5.

HRMS (ESI-TOF): *m/z* calculated for $C_{14}H_{12}N_2O_2$ $[M]^+$ = 240.0899, found 240.0895.

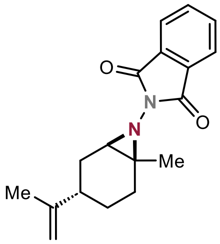

2-((1*R*,3*S*,6*S*)-3-(prop-1-en-2-yl)-7-azabicyclo[4.1.0]heptan-7-yl)isoindoline-1,3-dione (2au)

Following General Procedure A, prepared from 0.50 mmol of **AT1** and 2.0 equiv. of (+)-limonene (**1au**) in 24 h, the title compound was isolated as diastereomers via column chromatography (0–10% EtOAc/Hexanes) as a yellow solid (89 mg, 60% yield, d.r. 50:50). Minor product is the aziridination of the other alkene as 4% NMR yield (**2au1**).

TLC (SiO_2) R_f = 0.23 in 9:1 Hexanes:EtOAc.

^{1}H NMR (500 MHz, $CDCl_3$) (δ, ppm): 7.76 (ddd, *J* = 5.1, 3.1, 1.6 Hz, 4H), 7.67 (ddd, *J* = 4.4, 3.1, 1.2 Hz, 4H), 4.76 – 4.66 (m, 4H), 3.03 (dd, *J* = 3.9, 1.8 Hz, 1H), 2.94 (d, *J* = 7.4 Hz, 1H), 2.49 – 2.40 (m, 2H), 2.32 (ddd, *J* = 14.3, 7.3, 5.2 Hz, 1H), 2.19 (ddd, *J* = 14.8, 11.6, 5.9 Hz, 1H), 2.06 (tt, *J* = 10.9, 3.4 Hz, 1H), 2.01 – 1.85 (m, 4H), 1.79 – 1.63 (m, 9H), 1.50 – 1.41 (m, 2H), 1.31 (d, *J* = 11.3 Hz, 6H).

^{13}C NMR (126 MHz, $CDCl_3$) (δ, ppm): 166.4, 149.4, 134.00, 133.98, 131.0, 123.0, 122.9, 109.3, 109.1, 49.7, 48.5, 47.4, 47.3, 41.1, 37.3, 31.3, 30.1, 29.8, 29.6, 29.3, 26.5, 24.9, 21.3, 20.4, 19.7.

HRMS (ESI-TOF): m/z calculated for $C_{18}H_{20}N_2O_2$ $[M]^+$ = 296.1525, found 296.1518.

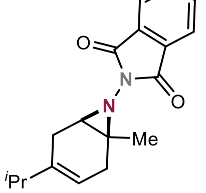

2-(4-isopropyl-1-methyl-7-azabicyclo[4.1.0]hept-3-en-7-yl)isoindoline-1,3-dione (2av)

Following General Procedure A, prepared from 0.50 mmol of **AT1** and 2 equiv. of γ-terpinene (**1av**) in 24 h, the title compound was isolated as diastereomers via column chromatography (0–10%

EtOAc/Hexanes) as a yellow solid (95 mg, 64% yield). Minor product from aziridination of other alkene as 14% NMR yield (**2av1**).
TLC (SiO_2) R_f = 0.18 in 9:1 Hexanes:EtOAc.
^{1}H NMR (400 MHz, $CDCl_3$) (δ, ppm): 7.76 (dd, *J* = 5.4, 3.1 Hz, 2H), 7.67 (dd, *J* = 5.4, 3.1 Hz, 2H), 5.28 (ddt, *J* = 5.3, 2.9, 1.5 Hz, 1H), 3.06 (dd, *J* = 4.3, 1.3 Hz, 1H), 2.85 (d, *J* = 18.2 Hz, 1H), 2.76 (d, *J* = 18.5 Hz, 1H), 2.53 (ddt, *J* = 15.9, 4.5, 2.4 Hz, 1H), 2.33 (ddt, *J* = 18.7, 4.7, 2.3 Hz, 1H), 2.19 (p, *J* = 6.9 Hz, 1H), 1.37 (s, 3H), 1.02 (dd, *J* = 6.8, 5.4 Hz, 6H).
^{13}C NMR (101 MHz, $CDCl_3$) (δ, ppm): 166.4, 138.5, 134.0, 123.0, 114.4, 48.6, 46.5, 34.8, 31.0, 26.2, 21.6, 21.1, 19.3.

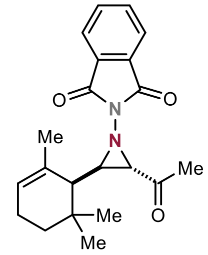

***Trans* 2-(2-acetyl-3-2,6,6-trimethylcyclohex-2-en-1-yl)aziridin-1-yl)isoindoline-1,3-dione (2aw)**
Following General Procedure A, prepared from 0.25 mmol of **AT1** and 2.0 equiv. of α-ionone (**1aw**) in 24 h, the title compound was isolated via Davisil column chromatography (0–15% EtOAc/Hexanes) as a white solid (94 mg, 84% yield).
TLC (SiO_2) R_f = 0.30 in 8:2, EtOAc:Hexanes
^{1}H NMR (400 MHz, $CDCl_3$) (δ, ppm): 7.75 – 7.71 (m, 2H), 7.65 (dd, *J* = 5.5, 3.1 Hz, 2H), 5.52 – 5.42 (m, 1H), 3.68 (dd, *J* = 7.1, 4.9 Hz, 1H), 3.37 (d, *J* = 4.9 Hz, 1H), 2.43 (s, 3H), 2.12 – 2.02 (m, 2H), 1.99 (d, *J* = 6.8 Hz, 1H), 1.72 (q, *J* = 2.0 Hz, 3H), 1.31 – 1.22 (m, 2H), 1.12 (s, 3H), 0.95 (s, 3H).
^{13}C NMR (101 MHz, $CDCl_3$) (δ, ppm): 200.0, 165.0, 133.8, 131.4, 130.0, 123.8, 122.8, 50.1, 49.9, 47.5, 32.7, 31.6, 30.5, 27.4, 26.8, 24.4, 23.1.
HRMS (ESI-TOF): *m/z* calculated for $C_{21}H_{24}N_2O_3$ $[M]^+$ = 352.1787, found = 352.1787.

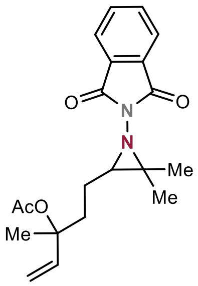

5-(1-(1,3-dioxoisoindolin-2-yl)-3,3-dimethylaziridin-2-yl)-3-methylpent-1-en-3-yl acetate (2ax)
Following General Procedure A, prepared from 0.25 mmol of **AT1** and 2.0 equiv. of linalyl acetate (**1ax**) in 24 h, the title compound was isolated via Davisil column chromatography (0–10% EtOAc/Hexanes) as a white solid (59 mg, 67% yield).
TLC (SiO_2) R_f = 0.25 in 9:1 EtOAc:Hexanes.
^{1}H NMR (400 MHz, $CDCl_3$) (δ, ppm): 7.76 (dd, *J* = 5.4, 3.1 Hz, 2H), 7.67 (dd, *J* = 5.5, 3.0 Hz, 2H), 5.99 (ddd, *J* = 36.2, 17.5, 11.0 Hz, 1H), 5.22 – 5.11 (m, 2H), 2.76 (td, *J* = 6.8, 3.6 Hz, 1H), 2.21 – 2.13 (m, 1H),

2.12 – 2.05 (m, 1H), 2.02 (d, J = 4.1 Hz, 3H), 1.89 – 1.75 (m, 1H), 1.59 (d, J = 16.8 Hz, 4H), 1.40 (d, J = 2.1 Hz, 3H), 1.27 (s, 3H).
^{13}C NMR (101 MHz, $CDCl_3$) (δ, ppm): 170.4, 166.5, 142.0, 141.8, 134.3, 123.2, 113.93, 113.86, 83.1, 82.9, 53.0, 48.1, 37.5, 37.51, 24.0, 24.0, 23.5, 22.6, 21.2, 19.5.
HRMS (ESI-TOF): *m/z* calculated for $C_{20}H_{24}N_2O_4$ $[M]^+$ = 356.1736, found 356.1723.

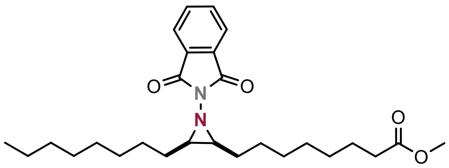

methyl 8-((2S,3R)-1-(1,3-dioxoisoindolin-2-yl)-3-octylaziridin-2-yl)octanoate (2ay)
Following General Procedure A, prepared from 0.25 mmol of **AT1** and 2.0 equiv. of methyl oleate (**1ay**) in 24 h, the title compound was isolated via column chromatography (0–10% EtOAc/Hexanes) as a white solid (84.5 mg, 74% yield).
TLC (SiO_2) R_f = 0.20 in 9:1 Hexanes:EtOAc.
^{1}H NMR (500 MHz, $CDCl_3$) (δ, ppm): 7.74 (dd, J = 5.4, 3.1 Hz, 2H), 7.66 (dd, J = 5.5, 3.1 Hz, 2H), 3.67 (s, 3H), 2.55 (td, J = 4.4, 2.0 Hz, 2H), 2.30 (t, J = 7.6 Hz, 2H), 1.74 (tt, J = 11.5, 5.8 Hz, 4H), 1.66 – 1.59 (m, 4H), 1.34 (m, 10H), 1.29 (m, 8H), 0.97 – 0.84 (m, 3H).
^{13}C NMR (126 MHz, $CDCl_3$) (δ, ppm):174.5, 165.3, 134.0, 130.7, 123.0, 51.6, 49.0, 49.0, 34.3, 32.0, 29.9, 29.7, 29.45, 29.3, 29.3, 27.5, 27.4, 27.3, 27.2, 25.1, 22.8, 14.3.
HRMS (ESI-TOF): *m/z* calculated for $C_{27}H_{40}N_2O_4$ $[M]^+$ = 456.2988, found = 456.2982.

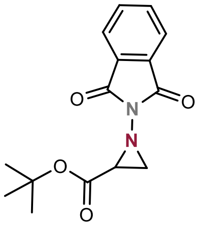

***tert*-butyl 1-(1,3-dioxoisoindolin-2-yl)aziridine-2-carboxylate (2az)**
Following General Procedure A, prepared from 0.25 mmol of **AT1** and 2.0 equiv. of *tert*-butyl acrylate (**1az**) in 24 h, the title compound was isolated via column chromatography (0–12% EtOAc/Hexane) as a white solid (32 mg, 43% yield, 1:3 *cis/trans* mixture). Analytical data for **2az** was in accordance with literature data.[8]
TLC (SiO_2) R_f = 0.20 in 9:1 Hexanes:EtOAc.
^{1}H NMR (500 MHz, $CDCl_3$) (δ, ppm): 7.78 (dd, J = 5.4, 3.1 Hz, 2H, *trans*), 7.77 – 7.74 (m, 2H, c*is*), 7.70 (dd, J = 5.5, 3.1 Hz, 2H, *trans*), 7.66 (dd, J = 5.4, 3.1 Hz, 2H, *cis*), 3.20 (dd, J = 5.6, 1.8 Hz, 1H, *cis*), 3.15 (d, J = 5.9 Hz, 1H, *cis*), 3.06 (dd, J = 7.8, 5.5 Hz, 1H, *trans*), 2.80 (dd, J = 5.4, 1.7 Hz, 1H, *cis*) 2.80 (dd, J = 5.4, 1.7 Hz, 1H, *trans*), 2.75 (dd, J = 7.8, 1.7 Hz, 1H, *trans*), 1.53 (s, 9H, *trans*), 1.38 (s, 9H, *cis*).
^{13}C NMR (126 MHz, $CDCl_3$) (δ, ppm): 167.5, 166.2, 165.1, 165.0, 134.7, 134.3, 130.7, 130.5, 123.7, 123.3, 83.1, 83.0, 41.3, 39.4, 36.8, 36.5, 28.4, 28.1.

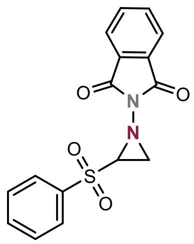

2-(2-(phenylsulfonyl)aziridin-1-yl)isoindoline-1,3-dione (2ba)

Following General Procedure A, prepared from 0.50 mmol **AT1** and 1.5 equiv. of phenyl vinyl sulfone (**1ba**) in 24 h, the title compound was isolated via flash column chromatography (0–10% EtOAc/Hexane) as a white solid (117 mg, 71% yield).

^{1}H NMR (400 MHz, $CDCl_3$) (δ, ppm): 8.16 – 8.00 (m, 2H), 7.82 – 7.56 (m, 7H), 4.21 – 4.05 (m, 1H), 3.34 (dd, *J* = 4.8, 2.3 Hz, 1H), 3.05 (dd, *J* = 7.3, 2.3 Hz, 1H).

^{13}C NMR (101 MHz, $CDCl_3$) (δ, ppm): 164.2, 137.4, 134.6, 134.6, 129.9, 129.6, 129.2, 123.6, 54.8, 36.2.

HRMS (ESI-TOF): m/z calculated for $C_{16}H_{12}N_2O_4S$ [M] = 328.0518, found 328.0509.

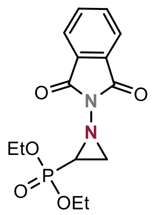

diethyl (1-(1,3-dioxoisoindolin-2-yl)aziridin-2-yl)phosphonate (2bb)

Following General Procedure B, prepared from 0.50 mmol of **AT1** and 2.0 equiv. of vinyl diethylphosphonate (**1bb**) in 24 h. The title compound was isolated via crystallizing the impurities at -70 °C and running a quick column through a small plug of silica with (1:09:90 Et_3N:EtOAc:Hexanes). The desired compound was collected at the baseline as brown liquid and recrystallized at -76 °C to access the desired product (65 mg, 40% yield).

^{1}H NMR (500 MHz, CD_2Cl_2) (δ, ppm): 2.88 – 2.71 (m, 3H). 7.78 (dd, *J* = 5.5, 3.1 Hz, 2H), 7.73 (dd, *J* = 5.5, 3.1 Hz, 2H), 4.33 – 4.13 (m, 4H), 2.73 – 2.66 (m, 3H), 1.37 (td, *J* = 7.1, 1.6 Hz, 6H).

^{13}C NMR (500 MHz, CD_2Cl_2) (δ, ppm): 165.1, 134.7, 130.5, 123.5, 63.5 (dd, *J* = 65.0, 6.1 Hz), 35.6 (d, *J* = 116.3 Hz), 34.9 (d, *J* = 85.6 Hz), 16.7 (d, *J* = 6.1 Hz).

HRMS (ESI-TOF): *m/z* calculated for $C_{14}H_{17}N_2O_5P$ $[M]^+$ = 324.0875, found 324.0878.

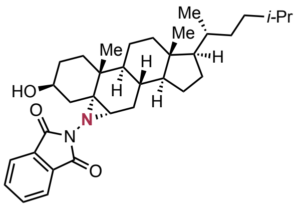

2-((3*S*,4a*R*,5a*S*,6a*S*,6b*S*,9*R*,9a*R*,11a*S*,11b*R*)-3-hydroxy-9a,11b-dimethyl-9-((*R*)-5-methylhexan-2-yl)hexadecahydro-5*H*-cyclopenta[1,2]phenanthro[8a,9-*b*]azirin-5-yl)isoindoline-1,3-dione (2bc)

Following General Procedure A, prepared from 0.25 mmol of **AT1** and 2.0 equiv. of cholesterol (**1bc**) in 24 h, the title compound was isolated via column chromatography (0-30% EtOAc/Hexane) as a white solid (51 mg, 37% yield).

TLC (SiO_2) Rf = 0.6 in 7:3 Hexanes:EtOAc.
^{1}H NMR (400 MHz, $CDCl_3$) (δ, ppm): 7.74 – 7.71 (m, 2H), 7.68 – 7.64 (m, 2H), 3.91 – 3.79 (m, 1H), 3.30 – 3.27 (m, 1H), 2.44 – 2.38 (m, 1H), 2.22 – 2.12 (m, 1H), 2.08 – 1.89 (m, 2H), 1.88 – 1.75 (m, 2H), 1.74 – 1.57 (m, 2H), 1.53 – 0.92 (m, 22H), 0.93 – 0.81 (m, 10H), 0.73 – 0.60 (m, 4H).
^{13}C NMR (101 MHz, $CDCl_3$) (δ, ppm): 166.9, 134.0, 122.8, 69.1, 57.0, 56.4, 54.4, 51.0, 49.9, 42.5, 40.1, 39.6, 37.1, 36.3, 36.0, 35.9, 35.8, 31.6, 30.9, 30.7, 28.4, 28.2, 24.4, 24.0, 23.0, 22.7, 22.4, 19.4, 18.8, 12.1.
HRMS (ESI-TOF): m/z calculated for $C_{20}H_{24}N_2O_4$ [M] = 546.3821, found 546.3798.

Characterization of Other Aziridine Products

Listed are other aziridination reactions that were low-yielding, and we were unable to isolate.

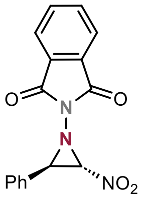

***Trans*-2-(2-nitro-3-phenylaziridin-1-yl)isoindoline-1,3-dione (2bd)**
Following General Procedure A, prepared from 0.50 mmol of **AT1** and 1.2 equiv. of *trans*-β-nitrostyrene (**1bd**) in 24 h. ^{1}H NMR yield was determined using CH_2Br_2 as an external standard (34% NMR yield). Isolation was proven difficult due to the sensitivity of the compound to column chromatography.
^{1}H NMR (crude) (500 MHz, $CDCl_3$) (δ, ppm): 3.48 (dd, J = 11.5, 4.7 Hz, 1H, aziridine proton).
HRMS (ESI-TOF): *m/z* calculated for $C_{16}H_{11}N_3O_4$ $[M]^+$ = 309.0750, found 309.0748.

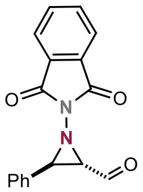

***Trans*-1-(1,3-dioxoisoindolin-2-yl)-3-phenylaziridine-2-carbaldehyde (2be)**
Following General Procedure A, prepared from 0.50 mmol of **AT1** and 1.2 equiv. of *trans*-cinnamaldehyde (**1be**) in 24 h. The title compound is reported as an 18% NMR yield. Isolation was proven difficult due to the sensitivity of the compound to column chromatography.
^{1}H NMR (crude) (500 MHz, $CDCl_3$) (δ, ppm): 3.48 (dd, J = 11.5, 4.7 Hz, 1H, aziridine proton).
HRMS (ESI-TOF): *m/z* calculated for $C_{17}H_{12}N_2O_3$ $[M]^+$ = 292.0848, found 292.0859.

Starting Material Characterization

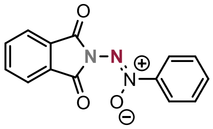

1-phenyl-2-phthalimidodiazene 1-oxide (AT1)
Following General Procedure E, using nitrosobenzene as the nitrosoarene, the title compound was isolated as an off-white solid (1.10 g, 42% yield). Analytical data in accordance with literature data.[1]
^{1}H NMR (500 MHz, $CDCl_3$) (δ, ppm): 8.30 – 8.24 (m, 1H), 7.93 (dd, *J* = 5.5, 3.1 Hz, 1H), 7.79 (dd, *J* = 5.4, 3.1 Hz, 1H), 7.69 – 7.61 (m, 1H), 7.55 (dd, *J* = 8.8, 7.3 Hz, 1H).
^{13}C NMR (126 MHz, $CDCl_3$) (δ, ppm): 162.4, 144.9, 134.7, 133.4, 131.2, 129.3, 124.2, 122.8.

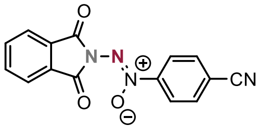

1-(4-cyanophenyl)-2-(1,3-dioxoisoindolin-2-yl)diazene 1-oxide (AT2)
Following General Procedure E, using 4-nitrosobenzonitrile as the nitrosoarene, the title compound was isolated as a fluffy white solid (0.88 g, 30% yield).
^{1}H NMR (500 MHz, $CDCl_3$) (δ, ppm): 8.43 (d, *J* = 8.8 Hz, 1H), 7.95 (dd, *J* = 5.5, 3.1 Hz, 1H), 7.88 (d, J = 8.8 Hz, 1H), 7.82 (dd, J = 5.5, 3.1 Hz, 1H).
^{13}C NMR (126 MHz, $CDCl_3$) (δ, ppm): 162.0, 147.2, 135.0, 133.4, 131.0, 124.4, 123.7, 117.3, 117.2.
HRMS (ESI-TOF): *m/z* calculated for $C_{15}H_8N_4O_3$ $[M]^+$ = 292.0596, found 292.0587.

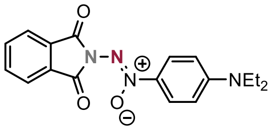

1-(4-(diethylamino)phenyl)-2-(1,3-dioxoisoindolin-2-yl)diazene 1-oxide (AT3)
Following General Procedure E, using *N,N*-Diethyl-4-nitrosoaniline as the nitrosoarene, the title compound was isolated as a fluffy white solid (0.88 g, 30% yield).
^{1}H NMR (500 MHz, $CDCl_3$) (δ, ppm): 8.15 (d, *J* = 9.4 Hz, 1H), 7.90 (dd, *J* = 5.4, 3.1 Hz, 1H), 7.75 (dd, *J* = 5.5, 3.1 Hz, 1H), 6.62 (d, *J* = 9.5 Hz, 1H), 3.45 (q, *J* = 7.1 Hz, 3H), 1.22 (t, *J* = 7.1 Hz, 4H).
^{13}C NMR (101 MHz, $CDCl_3$) (δ, ppm): 163.2, 151.4, 134.4, 131.3, 124.9, 123.9, 110.2, 45.0, 12.6.
HRMS (ESI-TOF): *m/z* calculated for $C_{18}H_{18}N_4O_3$ $[M]^+$ = 338.1379, found 338.1366.

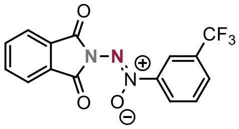

2-(1,3-dioxoisoindolin-2-yl)-1-(3-(trifluoromethyl)phenyl)diazene 1-oxide (AT4)
Following General Procedure E, using 3-nitroso-1-(trifluoromethyl)benzene as the nitrosoarene, the title compound was isolated as a fluffy white solid (1.7 g, 50% yield).
^{1}H NMR (500 MHz, $CDCl_3$) (δ, ppm): 8.57 (d, *J* = 2.0 Hz, 1H), 8.50 (dd, *J* = 8.2, 2.2 Hz, 1H), 7.97 – 7.93 (m, 2H), 7.92 (s, 1H), 7.81 (dd, *J* = 5.5, 3.1 Hz, 2H), 7.72 (t, *J* = 8.1 Hz, 1H).

^{13}**C NMR** (126 MHz, $CDCl_3$) (δ, ppm): 162.1, 145.0, 134.9, 132.2 (q, *J* = 33.8 Hz), 131.1, 130.2, 130.0 (q, *J* = 3.5 Hz), 126.0, 124.3, 123.2 (q, *J* = 272.8 Hz), 120.2 (q, *J* = 3.9 Hz).
HRMS (ESI-TOF): *m/z* calculated for $C_{15}H_8F_3N_3O_3$ $[M]^+$ = 335.0518, found 335.0505.

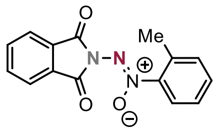

2-(1,3-dioxoisoindolin-2-yl)-1-(o-tolyl)diazene 1-oxide (AT5)
Following General Procedure E, using 2-nitrosotoluene as the nitrosoarene, the title compound was isolated as a white solid (1.3 g, 45% yield). Analytical data in accordance with literature data.[1]
^{1}**H NMR** (500 MHz, $CDCl_3$) (δ, ppm): 7.93 (dd, *J* = 5.5, 3.1 Hz, 1H), 7.79 (dd, *J* = 5.5, 3.1 Hz, 1H), 7.76 (dd, *J* = 7.9, 1.4 Hz, 1H), 7.47 (td, *J* = 7.5, 1.4 Hz, 1H), 7.35 (ddd, *J* = 11.4, 7.1, 2.0 Hz, 1H), 2.55 (s, 1H).
^{13}**C NMR** (126 MHz, $CDCl_3$) (δ, ppm): 162.3, 145.8, 134.7, 132.8, 132.1, 131.6, 131.2, 126.8, 124.5, 124.2, 18.5.

Deprotected and Derivatized Aziridine Characterization

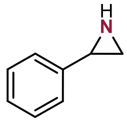

2-phenylaziridine (2b1)

Following General Procedure G, from 0.05 mmol of **2b**, using 8 mol % 1,2,3,5-Tetrakis(carbazol-9-yl)-4,6-dicyanobenzene (4CzIPN), the title compound is reported as an 42% NMR yield (2.0 equiv. of CH_2Br_2 was used as an external standard). ^{1}H NMR spectra is in good accordance with literature.[9] Product isolation is very challenging due to sensitivity of the compound on column.

^{1}H NMR (crude)(500 MHz, $CDCl_3$) (δ, ppm): 3.01 (dd, *J* = 6.1, 3.4 Hz, 1H), 2.20 (d, *J* = 6.1 Hz, 1H).

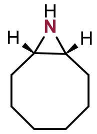

***Cis*-9-azabicyclo[6.1.0]nonane (2al1)**

Following General Procedure G, from 0.5 mmol of **2al**, using 0.05 mol % of [4,4′-Bis(1,1-dimethylethyl)-2,2′-bipyridine-N1,N1′]bis[2-(2-pyridinyl-N)phenyl-C]iridium(III) hexafluorophosphate ([Ir(dtbbpy)(ppy)$_2$]PF_6) in 12 h, the title compound is reported as a 73% NMR yield. ^{1}H NMR spectra is in good accordance with literature.[10] Isolation was proven difficult due to the sensitivity of the compound to column chromatography. Thus, the crude was derivatized without further purification (see below).

^{1}H NMR (crude)(500 MHz, $CDCl_3$) (δ, ppm): 2.11 – 2.03 (m, 2H).

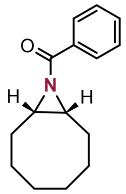

***Cis*-9-azabicyclo[6.1.0]nonan-9-yl)(phenyl)methanone (2al2)**

Reaction setup. To a stirred reaction vessel containing crude **2al1** was added CH_3Cl (1 mL), triethylamine (55.7 mg, 76.7 μL, 0.55 mmol) and Benzoyl chloride (141 mg, 116 μL, 1.00 mmol) dropwise at 0 °C. Then, 4-dimethylaminopyridine (6.11 mg, 0.1 Eq, 0.50 mmol) was added as a solid in one portion. The bath was immediately removed, and the reaction mixture was allowed to warm to ambient temperature under vigorous stirring for 3 h. The reaction mixture was then quenched by water and extracted with ether. The crude residue was dried with sodium sulfate and purified by column chromatography on basified silica (20% EtOAc with 5% Et_3N in hexanes) to afford the benzoyl-protected aziridine as a colorless oil (71 mg, 62% yield).

^{1}H NMR (500 MHz, $CDCl_3$) (δ, ppm): 8.02 – 7.94 (m, 2H), 7.58 – 7.51 (m, 1H), 7.44 (dd, *J* = 8.4, 7.0 Hz, 2H), 2.53 (td, *J* = 7.0, 6.6, 3.6 Hz, 2H), 2.31 (dq, *J* = 10.3, 3.0, 2.3 Hz, 2H), 1.67 (ddd, *J* = 10.1, 6.4, 3.6 Hz, 2H), 1.60 (dtd, *J* = 10.7, 8.7, 3.1 Hz, 2H), 1.52 – 1.41 (m, 6H).

^{13}C NMR (126 MHz, $CDCl_3$) (δ, ppm): 180.0, 133.9, 132.6, 129.2, 128.5, 41.7, 26.9, 26.59, 26.56.

HRMS (ESI-TOF): *m/z* calculated for $C_{15}H_{19}NO$ $[M]^+$ = 229.1467, found 229.1471.

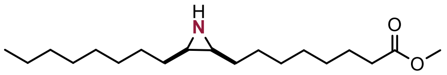

Cis-methyl 8-(3-octylaziridin-2-yl)octanoate (2ay1)
Following General Procedure G, from 0.25 mmol of **2ay**, using 1 mol % of [4,4′-Bis(1,1-dimethylethyl)-2,2′-bipyridine-N1,N1′]bis[2-(2-pyridinyl-N)phenyl-C]iridium(III) hexafluorophosphate ($[Ir(dtbbpy)(ppy)_2]PF_6$) in 8 h, the title compound was isolated by prepTLC (50% EtOAc /hexanes) to afford the unprotected-aziridine a light-brown solid (76 mg, 98% yield). Spectra is in good accordance with literature.[10]
TLC (SiO_2) R_f = ~0.35 in 1:1 Hexanes:EtOAc.
^{1}H NMR (600 MHz, $CDCl_3$) (δ, ppm): 3.66 (s, 3H), 2.30 (t, *J* = 7.6 Hz, 2H), 1.99 – 1.87 (m, 2H), 1.62 (p, *J* = 7.3 Hz, 2H), 1.51 – 1.21 (m, 25H), 0.94 – 0.85 (m, 3H).
^{13}C NMR (126 MHz, $CDCl_3$) (δ, ppm): 174.5, 51.6, 35.2, 35.1, 34.3, 32.0, 29.79, 29.77, 29.6, 29.43, 29.41, 29.3, 29.1, 29.0, 28.23, 28.17, 25.1, 22.8, 14.3.

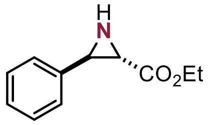

***Trans*-ethyl 3-phenylaziridine-2-carboxylate (2x1)**
Following General Procedure G, from 0.15 mmol of **2x**, using 8 mol % 1,2,3,5-Tetrakis(carbazol-9-yl)-4,6-dicyanobenzene (4CzIPN), the title compound is reported as a 41 % NMR yield (2.0 equiv. of CH_2Br_2 was used as an external standard). The ^{1}H NMR spectrum is in good accordance with literature.[11] Product isolation is very challenging due to the sensitivity of the compound on column.
^{1}H NMR (crude)(400 MHz, $CDCl_3$) (δ, ppm): 4.06 (q, *J* = 7.1 Hz, 2H), 2.96 (dd, *J* = 16.3, 8.5 Hz, 1H), 0.98 (t, *J* = 7.2 Hz, 3H).

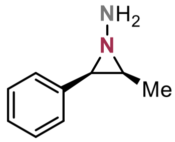

***Cis*-2-methyl-3-phenylaziridin-1-amine (*Z*-6c1).**
Reaction setup. In a 10 mL round-bottom flask equipped with a stir-bar, was charged 2-(2-methyl-3-phenylaziridin-1-yl)isoindoline-1,3-dione (*Z*-6c, 50 mg, 1.00 Eq, 0.18 mmol) and dissolved in Ethanol (0.05 M). Followed by the addition of hydrazine (58 mg, 56 μL, 10 Eq, 1.8 mmol) under N_2 atmosphere at room temperature. The reaction vessel was then capped with a septum and left to stir at room temperature for 12 h. Completion of the reaction was determined by TLC analysis. After the reaction was complete, the solvent was removed. The crude product was purified by crashing out the impurities with Et_2O and filtration to afford the 2-methyl-3-phenylaziridin-1-amine as colorless oil (20 mg, 79% yield).
^{1}H NMR (500 MHz, $CDCl_3$) (δ, ppm): 7.35 – 7.22 (m, 5H), 3.88 (s, 2H), 2.83 (d, *J* = 8.0 Hz, 1H), 2.12 (dq, *J* = 8.0, 5.9 Hz, 1H), 0.98 (d, *J* = 6.0 Hz, 3H).
^{13}C NMR (126 MHz, $CDCl_3$) (δ, ppm): 136.7, 128.4, 128.1, 127.1, 50.8, 46.4, 12.1.

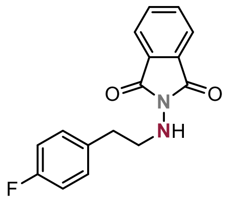

2-((4-fluorophenethyl)amino)isoindoline-1,3-dione (2a1)

Reaction setup. In a 5-dram vial equipped with a stir-bar, was charged 2-(2-(4-fluorophenyl)aziridin-1-yl)isoindoline-1,3-dione (2a, 30 mg, 1.00 Eq, 0.106 mmol) and dissolved in ethanol (0.1 M) and 10 mol % of Pd/C was added. The reaction vessel was then capped with a septum, and the atmosphere was displaced with a hydrogen balloon (1 atm). The reaction was allowed to stir vigorously for 3 h. Upon completion, the reaction was filtered through celite and concentrated down under reduced pressure to furnish the title compound as an oil (28 mg, 96% yield).

^{1}H NMR (400 MHz, CD_2Cl_2) (δ, ppm): 7.84 – 7.81 (m, 2H), 7.76 – 7.73 (m, 2H), 7.38 – 7.17 (m, 2H), 7.04 – 6.92 (m, 2H), 4.61 (t, *J* = 5.6 Hz, 1H), 3.29 (td, *J* = 7.3, 5.6 Hz, 2H), 2.86 (t, *J* = 7.5 Hz, 2H).

^{13}C NMR (126 MHz, CD_2Cl_2) (δ, ppm): 166.5, 134.2, 130.3, 130.2, 130.1, 123.1, 115.2, 115.0, 52.1, 33.7.

HRMS (ESI-TOF): *m/z* calculated for $C_{16}H_{13}FN_2O_2$ $[M]^+$ = 284.0961, found 284.0963.

Continuous-Photoflow Reaction Data

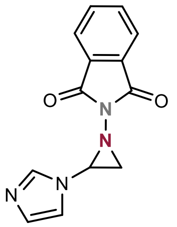

2-(2-(1*H*-imidazol-1-yl)aziridin-1-yl)isoindoline-1,3-dione (2ak)
Following General Procedure D, using 0.100 mmol of Z)-2-(1,3-dioxoisoindolin-2-yl)-1-phenyldiazene-1-oxide and 1.2 equiv. of 1-vinyl-1*H*-imidazole (**1ak**). The ^{1}H NMR yield was determined using CH_2Br_2, yield = 46%. Productivity = 0.023 mmol/hr, 4.2-fold increase from batch.

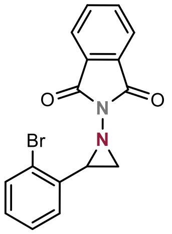

2-(2-(2-bromophenyl)aziridin-1-yl)isoindoline-1,3-dione (2m)
Following General Procedure D, using 0.100 mmol of Z)-2-(1,3-dioxoisoindolin-2-yl)-1-phenyldiazene-1-oxide and 1.2 equiv. of 1-bromo-2-vinylbenzene (**1l**). The ^{1}H NMR yield was determined using CH_2Br_2, yield = 60%. Productivity = 0.030 mmol/hr, 3.0-fold increase from batch.

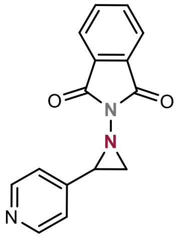

2-(2-(pyridin-4-yl)aziridin-1-yl)isoindoline-1,3-dione (2ai)
Following General Procedure D, using 0.100 mmol of Z)-2-(1,3-dioxoisoindolin-2-yl)-1-phenyldiazene-1-oxide and 1.2 equiv. of 4-vinylpyridine (**1ai**). The ^{1}H NMR yield was determined using CH_2Br_2, yield = 56%. Productivity = 0.028 mmol/hr, 3.1-fold increase from batch.

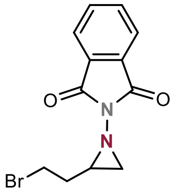

2-(2-(2-bromoethyl)aziridin-1-yl)isoindoline-1,3-dione (2ap)
Following General Procedure D, using 0.100 mmol of Z)-2-(1,3-dioxoisoindolin-2-yl)-1-phenyldiazene-1-oxide and 2.0 equiv. of 4-bromobut-1-ene (**1aq**). The ^{1}H NMR yield was determined using CH_2Br_2, yield = 34%. Productivity = 0.017 mmol/hr, 4.7-fold increase from batch.

Possible Reaction Pathways.

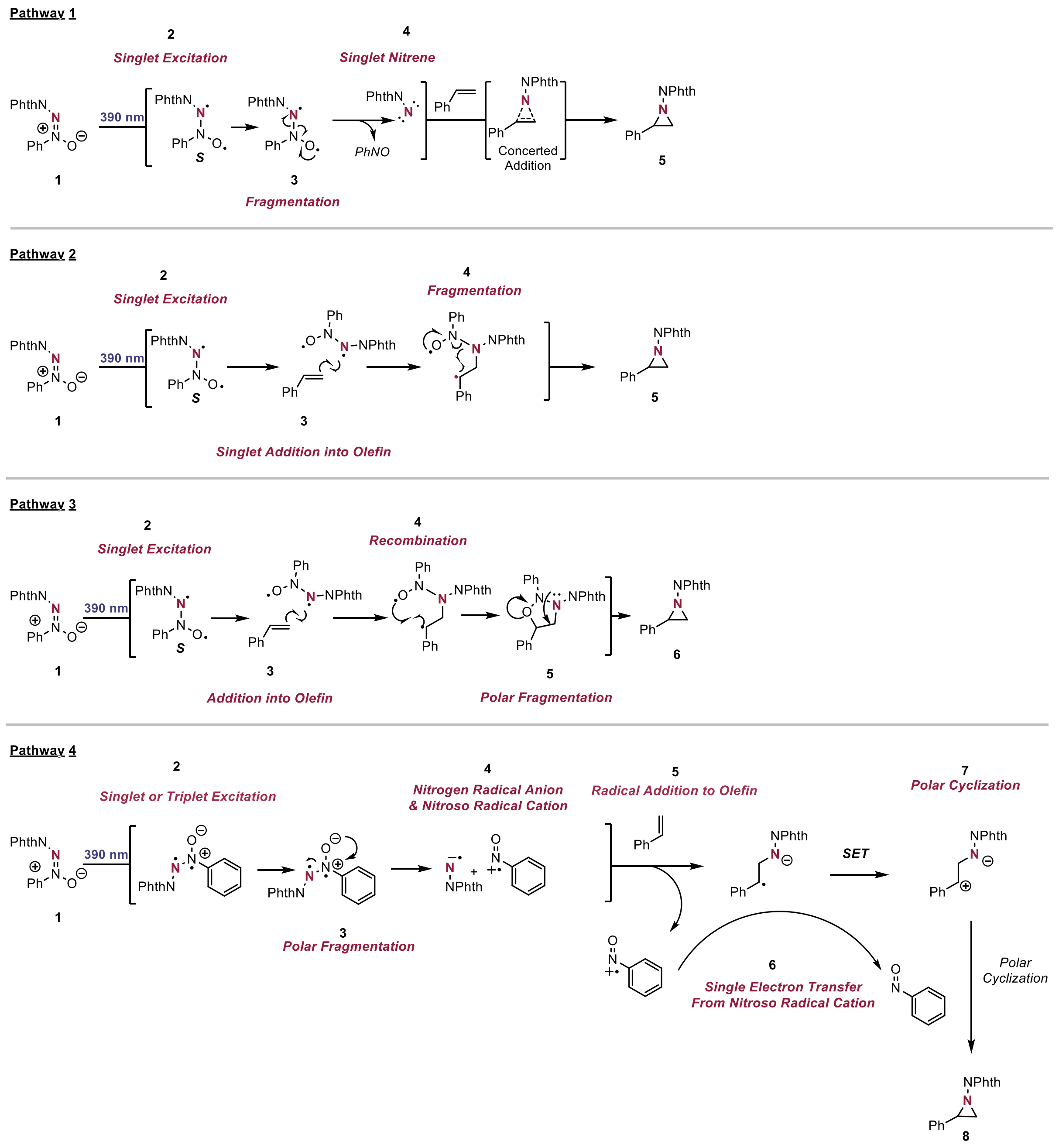

Scheme S1. Proposed possible reaction pathways considered for the aziridination of alkenes using azoxy-triazenes.

Reasoning: Based on the control experiments above (Table S4), and previous mechanistic studies following aromatic 1,3-dipoles like nitroarenes,[12,13,14] as well as mechanistic studies following other aziridination reactions, we considered the following 4 pathways: **Pathway 1**, nitrene transfer; **Pathway 2**, radical addition followed by radical fragmentation; **Pathway 3**, radical addition followed by polar fragmentation; and **Pathway 4**, via a radical anion intermediate.

Mechanistic studies

UV-Vis

To determine which components are photo-absorbing, UV-Vis absorption studies were performed. All absorption spectra were recorded on a Cary 100 UV-Visible spectrophotometer using the following parameter set: data interval 1.0 nm, bandwidth 1.0 nm, response time 0.1 s, scan speed 600 nm min-1, and baseline correction. Individual absorption spectra were recorded (Figure S3).

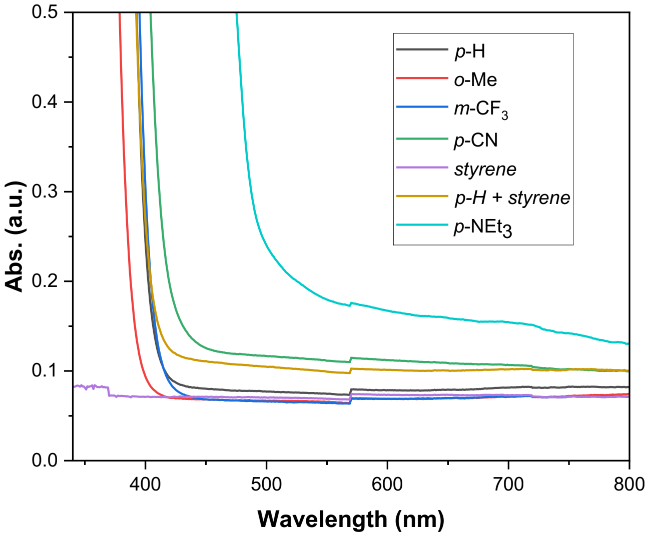

Figure S3. UV-Vis absorption spectra of the synthesized azoxy-triazenes, styrenes, and a solution of azoxy-triazene with styrene at reaction concentration.

Results. The data presented shows that the azoxy-triazenes are the sole absorbing species. No bathochromic shift was observed from a mixture of styrene and azoxy-triazene.

Light On/Off Studies

Reasoning. Light On/Off studies were conducted to determine whether or not the reaction still proceeded without the presence of light. If the reaction shows growth of aziridine or decay of the azoxy-triazene, it could indicate a radical cascade, where, based on the control studies, light was necessary only to initiate the reaction.

Reaction setup. **AT1** (6.68 mg, 0.025 mmol, 1.0 equiv.), 4-fluorostyrene (3.66 mg, 0.030 mmol, 1.2 equiv.), and MeCN-d_3 (0.50 mL, 0.050 M) were added under ambient conditions to an NMR tube. Under a PhotoNMR setup, a fiber-optic cable connected to a 395 nm lamp was added to the reaction, and ^{1}H NMR experiments were taken every 143 seconds. Product growth of **2a** (Figure S4a) was monitored by the integration of the peak from 2.64-2.76; **AT1** conversion (Figure S4b) was measured from the integration of the peak from 8.13-8.25 ppm.

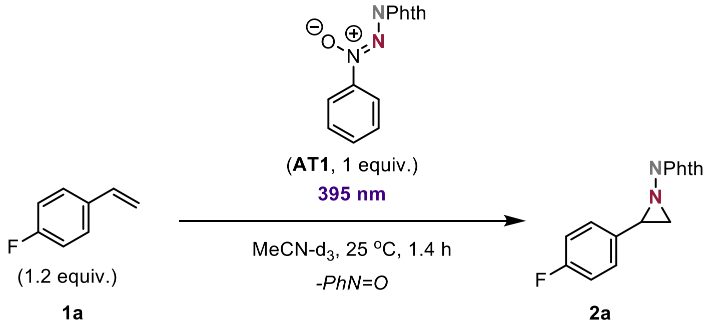

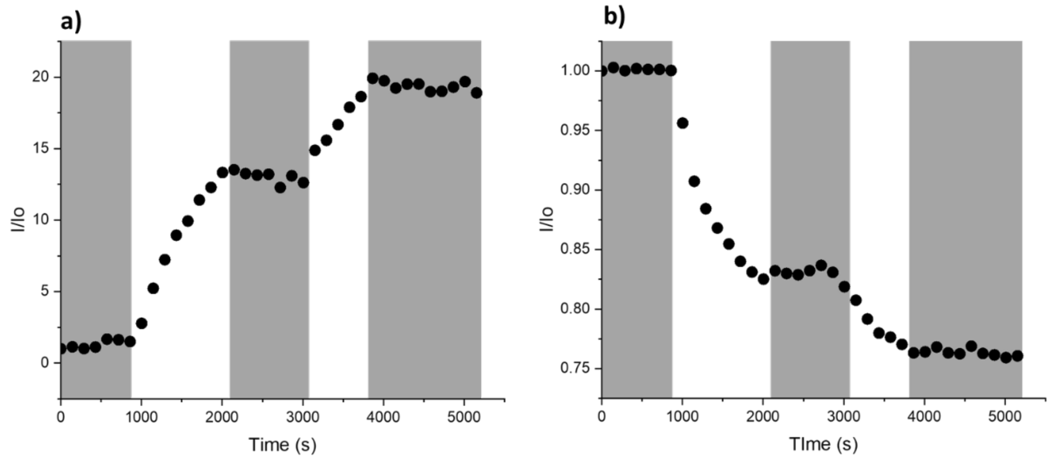

Figure S4. A PhotoNMR, light on/off study in relative integration vs time for (a) the production of **2a** and (b) consumption of **AT1** at 395 nm in MeCN-d_3.

Results. The lack of product formation (**2a**) or decomposition of azoxy-triazene (**AT1**) in the dark during these studies is evidence against a radical chain mechanism and that persistent light is necessary for the aziridination of the olefin.

Atmosphere Effects - Initial Rate Studies

Reasoning. To determine the effect the presence of oxygen had on the rate of the reaction, initial rate studies were conducted. As oxygen is a good quencher of species excited to the triplet-state, a lowered initial growth rate of the aziridination product under an oxygen-rich atmosphere is to be expected. If the rate has not lowered, it would indicate that the azoxy-triazene is likely promoted to its singlet-state.

Reaction setup. **AT1** (6.68 mg, 0.025 mmol, 1.0 equiv.), **1a** (3.66 mg, 0.030 mmol, 1.2 equiv.), and reaction-atmosphere-bubbled-MeCN-d_3 (0.50 mL, 0.050 M) were placed in an NMR tube. Under a PhotoNMR setup, a fiber-optic cable connected to a 395 nm lamp was added to the reaction, and ^{1}H NMR experiments were taken every 90 seconds. Product growth of **2a** (Figure S5) was monitored by the integration of the peak from 2.64-2.76 ppm.

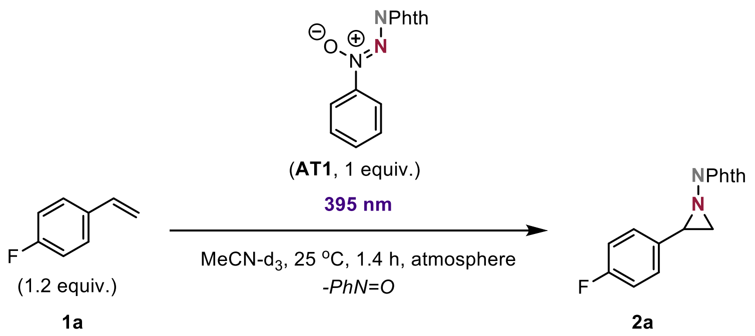

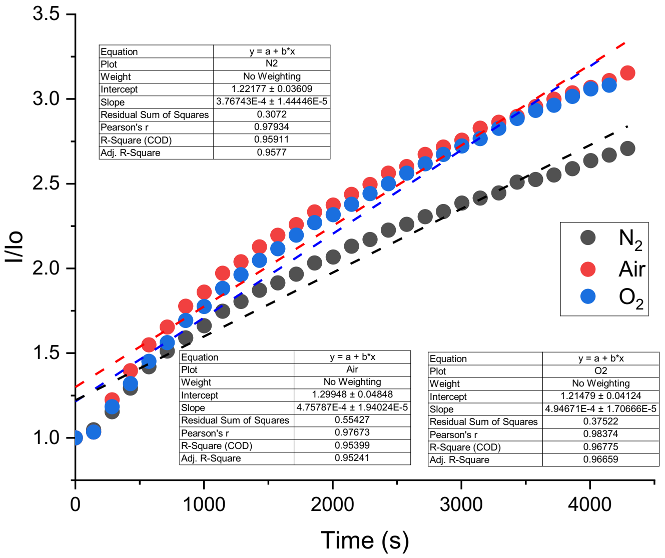

Figure S5. A ^{1}H PhotoNMR of the formation of **2a** under Nitrogen (black), Air (red), and Oxygen (blue) 395 nm.

Results. From Figure S5 and Table S4, the rate and yield of the reaction are not adversely impacted by the presence of oxygen. The presence of oxygen does exhibit a marginal increase in the rate. Therefore, it is likely the azoxy-triazene excites to its singlet-state.

Hammett Studies

Reasoning. To investigate whether the mechanism is going through a concerted or stepwise pathway, Hammett studies were conducted.

Competition experiments. The aziridination of *para*-substituted styrenes with **AT1** were carried out independently. In a vial equipped with a stir bar was charged *para*-substituted styrene (0.12 mmol, 1.0 equiv.), styrene (0.12 mmol, 1.0 equiv.), **AT1**(0.10 mmol) with MeCN-d_3 (0.050 M) under ambient conditions, which was then capped. The reaction mixture was then irradiated under 390 nm light for 15 minutes. ^{1}H NMR yield was taken as ratios. Reactions were taken in duplicates.

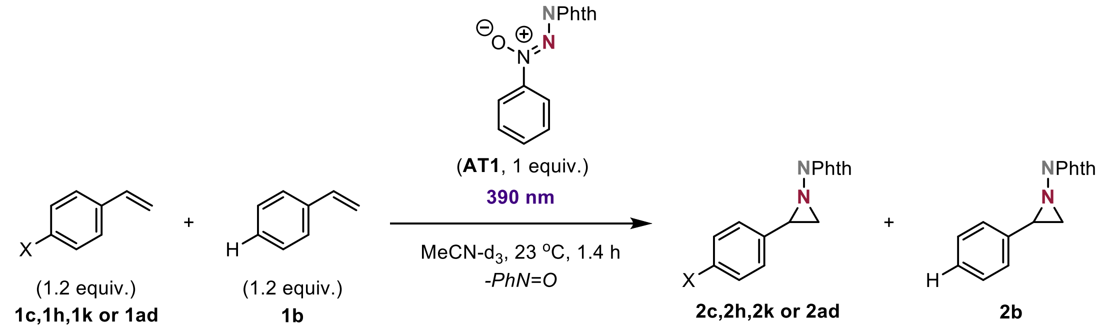

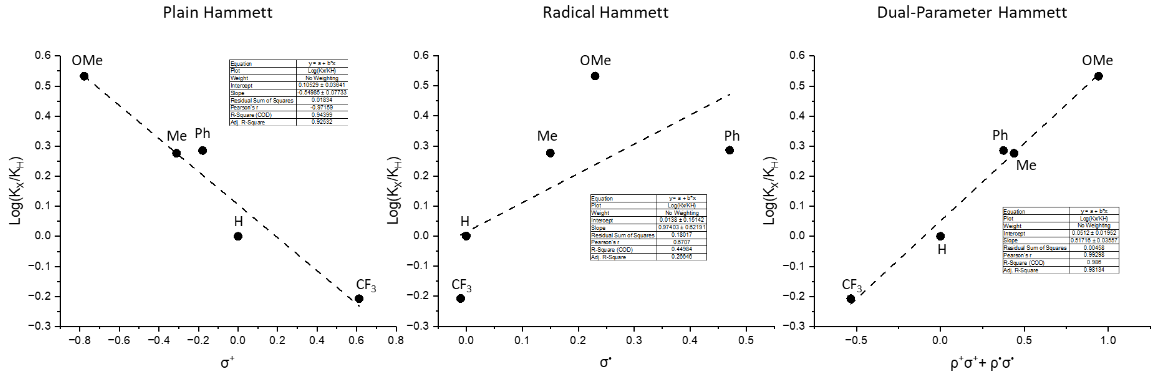

Figure S6. Competition Hammett plots showing averaged log(k_H/k_X) vs. A) plain Hammett parameter, B) Jiang & Ji radical Hammett parameter, and C) dual-Hammett parameter.

Results. Hammett studies conclude a linear trend for the plain Hammett (σ^+, ρ = -0.54, R^2 = 0.92). No linearity is observed using a radical parameter. As a plain Hammett showed good correlation, a good correlation (ρ = 0.52, R^2 = 0.98) with a dual-parameter is expected. The linear trend from the plain Hammett indicated a build-up of positive charge in the transition state, likely indicative a concerted mechanism rather than a stepwise (radical) mechanism.[15]

Pinacol Probe

Reasoning. Based on the Hammett studies (Figure S6), possible carbocation formation was tested for the aziridination reaction by subjecting a synthesized pinacol probe, 2-methyl-3-phenylbut-3-en-2-ol to General Procedure A.

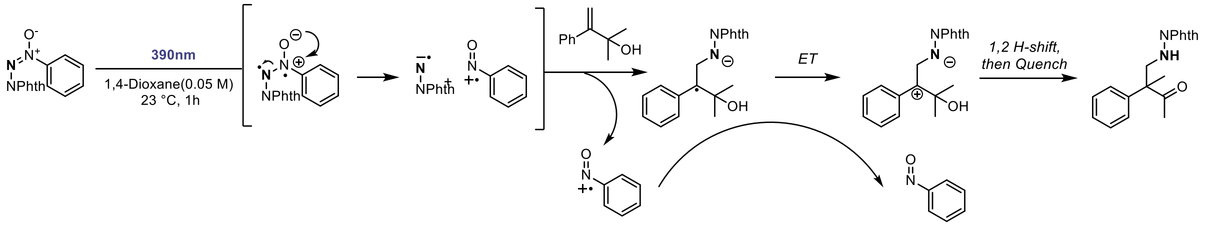

Scheme S2. Proposed pinacol probe reaction.

Results. 70% Aziridination of the pinacol probe was detected by ^{1}H NMR (shown below) using CH_2Br_2 as an external standard. A methyl shift of the pinacol probe from the conditions was not observed, indicating the reaction likely does not proceed through a carbocation intermediate. The lack of formation of a formal carbocation would indicate that **Pathway 4** (Scheme S1) is not likely.

^{1}H NMR (500 MHz, $CDCl_3$) of 2-methyl-3-phenylbut-3-en-2-ol being subjected to conditions.

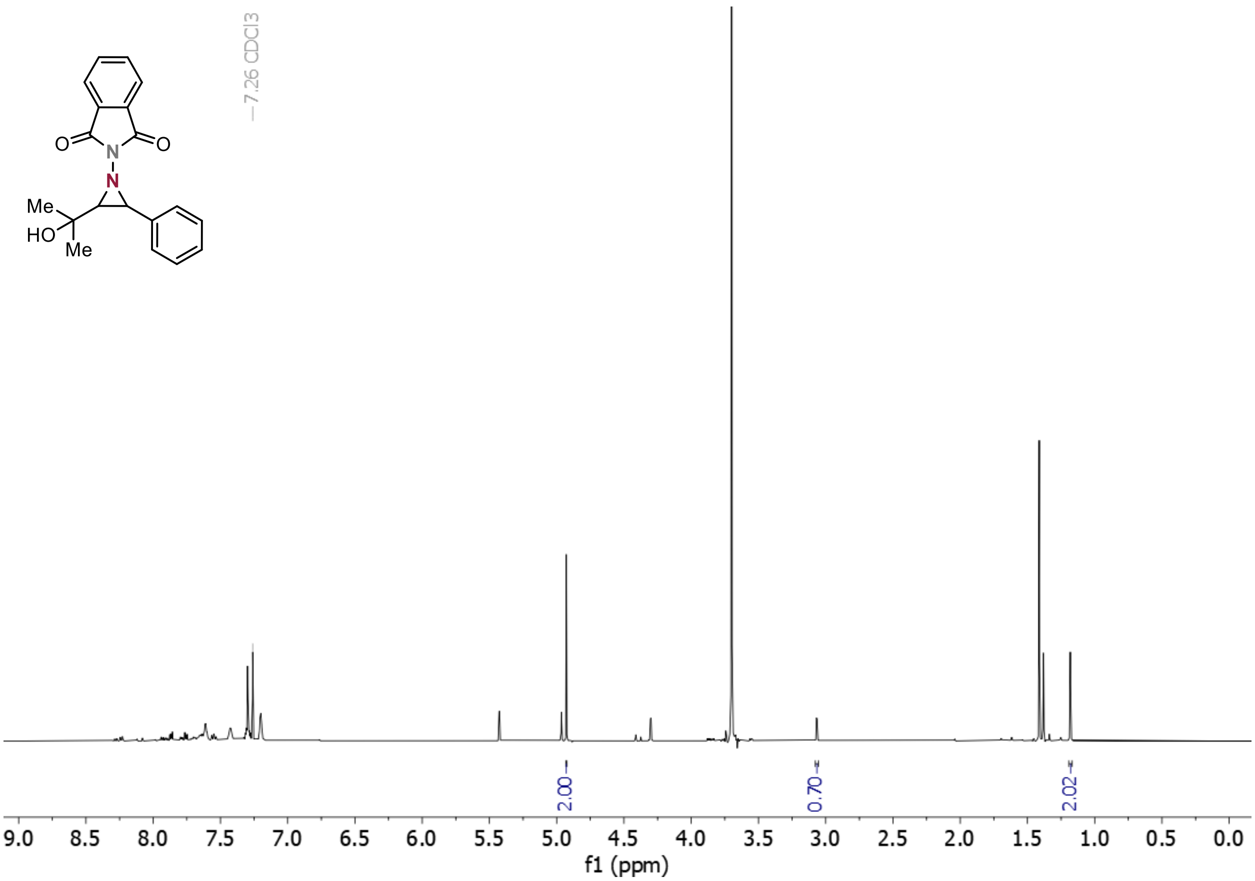

Nitrene Traps

Reasoning. Nitrene trap studies were conducted. The formation of nitrene intermediates is typically considered in the formation of aziridines. Furthermore, a concerted mechanism, as the competition Hammett (Figure S6) suggests, is indicative of a singlet-type nitrene. Thus, singlet nitrene traps dimethyl sulfide and dimethyl sulfoxide were used.[16,17]

Reaction setup. In a 1-dram vial, **AT1** (26.7 mg, 0.10 mmol, 1.0 equiv.), dimethyl sulfide (37.0 uL, 0.50 mmol, 5.0 equiv.) or dimethyl sulfoxide (35.5 uL, 0.50 mmol, 5.0 equiv.), and 1,4-dioxane (2.0 mL, 0.050 M) were left to stir at 1000 rpm and irradiated under 390 nm light for 24 h. ^{1}H NMR yields, using CH_2Br_2 as an external standard, were taken for any trapped products.

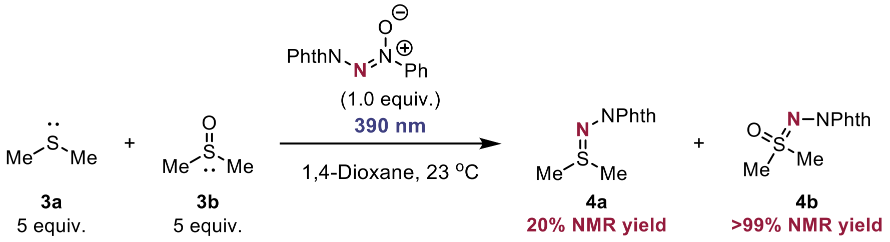

Scheme S3. Singlet nitrene traps, dimethyl sulfide (**3a**), and dimethyl sulfoxide (**3b**), subjected to the general reaction conditions.

Results. As dimethyl sulfide and dimethyl sulfoxide are known singlet nitrene traps, the trapped products **4a** (20% NMR yield), and **4b** (>99% NMR yield), strongly support the formation of a singlet nitrene species in the reaction.

^{1}H NMR (500 MHz, $CDCl_3$) of S,S-dimethyl-N-phthalimidosulfimide (4a)

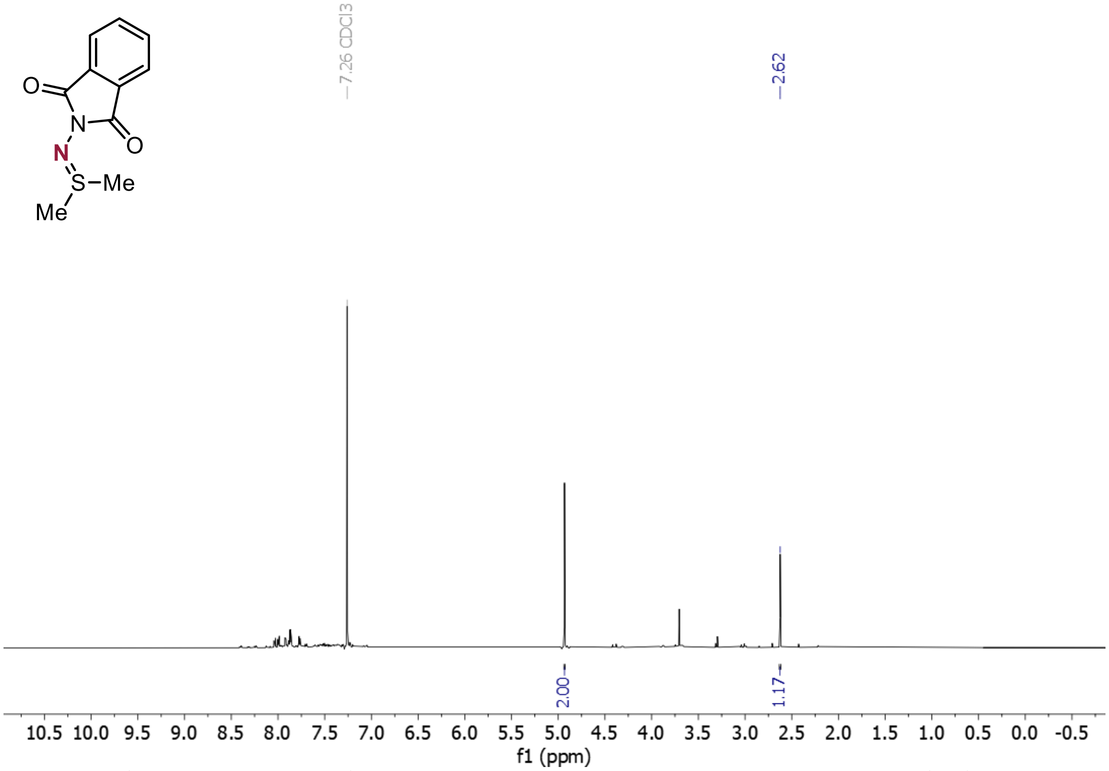

^{1}H NMR (400 MHz, $CDCl_3$) of S,S-dimethyl-N-phthalimido-sulfoximid (4b)

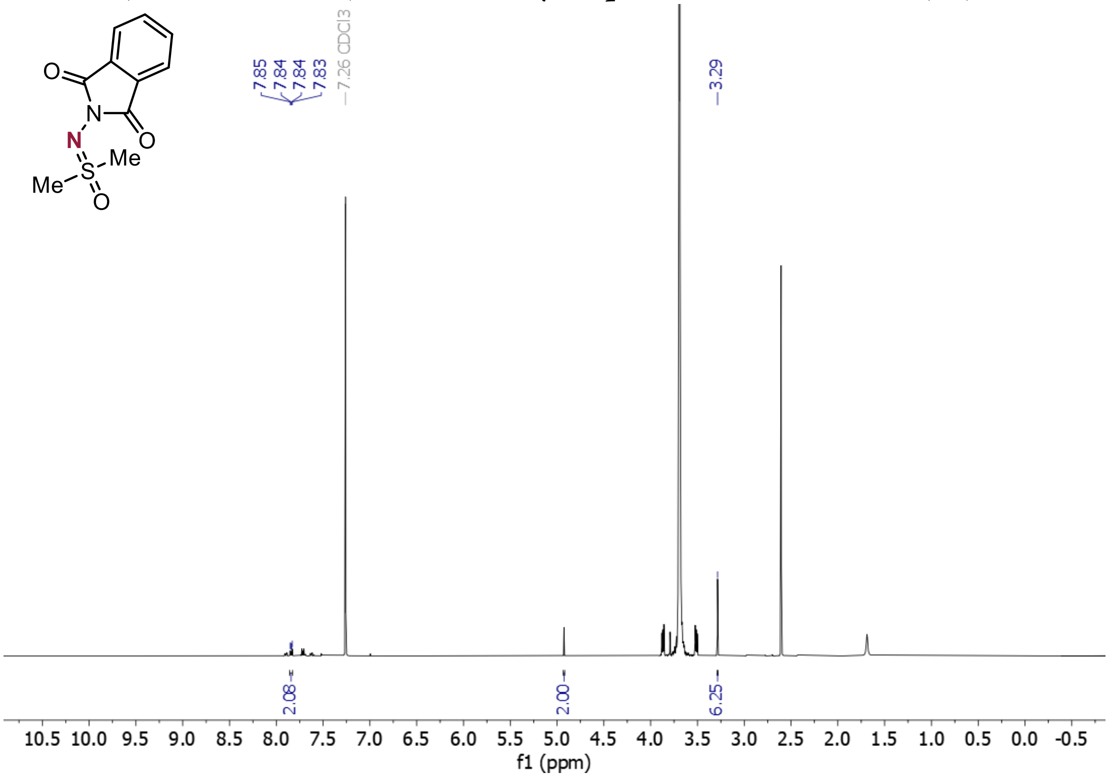

Stereochemical Probes

Reasoning. To further support the generation of a singlet nitrene intermediate, stereochemical probes were employed.[18,19] The preservation of stereochemistry frequently serves as a hallmark of a concerted pathway, contrasted to stereoablation which are suggestive of a stepwise pathway. Furthermore, it is established that a singlet nitrene typically undergoes a concerted mechanism, while a triplet nitrene is indicative of a stepwise mechanism.

Reaction setup. Geometrically defined alkenes, (*Z*)-1,4-dichlorobut-2-ene (**5a**) and (*E*)-1,4-dichlorobut-2-ene (**5b**) were subjected to General Procedure B and resulted in stereospecific aziridination (Figure S7). For aryl-substituted probes, (*Z*)-β-methylstyrene (**5c**) and (*E*)-β-methylstyrene (**5d**), which were subjected to General Procedure A, the former resulted in some stereoablation of the alkene geometry (3:1, cis to trans), while the latter was stereospecific (1:19, cis to trans) under the reaction conditions. Probes **5c** and **5d** were chosen particularly due to their standard use in the literature.

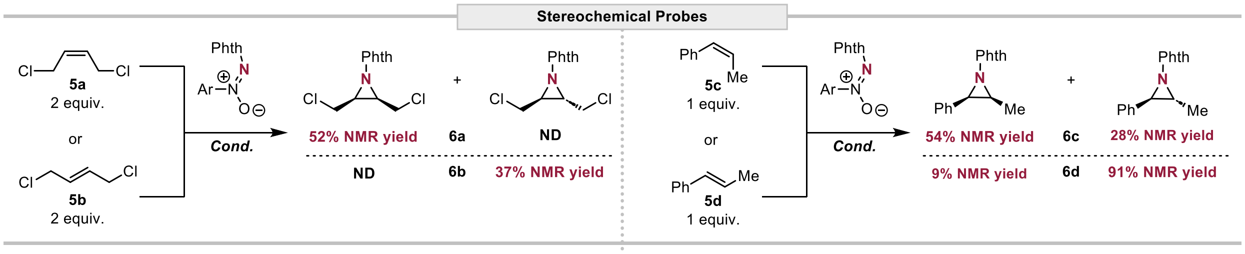

Figure S7. Stereochemical probes subjected to the general reaction conditions.

Table S5. Stereochemical probe **5c** subjected to the reaction conditions under different atmospheres.

Entry	*Solvent*	*Atmosphere*	***6c** (cis : trans)*[a]
1	**MeCN**	$\mathbf{N_2}$	**3:1**
2	MeCN	Air	2:1
3	MeCN	O_2	2:1
4	1,4-Dioxane	Air	1:1
5	1,4-Dioxane	N_2	2:1

Reactions were performed on a 0.1 mmol scale. [a] Based off ^{1}H NMR yields of **6c** using CH_2Br_2 as an external standard.

Results. Retention of initial geometry is observed for aliphatic probes (**5a-b**), and major retention is observed for the aromatic probes (**5c-d**). It has been observed that some aryl alkenes exhibit stereoablation of the alkene geometry by singlet nitrenes.[20,21,22] With this consideration, and the decrease in racemization under nitrogen, it is likely that the probes indicate a concerted mechanism, and the observed change in stereochemistry from employing **5c** is a result of other factors (see below).

^{1}H NMR (500 MHz, $CDCl_3$) of cis-2-(2,3-bis(chloromethyl)aziridin-1-yl)isoindole-1,3-dione (6a)

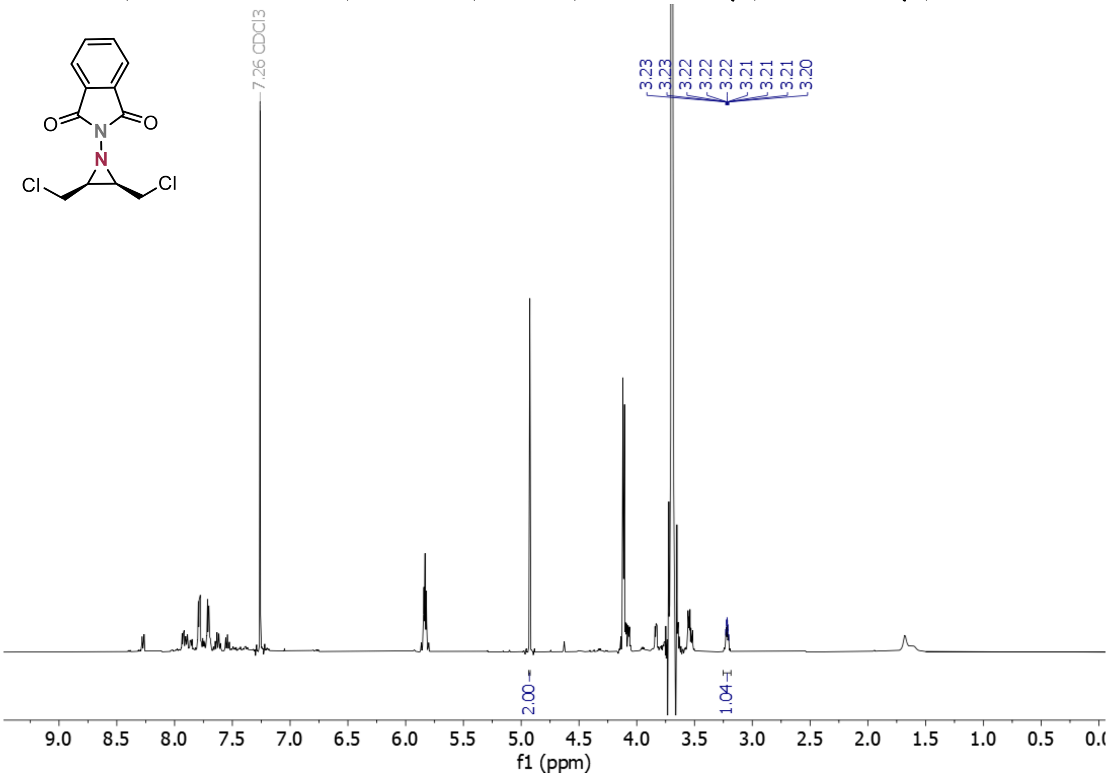

^{1}H NMR (500 MHz, $CDCl_3$) of trans-2-(2,3-bis(chloromethyl)aziridin-1-yl)isoindole-1,3-dione (6b)

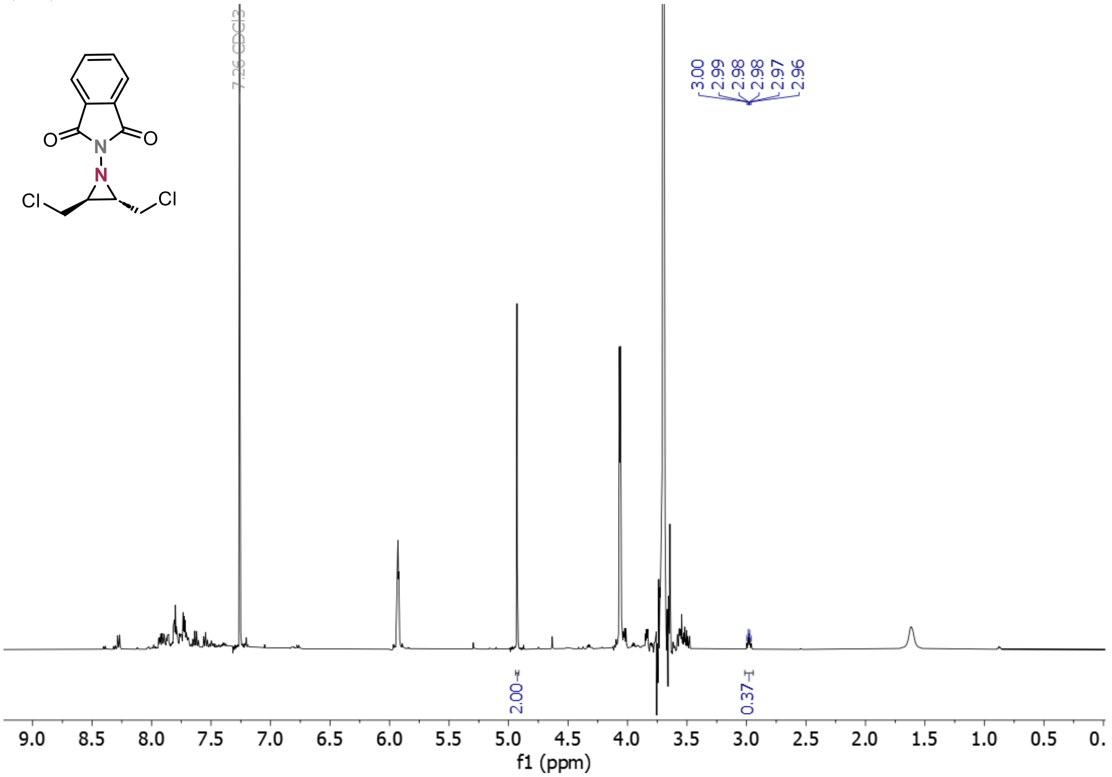

Stereoablation of Aziridine Product Geometry Under Visible Light.

Reasoning. Earlier reports have noted the reversibility of aziridines to reform their nitrene precursors under ultraviolet light.[23] Thus, it was investigated whether radical C–N disconnection could occur under light irradiation of the aziridine products, potentially leading to isomerization. If confirmed, this would imply that the observed stereochemistry from the use of (*Z*)-β-methylstyrene (**Z-5c**) under the reaction conditions is unlikely due to the presence of both a triplet and a singlet nitrene intermediate, but rather because the product itself isomerizes under these conditions. This phenomenon would provide an explanation for the absence of C–H insertion products typically associated with triplet nitrenes while still observing stereoablation of the stereochemical probes.

Reaction setup. In a 1-dram vial was added 0.1 mmol of synthesized aziridine **Z-5c**[24] and 2 mL of 1,4-dioxane. The reaction mixture was then irradiated using a Kessil lamp for 24 h and the isomeric ratio was determined.

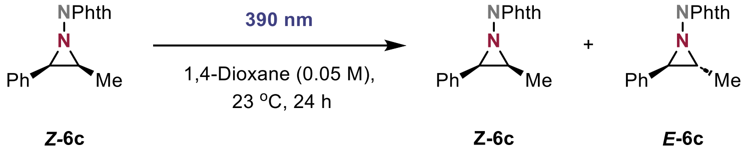

Table S6. Aziridine product ***Z*-6c** subjected to reaction conditions under varying wavelengths.

Entry	*Wavelength*	***Z*-6c**[a]	***E*-6c**[a]	**6c** (*cis* : *trans*)[b]
1	370 nm	42.3%	24.0%	1.76 : 1
2	390 nm	19.0%	16.0%	1.19 : 1
3	405 nm[a]	31.7%	25.3%	1.25 : 1
4	427 nm	22.3%	23.0%	0.97 : 1

[a] Denotes ^{1}H NMR yield using CH_2Br_2 as an external standard. [b] Using two 18 W Hepatochem lamps.

Results. It was observed that the aziridine product did exhibit stereoablation of the initial geometry under the reaction conditions as well as at other wavelengths, indicating that the observed outcome of using stereochemical probe **5c** (Figure S7) may not be a result of the presence of a triplet nitrene intermediate, but due to the reaction conditions itself causing the product to isomerize.

^{1}H NMR (500 MHz, $CDCl_3$) of 2-(2,3-bis(chloromethyl)aziridin-1-yl)isoindole-1,3-dione (6c)

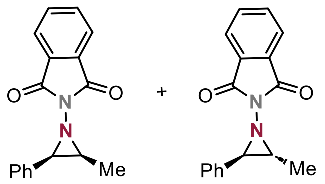

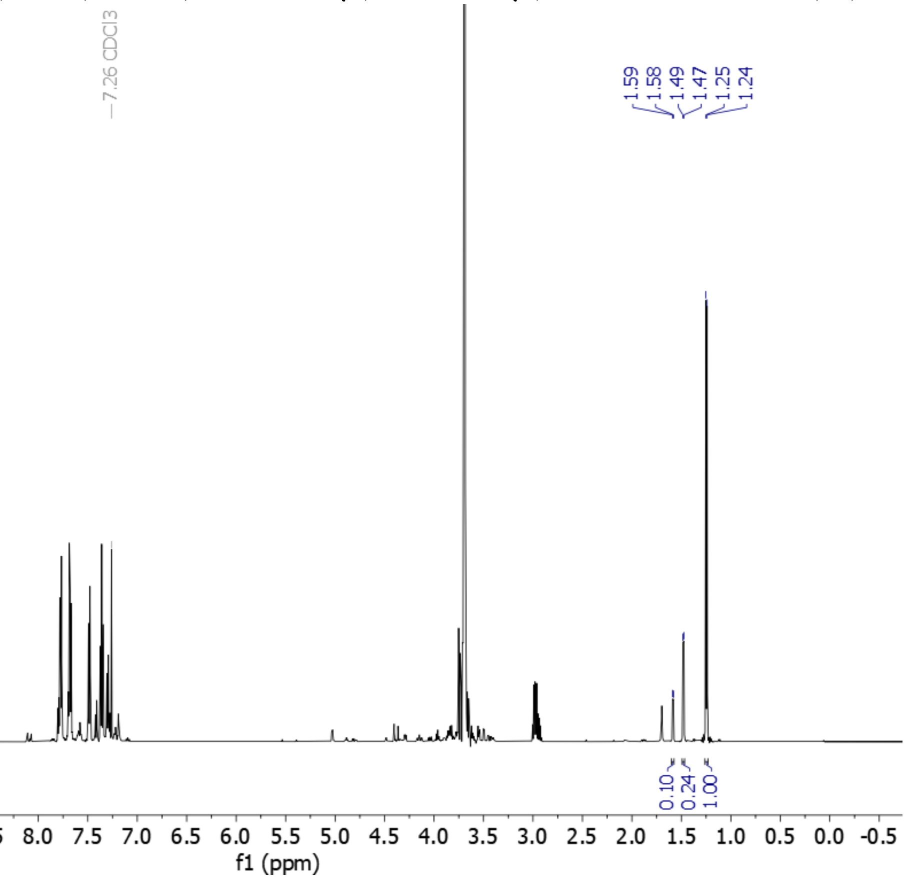

Concentration Dependence Study of Stereochemical Probes.

Reasoning. To further probe the possibility of a triplet nitrene intermediate, a concentration dependence study was conducted following prior literature.[25] The concept of this study follows that the nitrene generated does not need to remain in its ground state during a reaction nor does it need to be generated in its ground state. This means that within a particular reaction, there can exist a percentage of both the triplet and the singlet state of the free nitrene. This percentage is dependent on substrate concentration and the rates of interconversion between states. Thus, with changing alkene concentration, you would likely have a trend with the most populated intermediate reacting with the probe used. In the case of this method, the stereochemical probes would suggest a higher population of singlet nitrene than triplet. So, with an increasing concentration of olefin, we should see an increasing or decreasing amount of stereoablation.

Reaction setup. Geometrically defined alkene (*Z*)-1,4-dichlorobut-2-ene (**Z-5a**) was subjected to General Procedure B with varying equivalence of olefin (0.1-10 equiv.), simulating the study in prior literature (ref. 23).

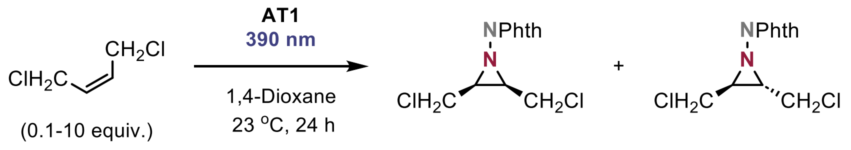

Z-5a Detected

***E*-6a** Not Detected

Results. For aliphatic probe **Z-5a**, no isomerization was detected when subjected to the reaction conditions at all concentrations of olefin. Hence, triplet nitrene formation is highly unlikely.

Kinetic Ratio Study

Reasoning. In 1991, Atkinson, Jones, and Kelly discovered that generation of free aminopthalimidonitrenes without formation of further intermediates resulted in a kinetic ratio of ~1.8 in the reactivity of methyl acrylate over styrene for competition studies.[26] Thus, a competition study resulting in a 1.8 ratio of methyl acrylate to styrene under the reaction conditions would indicate the likelihood of a free nitrene intermediate.

Reaction setup. Following General Conditions B. In a 1-dram vial was added 0.1 mmol of **AT1**, methyl acrylate (2.0 equiv.) and styrene (2.0 equiv.) and 1,4-Dioxane (0.05 M). The reaction vessel was irradiated under 390 nm light for 2 h. The reaction mixture was then concentrated down under a stream of nitrogen and then diluted in deuterated chloroform. Dibromomethane was added as an external standard and the kinetic ratios were recorded.

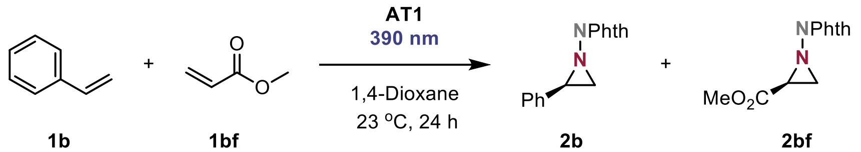

1b **1bf** **2b** **2bf**

Table S7. Kinetic Ratio of the competitive competition study from methyl acrylate end styrene.

Entry	*Equiv. of alkenes*	***2bf***[a]	***2b***[b]	*Kinetic Ratio*
1	**2.0**	**41.7%**	**23.0%**	**1.81**
2	10.0[c]	38.7%	21.0%	1.84
				**1.83**[d]

[a] Denotes ^{1}H NMR yield using CH_2Br_2 as an external standard as a total of methyl acrylate CH_3-peaks at 3.84 ppm 3.71 ppm (invertomers) and the average of aziridine peaks of styrene at 3.6 ppm and 2.79 ppm. [c] Alkene equivalence used by Atkinson in their Kinetic Ratio Study. [d] Average Kinetic Ratio across Entry 1 and Entry 2.

Results. The results exhibit a 1.81 ratio of the aziridination of methyl acrylate over the aziridination of styrene aziridine. This strongly supports the formation of a free nitrene.

^{1}H NMR (500 MHz, CDCl$_3$) from Kinetic Ratio Study

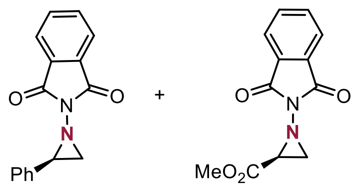

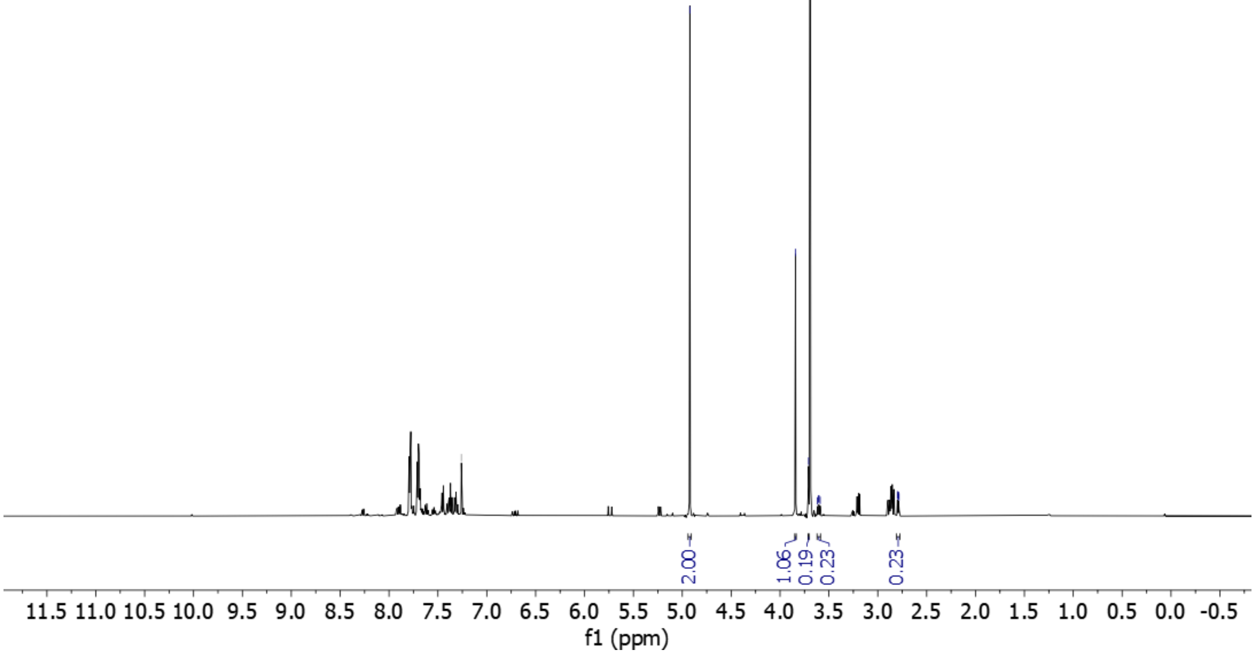

Intermediate Controls

Reasoning 1. We wondered if fragmentation of the azoxy-triazene necessitated the presence of an alkene. Excitation of **AT1** without styrene was completely converted to phthalimide (H-**NPhth**) as a major product in 1,4-dioxane under 390 nm irradiation. It was considered if this is a result of a tetrazene intermediate, which is reported to form from free nitrenes.[27]

Reaction setup. In a 1-dram vial equipped with a stir-bar, was charged **AT1** (0.10 mmol, 1.0 equiv.), and 1,4-dioxane (2.0 mL, 0.050 M). The reaction vessel was then capped and left to stir at 1000 rpm and irradiated under 390 nm Kessil lamps with a cooling fan for 24 h.

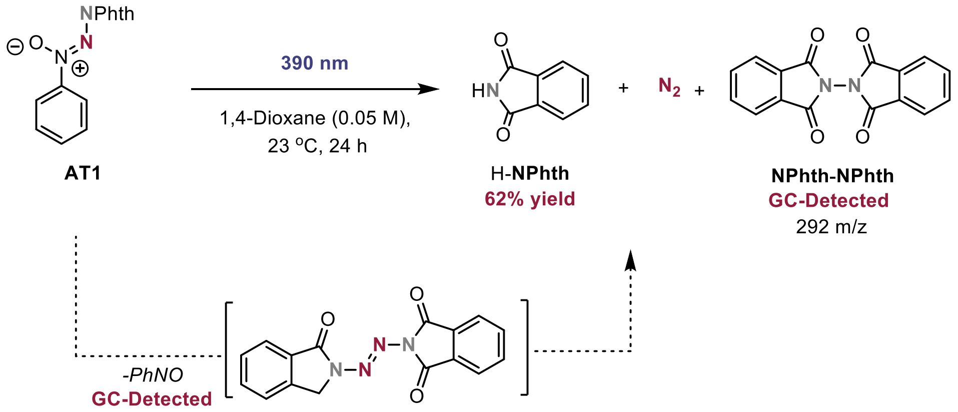

Scheme S4. Azoxy-triazene **AT1** subjected to the reaction conditions without the presence of an alkene.

Results. The reaction gave 62% isolated yield of H-**NPhth** as the major product, indicating the photodecomposition of **AT1** is possible without the presence of alkene. Phthalimide product formation likely occurs via a nitrene dimer (tetrazene) intermediate, which is known to form from amino nitrenes.[27]

Reasoning 2. To further confirm a tetrazene intermediate would generate H-**NPhth** under photochemical conditions, we subjected synthesized 1,4-bis-phthaloyltetrazene (90% purity) to the reaction conditions.

Reaction setup. In a 1-dram vial equipped with a stir-bar, was charged synthesized tetrazene[27] (80.0 mg, 0.25 mmol, 1.0 equiv.), and 1,4-dioxane (5.0 mL, 0.050 M). The reaction vessel was then capped and left to stir at 1000 rpm and irradiated under 390 nm Kessil lamps with a cooling fan for 24 h. The reaction yielded Phthalimide (H-**NPhth**) in 90% yield.

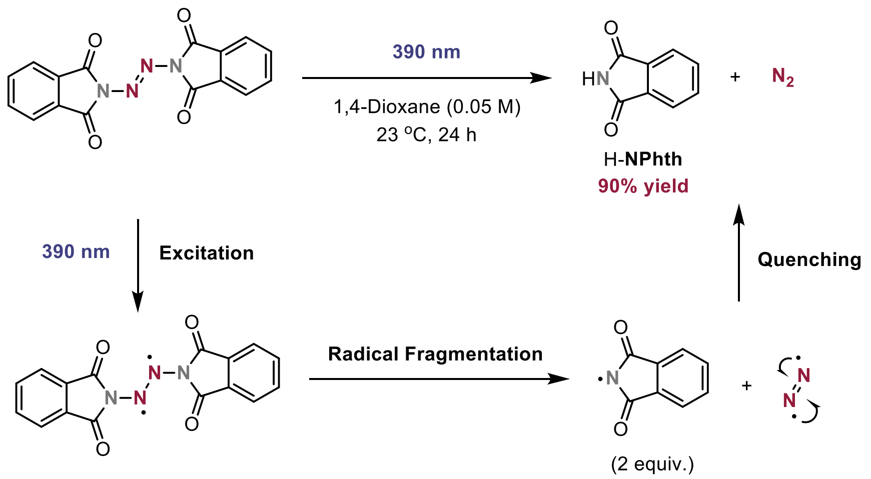

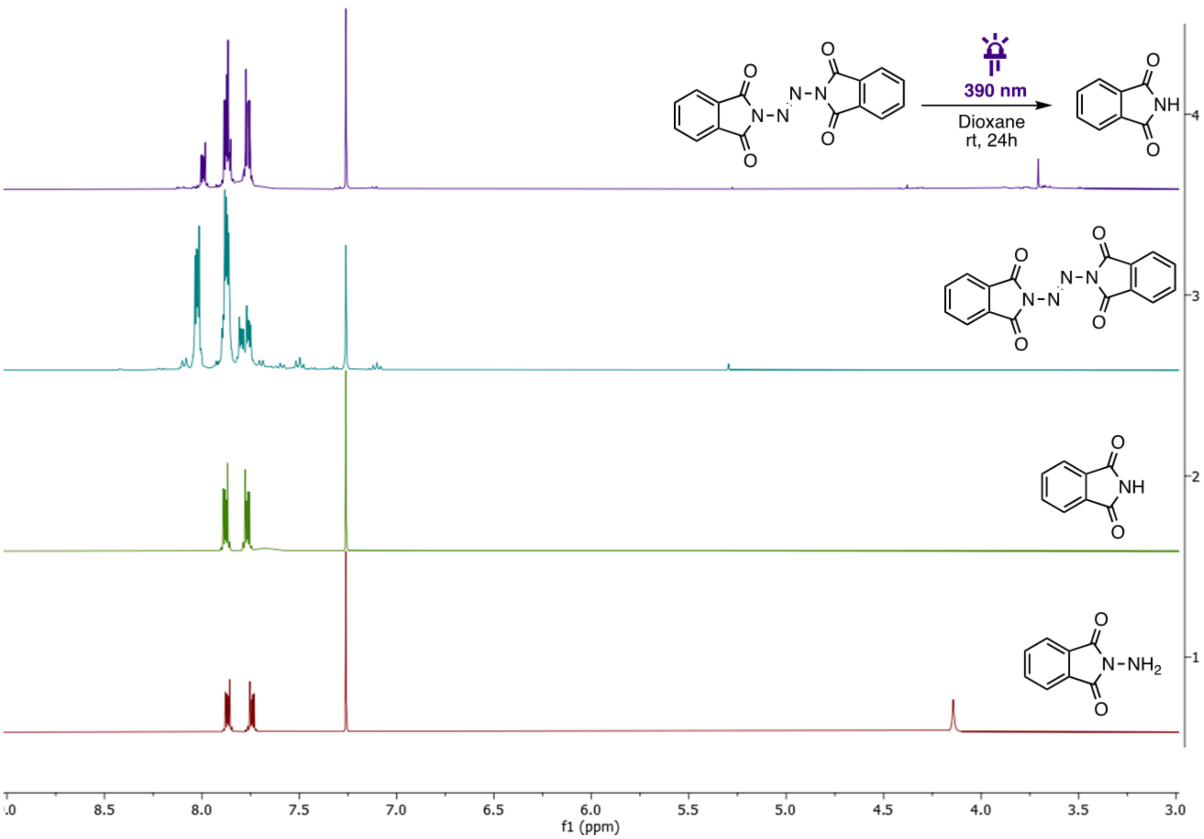

Figure S10. ^{1}H NMR spectra were obtained for the decomposition of the synthesized 1,4-bis-phthaloyltetrazene under reaction conditions (4), the partially isolated, synthesized 1,4-bis-phthaloyltetrazene (3), commercial phthalimide (2), and commercial *N*-aminophthalimide (1).

Results. Subjecting synthesized 1,4-bis-phthaloyltetrazene under the reaction conditions led to significant growth of the phthalimide.

Quenching Experiments

Reasoning. Quenching experiments were employed to help probe the reaction intermediates involved.

Reaction setup. In a 1-dram vial, **AT1** (26.7 mg, 0.10 mmol, 1.0 equiv.), **1a** (14.6 mg, 1.2 equiv., 0.12 mmol), Quencher (2.0 - 5.0 equiv.), and 1,4-dioxane (2.0 mL, 0.050 M) were irradiated under 390 nm light for 24 h. ^{1}H NMR yields, using CH_2Br_2 as an external standard, were taken of **2a**.

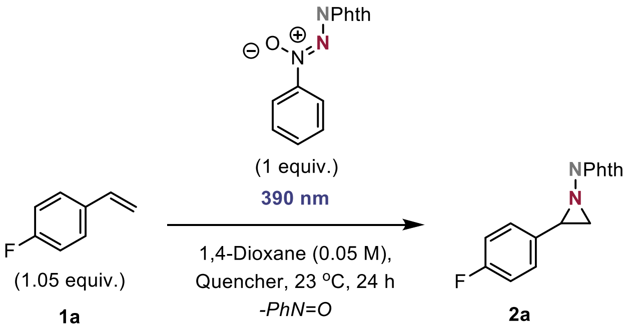

Table S8. Quenchers as additive under the reaction conditions.

Entry	*Quencher*	*Equiv.*	*Yield, % (**2a**)* [a]
1	None	2.0	94
2	N,N-Dimethylaniline	2.0	36
3	Imidazole	2.0	0
4	DABCO	2.0	0
5	Sodium Azide	2.0	76
6	2,4,6-TTBP	2.0	>99
7	Gavinoxyl	2.0	0
8	TEMPO	2.0 / 5.0	74 / 39
9	Ascorbate	2.0 / 5.0	76 / 87
10	Naphthalene	2.0 / 5.0	73 / 62

Results. Fluorescence quenchers, N,N-dimethylaniline and imidazole, exhibited substantial to complete quenching of the reaction. Notably, top singlet quenchers such as DABCO completely extinguished the reaction, while sodium azide displayed partial quenching, likely attributed to its limited solubility in 1,4-dioxane. This observation further substantiates that the azoxy-triazene predominantly adopts the singlet-state. Conversely, triplet quencher, 2,4,6-TTBP (2,4,6-tri-tertbutylphenol) demonstrated no quenching effect on the reaction. Radical traps, including gavinoxyl and TEMPO, were employed. Gavinoxyl exhibited complete quenching of the reaction; however, this effect might be attributed to the darkening of the solution. On the other hand, TEMPO displayed minimal quenching at 2.0 equivalence, and no trapped products were observed. Radical scavenger, sodium ascorbate led to minimal reduction in yield. This observation suggests that radical intermediates do not predominantly govern the reaction.

Radical Clock Study

Reasoning: Radical clocks have served as effective probes for detecting radical intermediates.[28] By subjecting an alkene radical clock to the reaction conditions, ring opening would suggest the presence of a radical intermediate, and thus **Pathway 2** and **Pathway 3** are likely.

Overall Reaction setup: We independently subjected synthesized (2-vinylcyclopropyl)benzene and (2-(2-methylprop-1-en-1-yl)cyclopropyl)benzene to the following reaction conditions:

For synthesized (2-vinylcyclopropyl)benzene: [29]

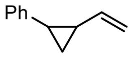

^{1}H NMR (400 MHz, $CDCl_3$) (δ, ppm): 7.27 (t, *J* = 7.5 Hz, 2H), 7.19 – 7.12 (m, 1H), 7.10 – 7.05 (m, 2H), 5.55 (ddd, *J* = 17.0, 10.3, 8.5 Hz, 1H), 5.11 (dd, *J* = 17.0, 1.6 Hz, 1H), 4.94 (dd, *J* = 10.3, 1.6 Hz, 1H), 1.93 (ddd, *J* = 9.3, 5.7, 4.4 Hz, 1H), 1.71 (tt, *J* = 8.8, 4.9 Hz, 1H), 1.21 (dt, *J* = 8.6, 5.4 Hz, 1H), 1.11 (dt, *J* = 8.9, 5.3 Hz, 1H).
^{13}C NMR (101 MHz, $CDCl_3$) (δ, ppm): 142.48, 140.80, 128.48, 125.83, 125.77, 112.68, 27.54, 25.38, 16.88.

Radical Clock Study using (2-vinylcyclopropyl)benzene

Reaction Setup: In a 4-dram vial equipped with a stir-bar, was charged 1- phenyl-2-phthalimidodiazene 1-oxide (**AT4**, 50 mg, 0.19 mmol, 1.0 equiv.), (2-vinylcyclopropyl)benzene (54 mg, 0.37 mmol, 2.0 equiv.), and 1,4-dioxane (7.51 mL, 0.025 M). The reaction vessel was then capped and left to stir at 1000 rpm and irradiated under 390 nm Kessil lamps with a cooling fan for 8 h. Completion of the reaction was determined by TLC analysis. After the reaction was complete, the solvent was removed. The crude product was purified by column chromatography (Hexanes: EtOAc: Et_3N= 9.3:0.5:0.2) to afford the aziridine products (2-(2-(2-phenylcyclopropyl)aziridin-1-yl)isoindoline-1,3-dione; as diastereomers, 1:0.4) in 16% (8.9 mg) as colorless oil.

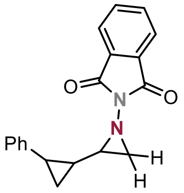

^{1}H NMR (500 MHz, $CDCl_3$) (δ, ppm): 7.82 – 7.72 (m, 3H), 7.72 – 7.64 (m, 3H), 7.50 – 7.28 (m, 2H), 7.26 (dd, *J* = 7.8, 6.5 Hz, 2H), 7.19 – 7.07 (m, 3H), 2.67 (tdd, *J* = 7.6, 5.3, 1.7 Hz, 1H), 2.50 (ddd, *J* = 7.9, 6.0, 2.5 Hz, 1H), 2.37 (ddd, *J* = 10.2, 5.8, 2.4 Hz, 1H), 2.14 (dt, *J* = 9.6, 5.1 Hz, 1H), 1.45 – 1.39 (m, 1H), 1.38 – 1.33 (m, 1H), 1.30 – 1.20 (m, 5H), 1.06 (dt, *J* = 8.7, 5.3 Hz, 1H), 0.98 (dt, *J* = 8.6, 5.2 Hz, 1H).
^{13}C NMR (126 MHz, $CDCl_3$) (δ, ppm): 165.5, 143.0, 142.9, 134.4, 130.8, 128.7, 128.7, 126.3, 126.2, 126.0, 123.2, 44.5, 44.3, 37.9, 37.4, 23.3, 23.0, 21.4, 20.5, 13.5, 12.7.
HRMS (ESI-TOF): *m/z* calculated for $C_{19}H_{16}N_2O_2$ $[M]^+$ = 298.0805 , found 298.0800.

For synthesized (2-(2-methylprop-1-en-1-yl)cyclopropyl)benzene: [29,30]

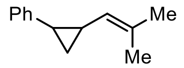

^{1}H NMR (500 MHz, $CDCl_3$) (δ, ppm): 7.27 (t, *J* = 7.5 Hz, 2H), 7.19 – 7.12 (m, 1H), 7.10 – 7.05 (m, 2H), 5.55 (ddd, *J* = 17.0, 10.3, 8.5 Hz, 1H), 5.11 (dd, *J* = 17.0, 1.6 Hz, 1H), 4.94 (dd, *J* = 10.3, 1.6 Hz, 1H), 1.93 (ddd, *J* = 9.3, 5.7, 4.4 Hz, 1H), 1.71 (tt, *J* = 8.8, 4.9 Hz, 1H), 1.21 (dt, *J* = 8.6, 5.4 Hz, 1H), 1.11 (dt, *J* = 8.9, 5.3 Hz, 1H).
^{13}C NMR (126 MHz, $CDCl_3$) (δ, ppm): 142.5, 140.8, 128.5, 125.83, 125.77, 112.7, 27.5, 25.4, 16.9.

Radical Clock Study using (2-(2-methylprop-1-en-1-yl)cyclopropyl)benzene

Reaction setup: In a 4-dram vial equipped with a stir-bar, was charged 1- phenyl-2-phthalimidodiazene 1-oxide (**AT1**, 50 mg, 0.19 mmol, 1.0 equiv.), (2-(2-methylprop-1-en-1-yl)cyclopropyl)benzene (64 mg, 0.37 mmol, 2.0 equiv.), and 1,4-dioxane (7.51 mL, 0.025 M). The reaction vessel was then capped and left to stir at 1000 rpm and irradiated under 390 nm Kessil lamps with a cooling fan for 8 h. After the reaction 8 h, the solvent was removed. The crude product was purified by column chromatography (Hexanes: EtOAc: Et_3N= 9.3:0.5:0.2) to afford the aziridine products (as diastereomers, 1:0.8) in 51% (32 mg) as yellow oil.

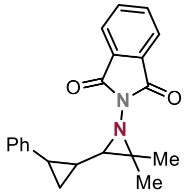

^{1}H NMR (500 MHz, $CDCl_3$) (δ, ppm): δ 7.75 (ddd, *J* = 5.4, 3.1, 1.9 Hz, 4H), 7.70 (dt, *J* = 5.3, 2.6 Hz, 4H), 7.26 (dt, *J* = 13.0, 7.4 Hz, 4H), 7.17 – 7.10 (m, 5H), 2.70 (d, *J* = 5.5 Hz, 1H), 2.65 – 2.62 (m, 1H), 2.43 (dt, *J* = 8.8, 5.0 Hz, 1H), 2.26 (dt, *J* = 8.8, 5.1 Hz, 1H), 1.50 (dt, *J* = 8.9, 5.1 Hz, 1H), 1.45 (s, 3H), 1.43 (s, 3H), 1.36 – 1.32 (m, 1H), 1.28 (s, 6H), 1.21 – 1.17 (m, 1H), 1.15 – 1.12 (m, 2H), 1.07 (ddd, *J* = 8.7, 5.7, 4.7 Hz, 1H).
^{13}C NMR (126 MHz, $CDCl_3$) (δ, ppm): 166.52, 143.33, 143.18, 134.29, 134.27, 131.20, 128.67, 128.64, 126.28, 126.18, 125.90, 125.88, 123.02, 54.89, 54.57, 49.07, 48.88, 22.05, 21.74, 21.13, 21.06, 20.75, 20.04, 20.01, 14.70, 14.18.

HRMS (ESI-TOF): *m/z* calculated for $C_{21}H_{20}N_2O_2$ $[M]^+$ = 332.1525, found 332.1536.

Radical Clock Scheme:

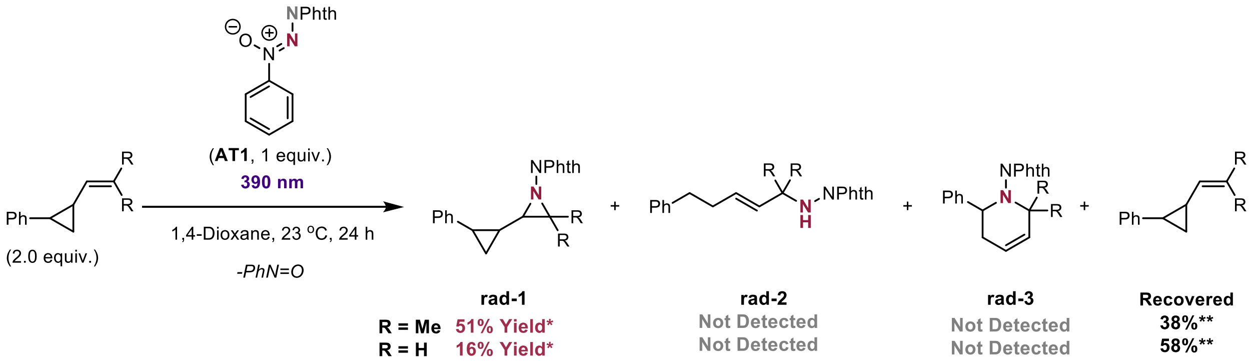

(Rad-1 is unstable, and easily decomposes. Partial product loss observed during isolation),
R = Me, 61% NMR Yield; R = H, 28% NMR Yield.
**Normalized off of 2.0 equiv of alkene.

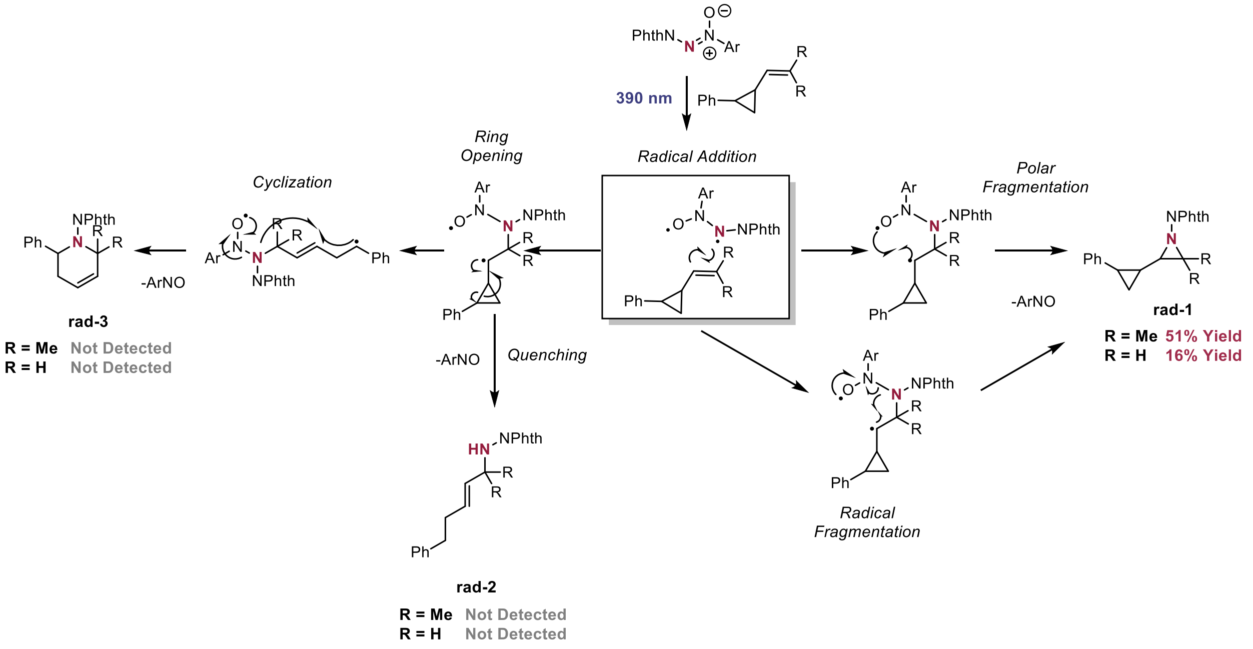

Results: Opening of the radical clock ring was not observed (**rad-2**; **rad-3**), indicating radical intermediates were likely not involved in the reaction. Using CH_2Br_2 as an external standard revealed 61% and 28% ^1H NMR yield of aziridine products when using (2-(2-methylprop-1-en-1-yl)cyclopropyl)benzene and (2-vinylcyclopropyl)benzene, respectively. This resulted in a respective isolated yield of 51% and 16% with a 38% and 58% recovery of the alkene starting material, respectively. It was observed these compounds are highly sensitive to column conditions and thus loss of product is observed during isolation. No ring-opened products were detected.

^{1}H NMR (400 MHz, $CDCl_3$) of (2-vinylcyclopropyl)benzene

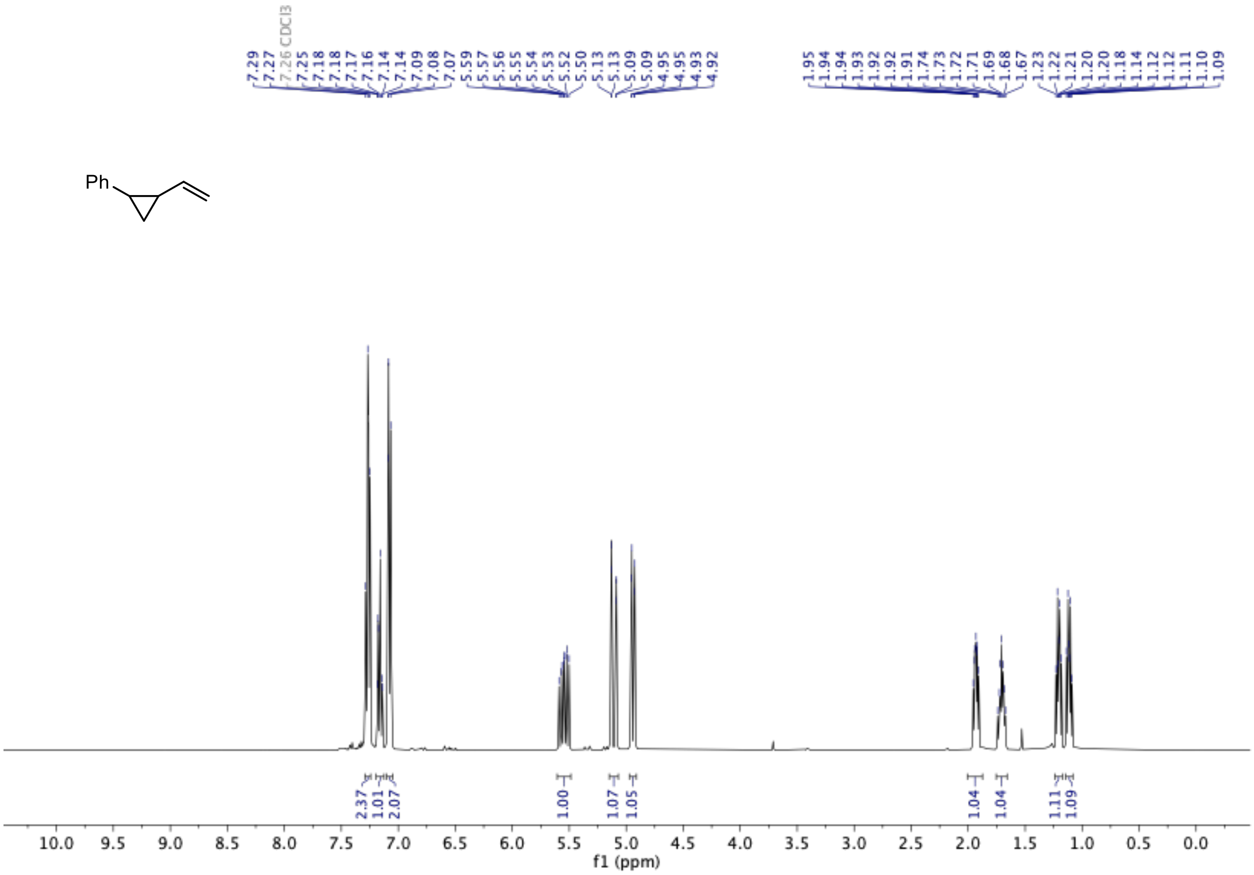

^{13}C NMR (101 MHz, $CDCl_3$) of (2-vinylcyclopropyl)benzene

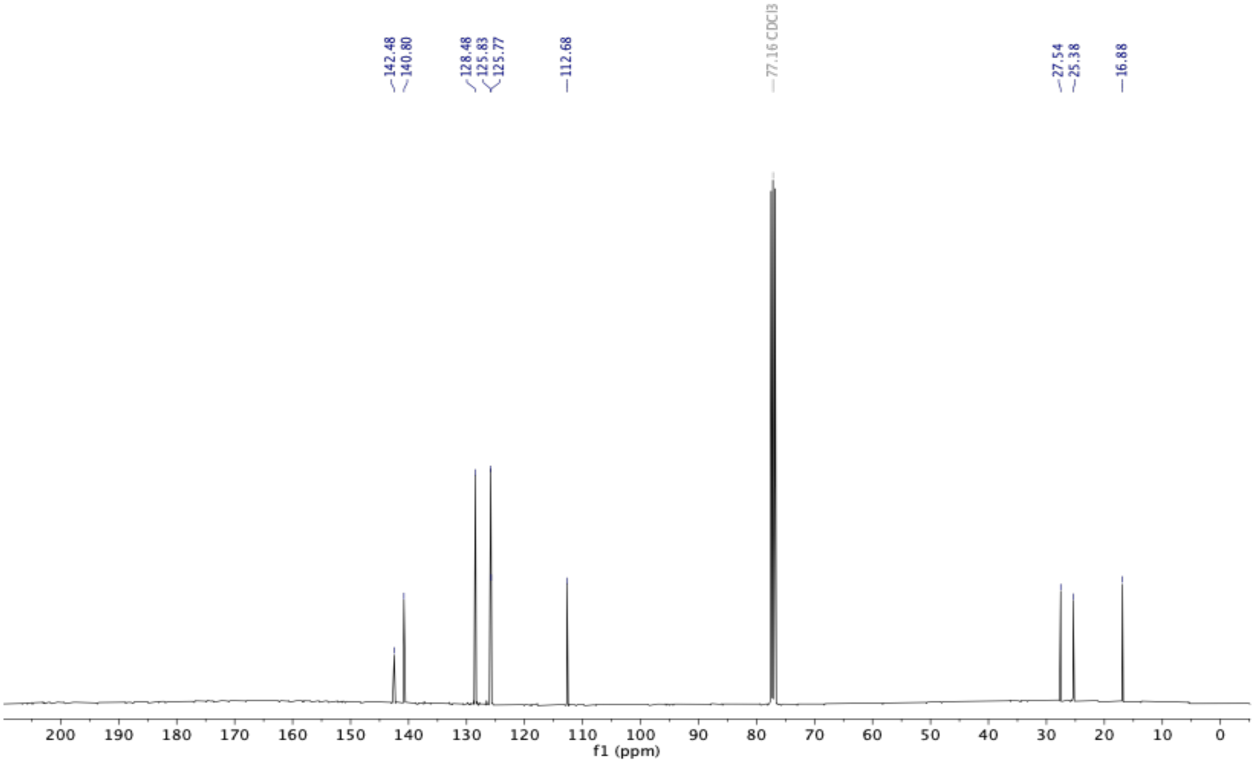

^{1}H NMR (500 MHz, $CDCl_3$) of (2-(2-methylprop-1-en-1-yl)cyclopropyl)benzene

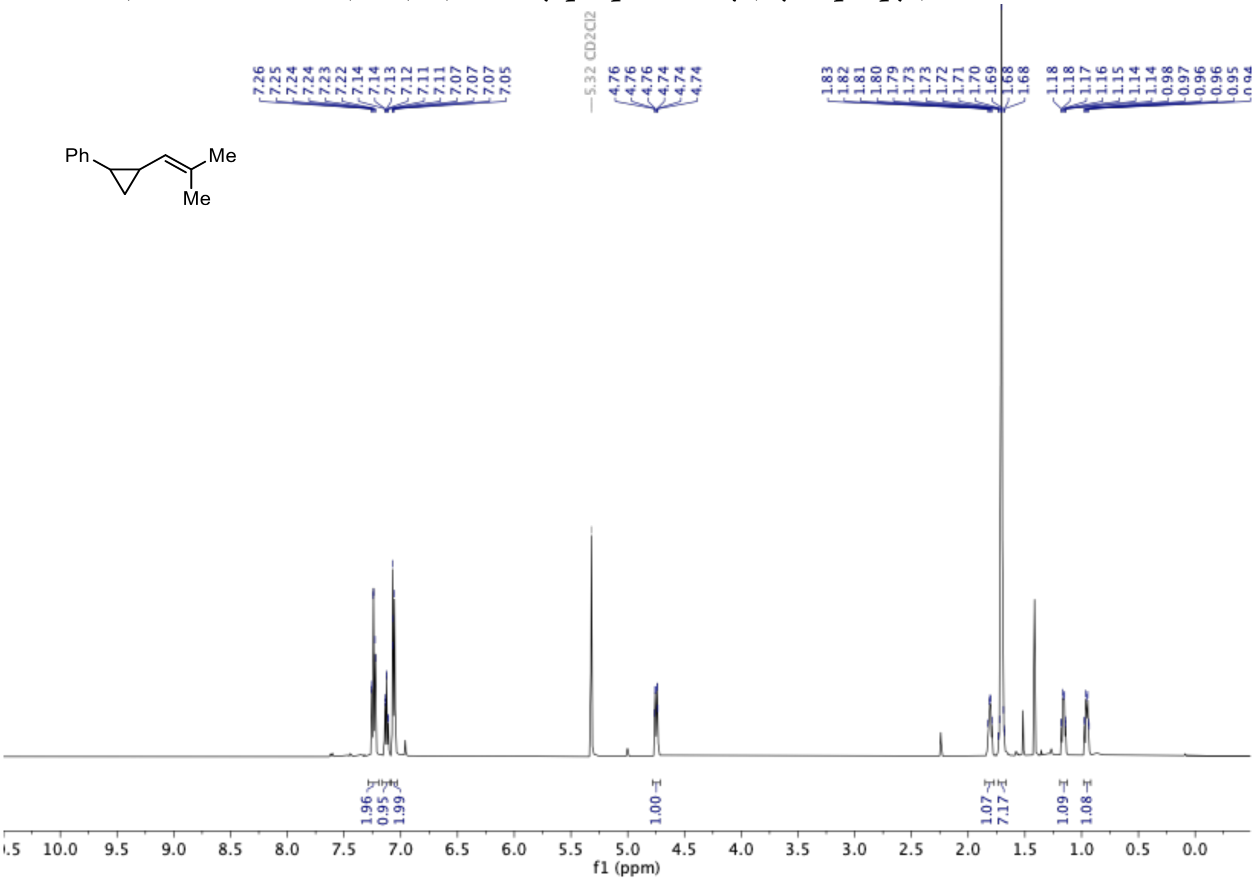

^{13}C NMR (126 MHz, $CDCl_3$) of (2-(2-methylprop-1-en-1-yl)cyclopropyl)benzene

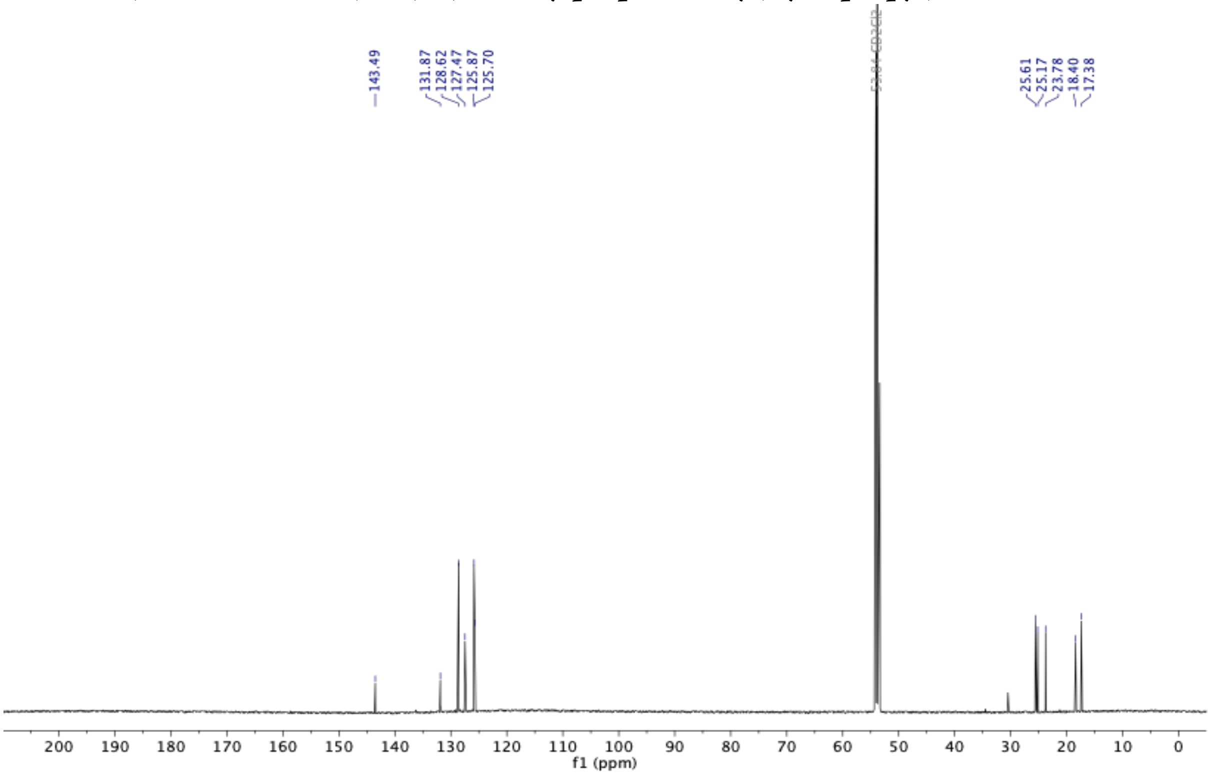

^{1}H NMR Yield (500 MHz, $CDCl_3$) of Crude Radical Clock Study using (2-vinylcyclopropyl)benzene

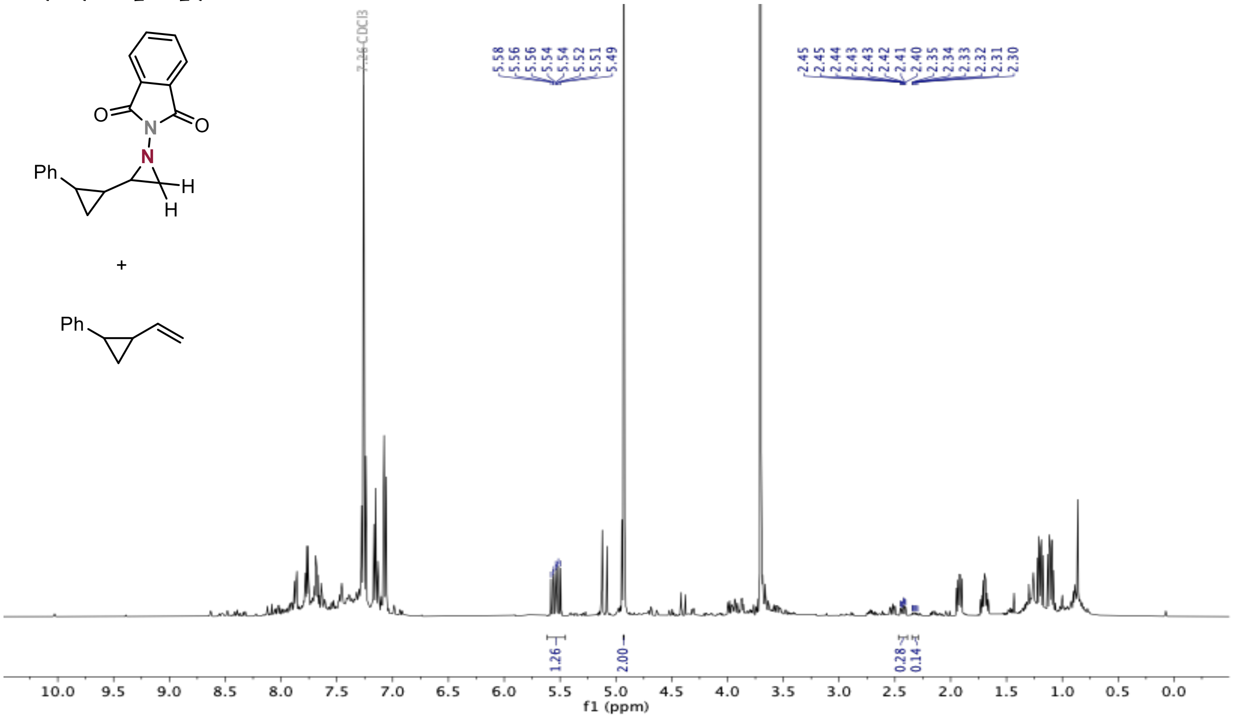

^{1}H NMR Yield (500 MHz, $CDCl_3$) of Crude Radical Clock Study using (2-(2-methylprop-1-en-1-yl)cyclopropyl)benzene

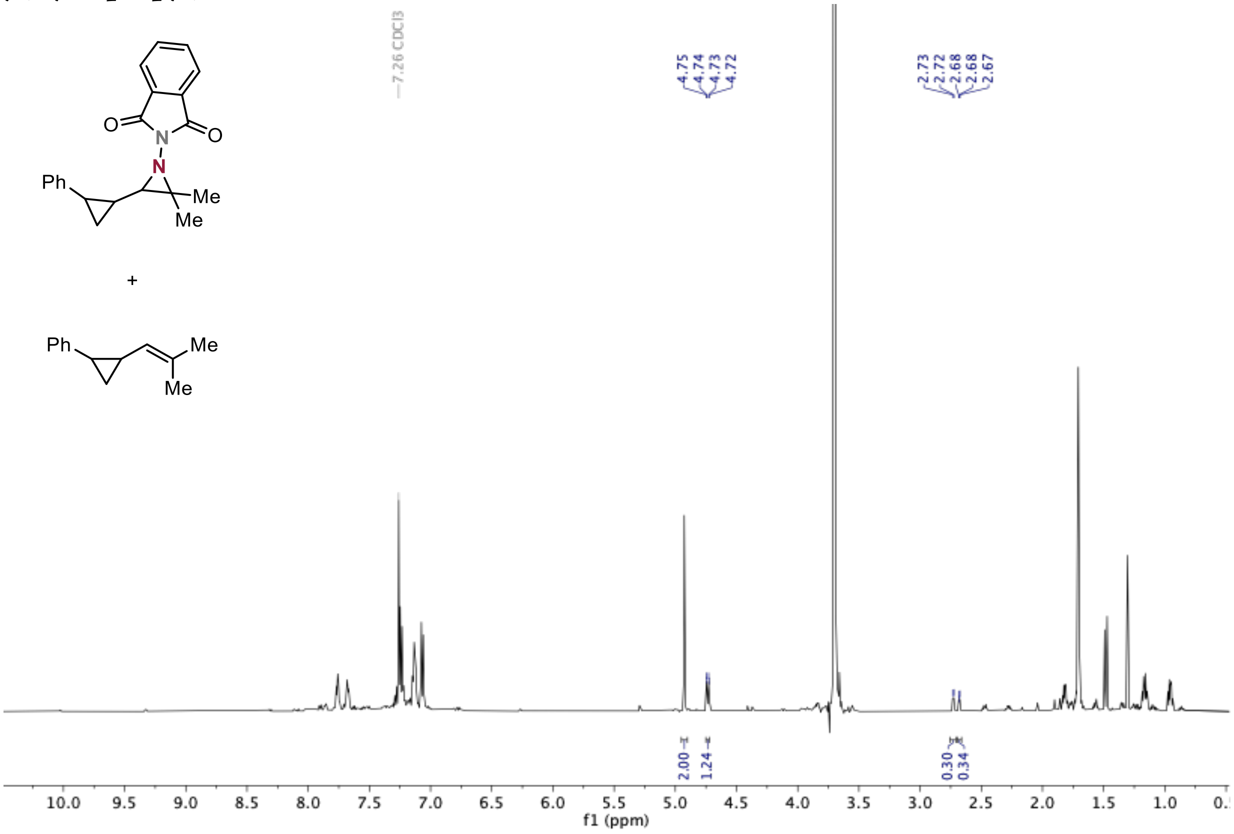

^{1}H NMR (500 MHz, $CDCl_3$) of 2-(2-(2-phenylcyclopropyl)aziridin-1-yl)isoindoline-1,3-dione

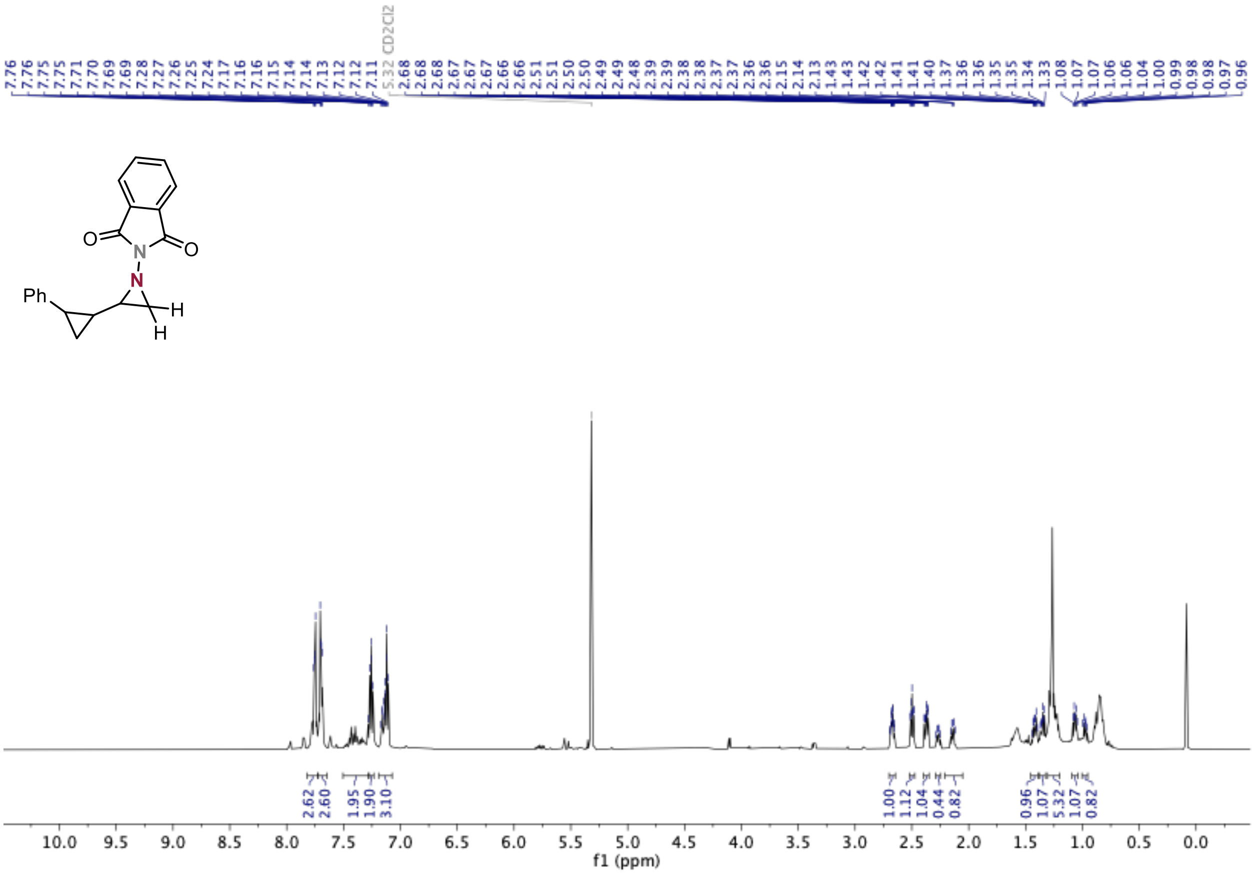

^{13}C NMR (126 MHz, $CDCl_3$) of 2-(2-(2-phenylcyclopropyl)aziridin-1-yl)isoindoline-1,3-dione

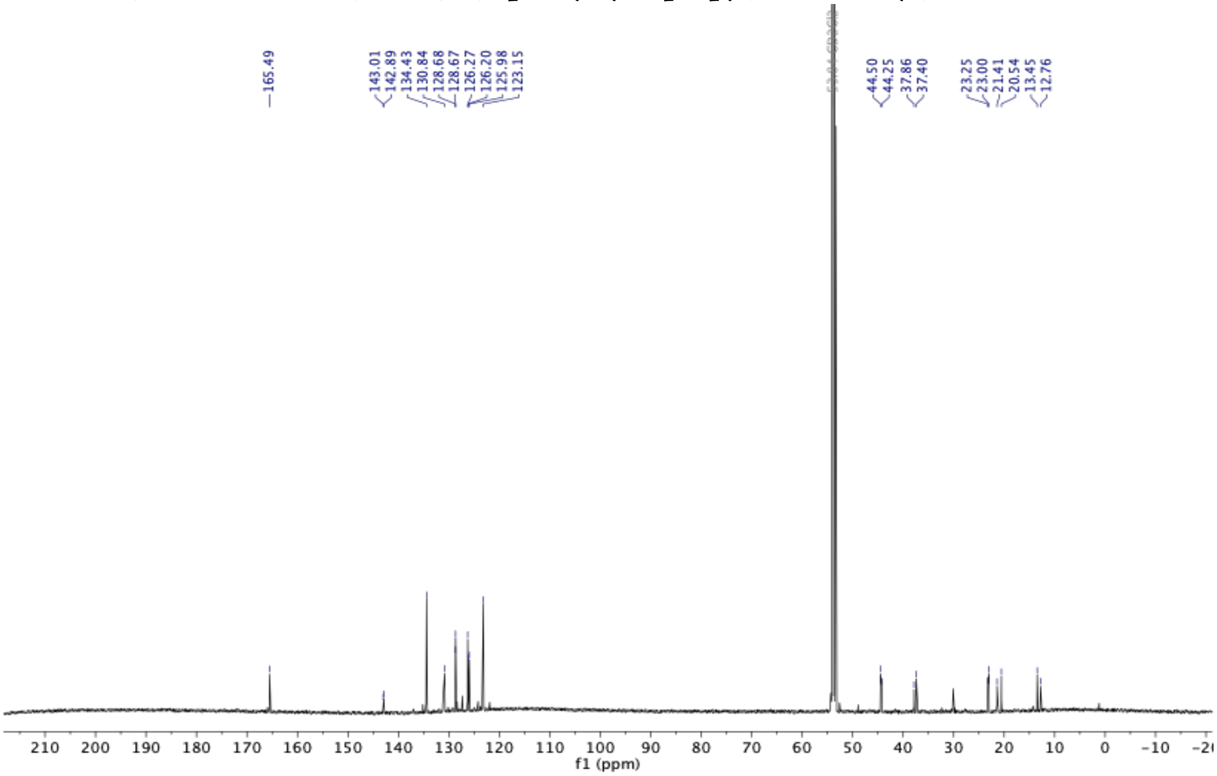

^{1}H NMR (500 MHz, CDCl$_3$) of 2-(2,2-dimethyl-3-(2-phenylcyclopropyl)aziridin-1-yl)isoindoline-1,3-dione

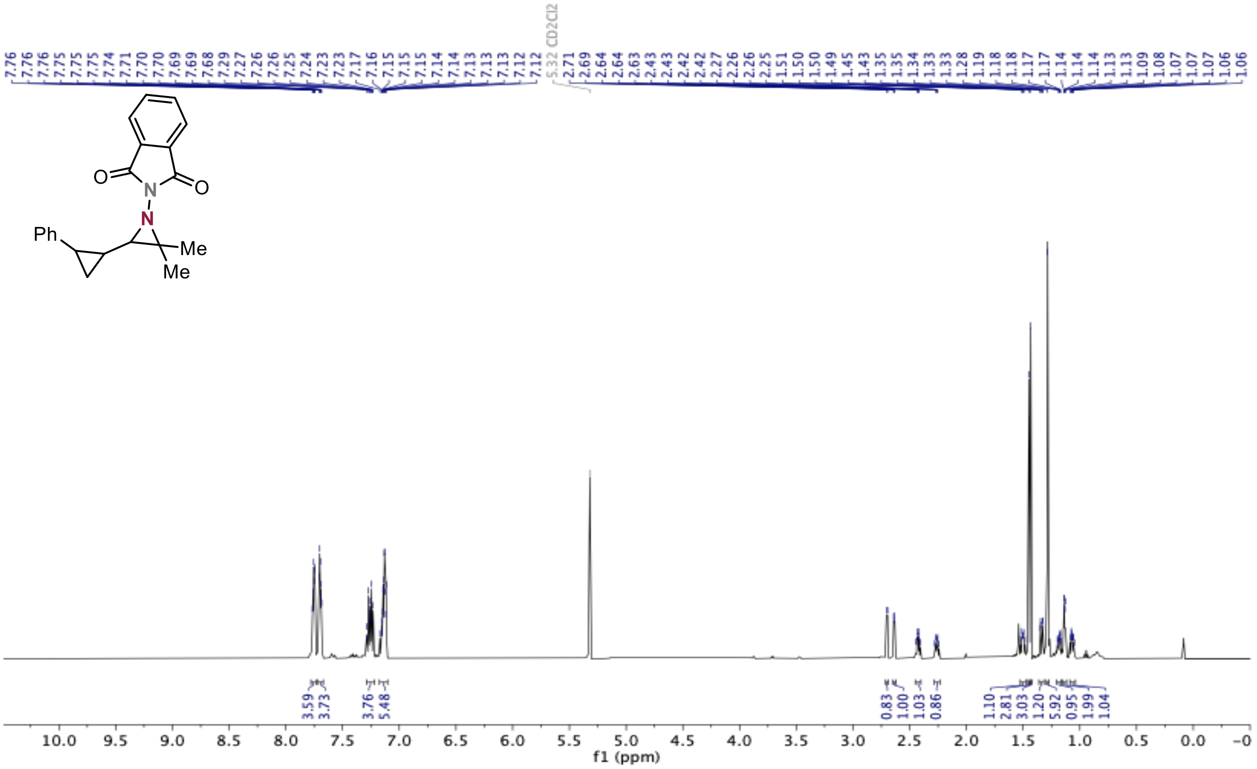

^{13}C NMR (126 MHz, CDCl$_3$) of 2-(2,2-dimethyl-3-(2-phenylcyclopropyl)aziridin-1-yl)isoindoline-1,3-dione

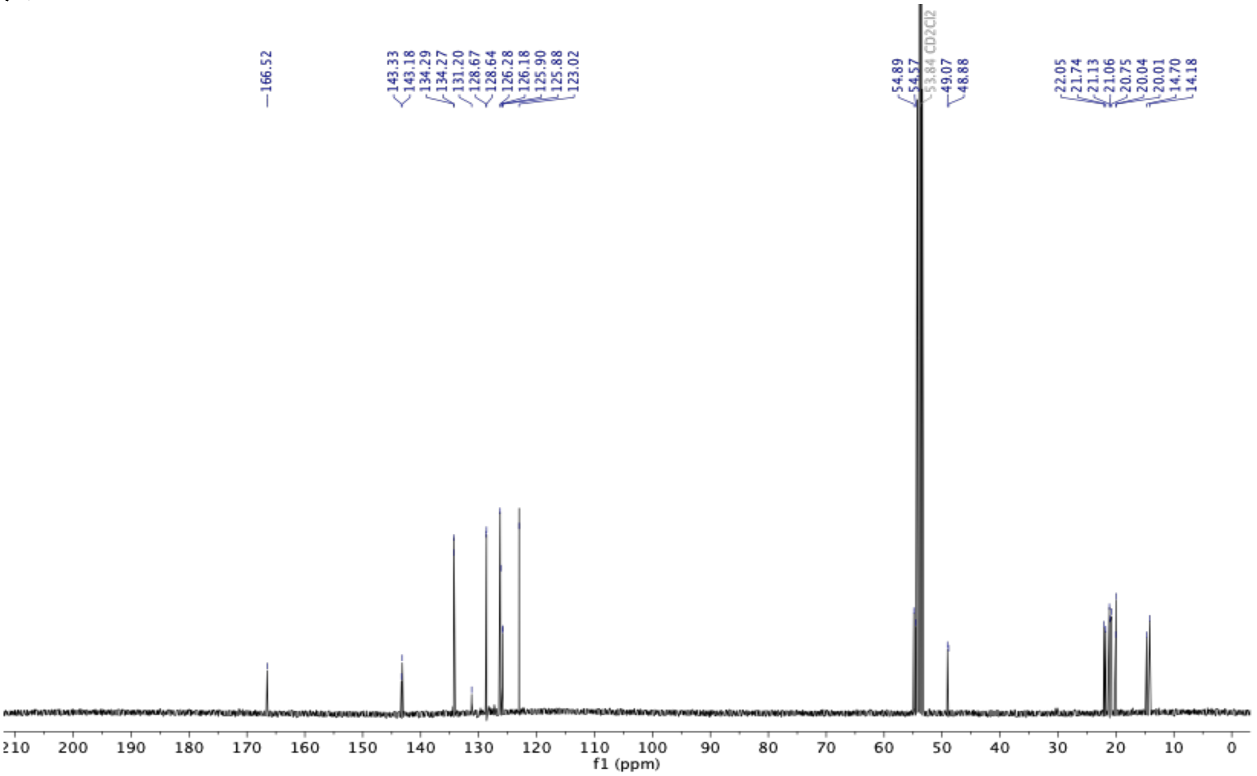

NMR spectra

NMR spectra of Isolated Compounds

^{1}H NMR (400 MHz, $CDCl_3$) of 2-(2-(4-fluorophenyl)aziridin-1-yl)isoindoline-1,3-dione (2a)

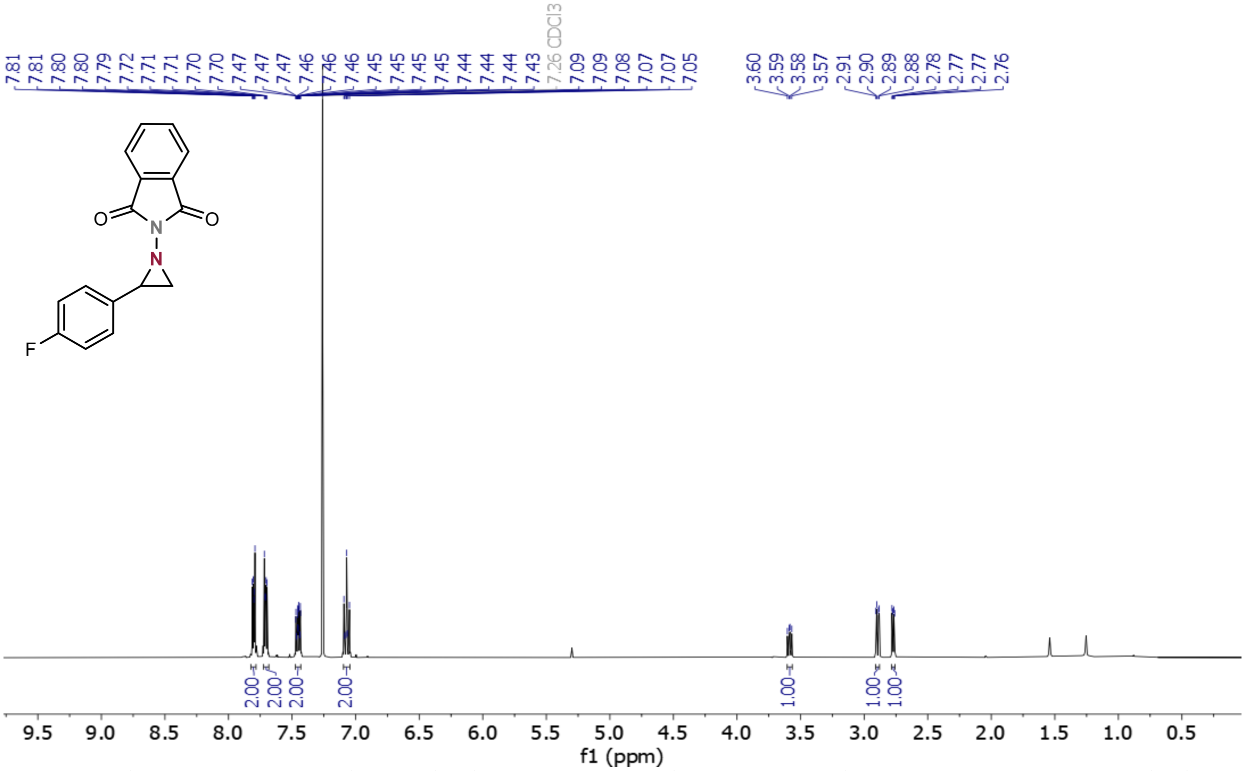

^{13}CNMR (101 MHz, $CDCl_3$) of 2-(2-(4-fluorophenyl)aziridin-1-yl)isoindoline-1,3-dione (2a)

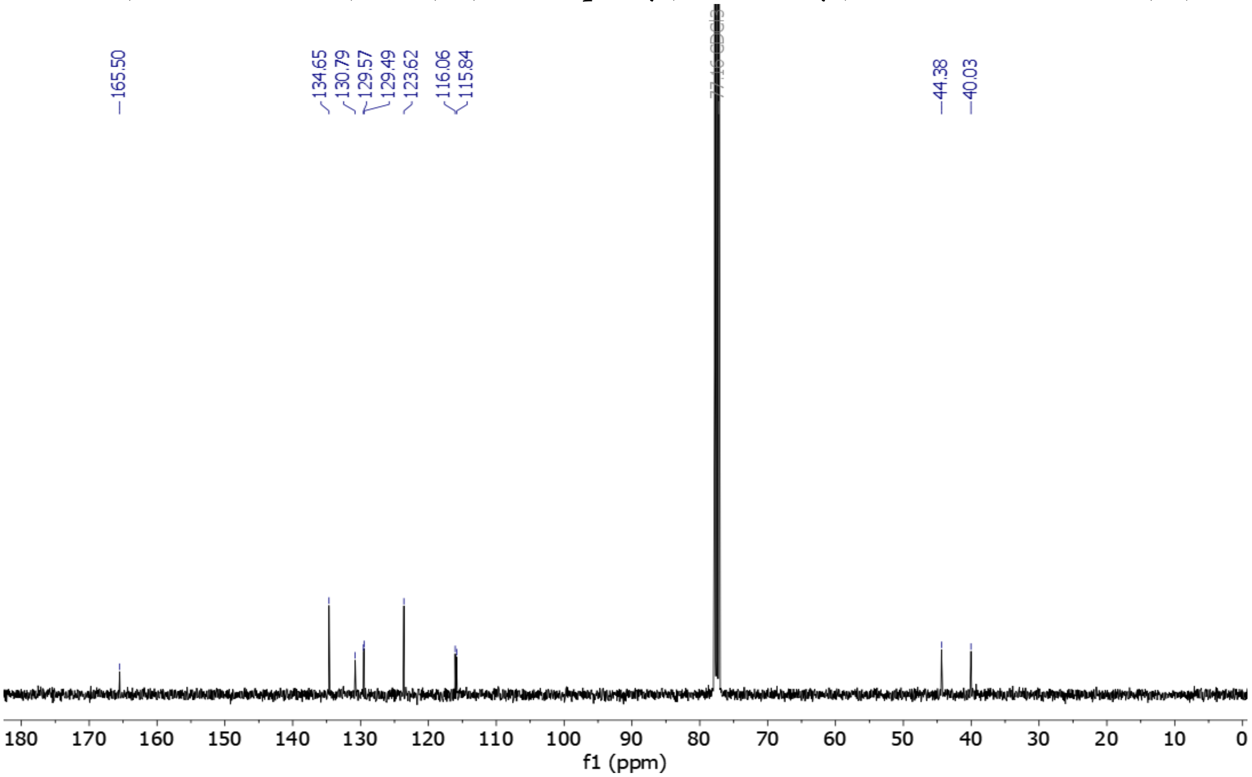

^{1}H NMR (400 MHz, $CDCl_3$) of 2-(2-phenylaziridin-1-yl)isoindoline-1,3-dione (2b)

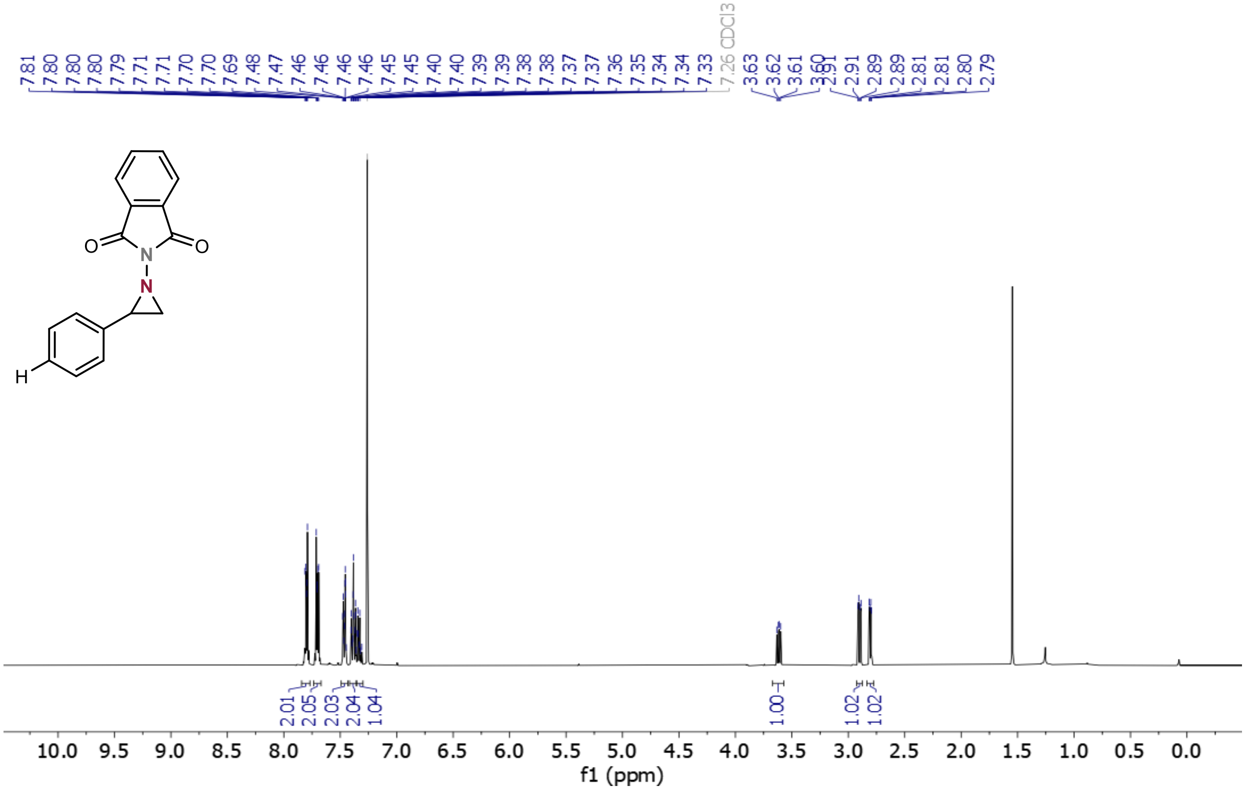

^{13}CNMR (101 MHz, $CDCl_3$) of 2-(2-phenylaziridin-1-yl)isoindoline-1,3-dione (2b)

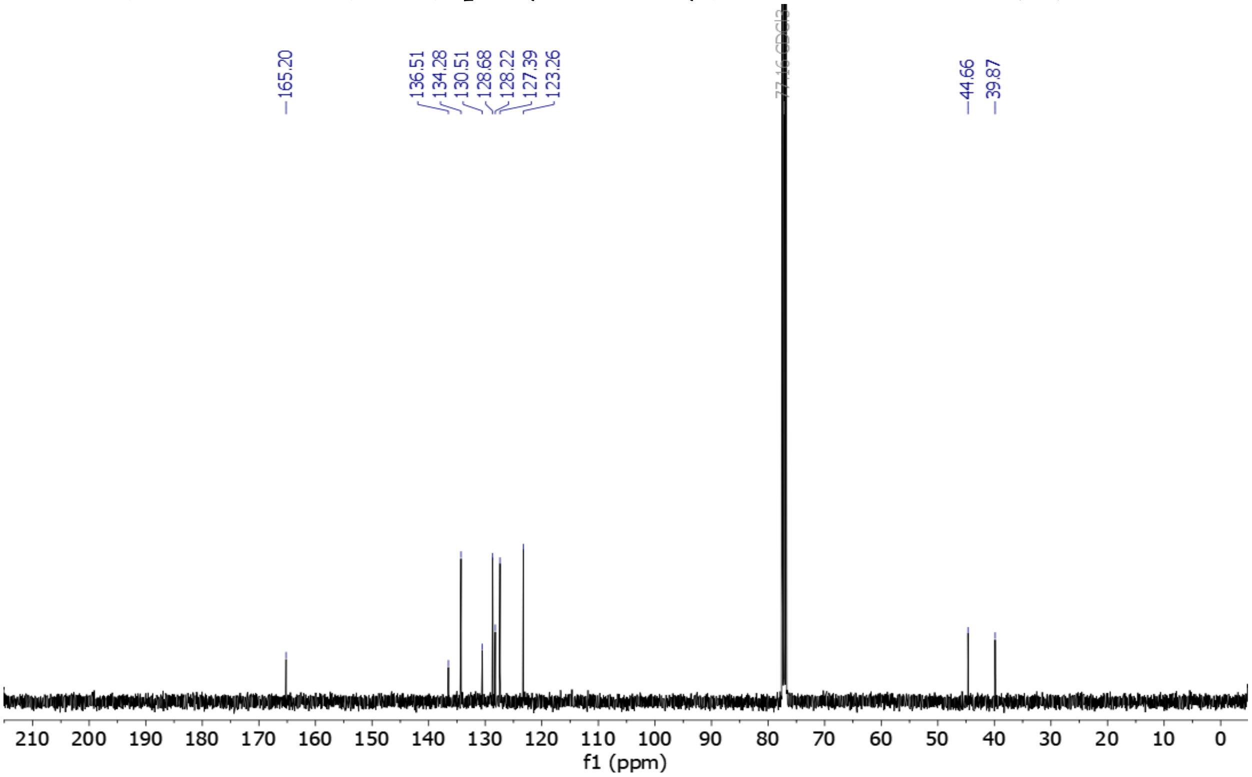

^{1}H NMR (500 MHz, $CDCl_3$) of 2-(2-(*p*-tolyl)aziridin-1-yl)isoindoline-1,3-dione (2c)

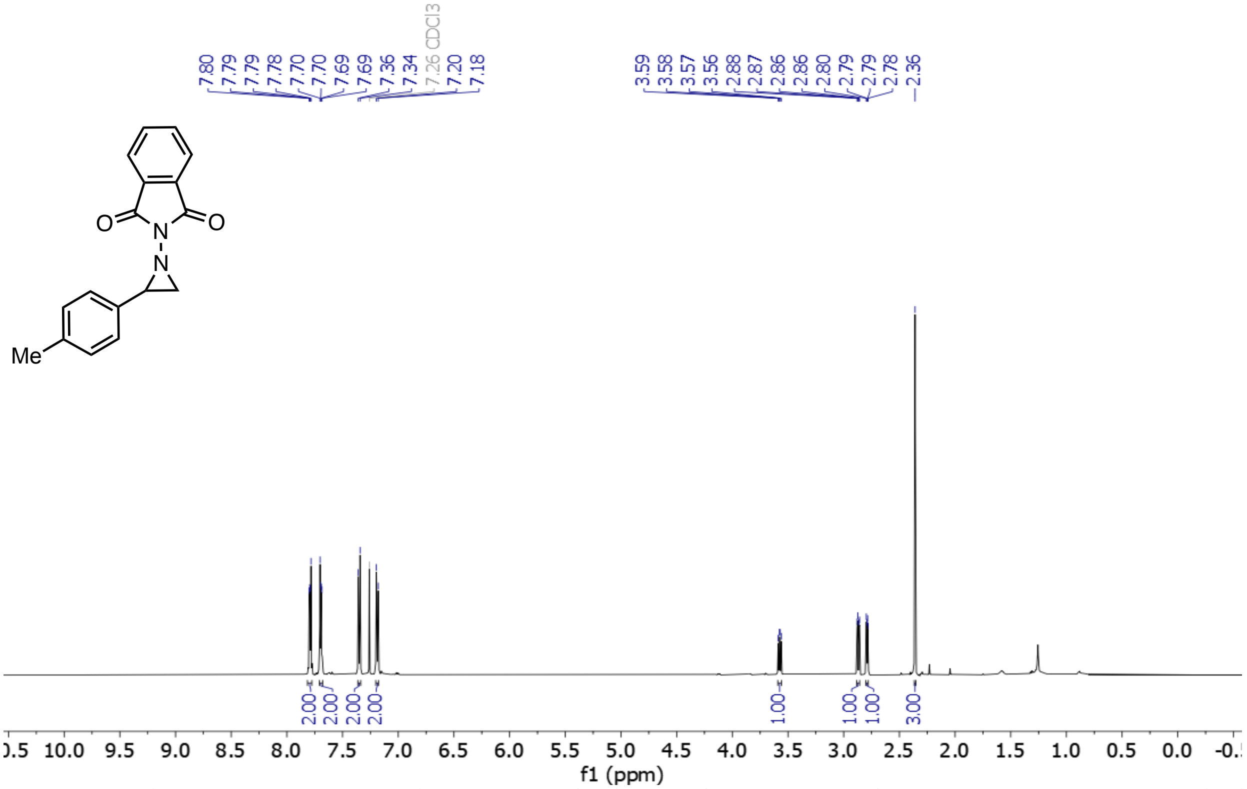

^{13}CNMR (126 MHz, $CDCl_3$) of 2-(2-(*p*-tolyl)aziridin-1-yl)isoindoline-1,3-dione (2c)

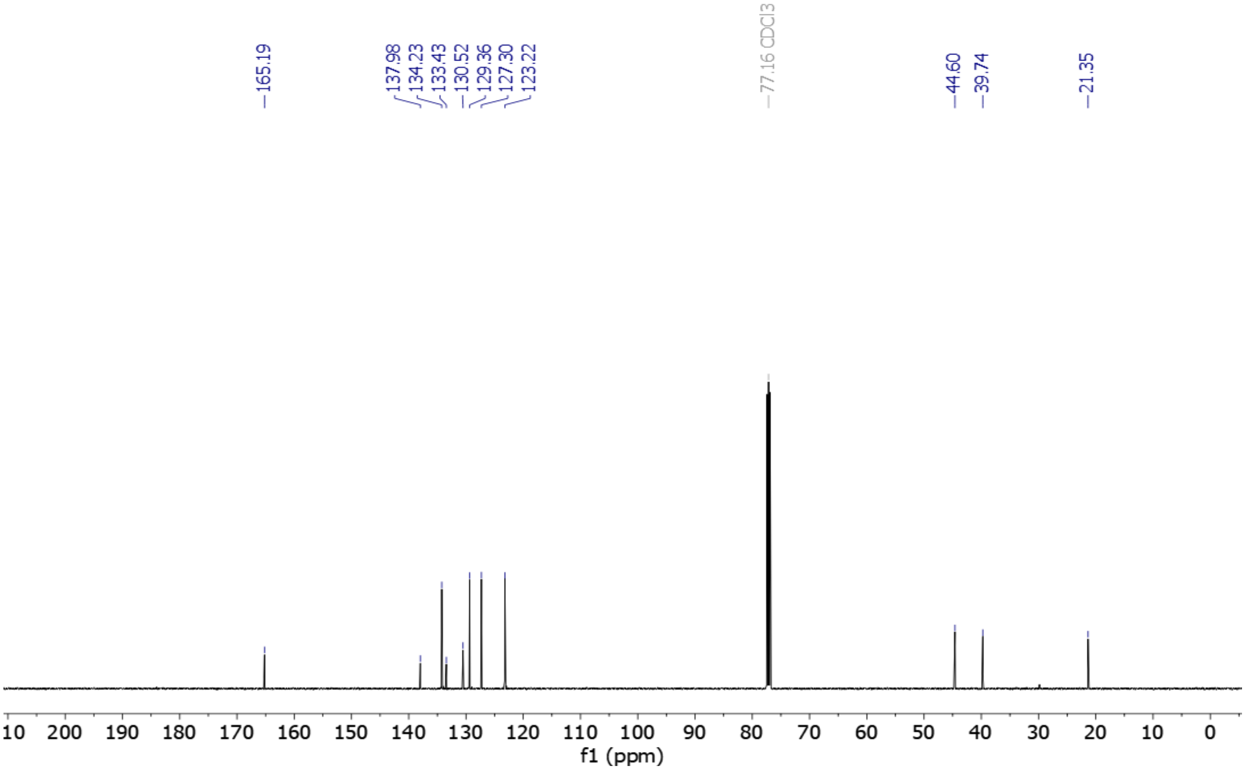

^{1}H NMR (400 MHz, $CDCl_3$) of 2-(2-(4-(*tert*-butyl)phenyl)aziridin-1-yl)isoindoline-1,3-dione (**2d**)

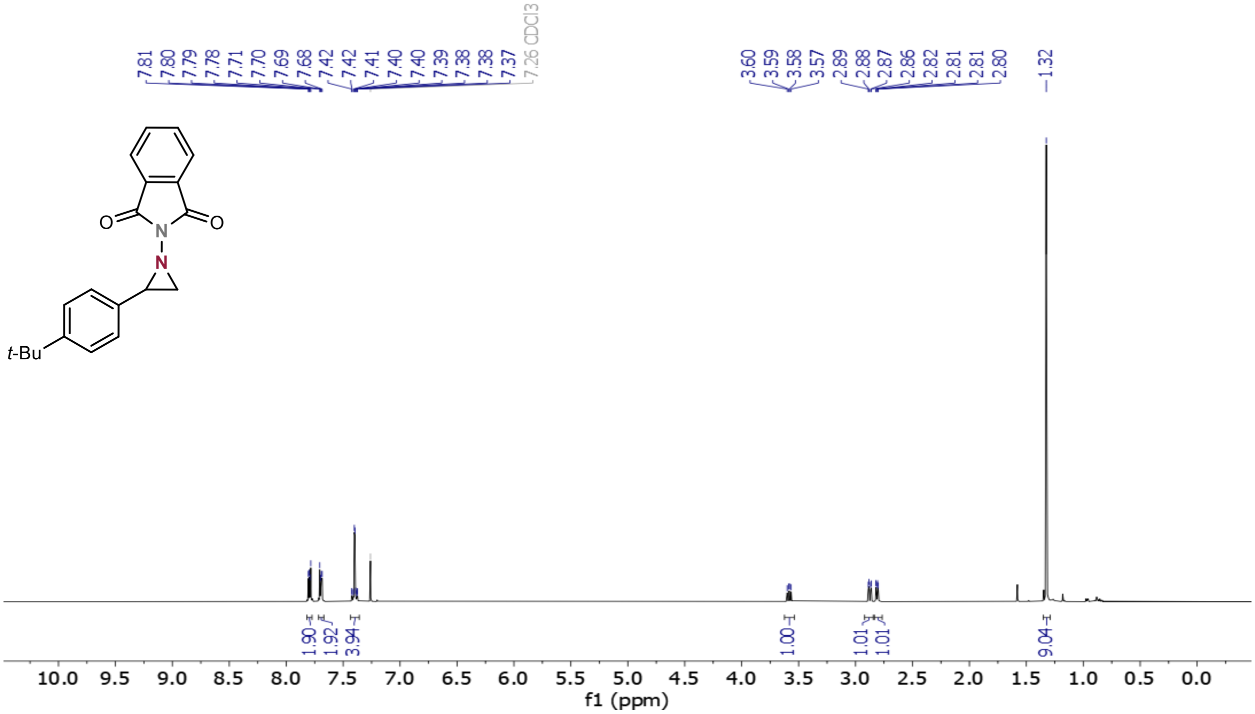

^{13}C NMR (101 MHz, $CDCl_3$) of 2-(2-(4-(*tert*-butyl)phenyl)aziridin-1-yl)isoindoline-1,3-dione (**2d**)

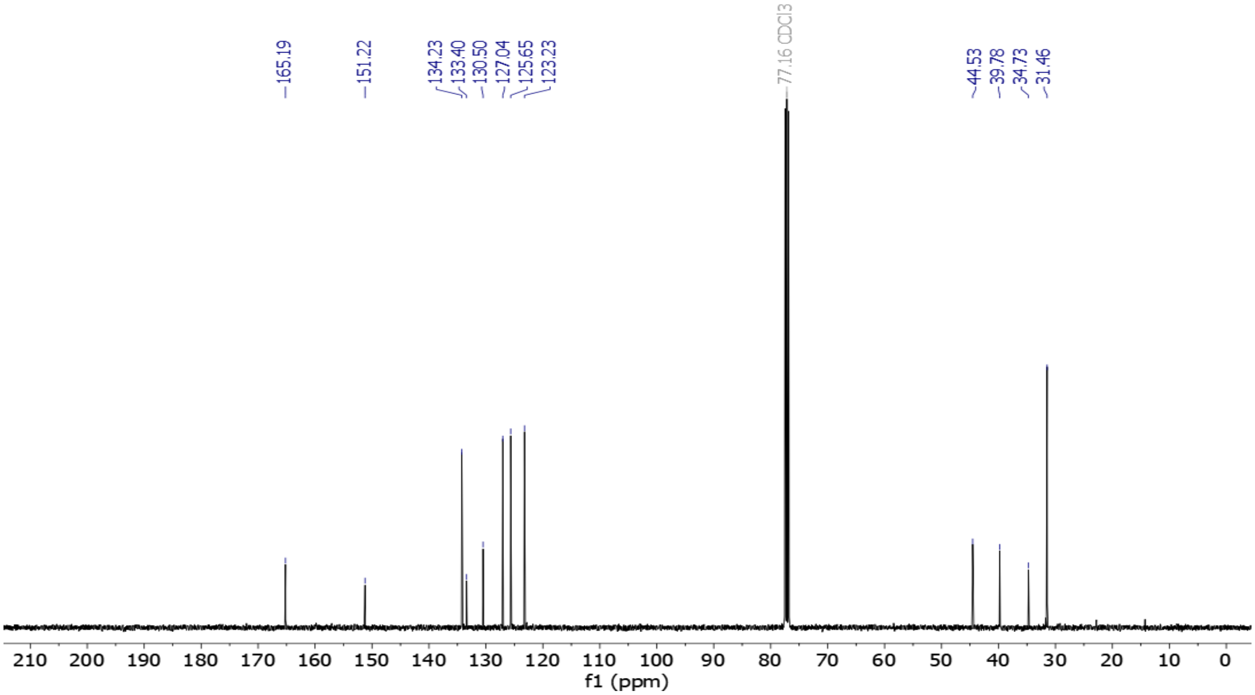

^{1}H NMR (400 MHz, $CDCl_3$) of 2-(2-(4-chlorophenyl)aziridin-1-yl)isoindoline-1,3-dione (2e)

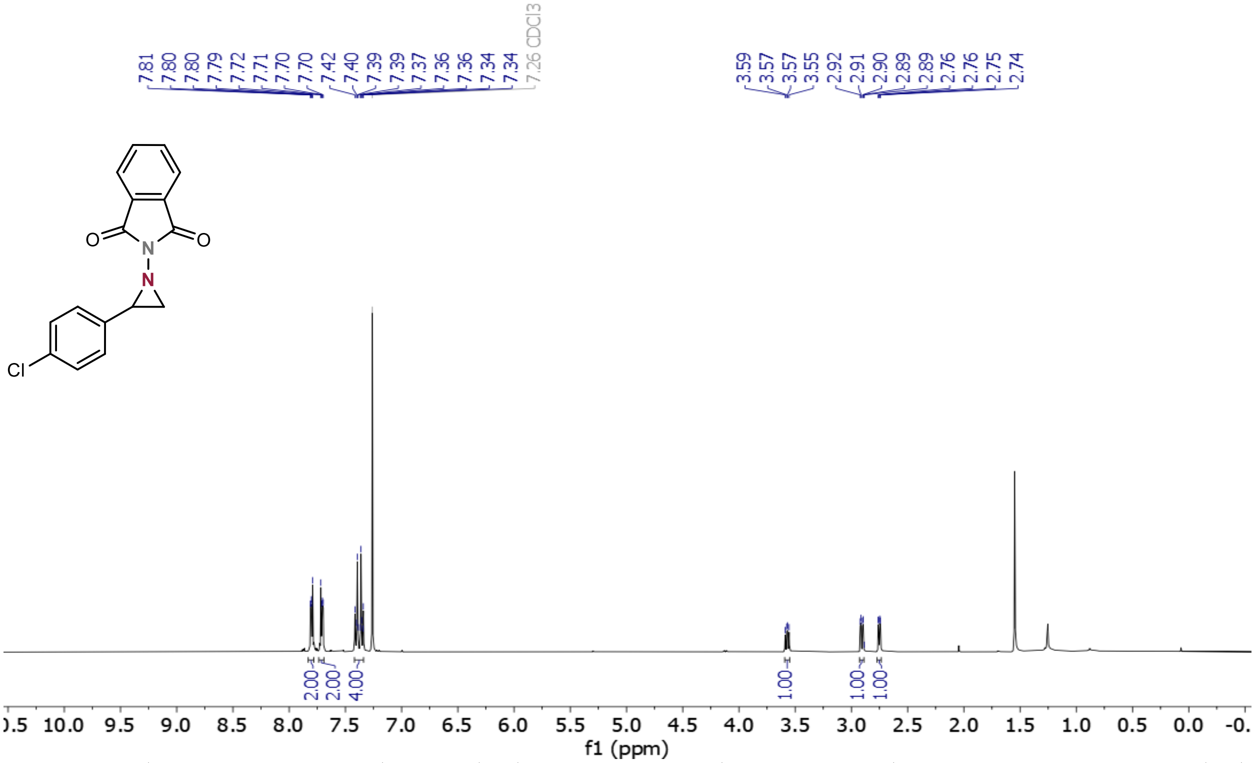

^{13}C NMR (101 MHz, $CDCl_3$) of 2-(2-(4-chlorophenyl)aziridin-1-yl)isoindoline-1,3-dione (2e)

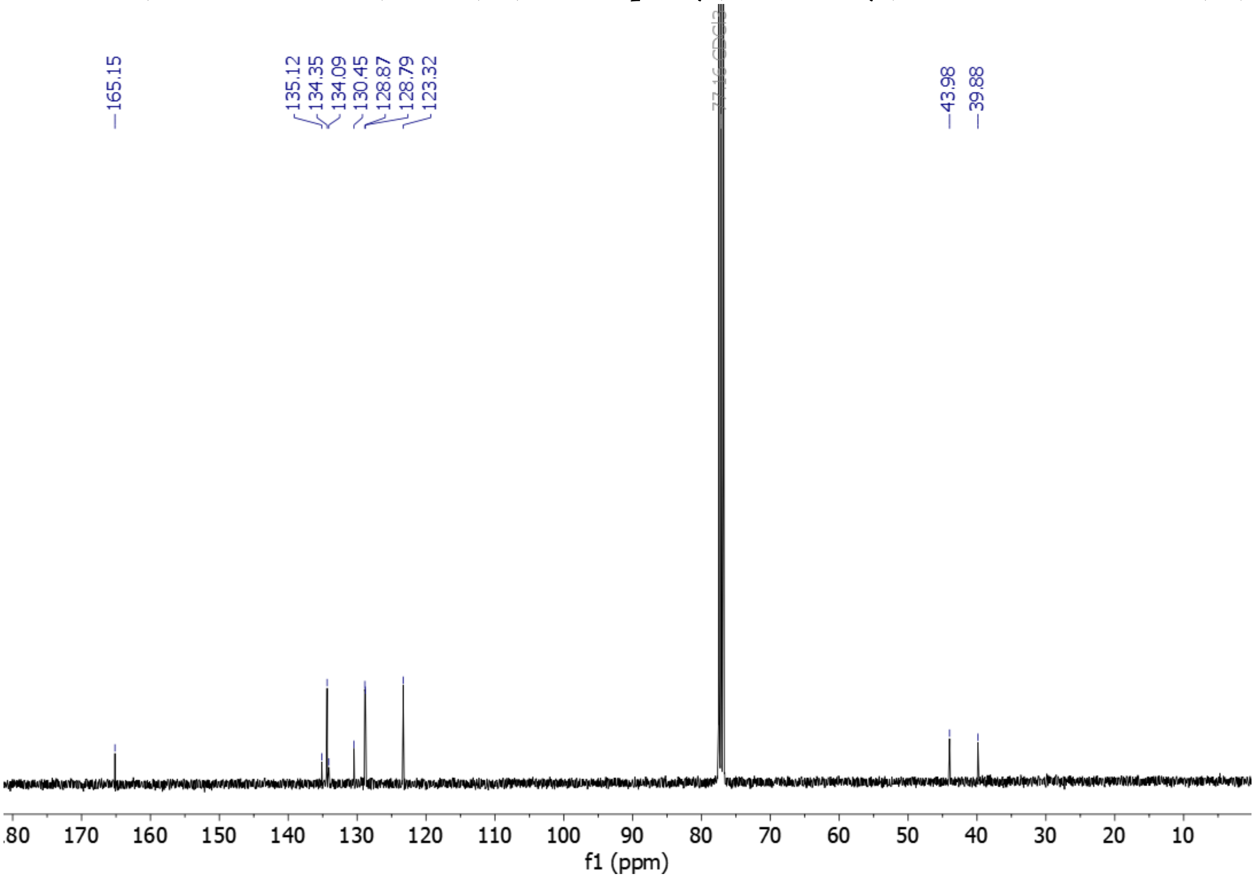

^{1}H NMR (400 MHz, $CDCl_3$) of 2-(2-(4-bromophenyl)aziridin-1-yl)isoindoline-1,3-dione (2f)

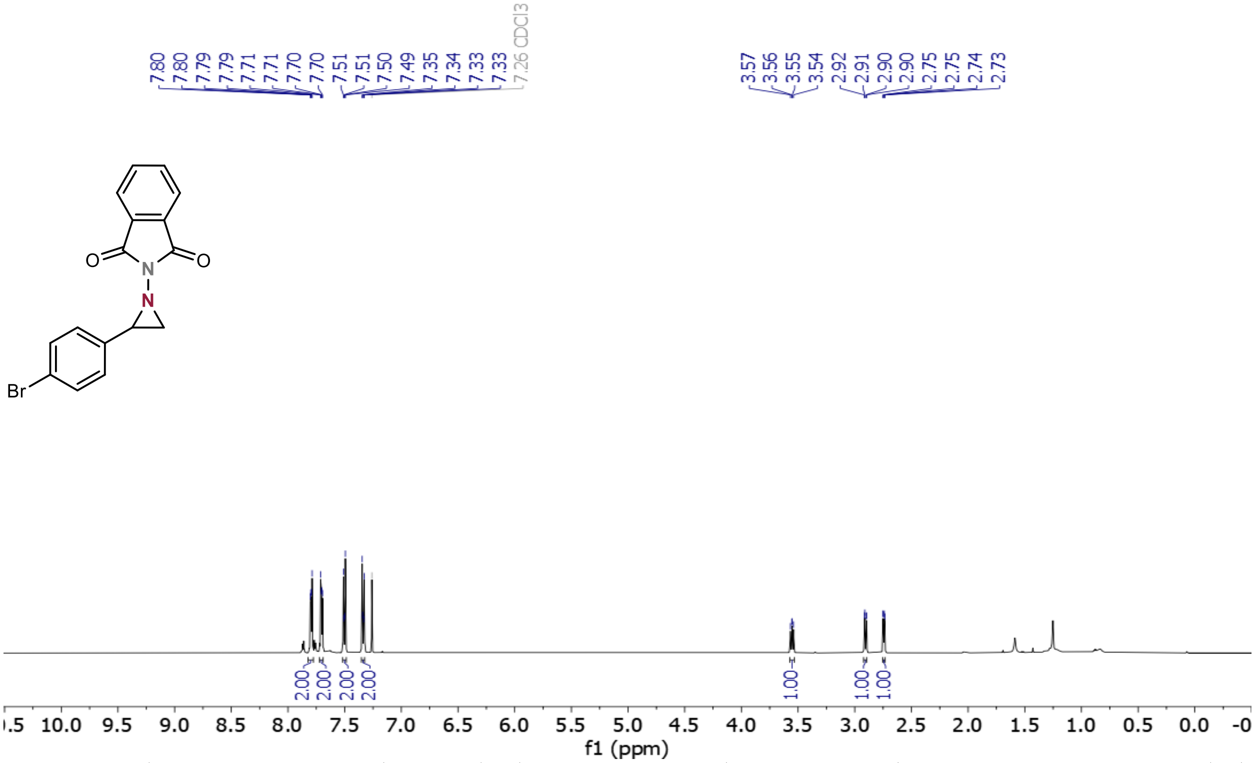

^{13}C NMR (101 MHz, $CDCl_3$) of 2-(2-(4-bromophenyl)aziridin-1-yl)isoindoline-1,3-dione (2f)

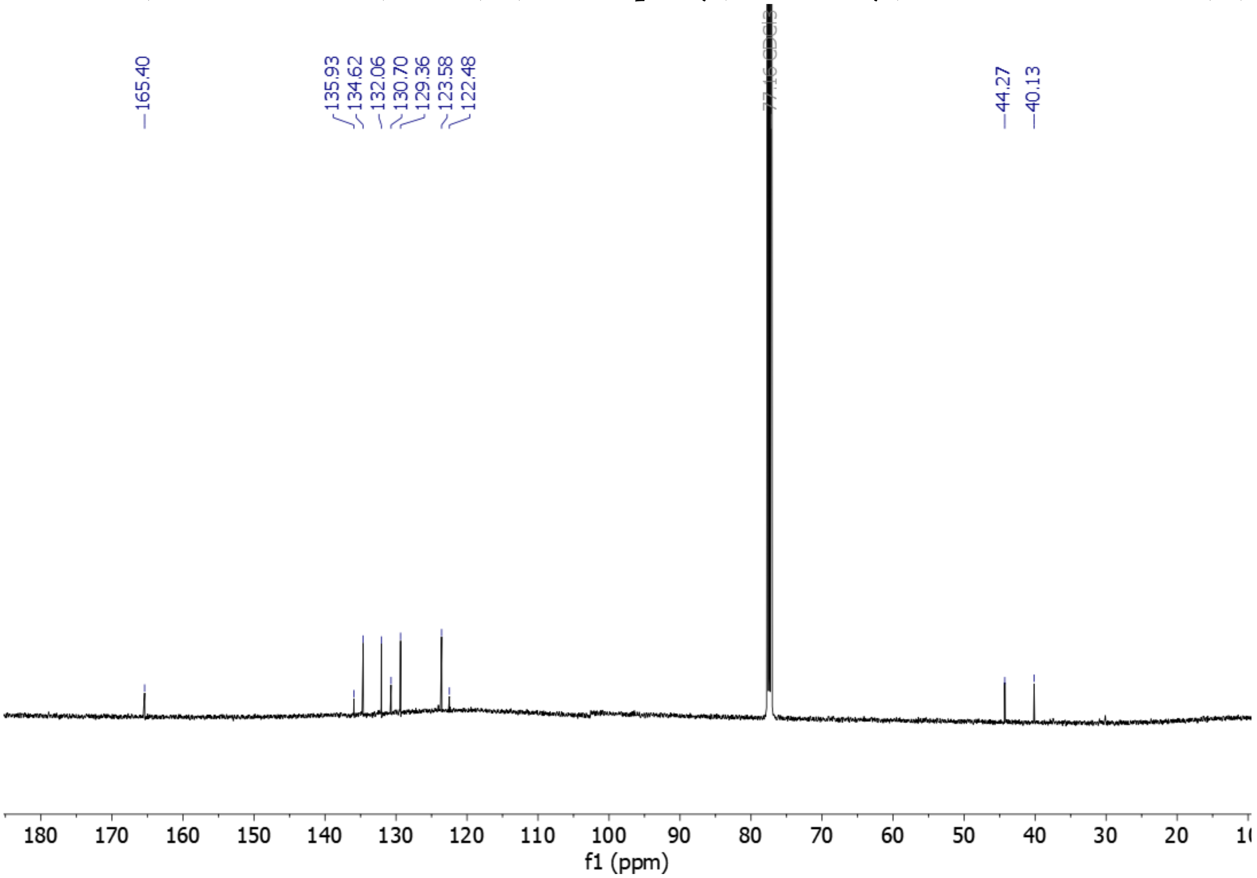

^{1}H NMR (400 MHz, $CDCl_3$) of 4-(1-(1,3-dioxoisoindolin-2-yl)aziridin-2-yl)phenyl acetate (2g)

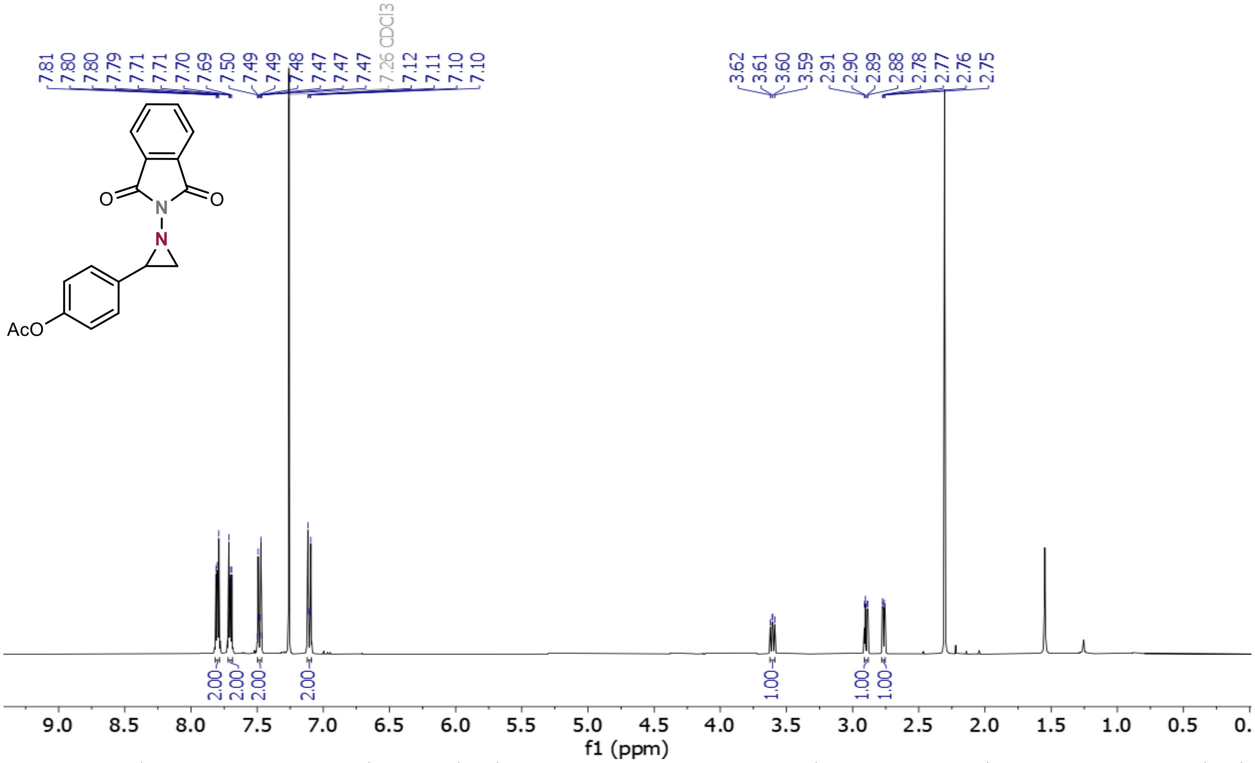

^{13}C NMR (101 MHz, $CDCl_3$) of 4-(1-(1,3-dioxoisoindolin-2-yl)aziridin-2-yl)phenyl acetate (2g)

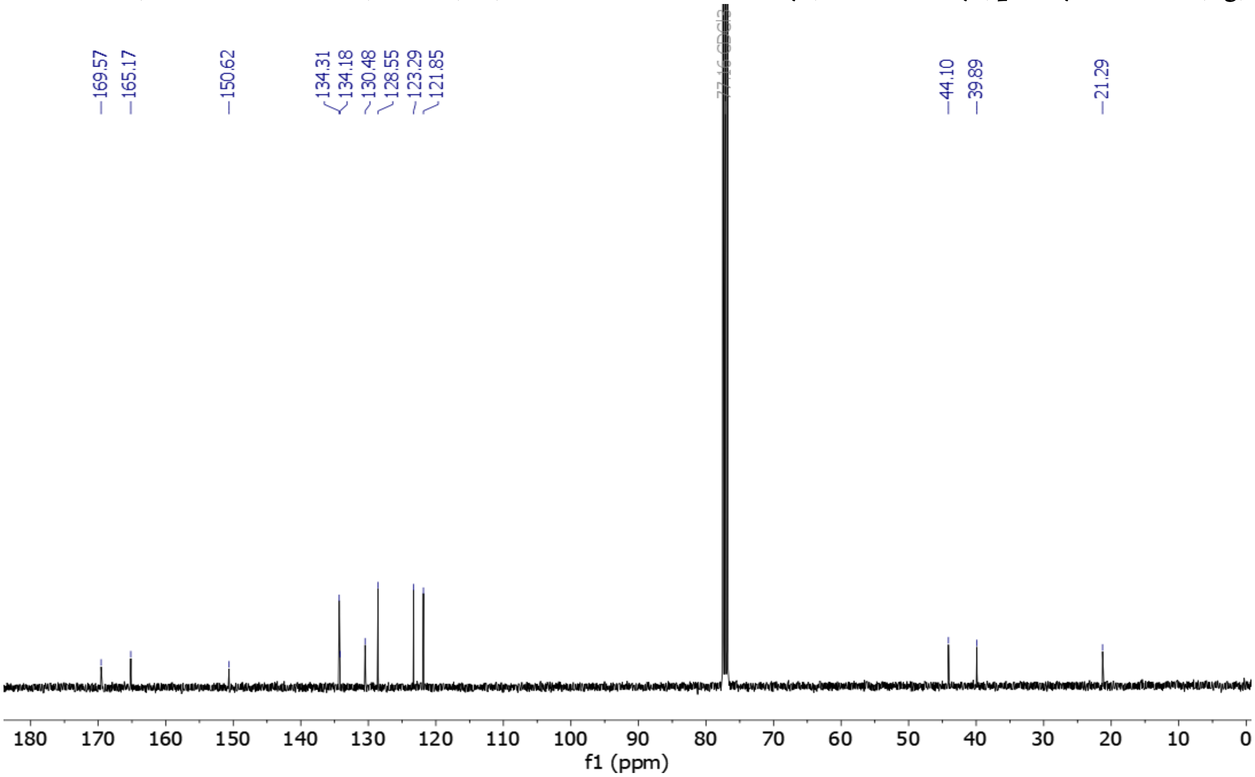

^{1}H NMR (400 MHz, $CDCl_3$) of 2-(2-(4-methoxyphenyl)aziridin-1-yl)isoindoline-1,3-dione (2h)

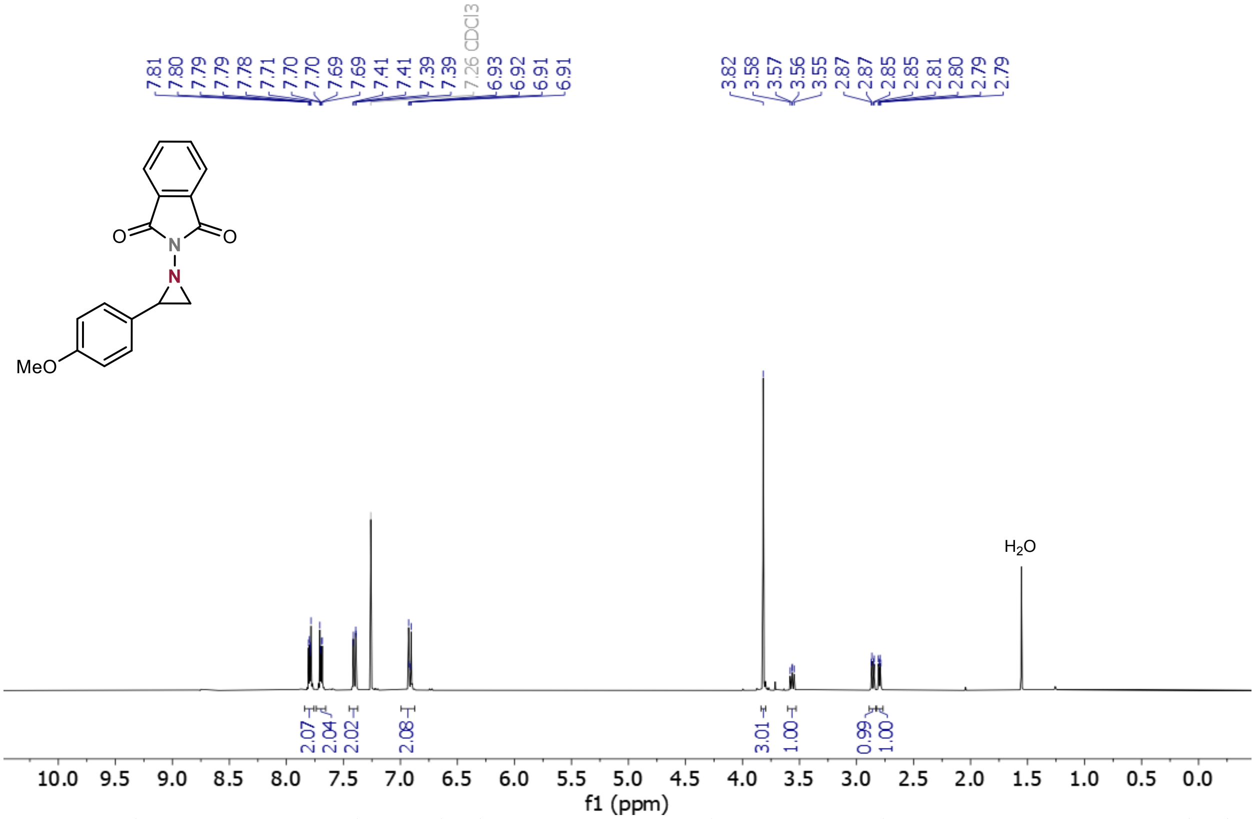

^{13}C NMR (101 MHz, $CDCl_3$) of 2-(2-(4-methoxyphenyl)aziridin-1-yl)isoindoline-1,3-dione (2h)

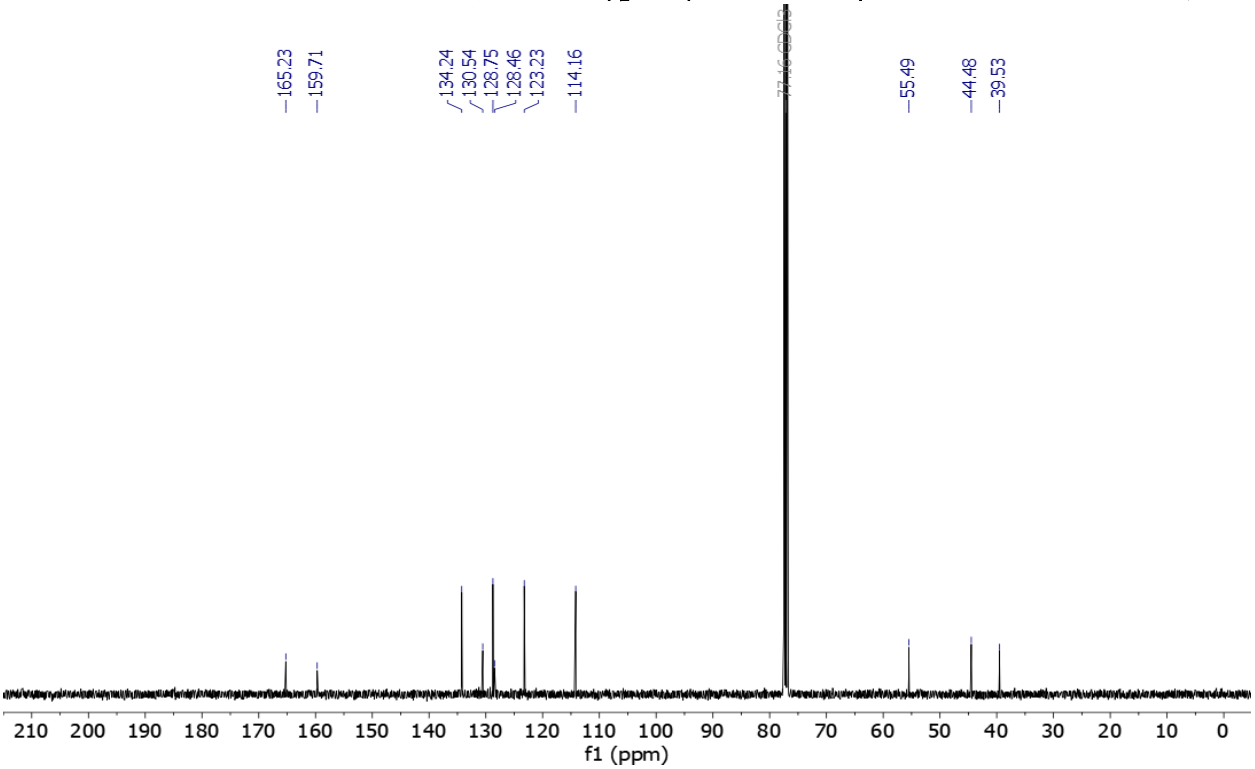

^{1}H NMR (400 MHz, $CDCl_3$) of 4-(1-(1,3-dioxoisoindolin-2-yl)aziridin-2-yl)benzonitrile (2j)

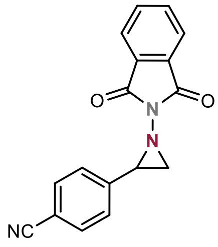

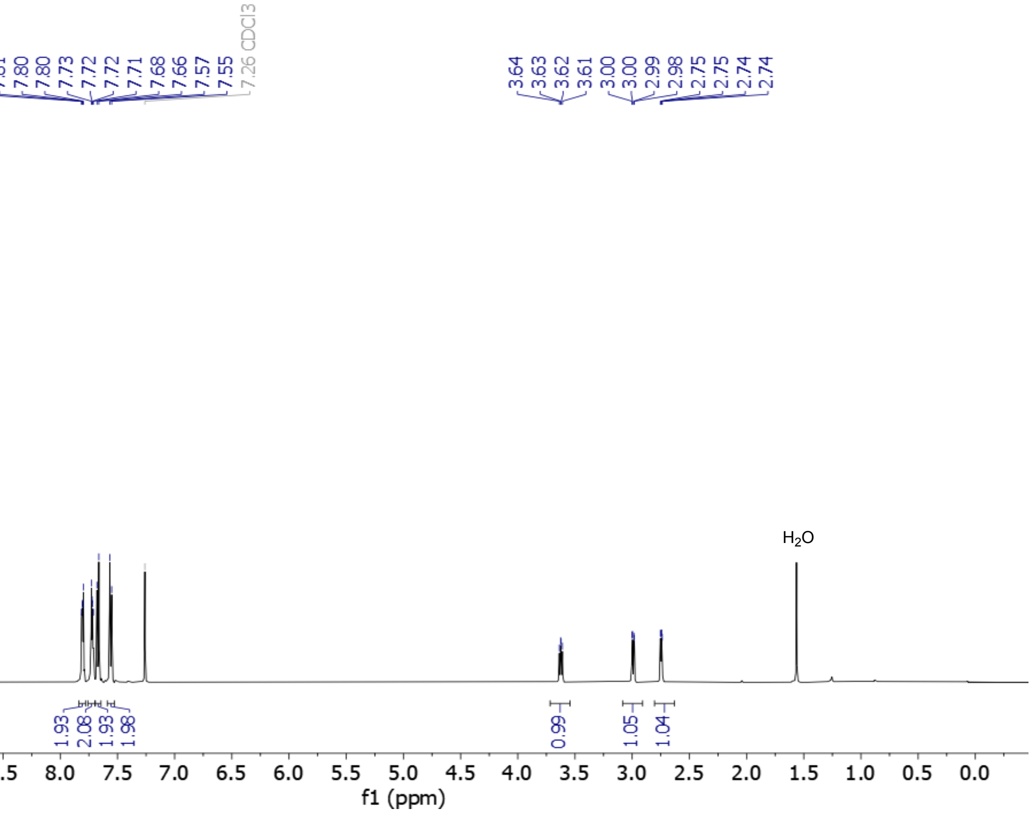

^{13}C NMR (101 MHz, $CDCl_3$) of 4-(1-(1,3-dioxoisoindolin-2-yl)aziridin-2-yl)benzonitrile (2j)

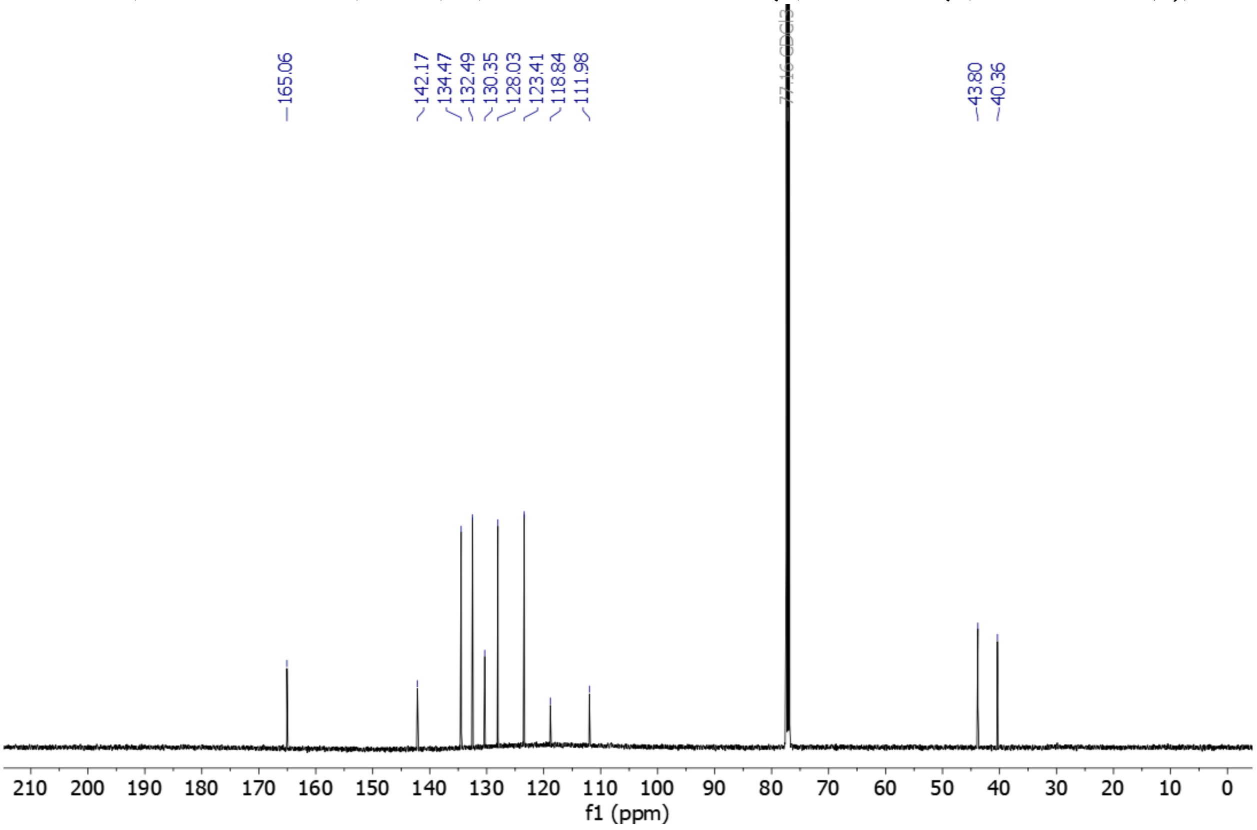

^{1}H NMR (600 MHz, $CDCl_3$) of 2-(2-(4-(trifluoromethyl)phenyl)aziridin-1-yl)isoindoline-1,3-dione (2k)

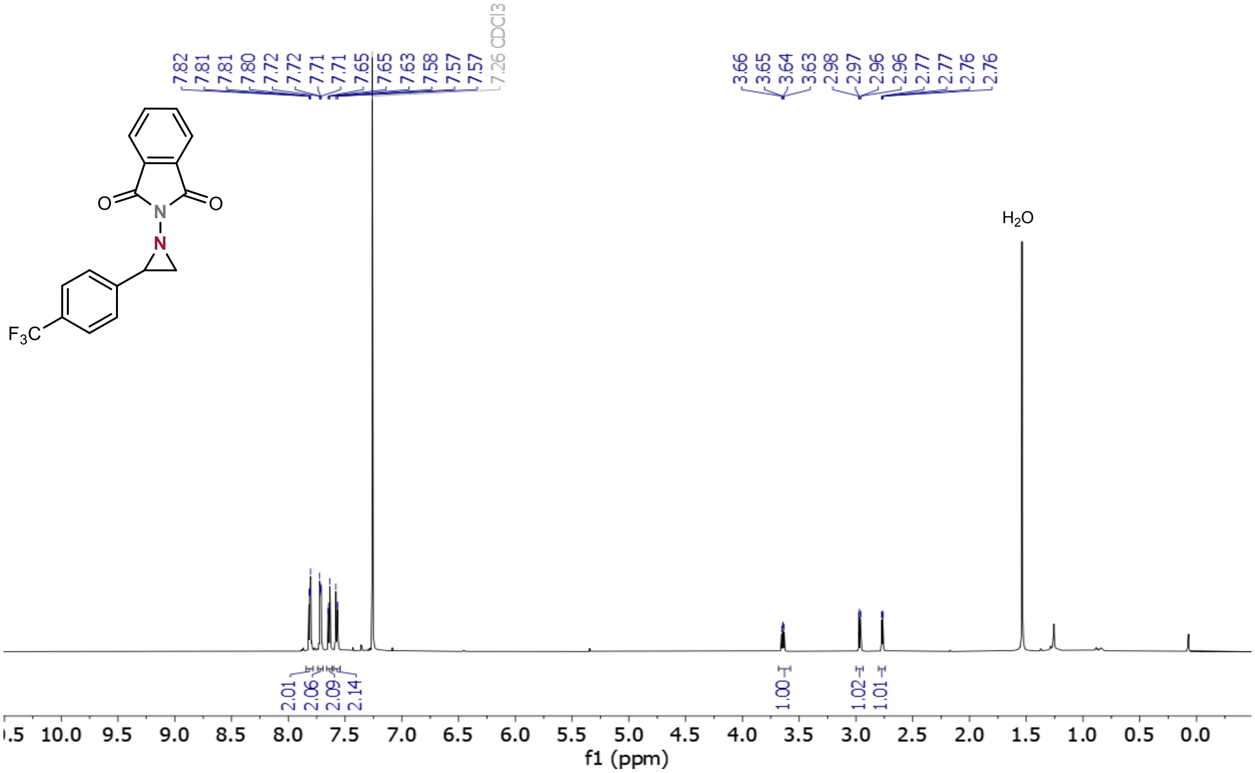

^{13}C NMR (151 MHz, $CDCl_3$) of 2-(2-(4-(trifluoromethyl)phenyl)aziridin-1-yl)isoindoline-1,3-dione (2k)

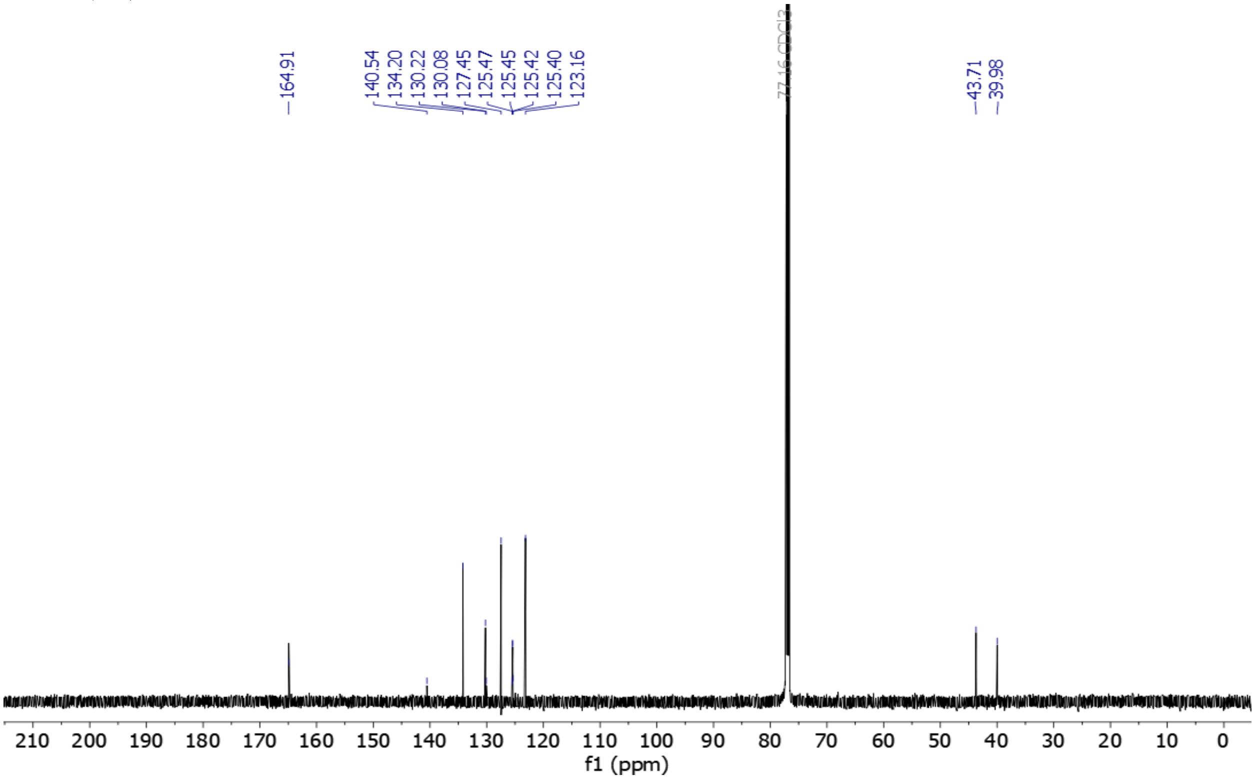

^{1}H NMR (500 MHz, $CDCl_3$) of 4-(1-(1,3-dioxoisoindolin-2-yl)aziridin-2-yl)benzaldehyde (2l)

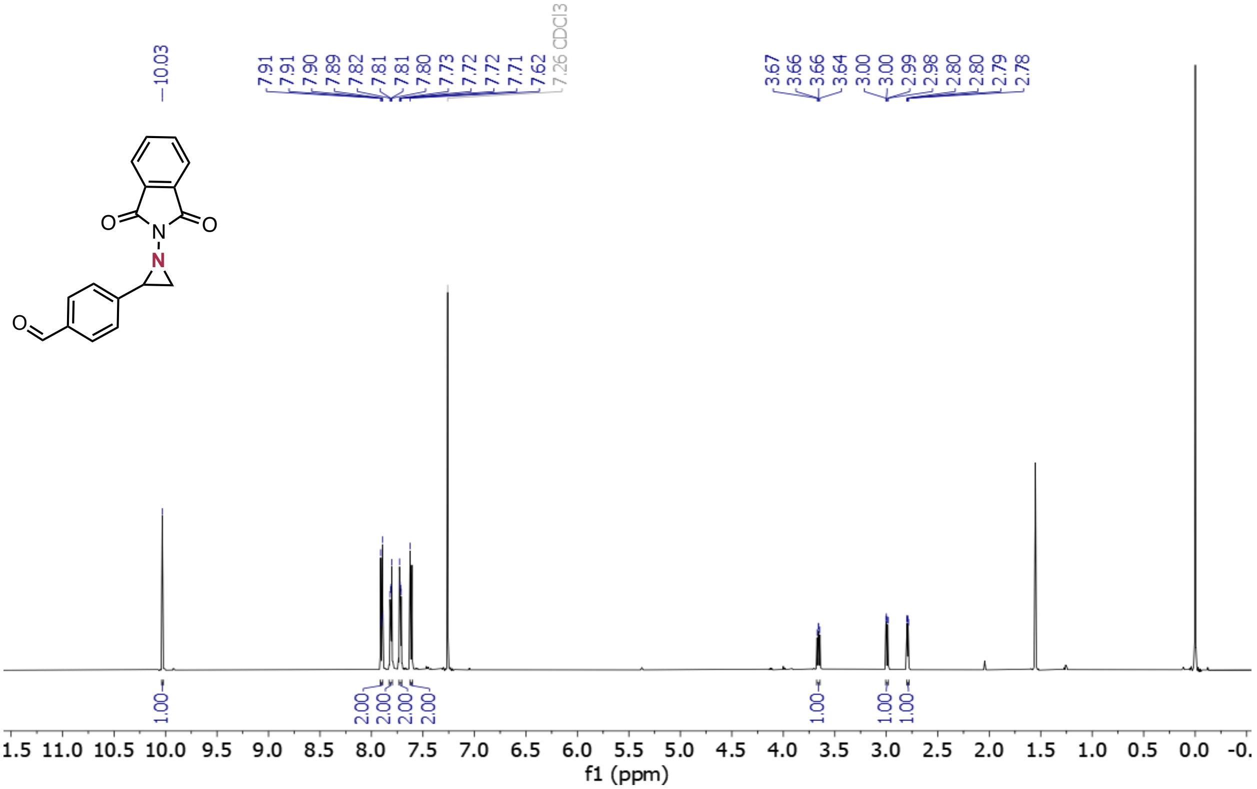

^{13}C NMR (151 MHz, $CDCl_3$) of 4-(1-(1,3-dioxoisoindolin-2-yl)aziridin-2-yl)benzaldehyde (2l)

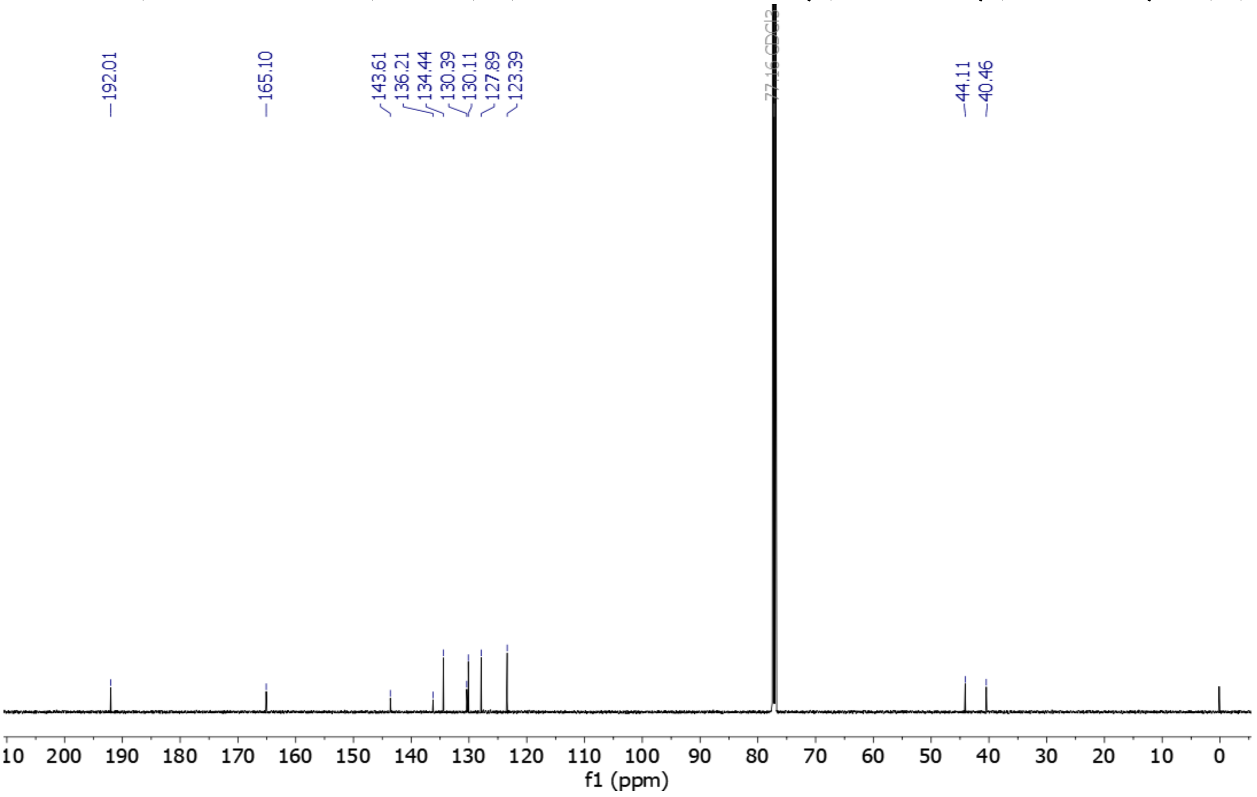

^{1}H NMR (600 MHz, $CDCl_3$) of 2-(2-(2-bromophenyl)aziridin-1-yl)isoindoline-1,3-dione (2m)

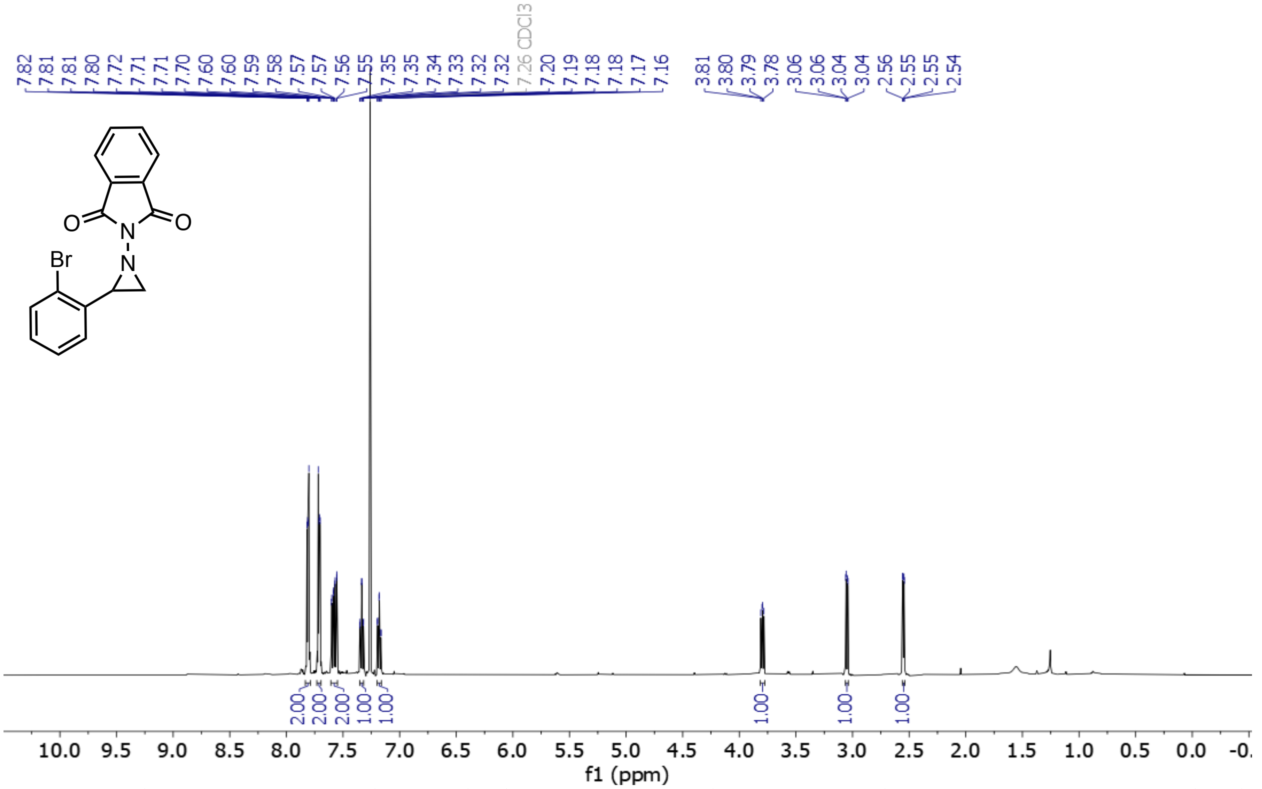

^{13}C NMR (151 MHz, $CDCl_3$) of 2-(2-(2-bromophenyl)aziridin-1-yl)isoindoline-1,3-dione (2m)

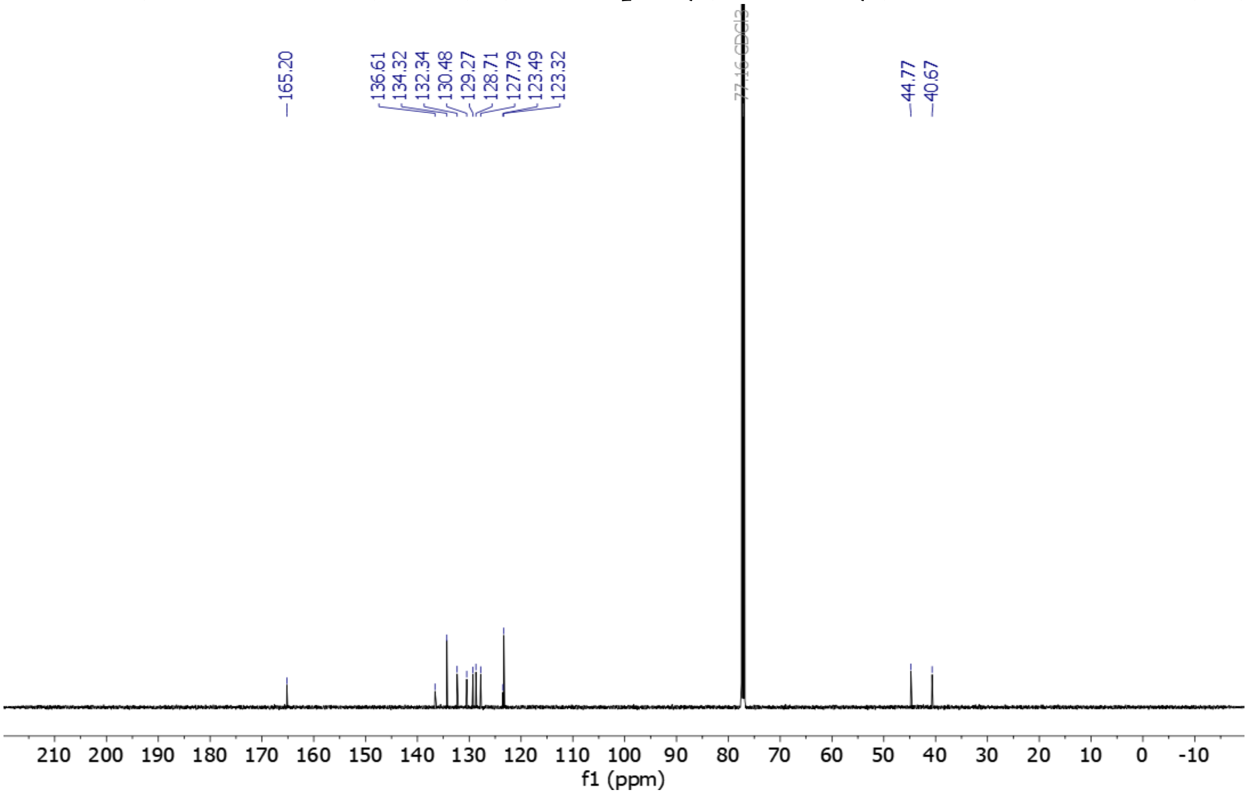

^1H NMR (500 MHz, CD_3CN) of 2-(2-(*m*-tolyl)aziridin-1-yl)isoindoline-1,3-dione (2p)

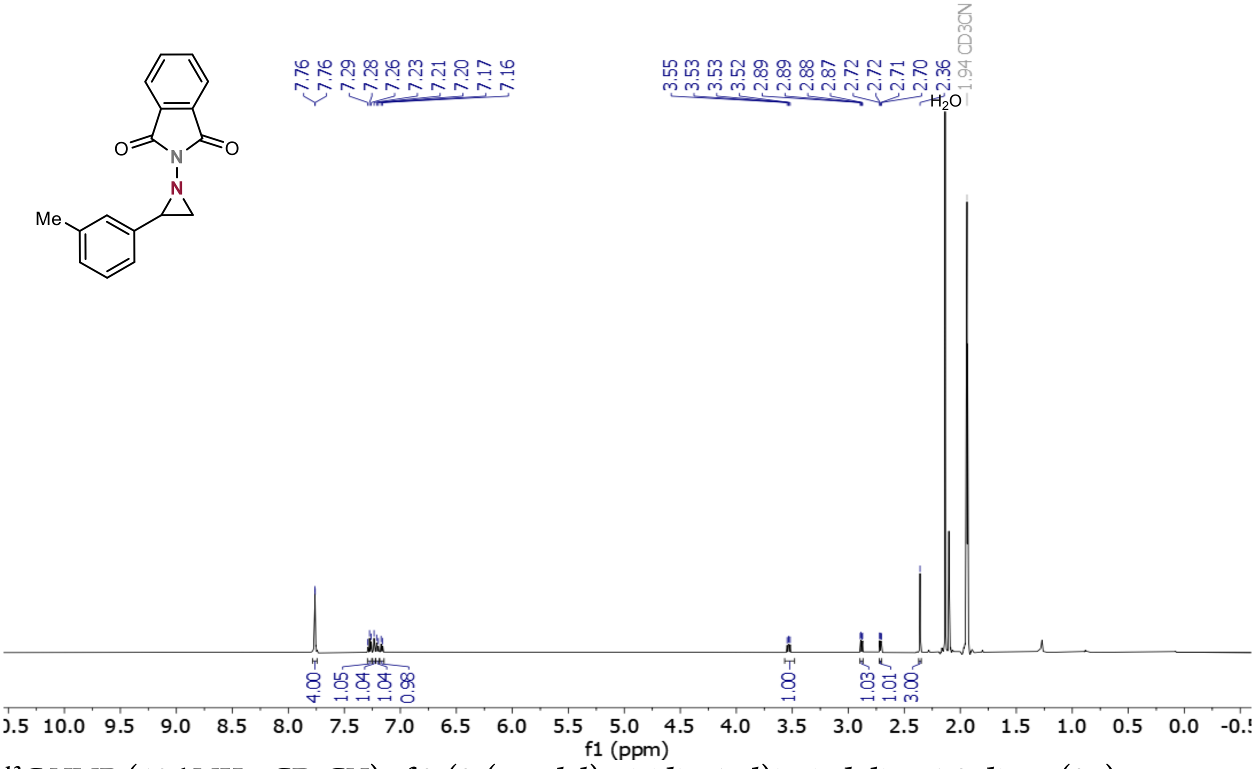

^{13}C NMR (126 MHz, CD_3CN) of 2-(2-(*m*-tolyl)aziridin-1-yl)isoindoline-1,3-dione (2p)

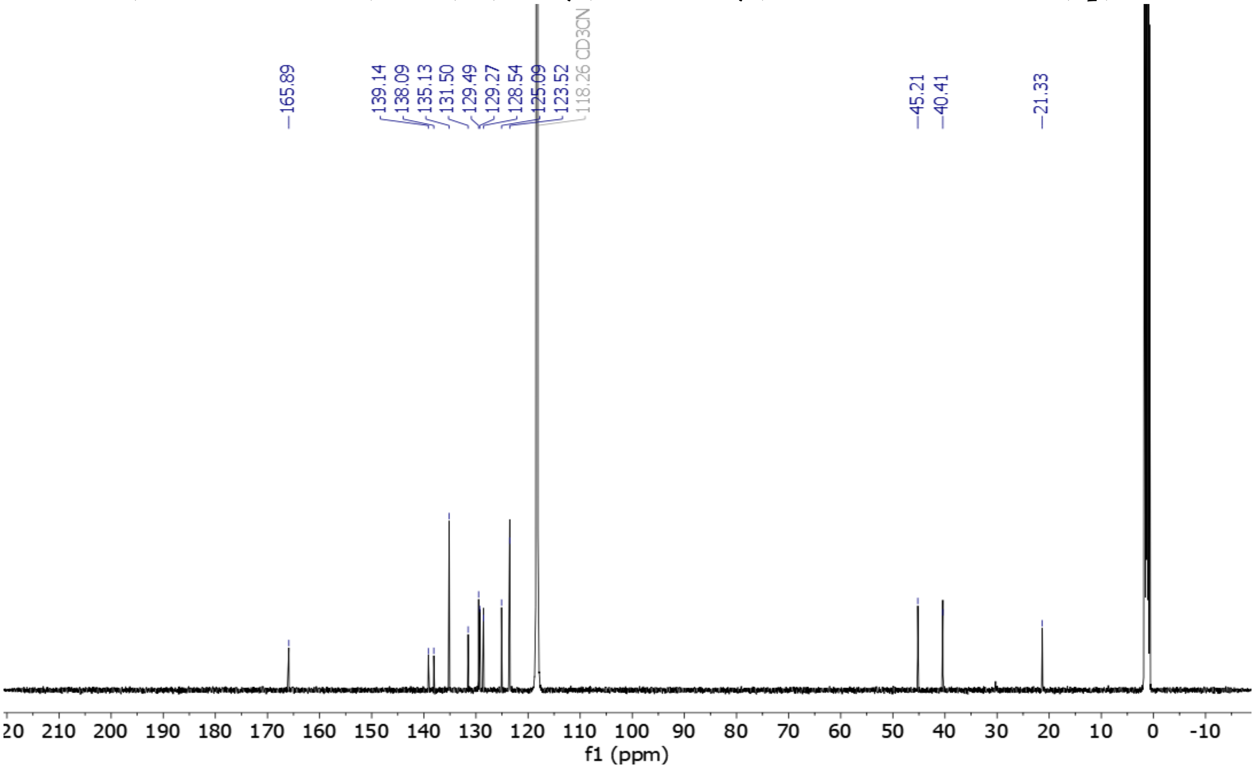

^{1}H NMR (400 MHz, $CDCl_3$) of 2-(2-methyl-2-phenylaziridin-1-yl)isoindoline-1,3-dione (2q)

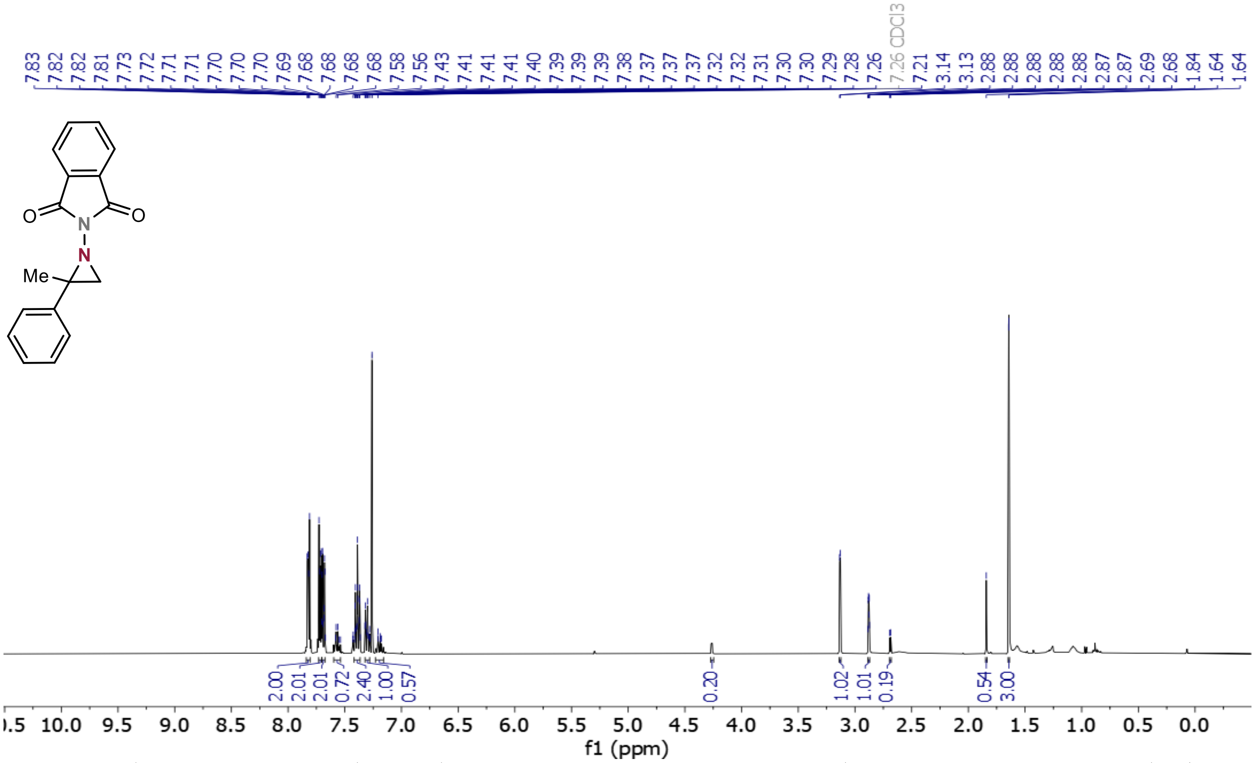

^{13}C NMR (101 MHz, $CDCl_3$) of 2-(2-methyl-2-phenylaziridin-1-yl)isoindoline-1,3-dione (2q)

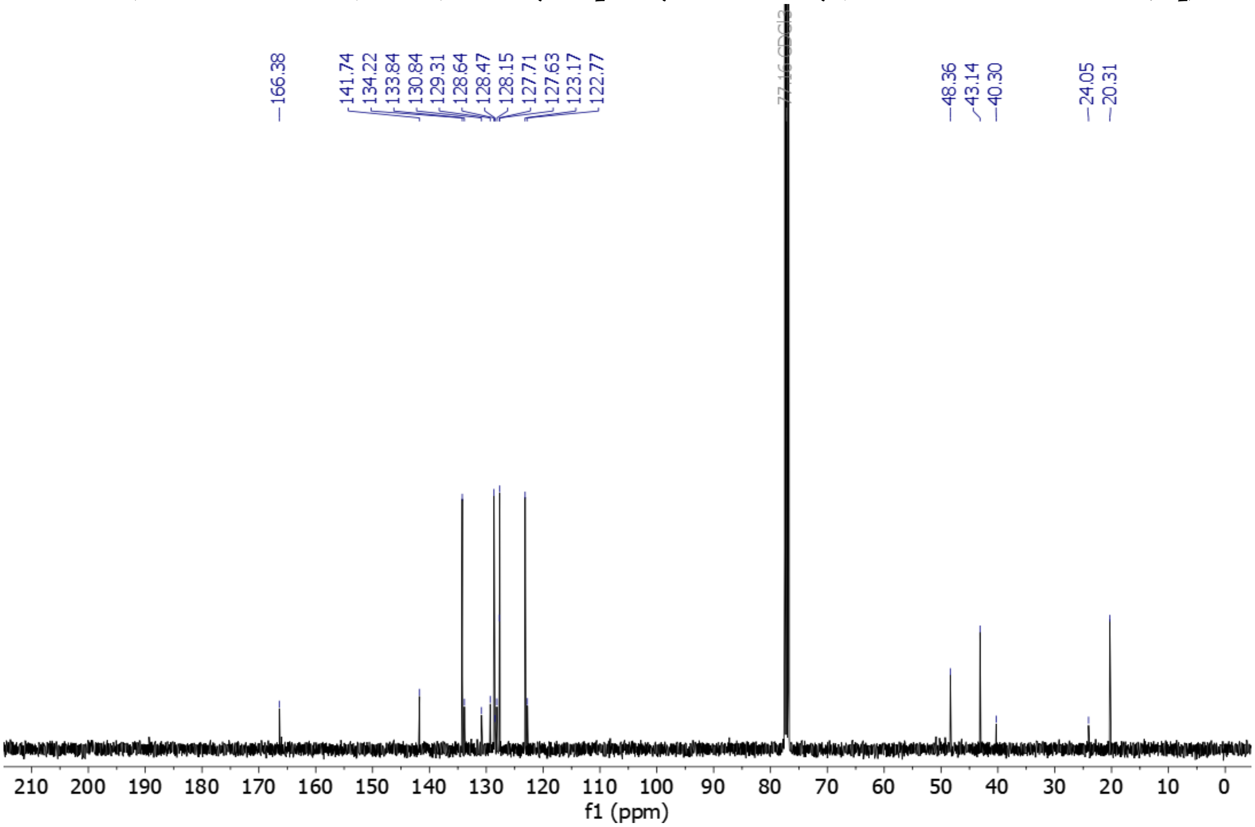

^{1}H NMR (400 MHz, $CDCl_3$) of 2-(2,2-diphenylaziridin-1-yl)isoindoline-1,3-dione (2r)

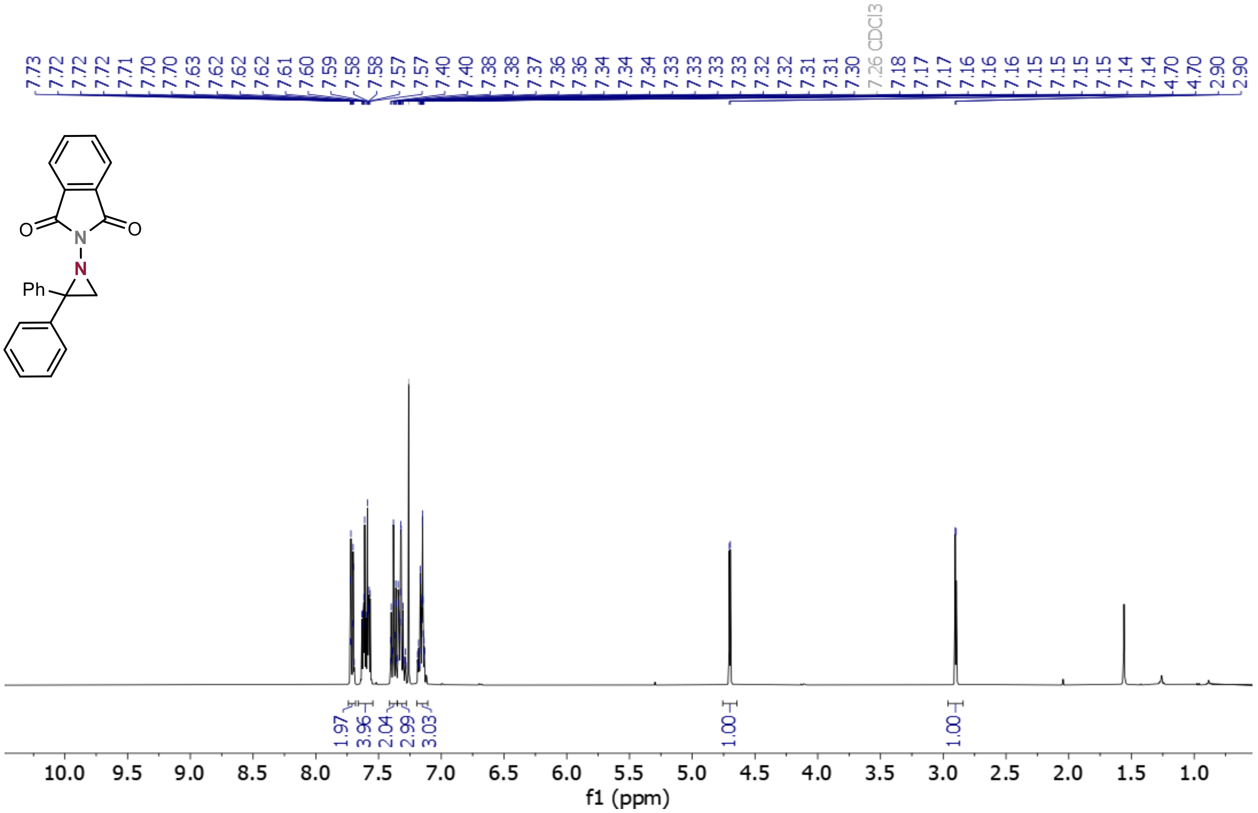

^{13}C NMR (101 MHz, $CDCl_3$) of 2-(2,2-diphenylaziridin-1-yl)isoindoline-1,3-dione (2r)

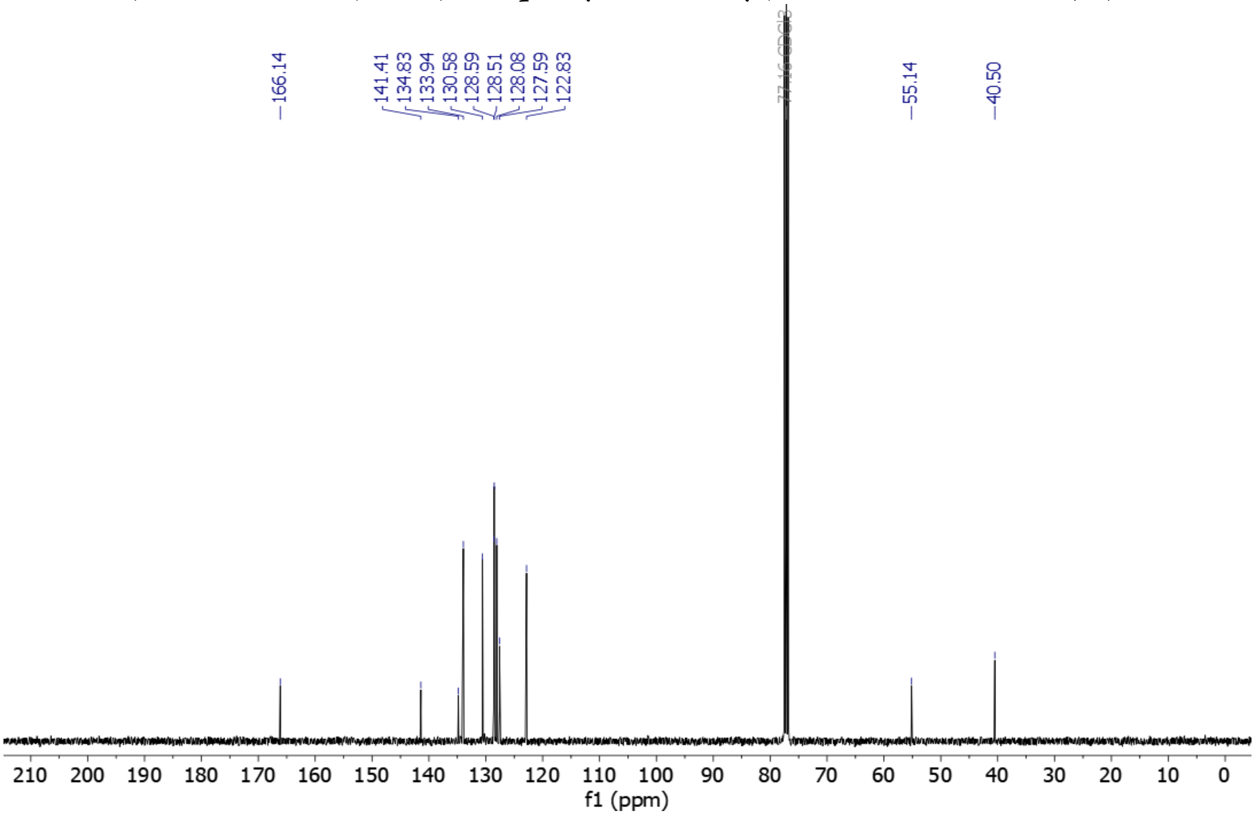

^{1}H NMR (500 MHz, $CDCl_3$) of 2-(2-phenyl-2-(trifluoromethyl)aziridin-1-yl)isoindoline-1,3-dione (2s)

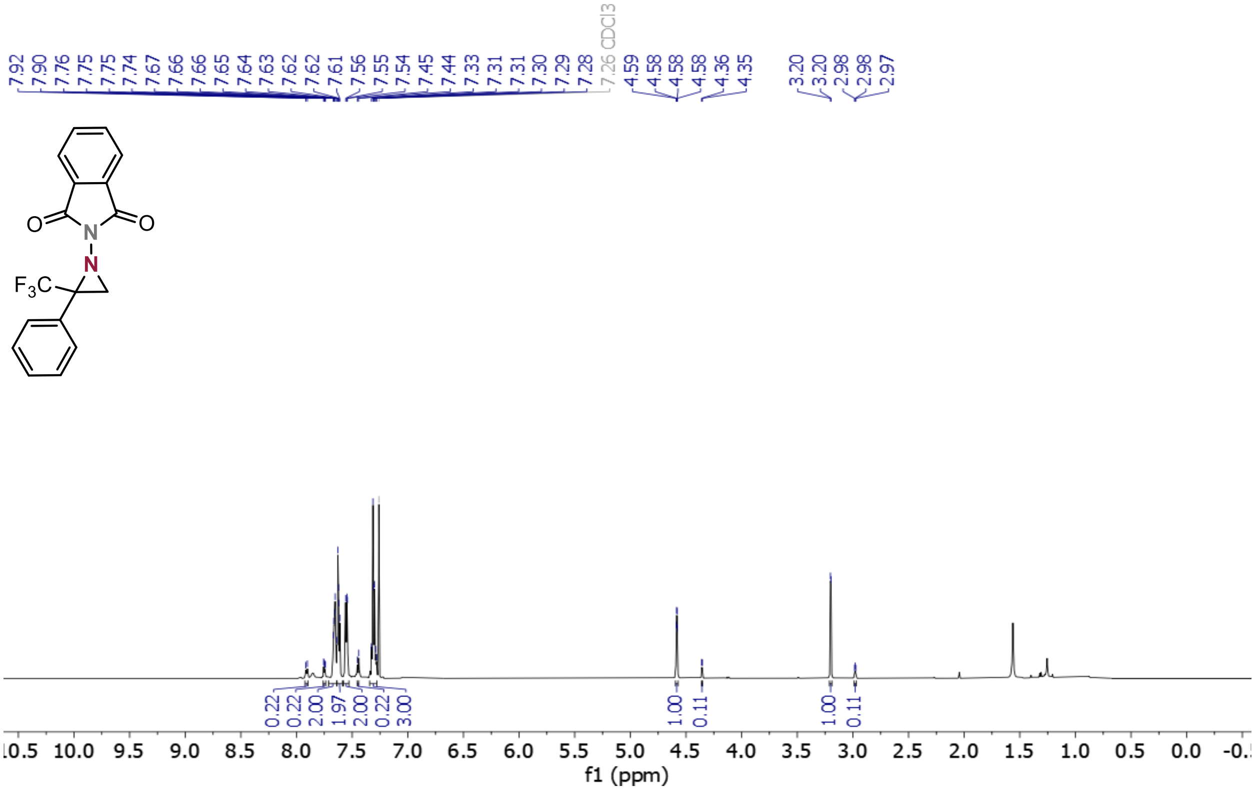

^{13}C NMR (101 MHz, $CDCl_3$) of 2-(2-phenyl-2-(trifluoromethyl)aziridin-1-yl)isoindoline-1,3-dione (2s)

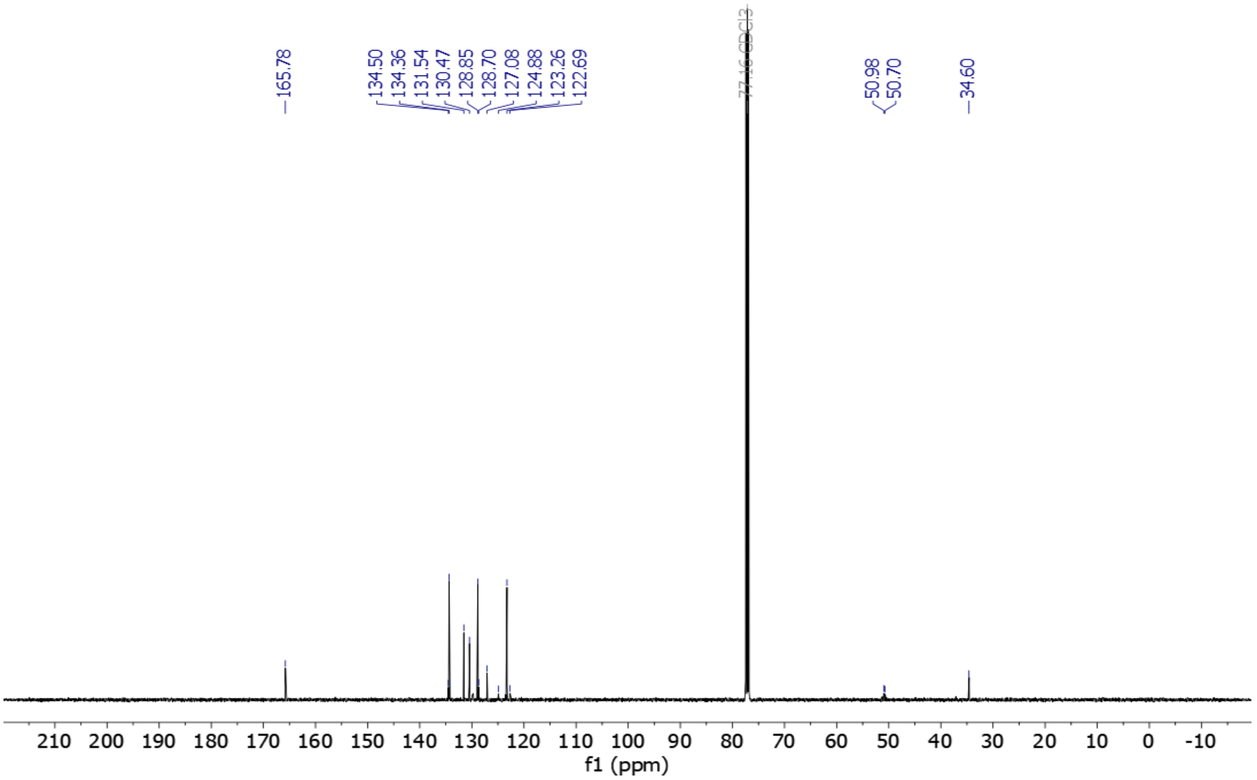

^{19}F NMR (471 MHz, $CDCl_3$) of 2-(2-phenyl-2-(trifluoromethyl)aziridin-1-yl)isoindoline-1,3-dione (2s)

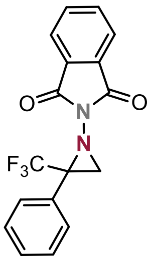

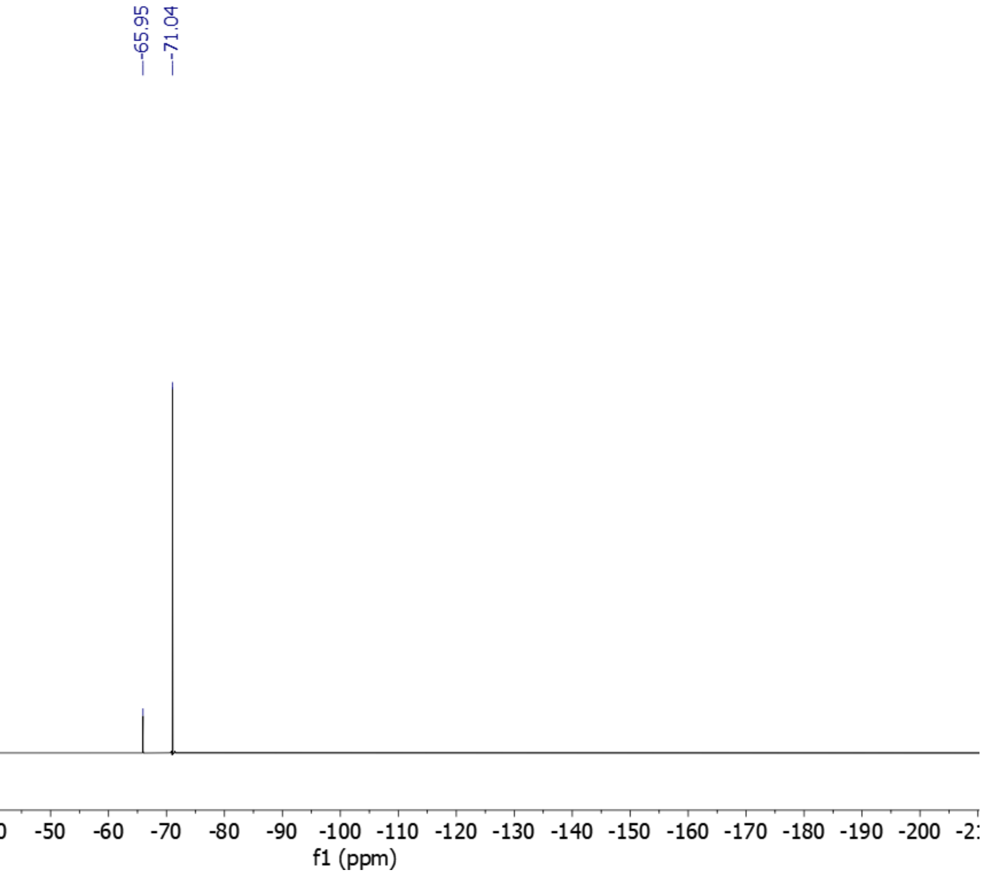

^{1}H NMR (400 MHz, $CDCl_3$) of 2-(2',3'-dihydrospiro[aziridine-2,1'-inden]-1-yl)isoindoline-1,3-dione (2t)

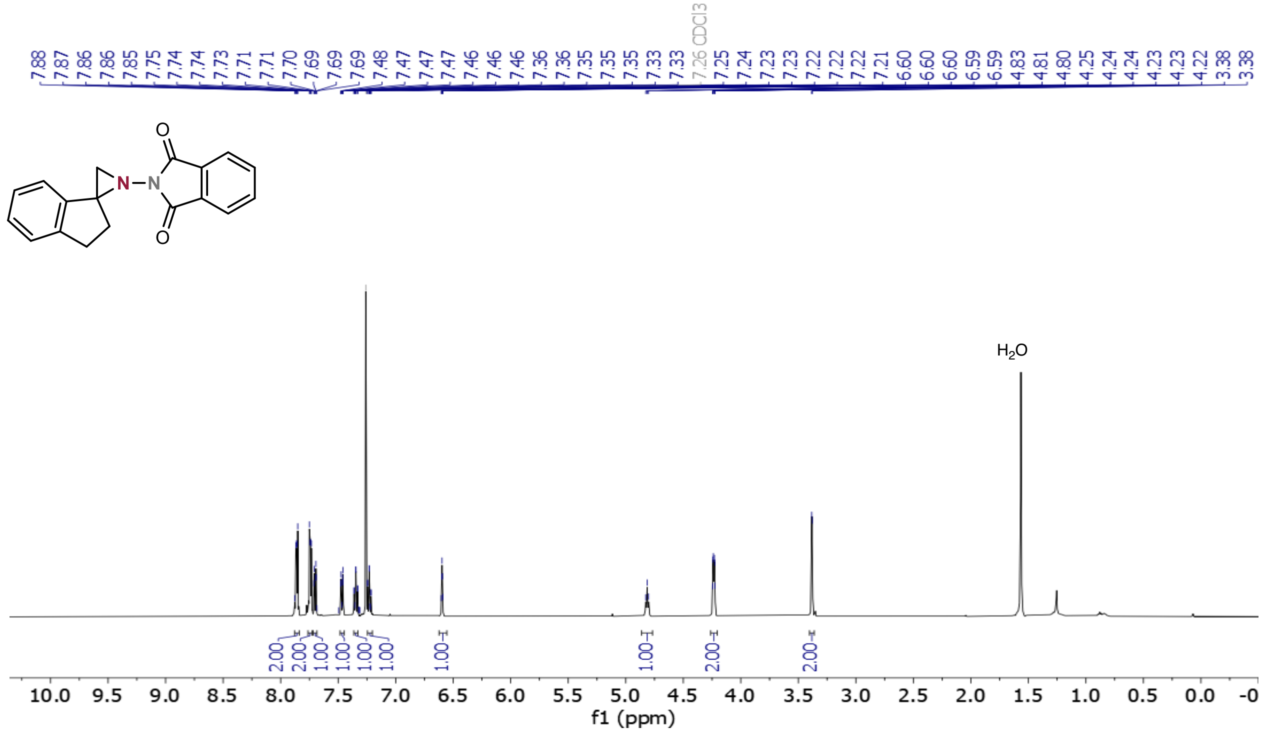

^{13}C NMR (101 MHz, $CDCl_3$) of of 2-(2',3'-dihydrospiro[aziridine-2,1'-inden]-1-yl)isoindoline-1,3-dione (2t)

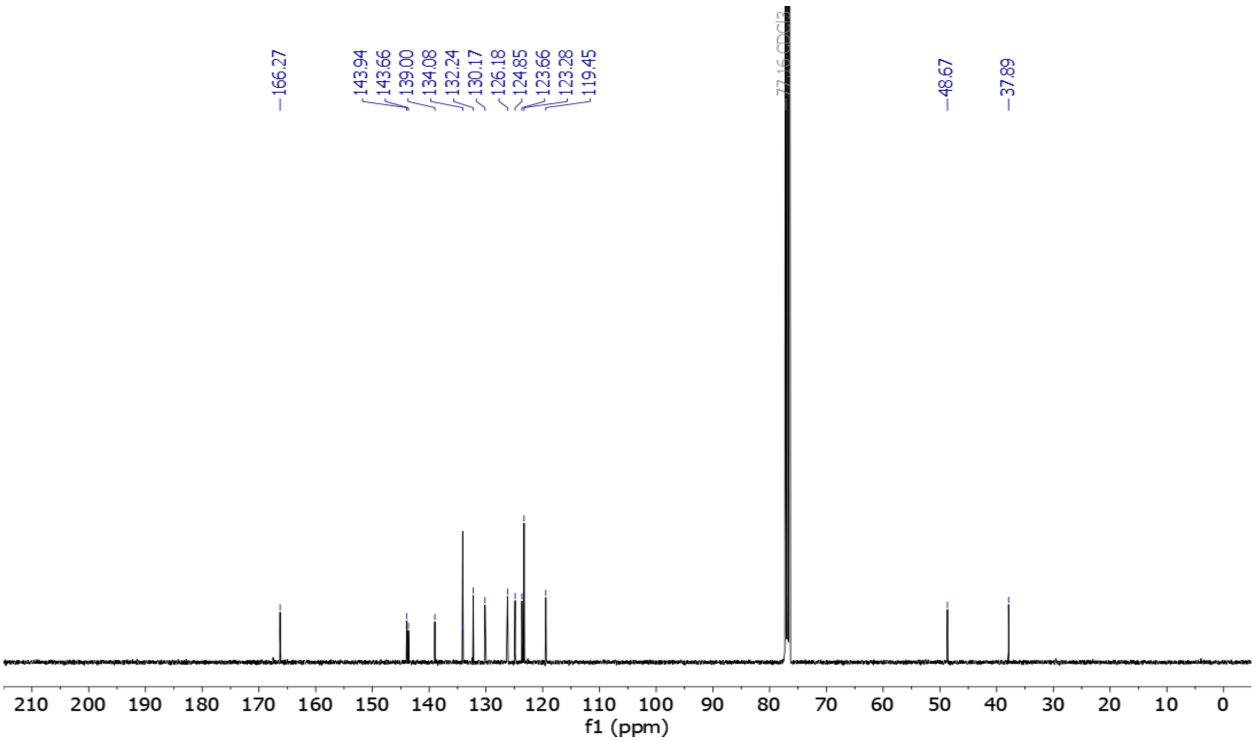

^{1}H NMR (400 MHz, $CDCl_3$) of 2-(6,6a-dihydroindeno[1,2-*b*]azirin-1(1a*H*)-yl)isoindoline-1,3-dione (2u)

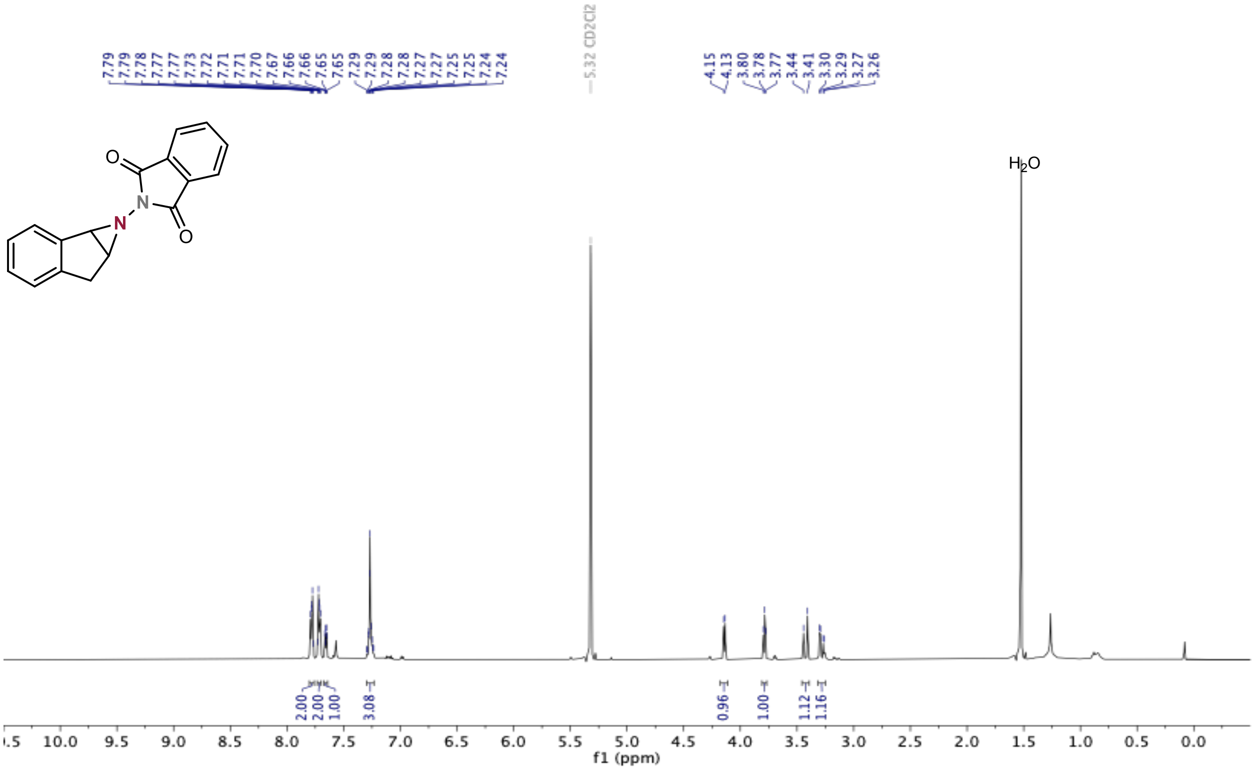

^{13}C NMR (101 MHz, $CDCl_3$) of 2-(6,6a-dihydroindeno[1,2-*b*]azirin-1(1a*H*)-yl)isoindoline-1,3-dione (2u)

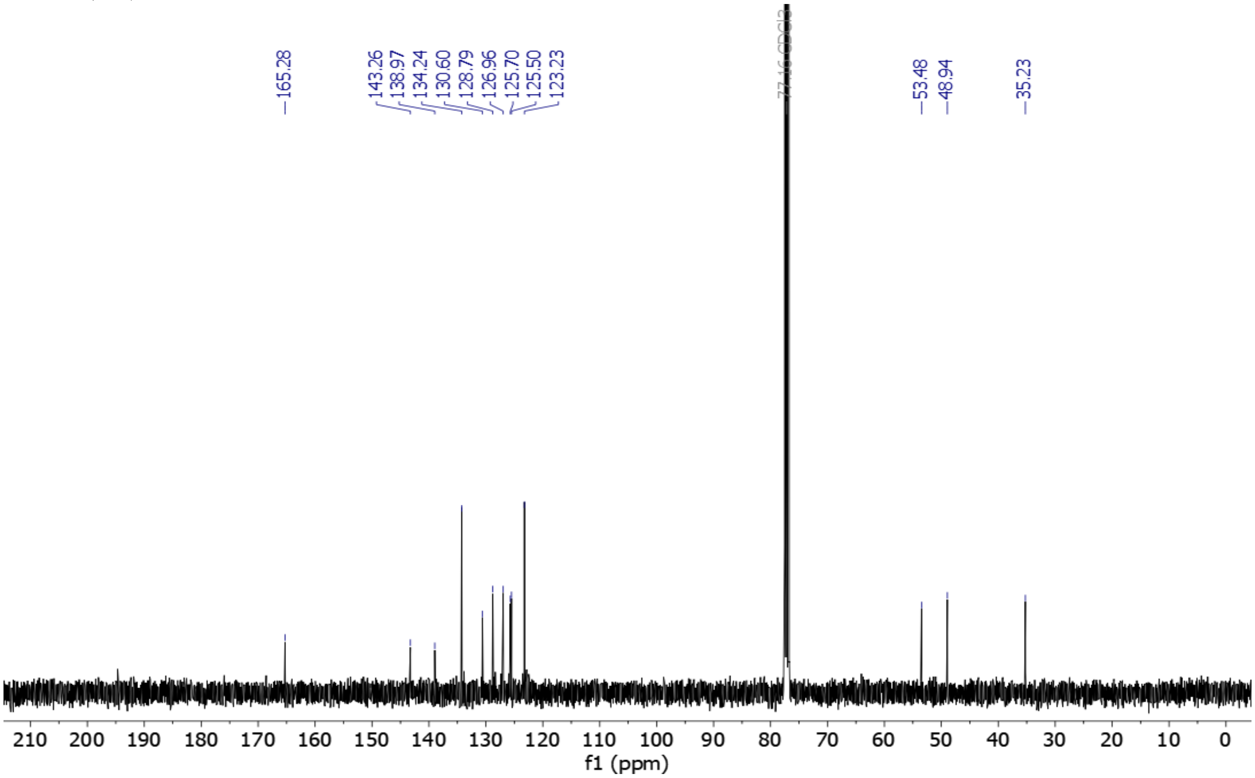

^{1}H NMR (400 MHz, CD_2Cl_2) of 2-(1a,2,3,7b-tetrahydro-1*H*-naphtho[1,2-*b*]azirin-1-yl)isoindoline-1,3-dione (2v)

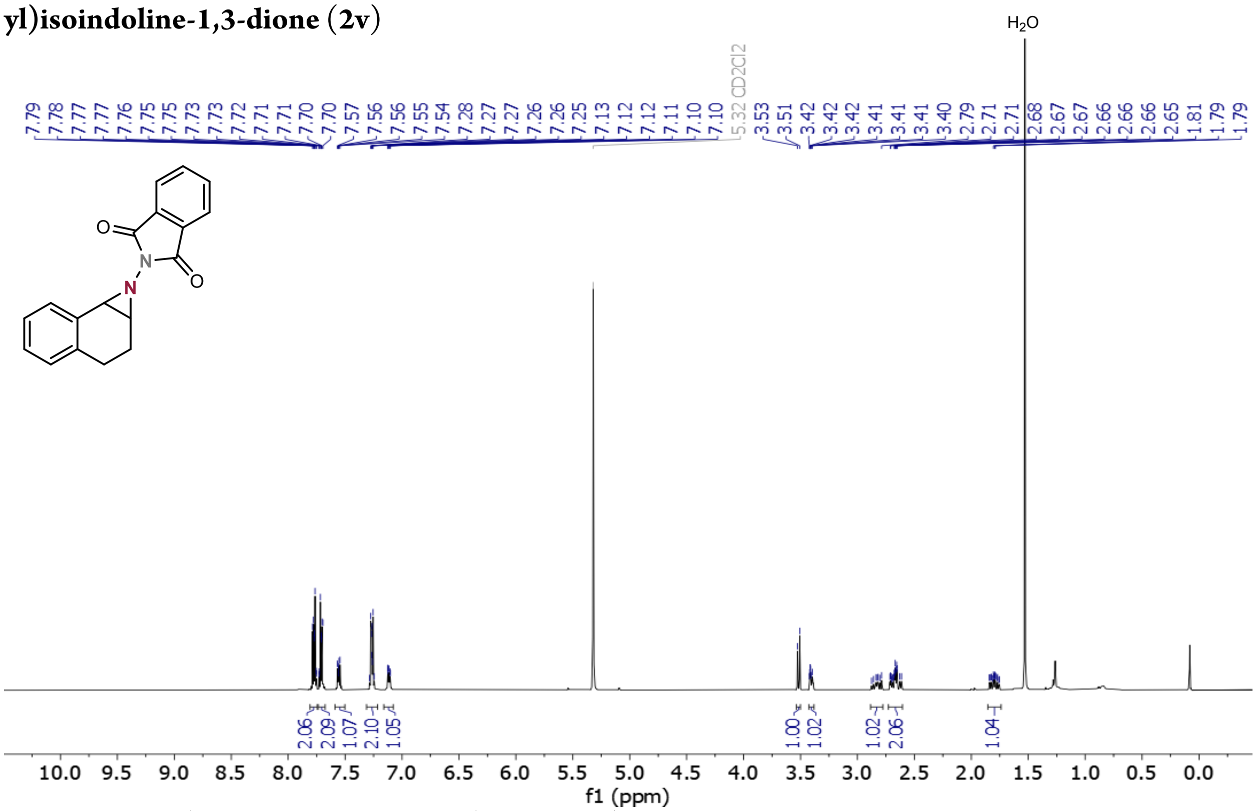

^{13}C NMR (101 MHz, CD_2Cl_2) of 2-(1a,2,3,7b-tetrahydro-1*H*-naphtho[1,2-*b*]azirin-1-yl)isoindoline-1,3-dione (2v)

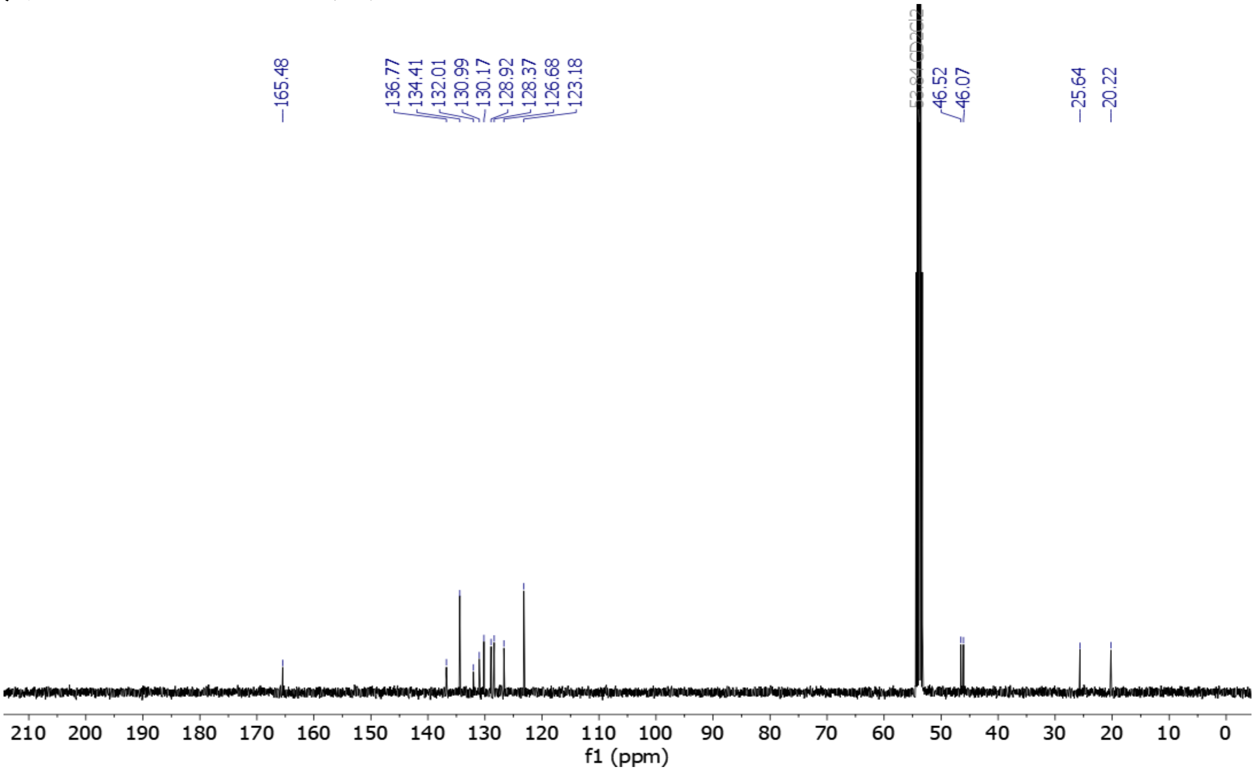

^{1}H NMR (400 MHz, CDCl$_3$) of 2-(2-benzoyl-3-phenylaziridin-1-yl)isoindoline-1,3-dione (2w)

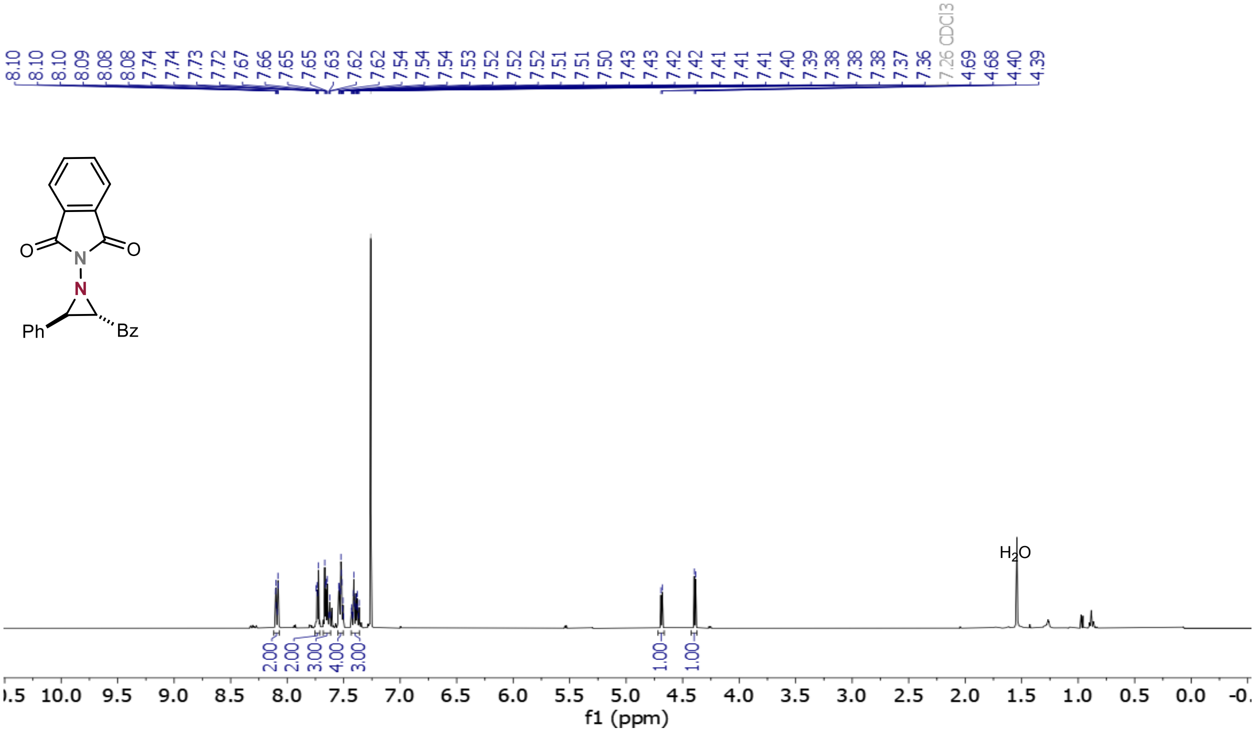

^{13}C NMR (101 MHz, CDCl$_3$) of 2-(2-benzoyl-3-phenylaziridin-1-yl)isoindoline-1,3-dione (2w)

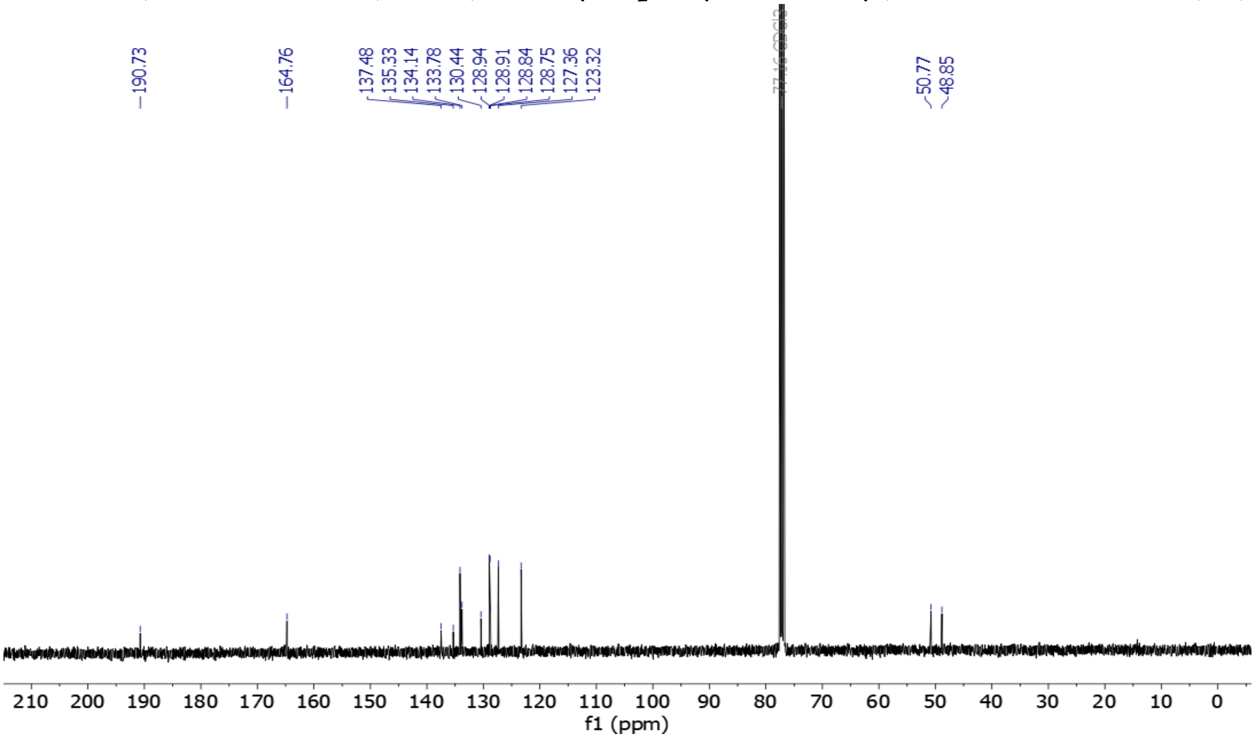

^{1}H NMR (400 MHz, $CDCl_3$) of ethyl 1-(1,3-dioxoisoindolin-2-yl)-3-phenylaziridine-2-carboxylate (2x)

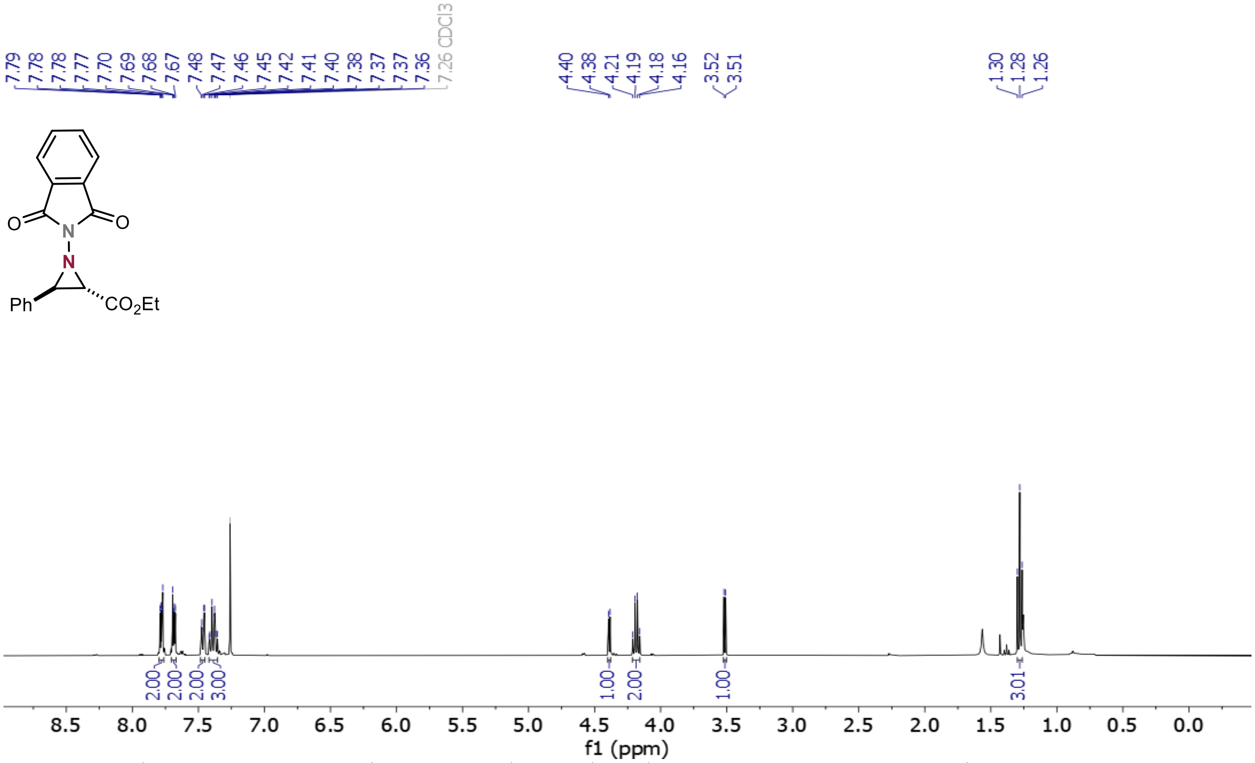

^{13}C NMR (101 MHz, $CDCl_3$) of ethyl (2*S*,3*R*)-1-(1,3-dioxoisoindolin-2-yl)-3-phenylaziridine-2-carboxylate (2x)

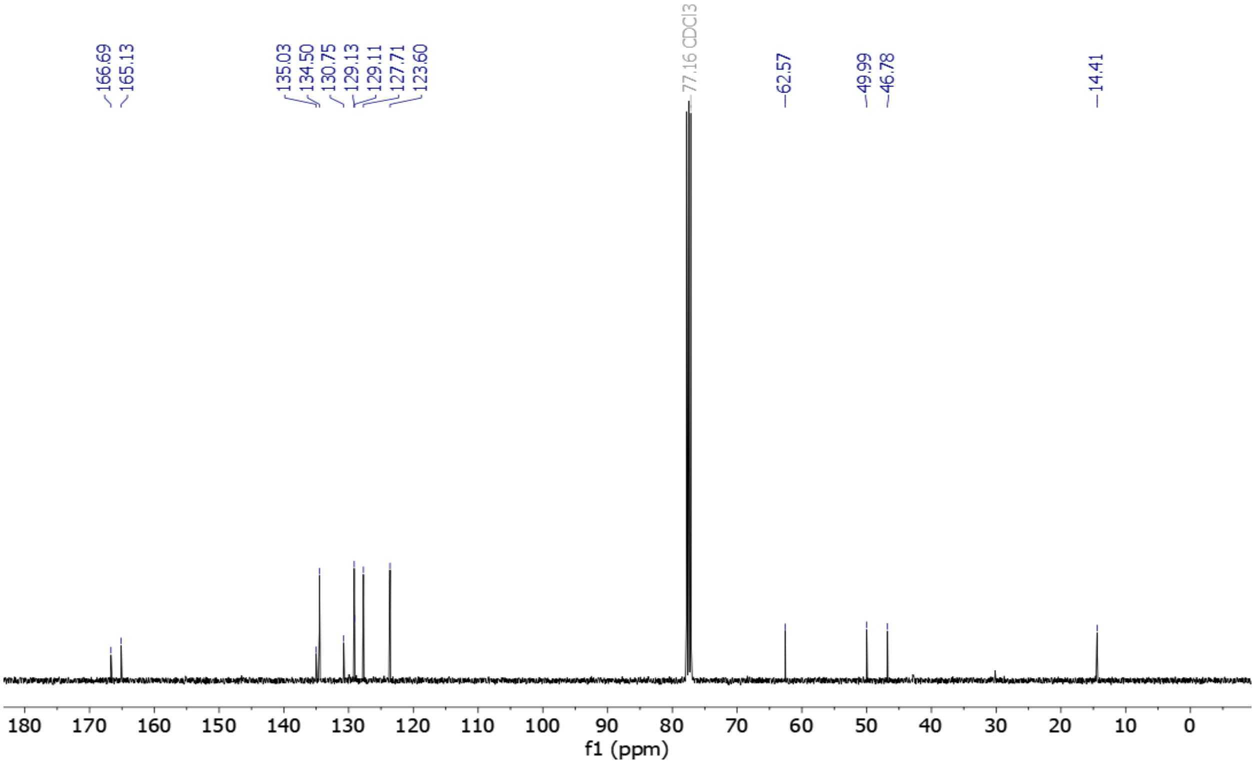

^{1}H NMR (400 MHz, $CDCl_3$) of 2-(1-phenyl-7-azabicyclo[4.1.0]heptan-7-yl)isoindoline-1,3-dione (2aa)

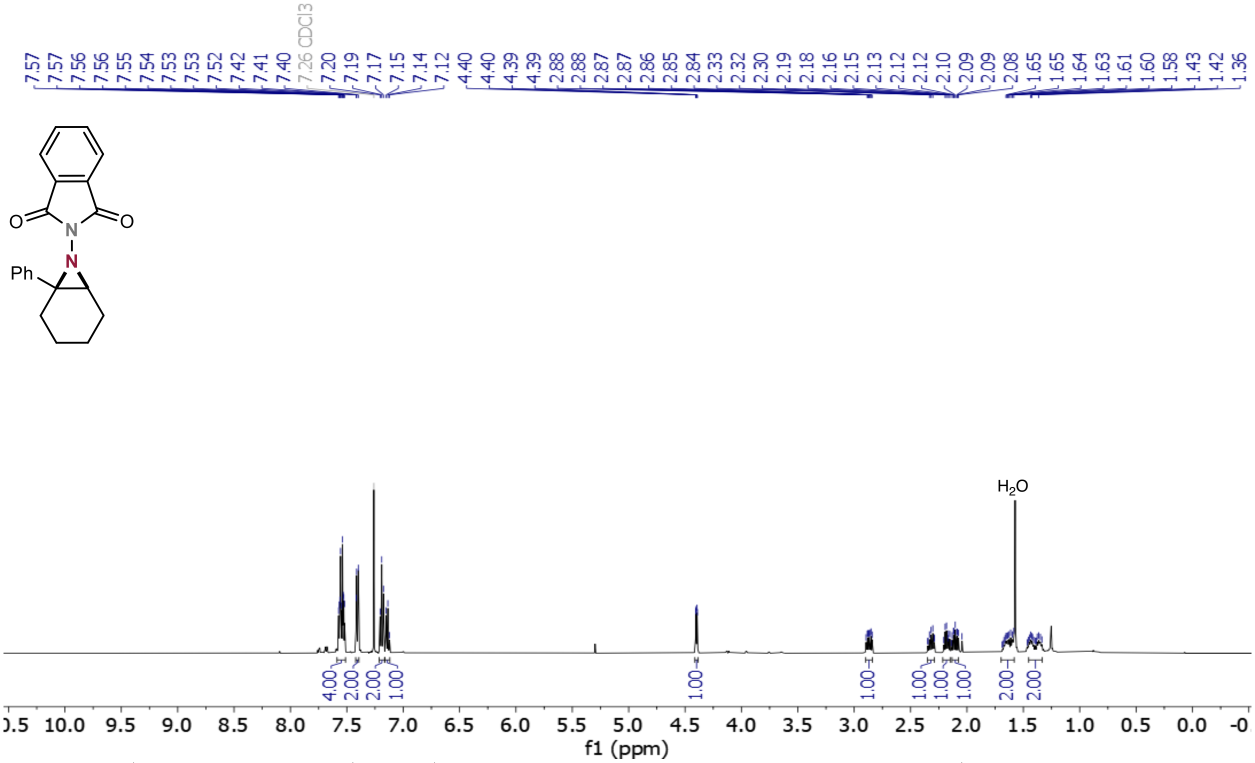

^{13}C NMR (101 MHz, $CDCl_3$) of 2-(1-phenyl-7-azabicyclo[4.1.0]heptan-7-yl)isoindoline-1,3-dione (2aa)

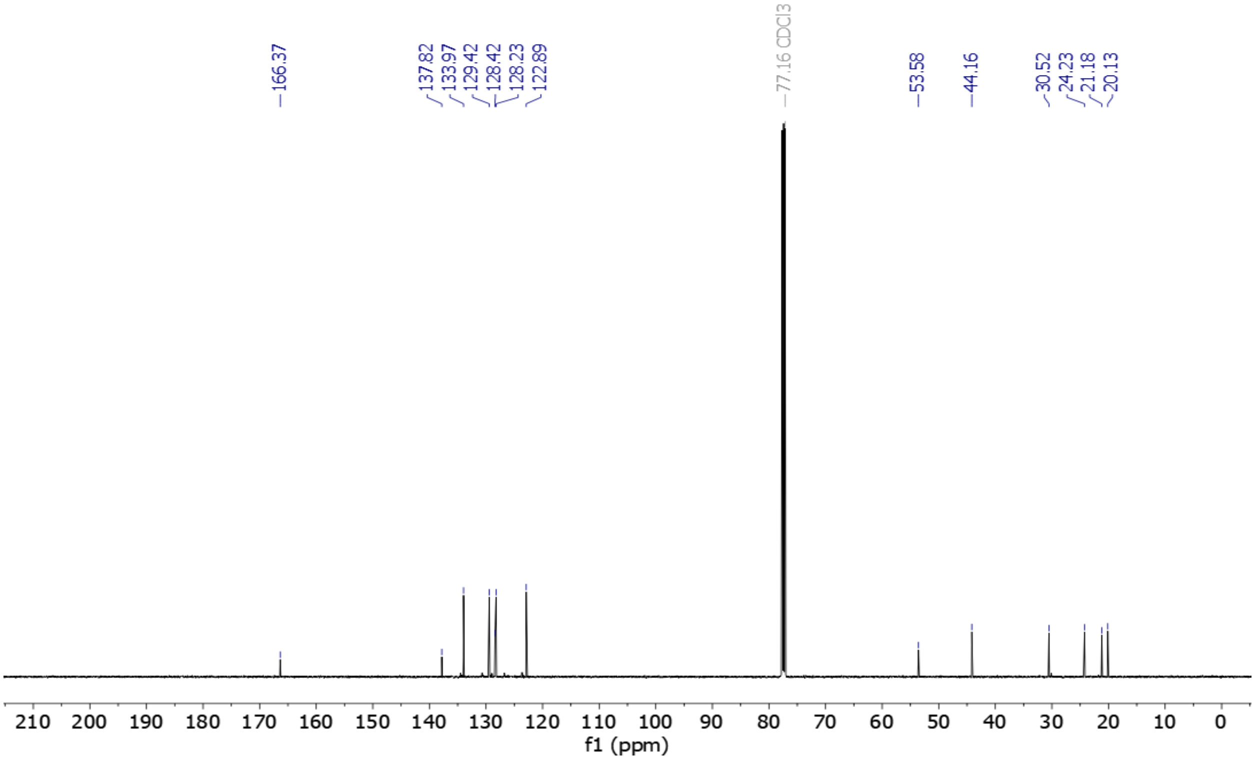

^{1}H NMR (500 MHz, $CDCl_3$) of 2-(2,2,3-trimethyl-3-(4-(trifluoromethyl)phenyl)aziridin-1-yl)isoindoline-1,3-dione (2ab)

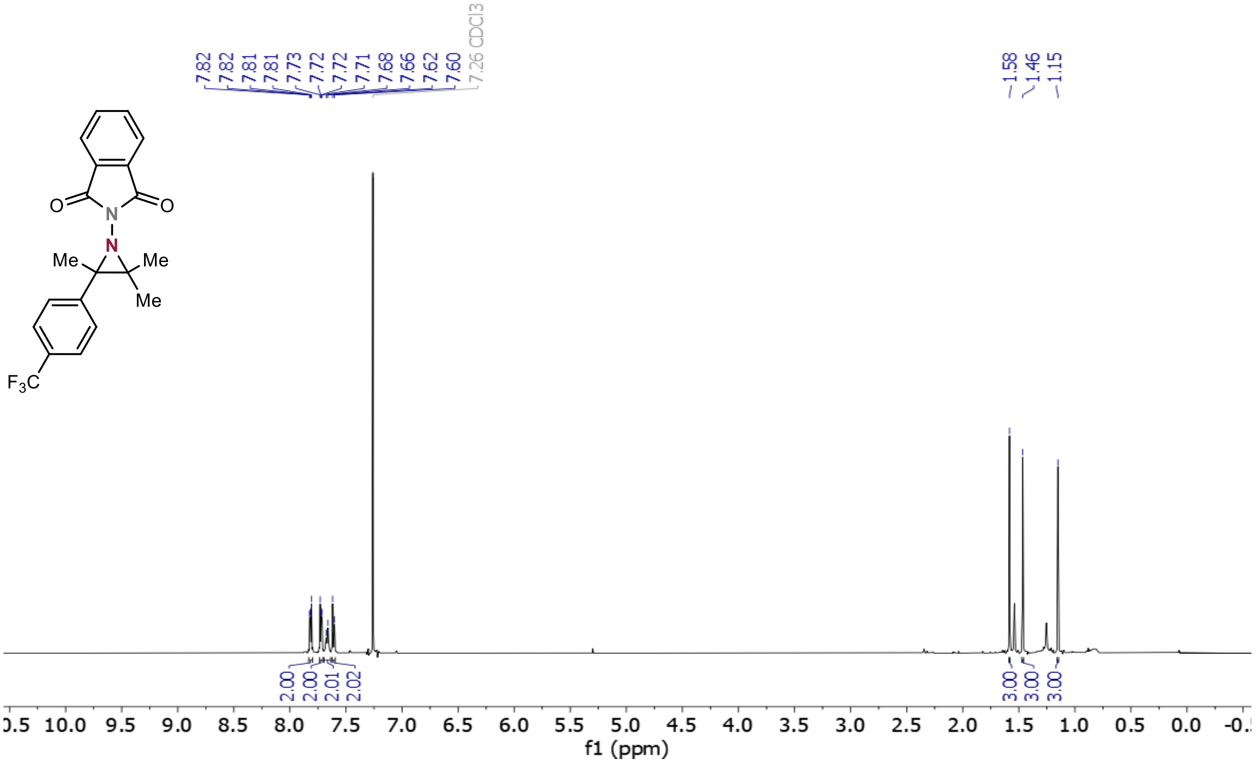

^{13}C NMR (101 MHz, $CDCl_3$) of 2-(2,2,3-trimethyl-3-(4-(trifluoromethyl)phenyl)aziridin-1-yl)isoindoline-1,3-dione (2ab)

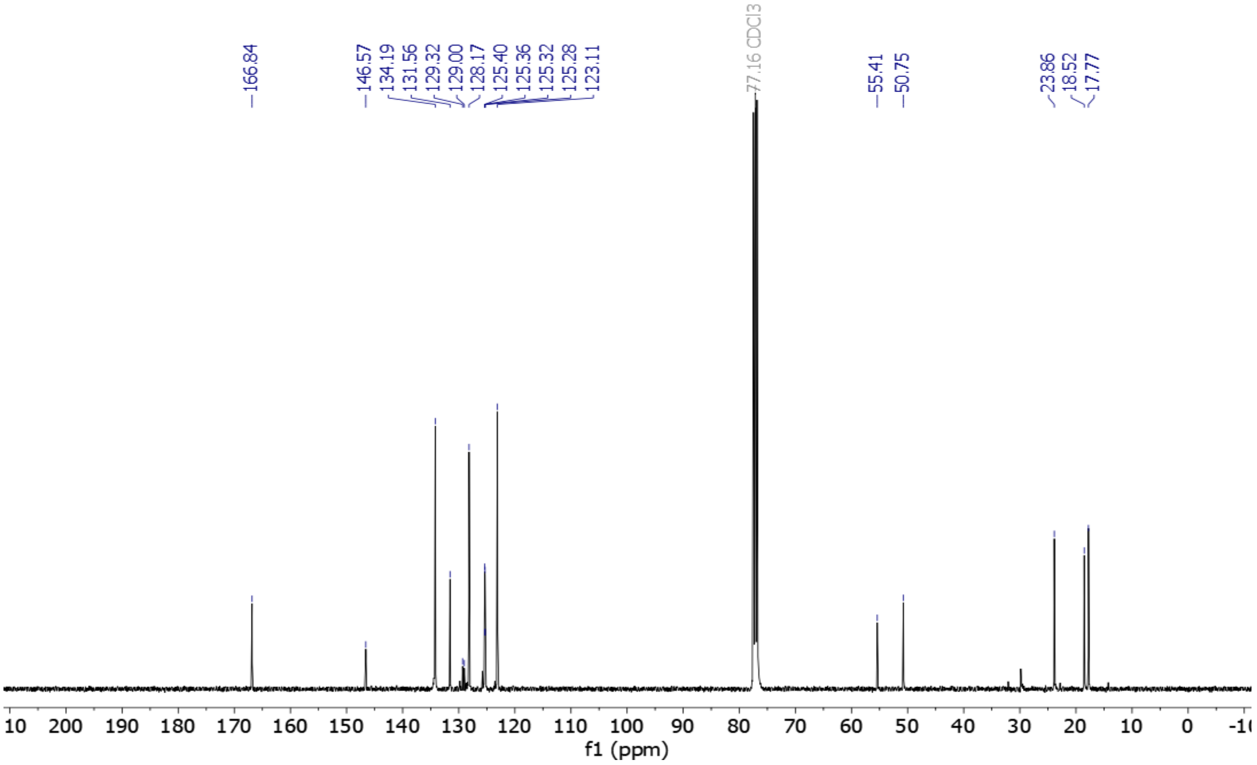

^{19}F NMR (471 MHz, $CDCl_3$) of 2-(2,2,3-trimethyl-3-(4-(trifluoromethyl)phenyl)aziridin-1-yl)isoindoline-1,3-dione (2ab)

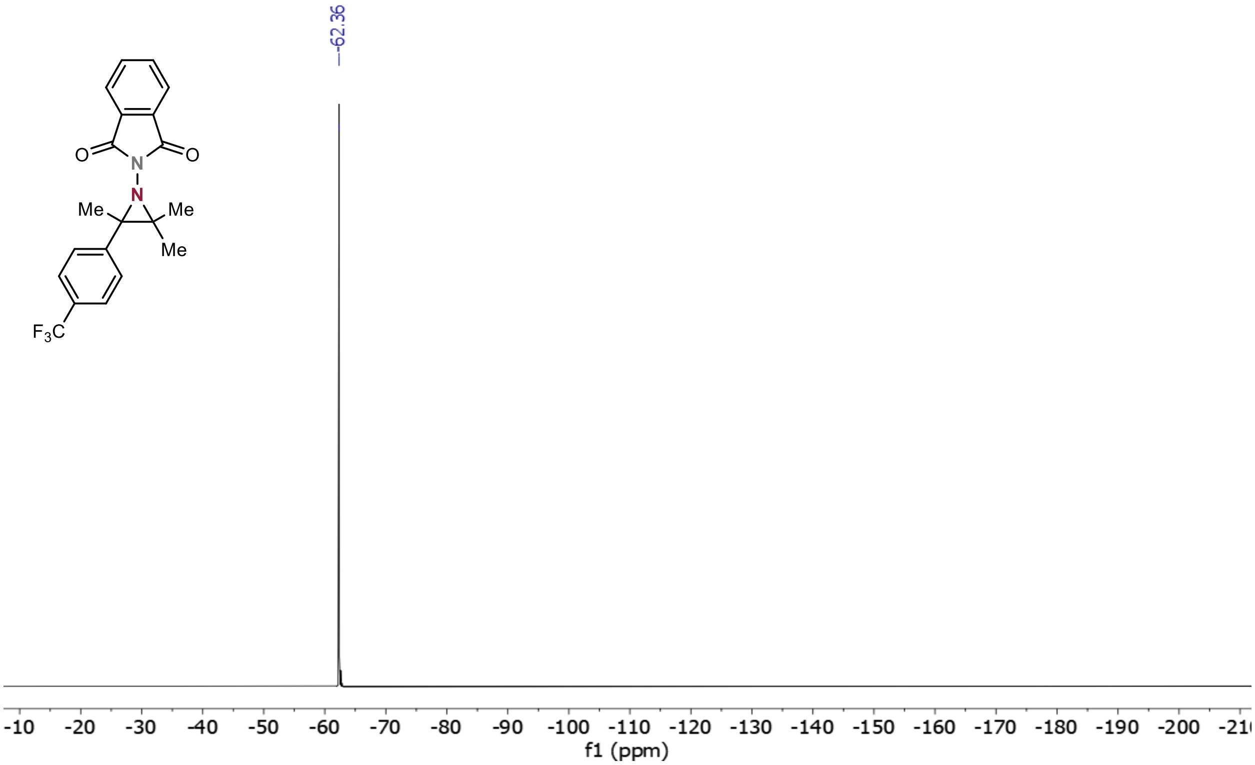

^{1}H NMR (400 MHz, $CDCl_3$) of 2-(2-(bicyclo[4.2.0]octa-1(6),2,4-trien-3-yl)aziridin-1-yl)isoindoline-1,3-dione (2ac)

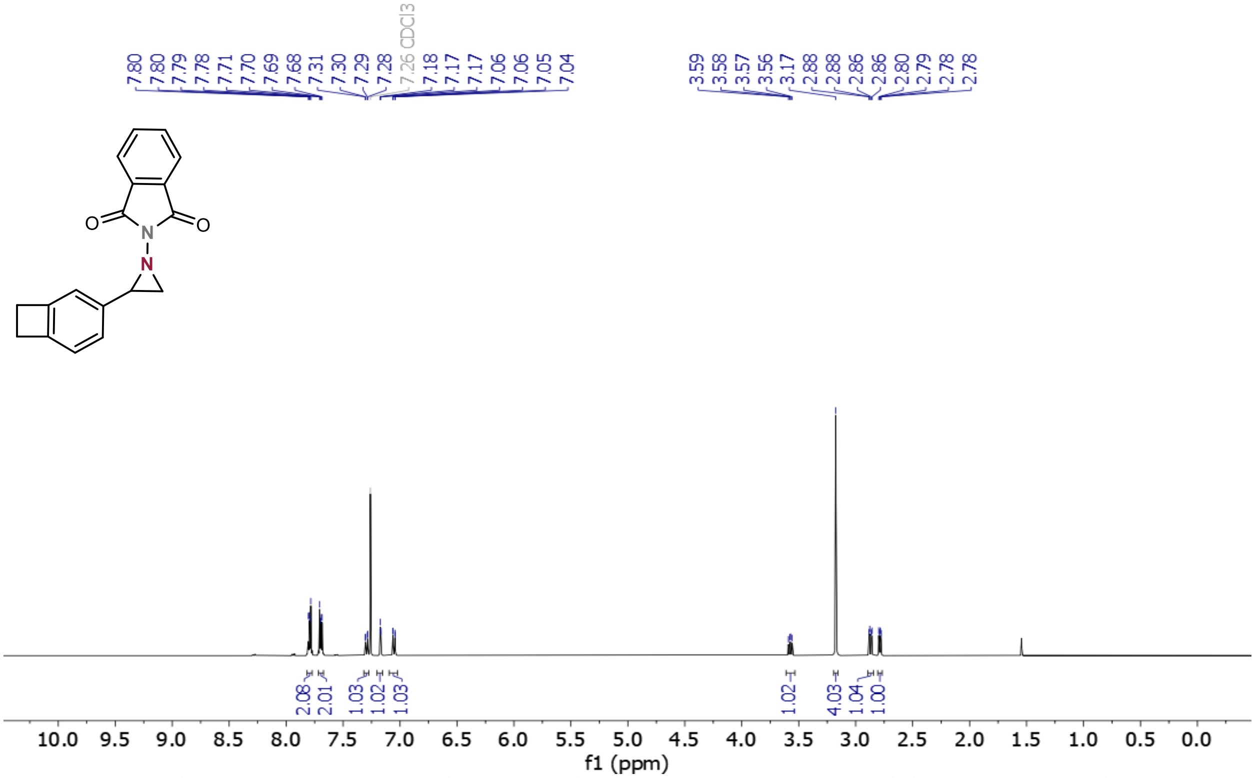

^{13}C NMR (101 MHz, $CDCl_3$) of 2-(2-(bicyclo[4.2.0]octa-1(6),2,4-trien-3-yl)aziridin-1-yl)isoindoline-1,3-dione (2ac)

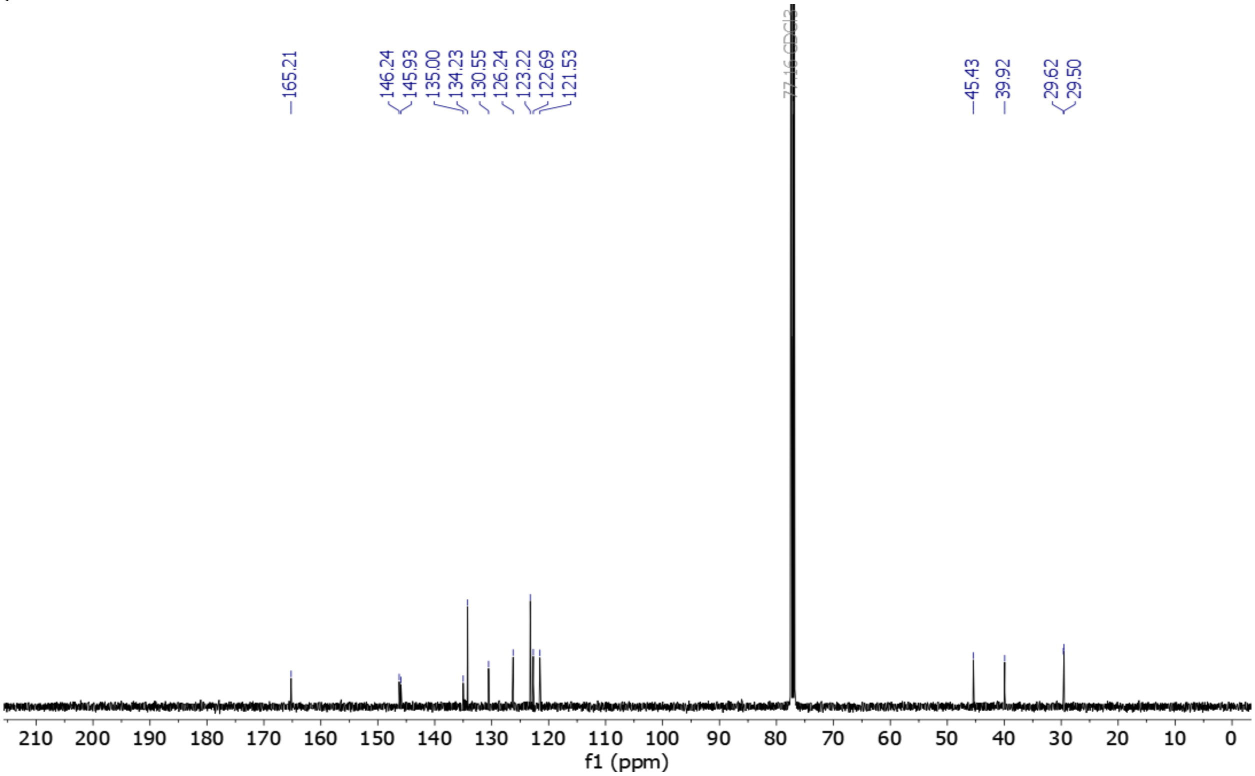

^{1}H NMR (400 MHz, $CDCl_3$) of 2-(2-([1,1'-biphenyl]-4-yl)aziridin-1-yl)isoindoline-1,3-dione (2ad)

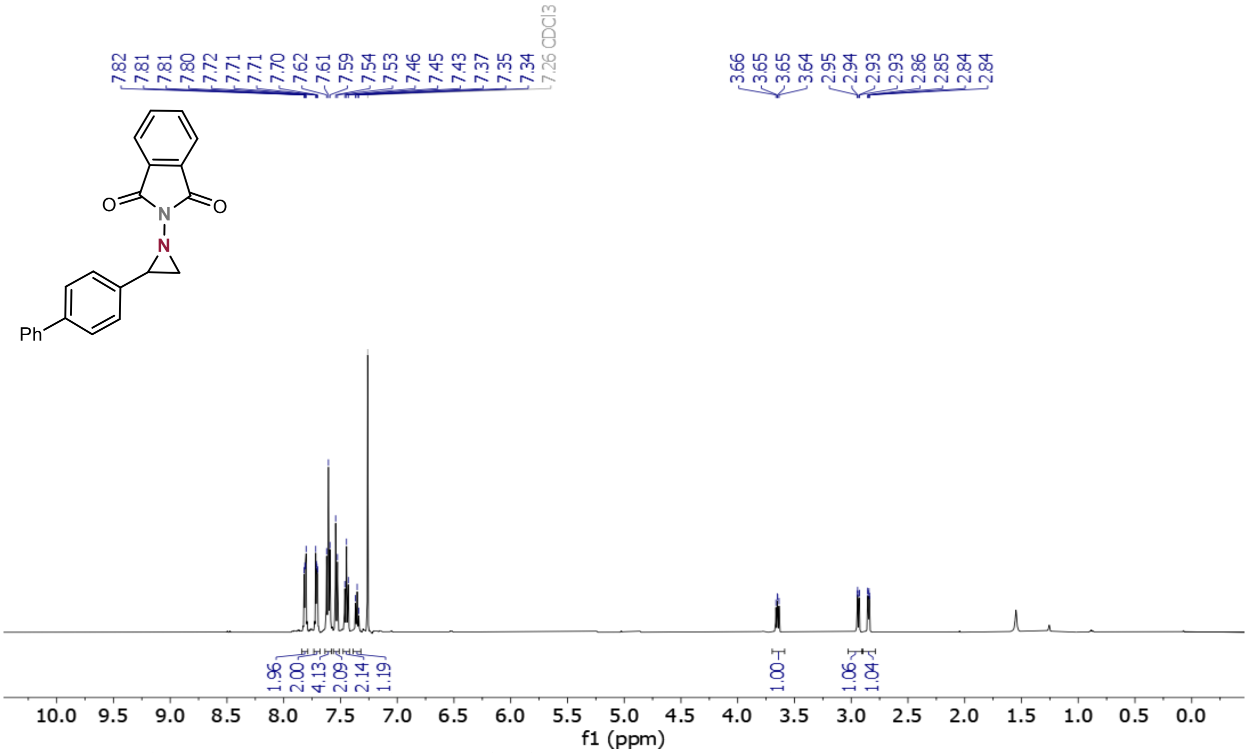

^{13}C NMR (101 MHz, $CDCl_3$) of 2-(2-([1,1'-biphenyl]-4-yl)aziridin-1-yl)isoindoline-1,3-dione (2ad)

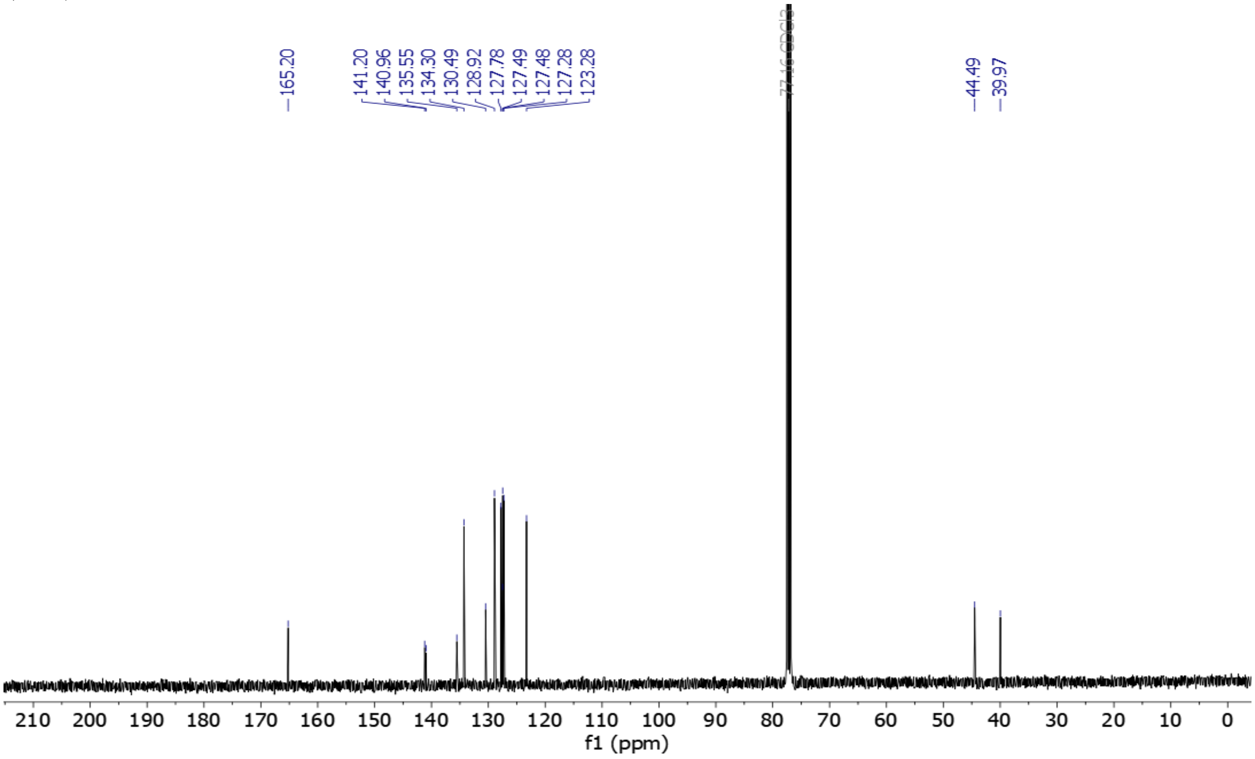

^{1}H NMR (400 MHz, CD_3CN) of 8-(4-(1-(1,3-dioxoisoindolin-2-yl)aziridin-2-yl)phenyl)-4-methyldihydro-4λ^{4},8λ^{4}-[1,3,2]oxazaborolo[2,3-*b*][1,3,2]oxazaborole-2,6(3*H*,5*H*)-dione (2ae)

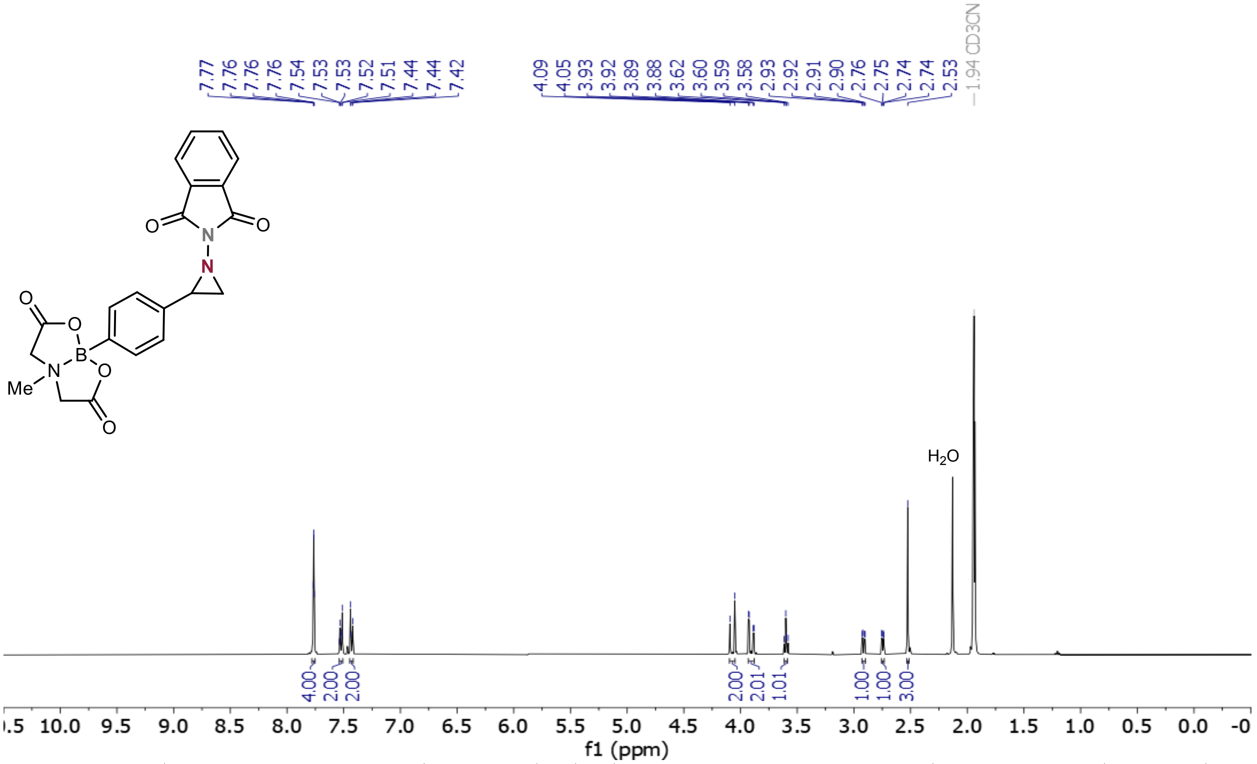

^{13}C NMR (101 MHz, CD_2Cl_2) of 8-(4-(1-(1,3-dioxoisoindolin-2-yl)aziridin-2-yl)phenyl)-4-methyldihydro-4λ^{4},8λ^{4}-[1,3,2]oxazaborolo[2,3-*b*][1,3,2]oxazaborole-2,6(3*H*,5*H*)-dione (2ae)

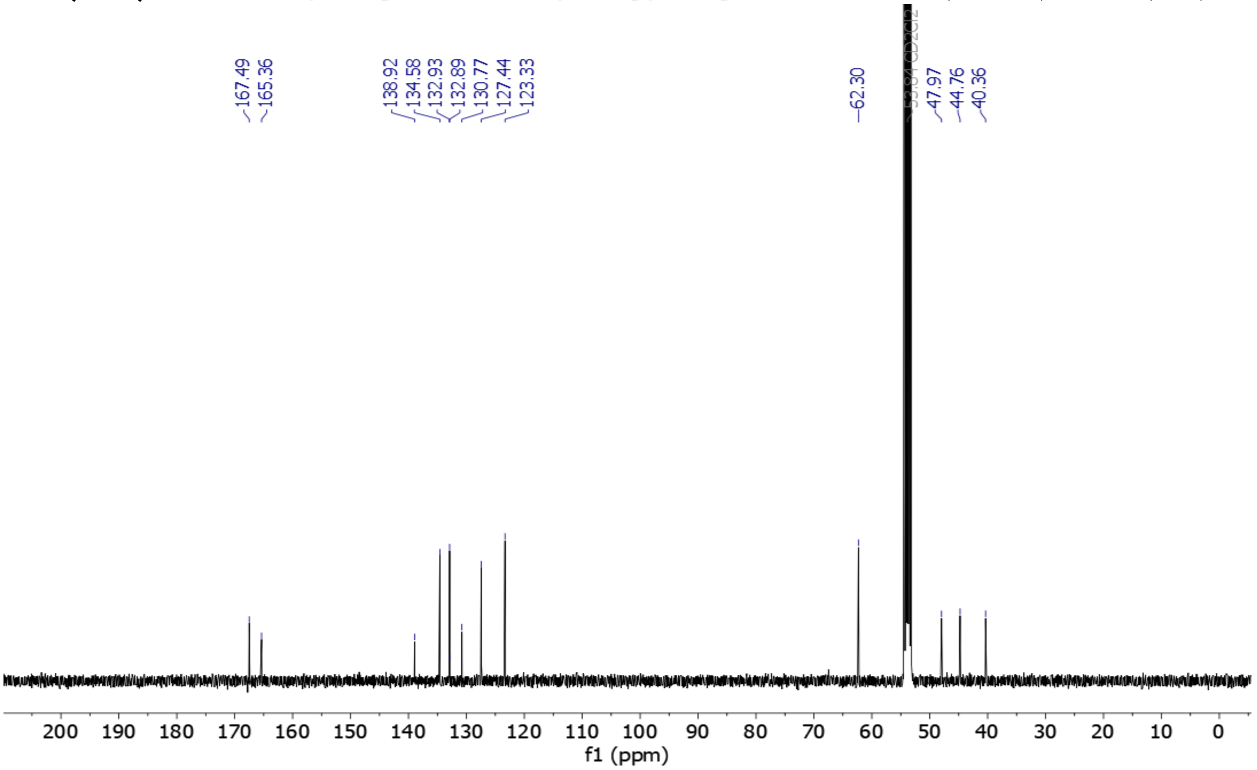

^{1}H NMR (500 MHz, CD_2Cl_2) of 2-(2-(2-bromo-4-fluorophenyl)aziridin-1-yl)isoindoline-1,3-dione (2af)

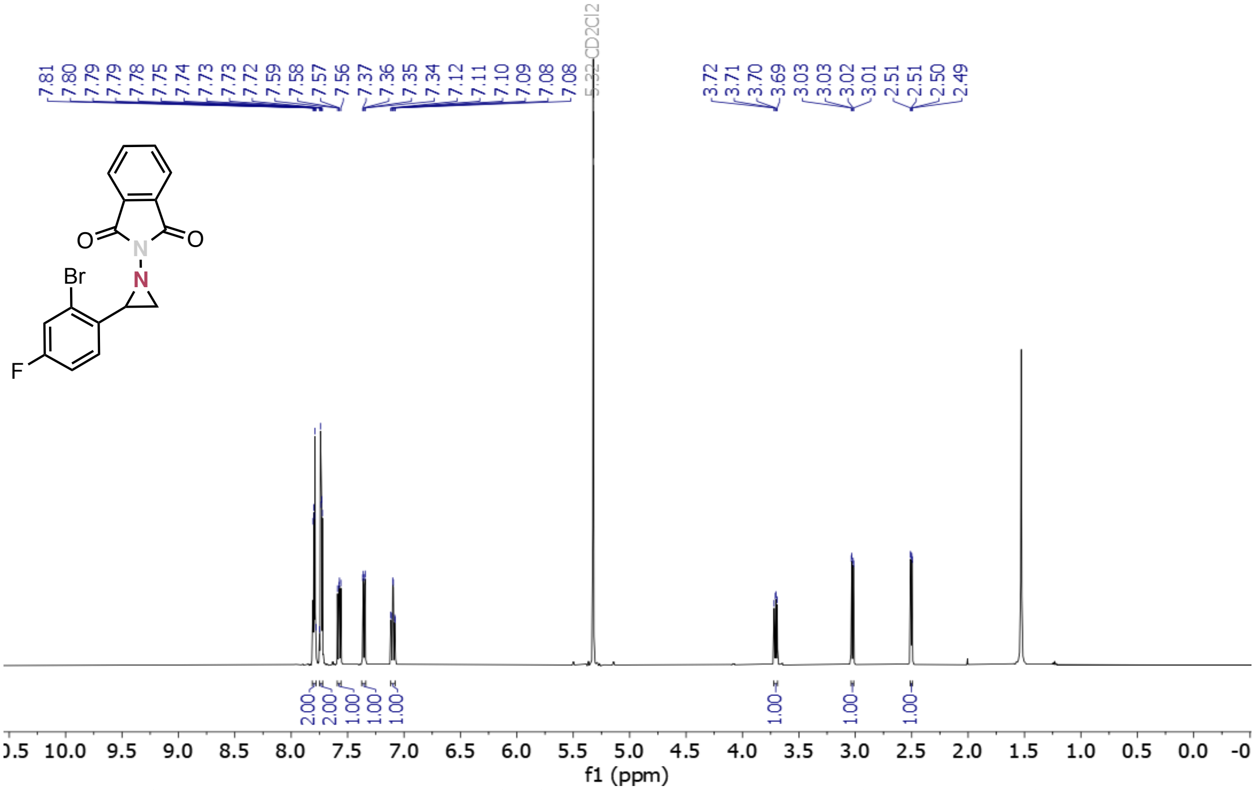

^{13}C NMR (126 MHz, CD_2Cl_2) of 2-(2-(2-bromo-4-fluorophenyl)aziridin-1-yl)isoindoline-1,3-dione (2af)

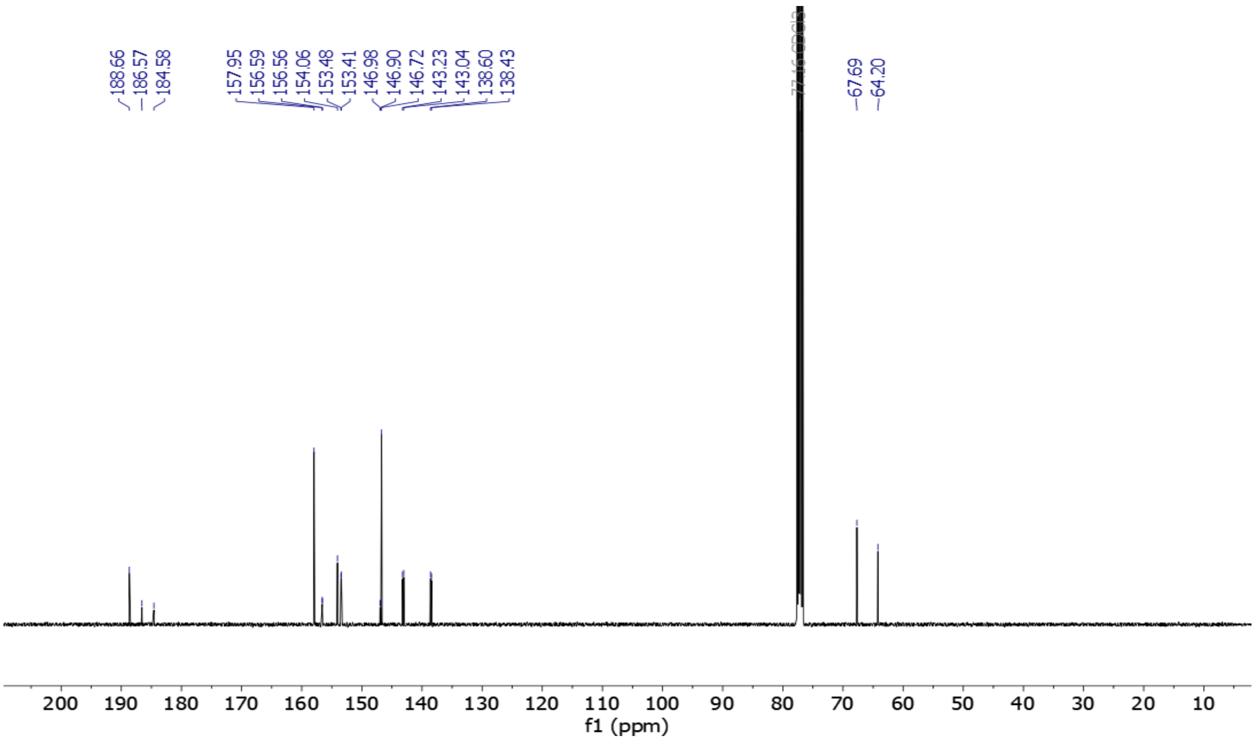

^{19}F NMR (471 MHz, CD_2Cl_2) of 2-(2-(2-bromo-4-fluorophenyl)aziridin-1-yl)isoindoline-1,3-dione (2af)

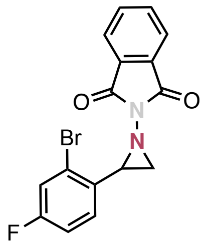

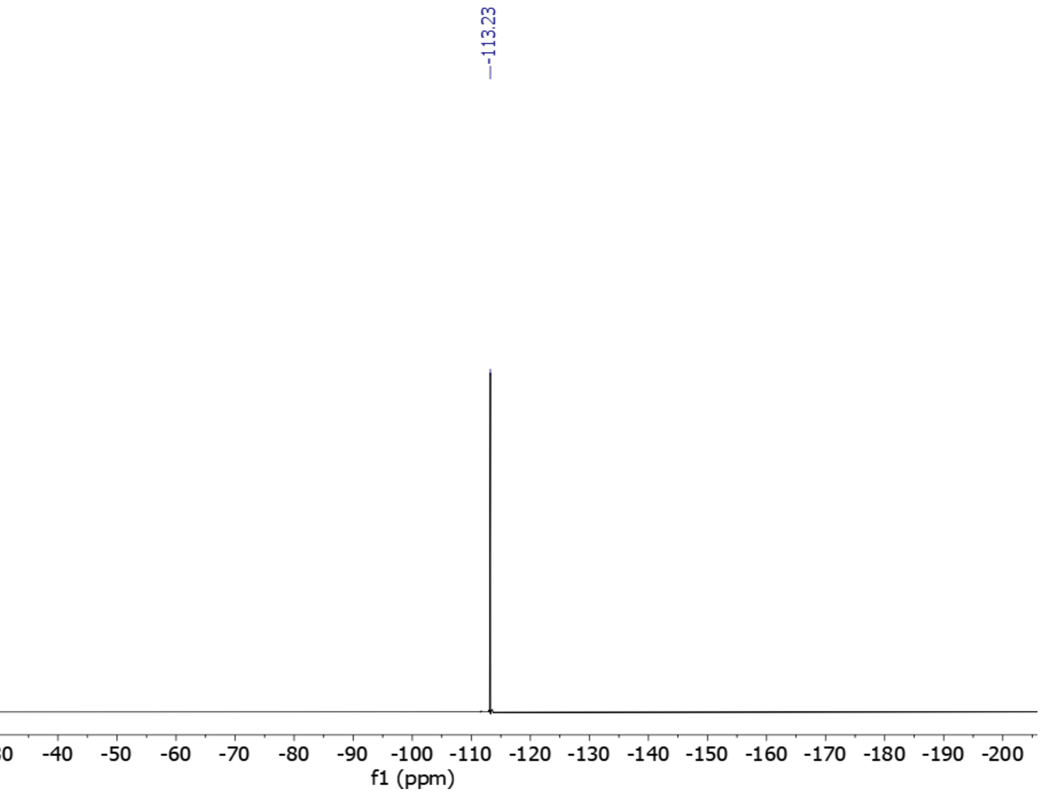

^{1}H NMR (500 MHz, $CDCl_3$) of 2-(2-(perfluorophenyl)aziridin-1-yl)isoindoline-1,3-dione (2ag)

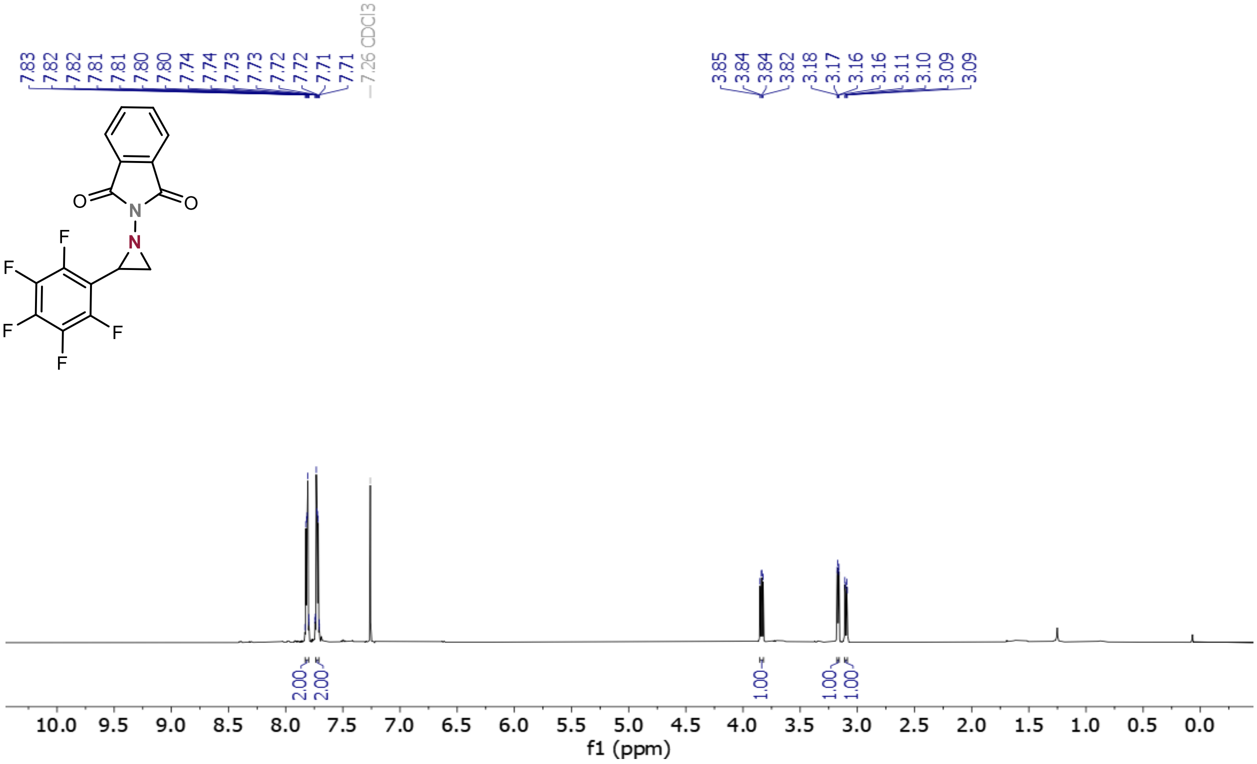

^{13}C NMR (126 MHz, CD_2Cl_2) of 2-(2-(perfluorophenyl)aziridin-1-yl)isoindoline-1,3-dione (2ag)

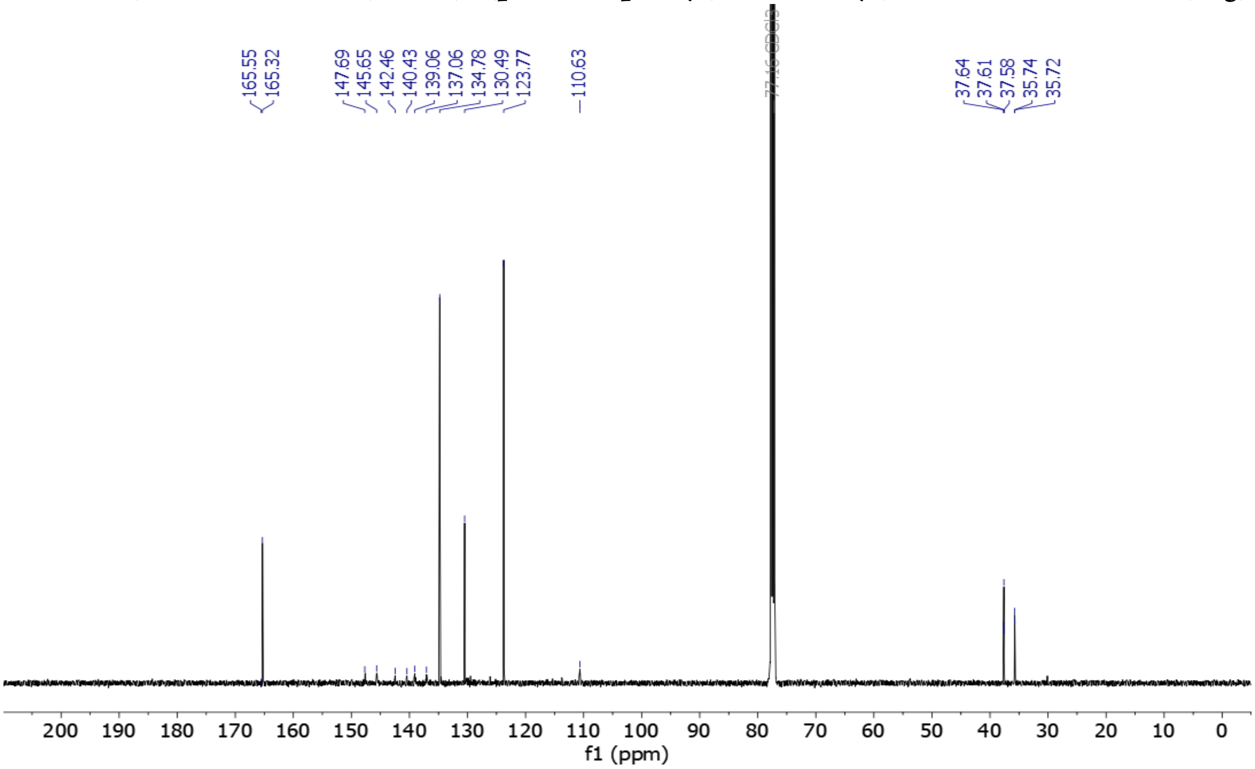

^{19}F NMR (471 MHz, $CDCl_3$) of 2-(2-(perfluorophenyl)aziridin-1-yl)isoindoline-1,3-dione (2ag)

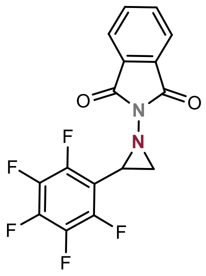

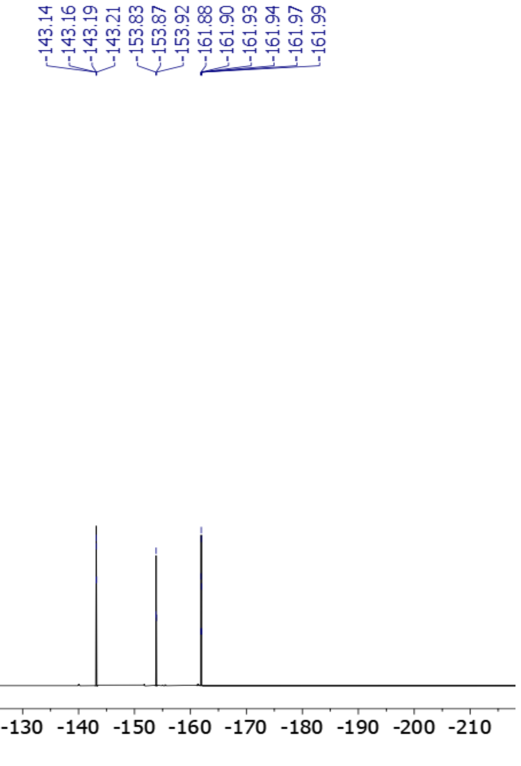

^{1}H NMR (500 MHz, $CDCl_3$) of 2-((2-(benzo[*d*][1,3]dioxol-5-yl)-2-ethoxyethyl)amino) isoindoline-1,3-dione (2ah1)

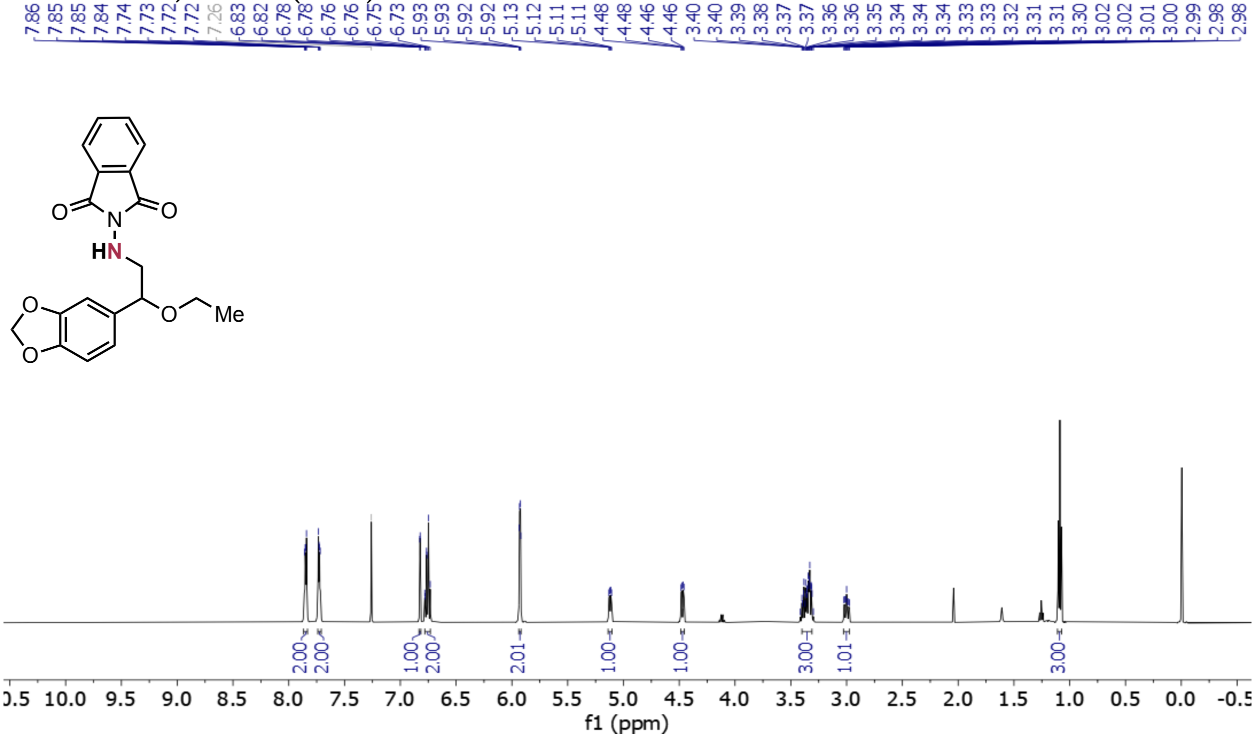

^{13}CNMR (126 MHz, $CDCl_3$) of 2-((2-(benzo[*d*][1,3]dioxol-5-yl)-2-ethoxyethyl)amino) isoindoline-1,3-dione (2ah1)

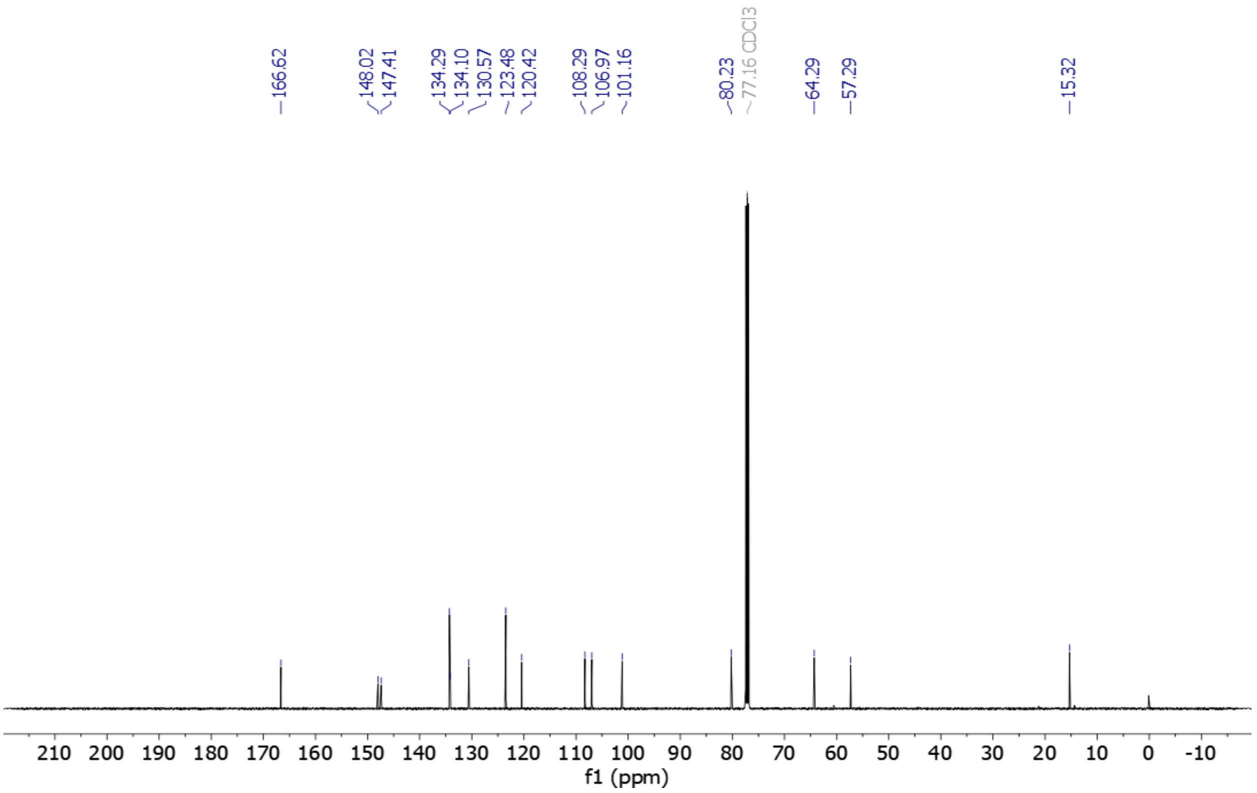

^{1}H NMR (500 MHz, $CDCl_3$) of 2-(2-(pyridin-4-yl)aziridin-1-yl)isoindoline-1,3-dione (2ai)

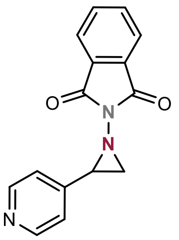

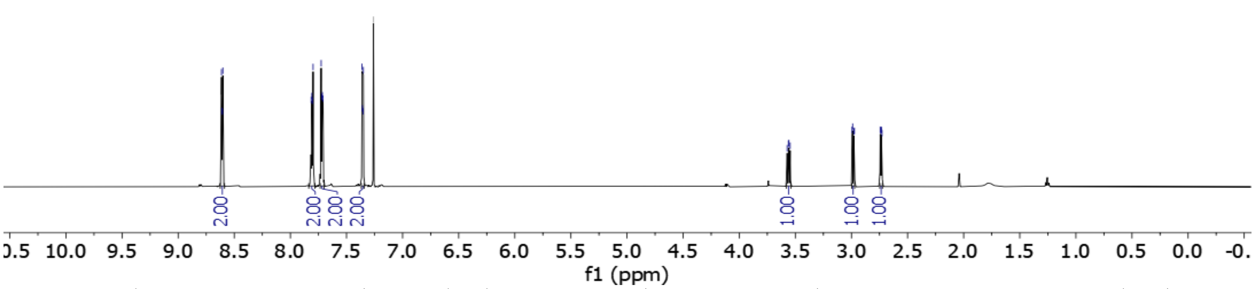

^{13}C NMR (126 MHz, CDCl3) of 2-(2-(pyridin-4-yl)aziridin-1-yl)isoindoline-1,3-dione (2ai)

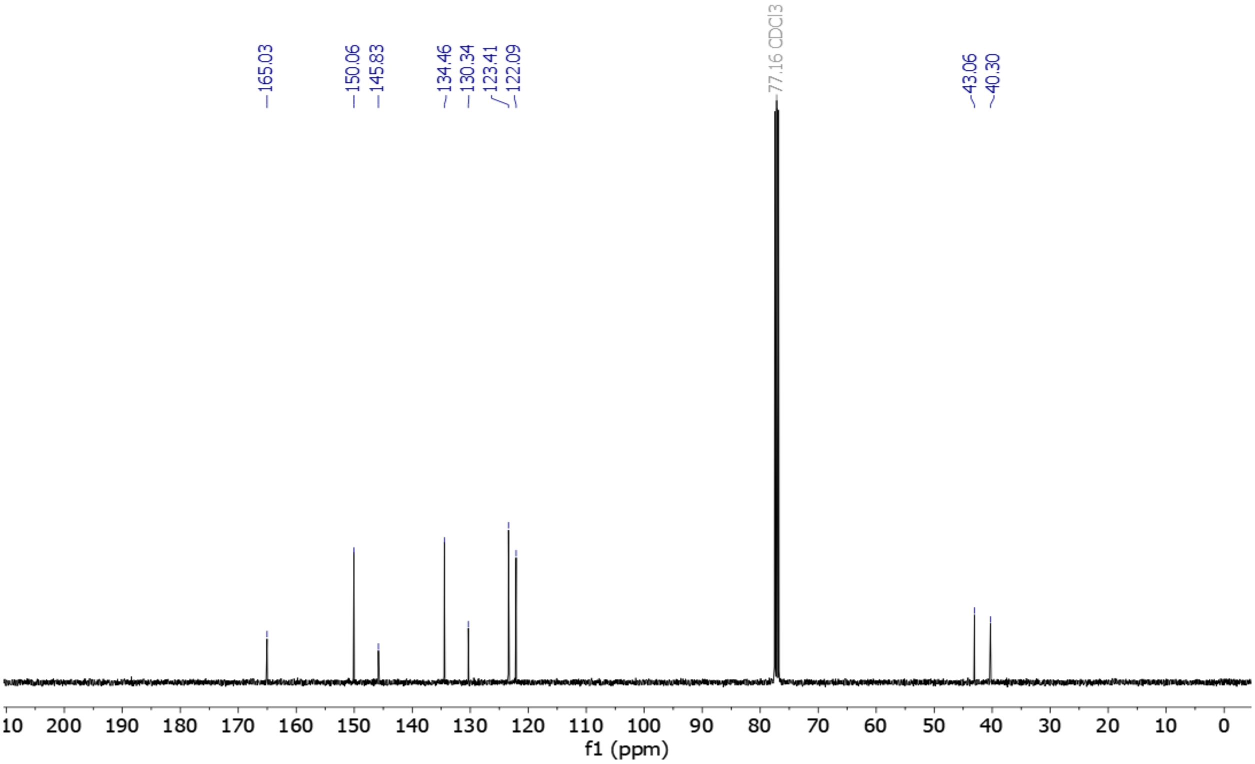

^{1}H NMR (500 MHz, $CDCl_3$) of 2-(2-(pyridin-2-yl)aziridin-1-yl)isoindoline-1,3-dione (2aj)

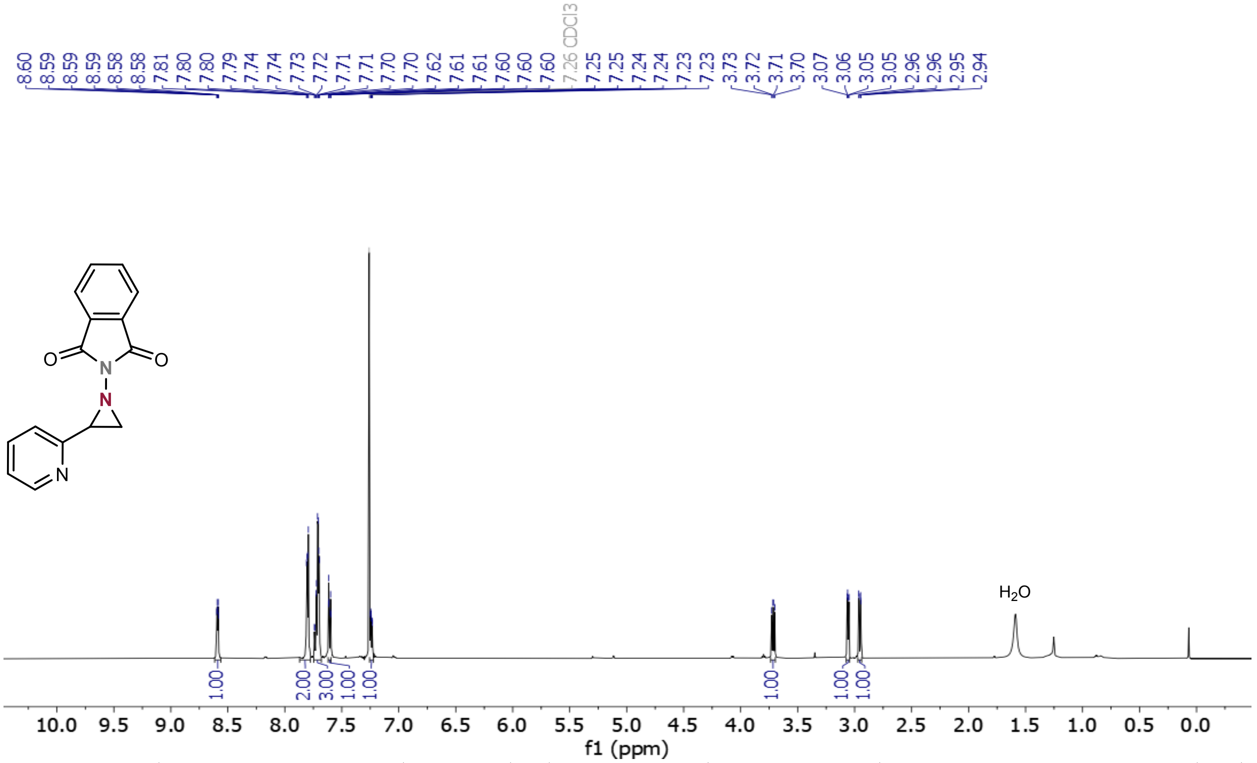

^{13}C NMR (126 MHz, $CDCl_3$) of 2-(2-(pyridin-2-yl)aziridin-1-yl)isoindoline-1,3-dione (2aj)

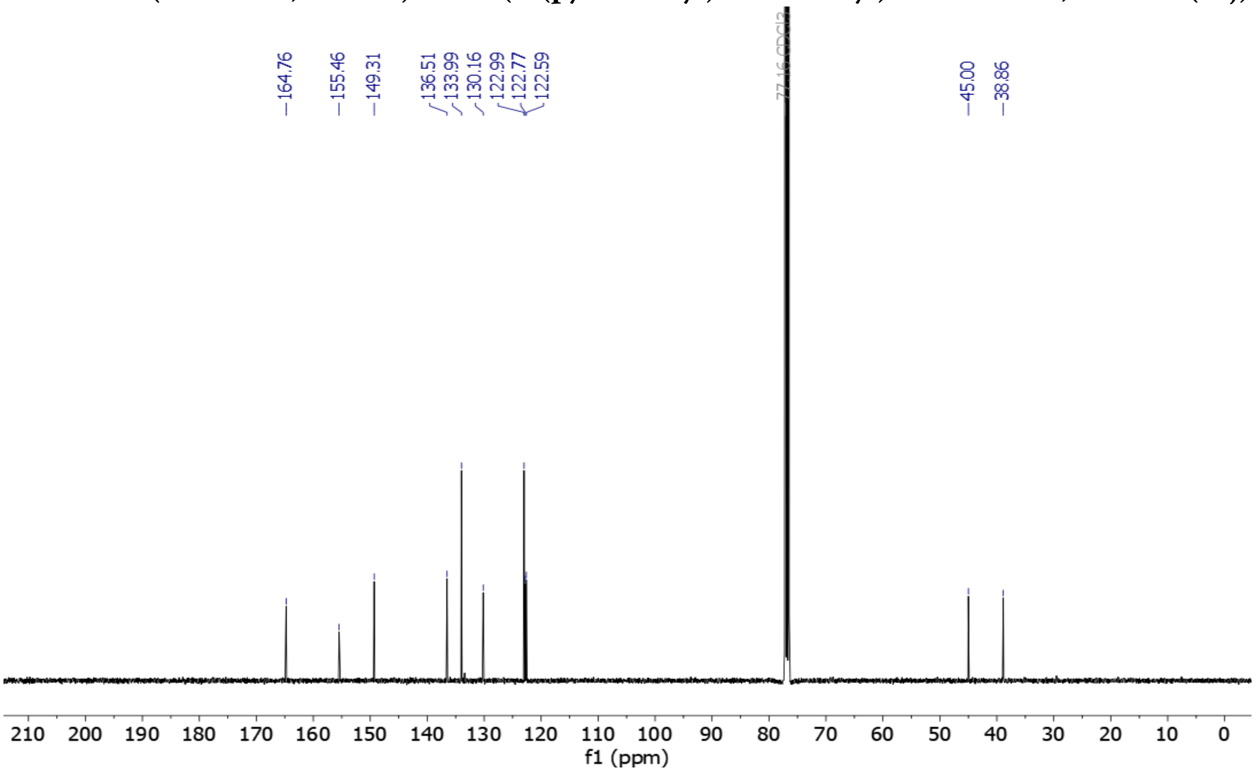

^{1}H NMR (600 MHz, $CDCl_3$) of 2-(2-(1*H*-imidazol-1-yl)aziridin-1-yl)isoindoline-1,3-dione (2ak)

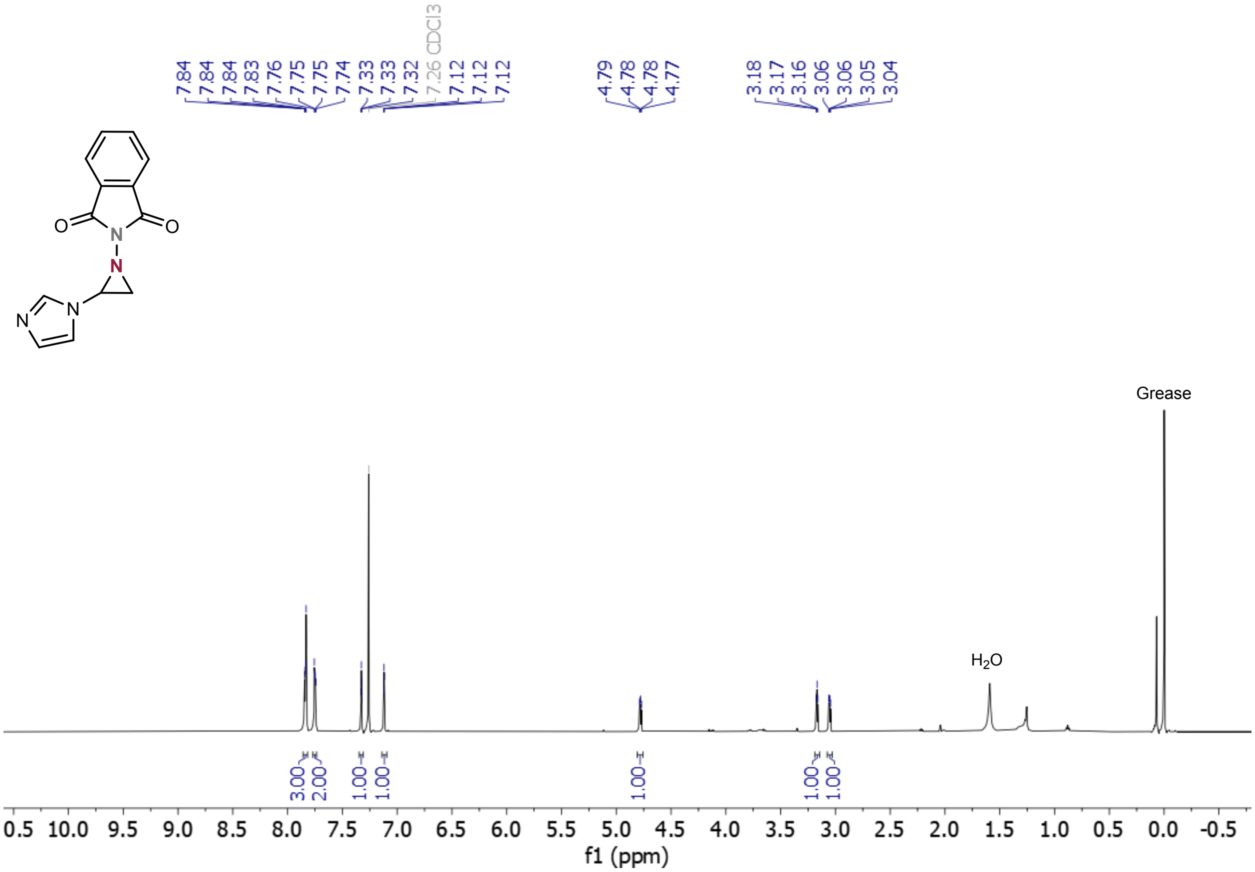

^{13}C NMR (126 MHz, $CDCl_3$) of 2-(2-(1*H*-imidazol-1-yl)aziridin-1-yl)isoindoline-1,3-dione (2ak)

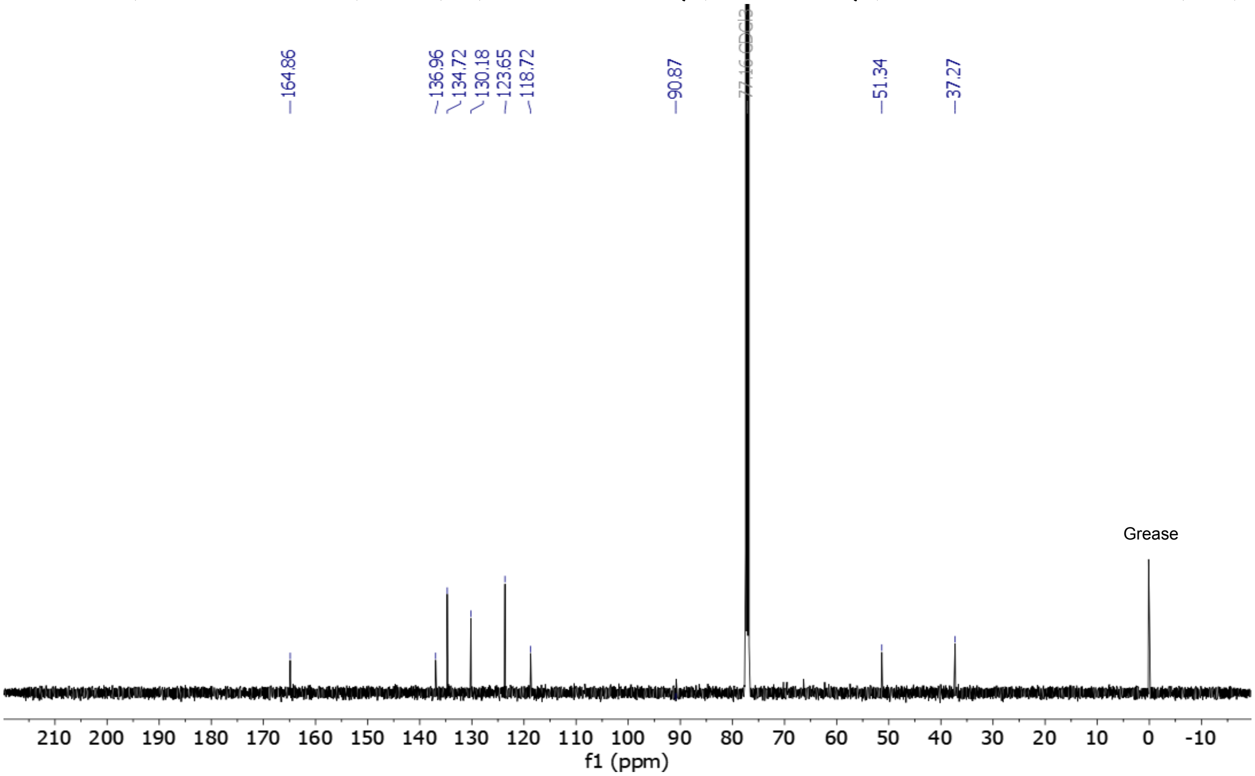

^{1}H NMR (500 MHz, $CDCl_3$) of 2-(-9-azabicyclo[6.1.0]nonan-9-yl)isoindoline-1,3-dione (2al)

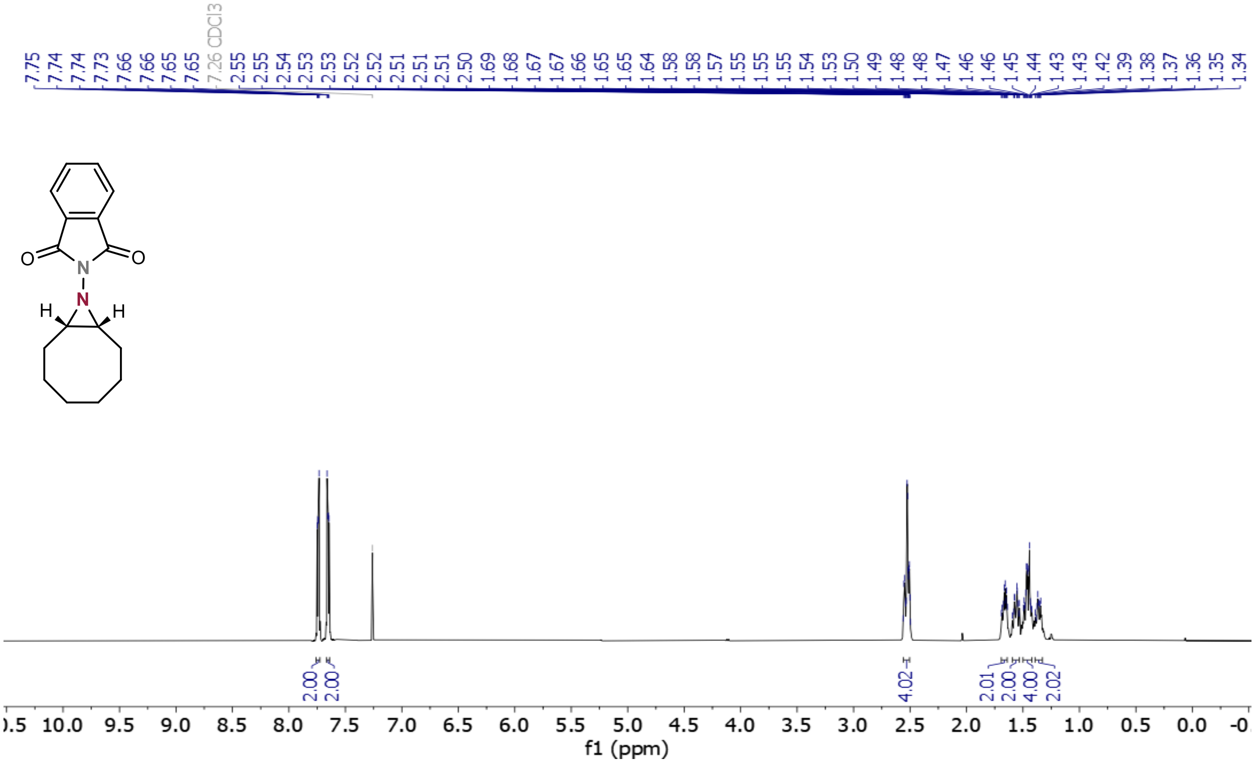

^{13}C NMR (126 MHz, $CDCl_3$) 2-(-9-azabicyclo[6.1.0]nonan-9-yl)isoindoline-1,3-dione (2al)

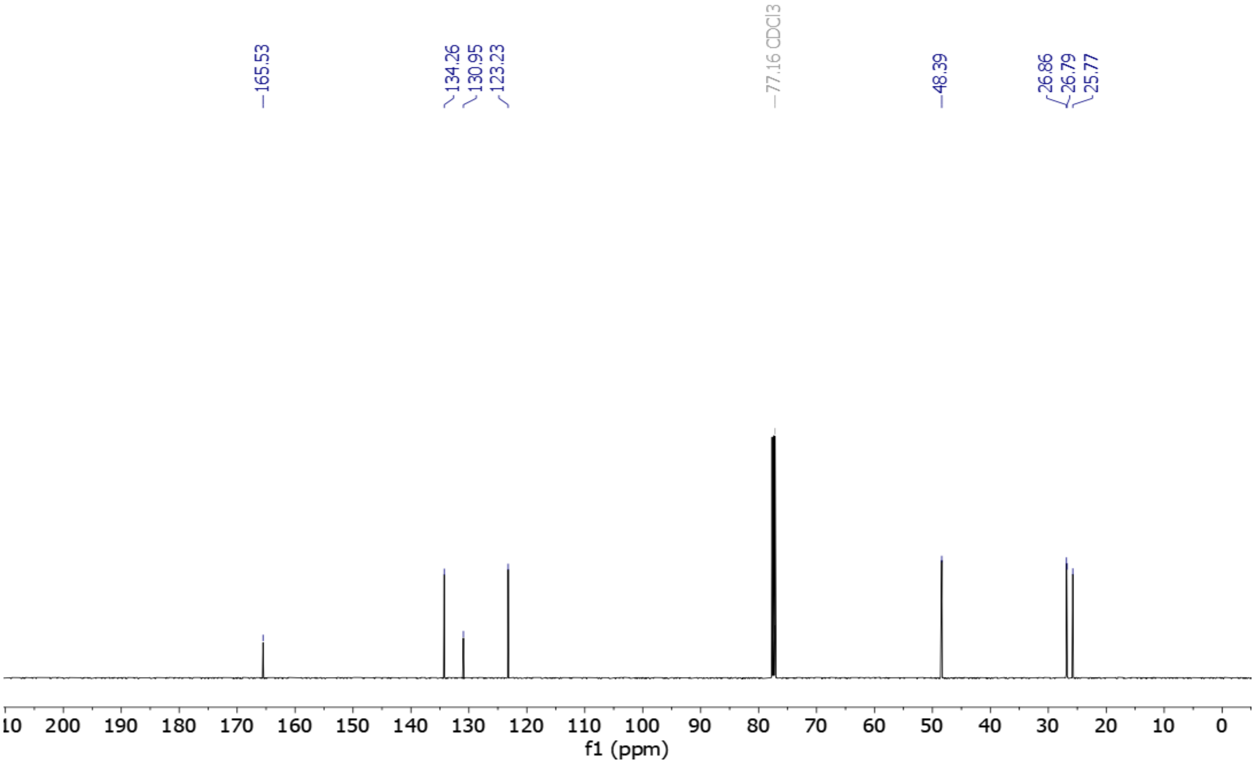

^{1}H NMR (500 MHz, $CDCl_3$) of 2-(7-azabicyclo[4.1.0]heptan-7-yl)isoindoline-1,3-dione (2am)

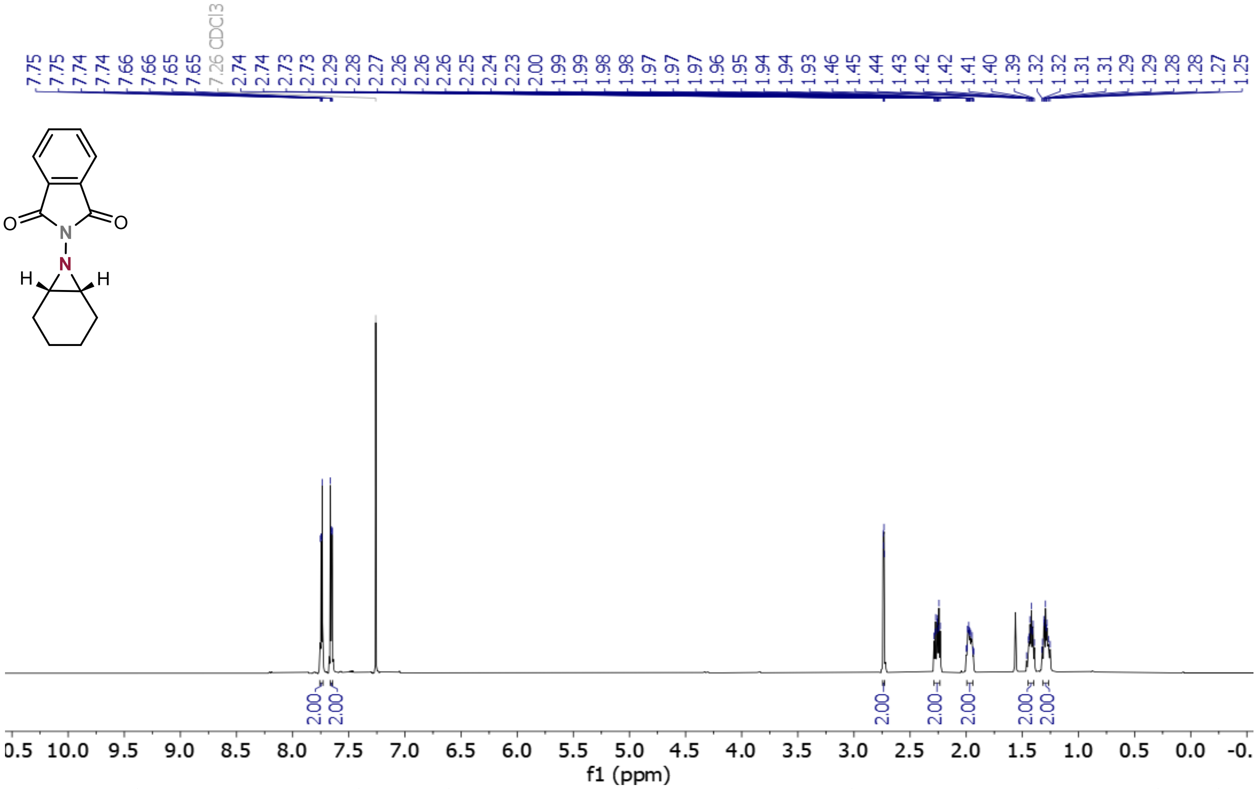

^{13}C NMR (126 MHz, $CDCl_3$) of 2-(7-azabicyclo[4.1.0]heptan-7-yl)isoindoline-1,3-dione (2am)

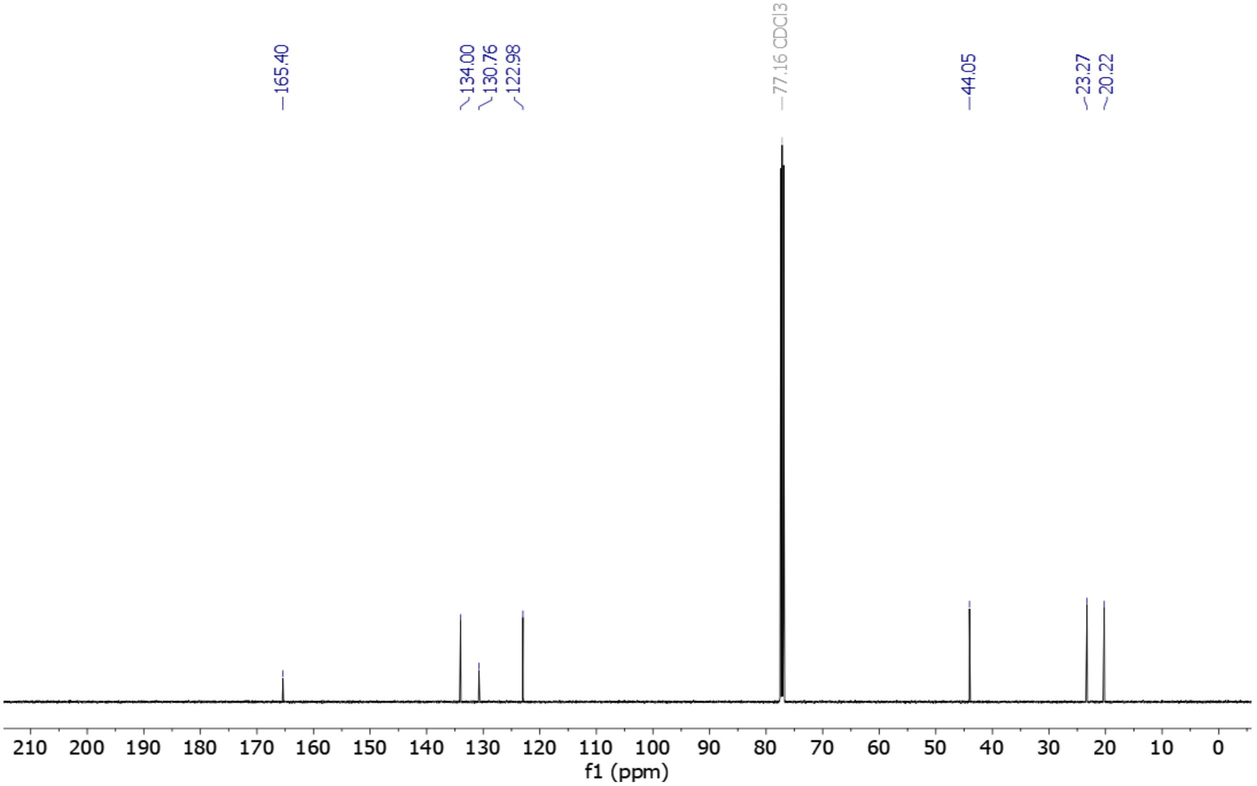

^{1}H NMR (500 MHz, $CDCl_3$) of 2-(3-azatricyclo[3.2.1.0^{2,4}]octan-3-yl)isoindoline-1,3-dione (2an)

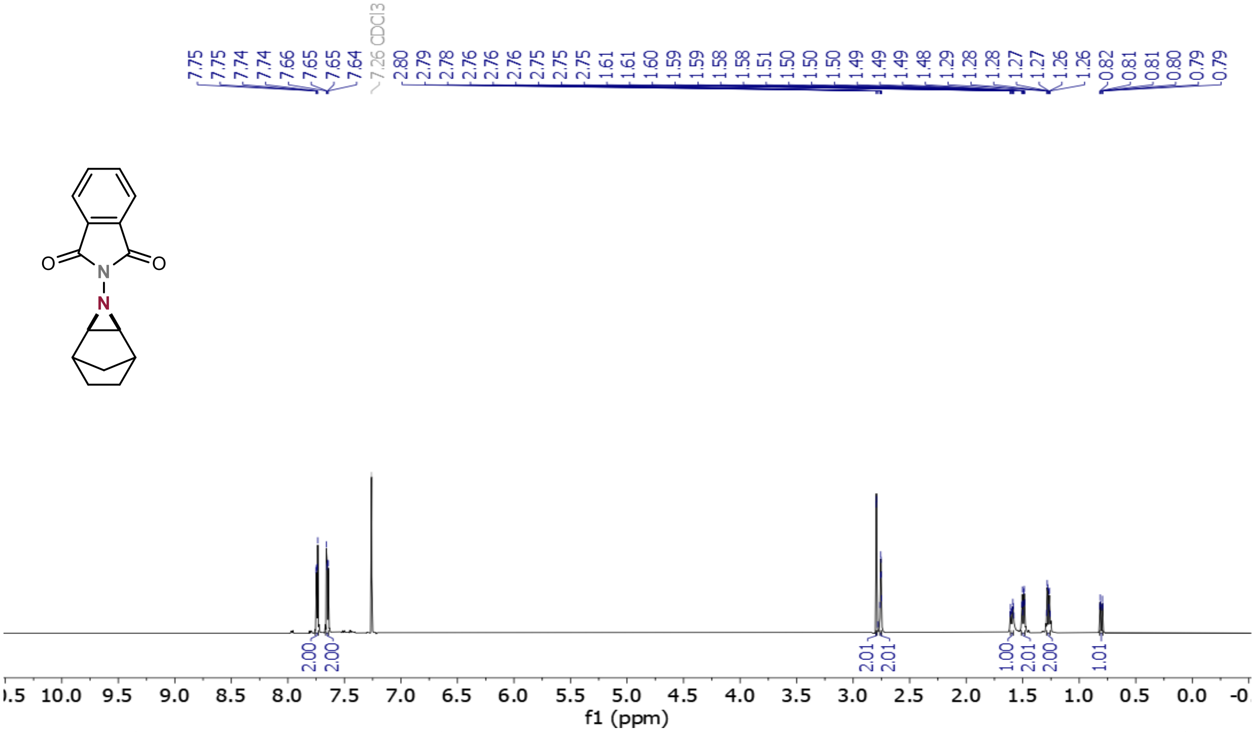

^{13}C NMR (126 MHz, $CDCl_3$) of 2-(3-azatricyclo[3.2.1.0^{2,4}]octan-3-yl)isoindoline-1,3-dione (2an)

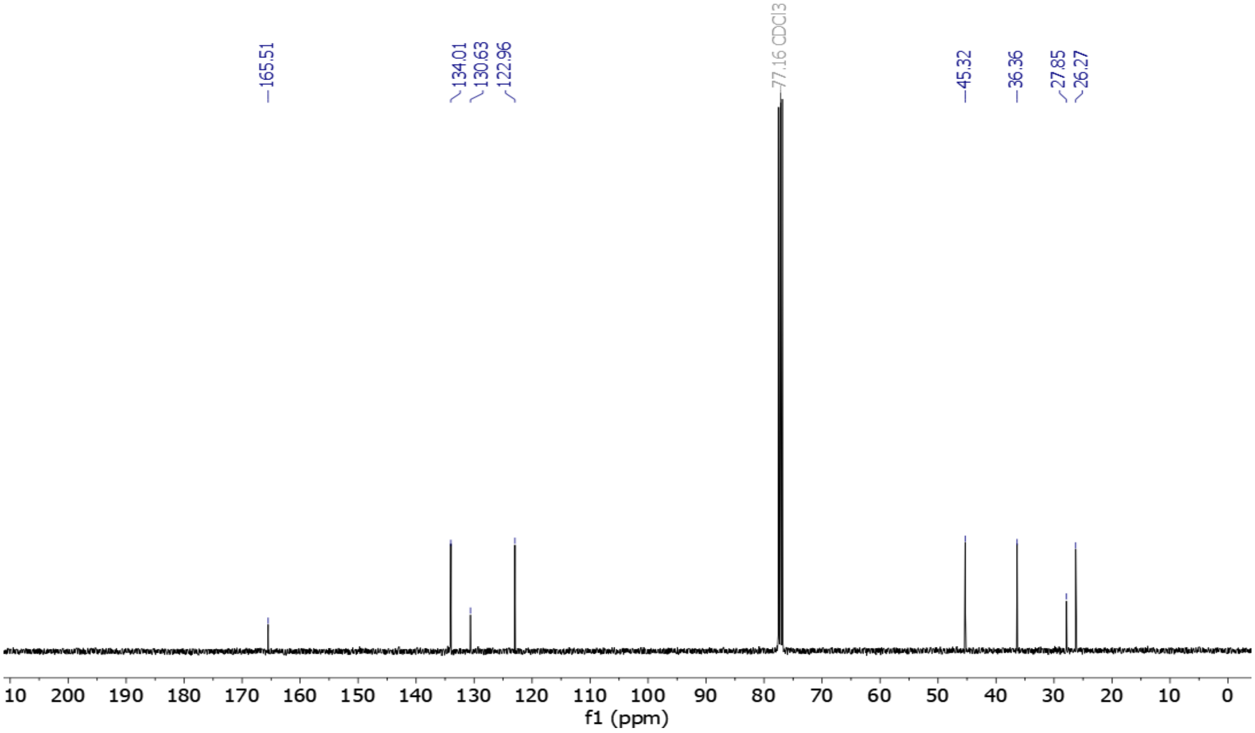

^{1}H NMR (500 MHz, $CDCl_3$) of 2-(1-methyl-7-azabicyclo[4.1.0]heptan-7-yl)isoindoline-1,3-dione (2ao)

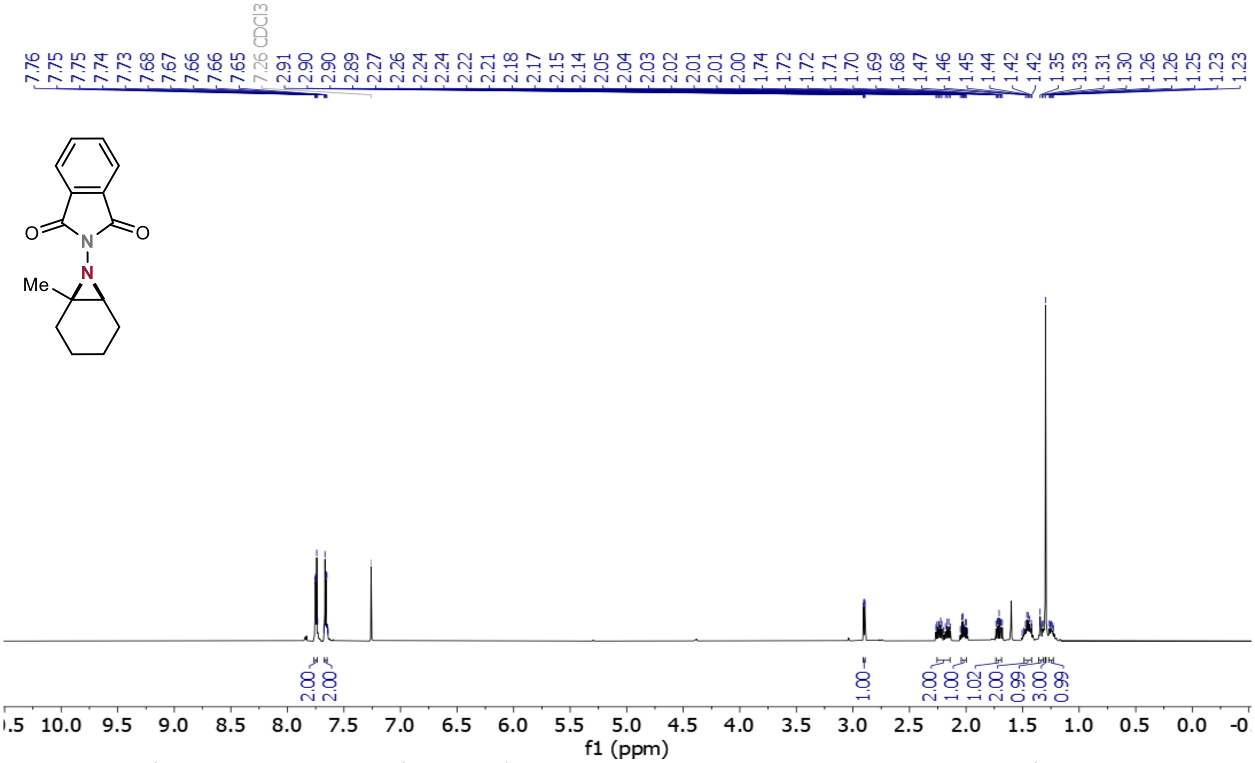

^{13}C NMR (126 MHz, CDCl3) of 2-(1-methyl-7-azabicyclo[4.1.0]heptan-7-yl)isoindoline-1,3-dione (2ao)

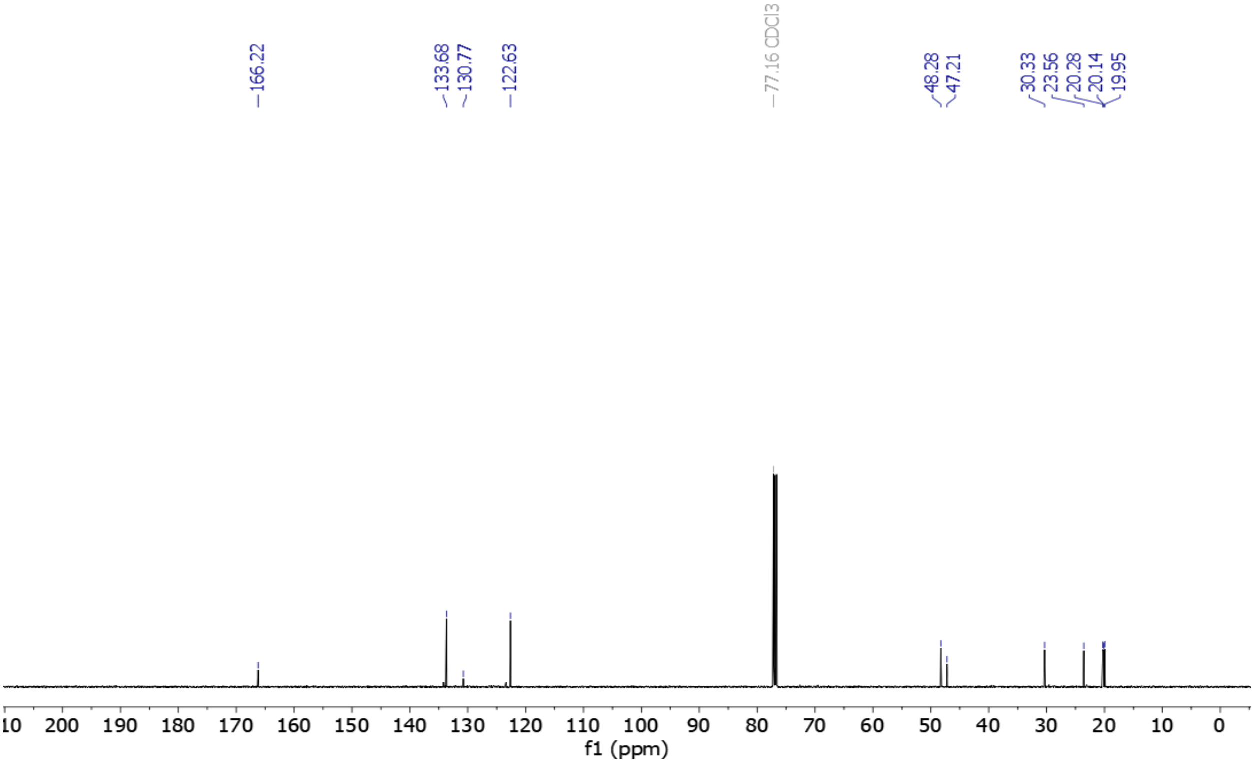

^{1}H NMR (400 MHz, $CDCl_3$) of 2-(2-(2-bromoethyl)aziridin-1-yl)isoindoline-1,3-dione (2ap)

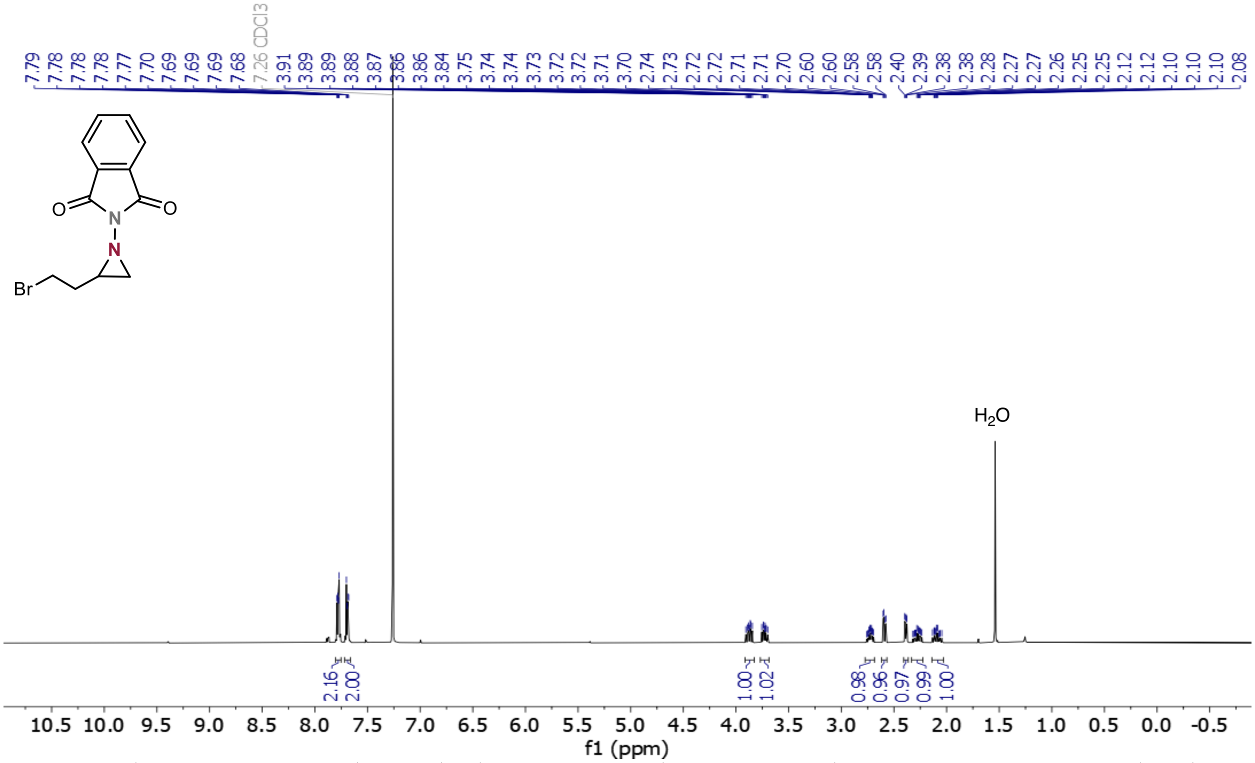

^{13}C NMR (101 MHz, $CDCl_3$) of 2-(2-(2-bromoethyl)aziridin-1-yl)isoindoline-1,3-dione (2ap)

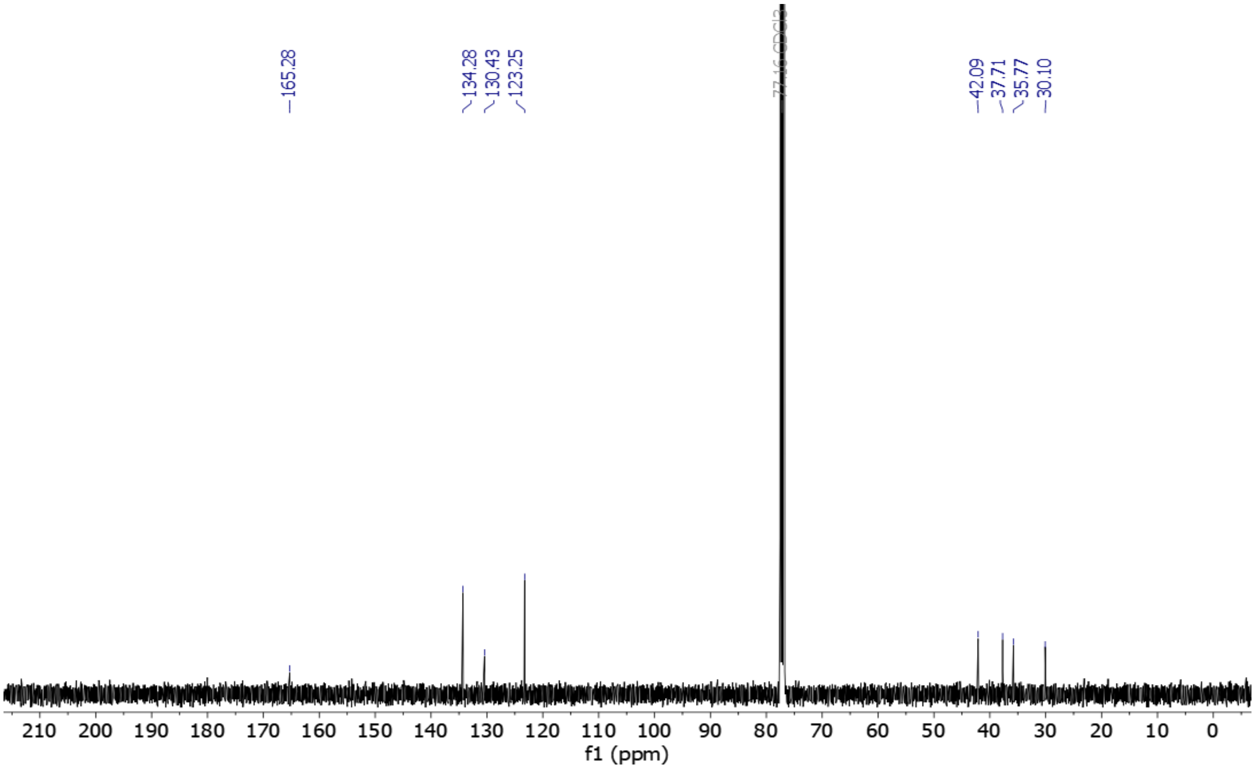

^{1}H NMR (400 MHz, $CDCl_3$) of 2-(2-decylaziridin-1-yl)isoindoline-1,3-dione (2aq)

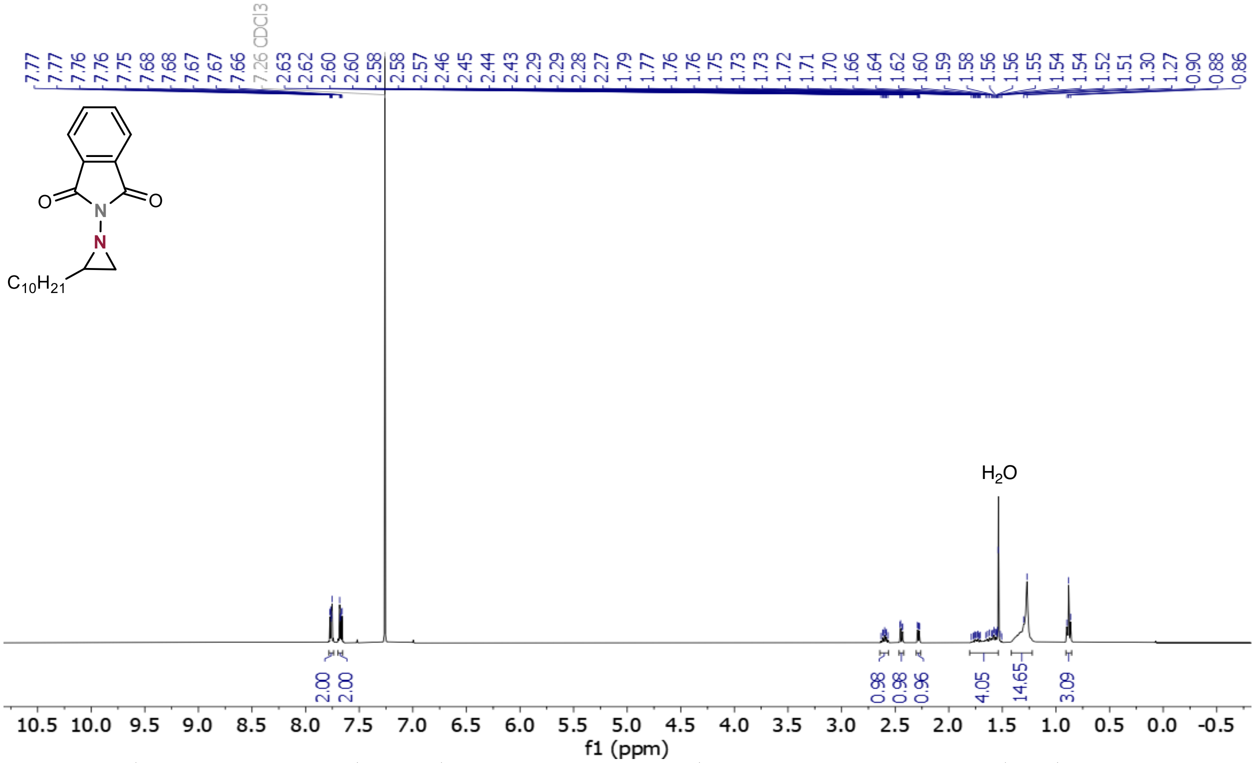

^{13}C NMR (101 MHz, $CDCl_3$) of 2-(2-decylaziridin-1-yl)isoindoline-1,3-dione (2aq)

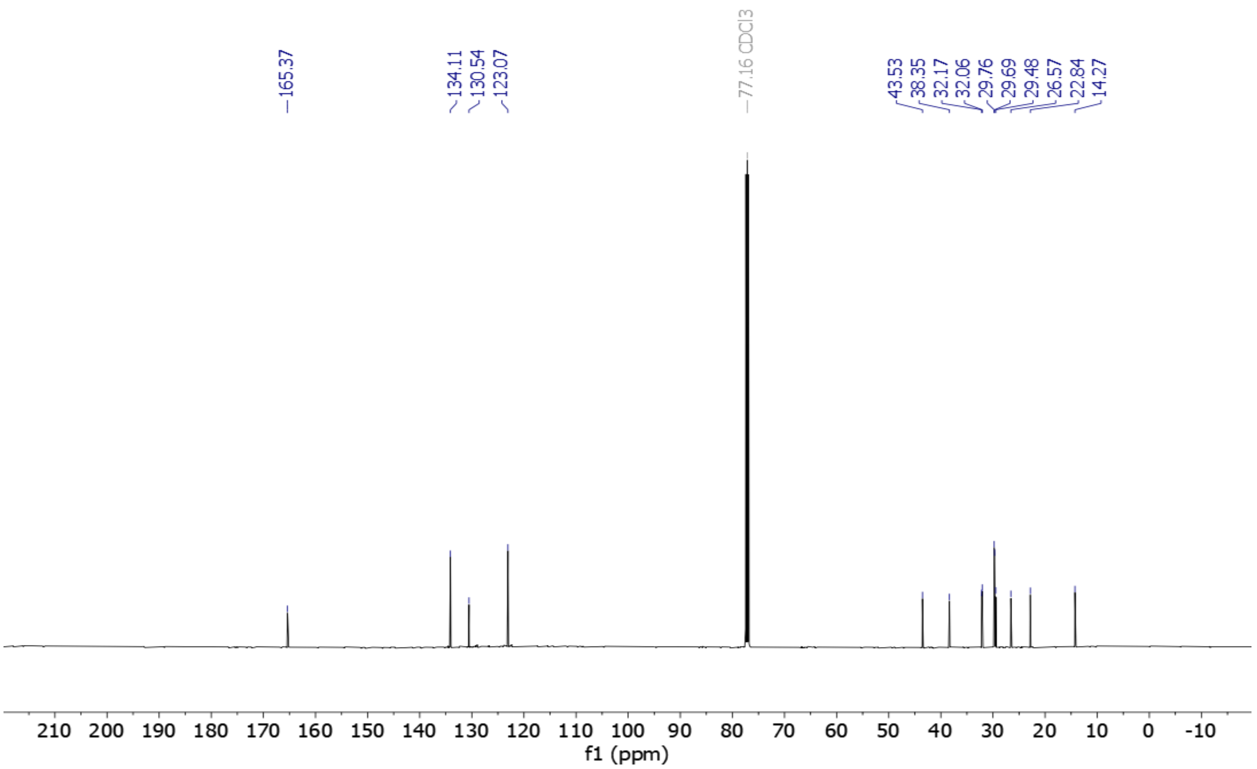

^{1}H NMR (400 MHz, $CDCl_3$) of *Trans* 2-(2,3-dibutylaziridin-1-yl)isoindoline-1,3-dione (2ar)

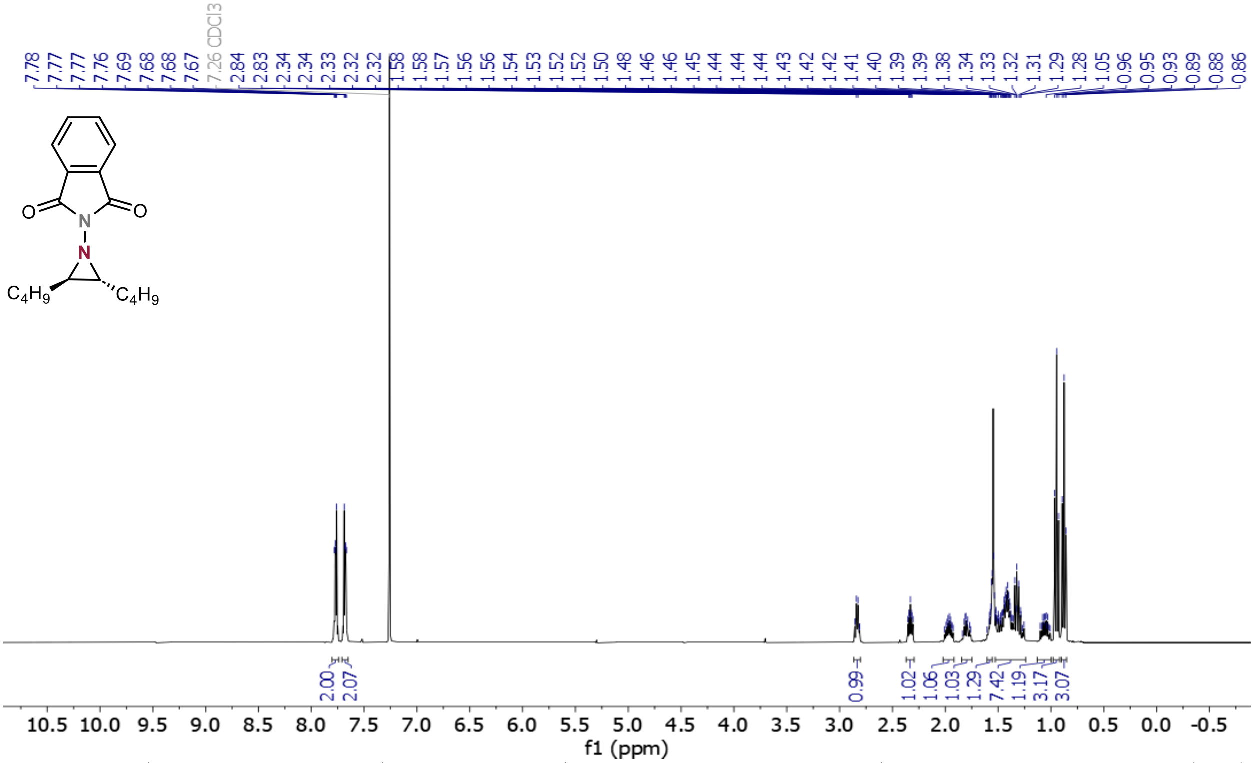

^{13}C NMR (101 MHz, $CDCl_3$) of *Trans* 2-(-2,3-dibutylaziridin-1-yl)isoindoline-1,3-dione (2ar)

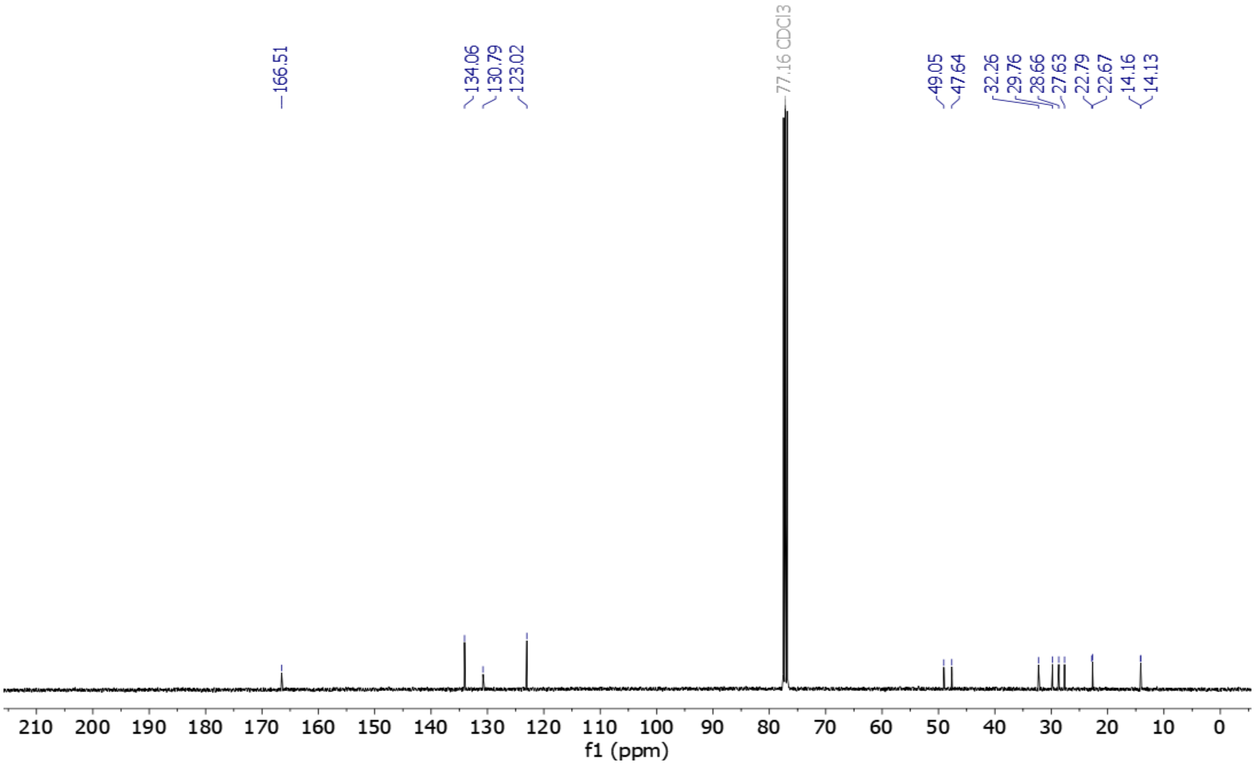

^{1}H NMR (500 MHz, $CDCl_3$) of 2-(2,2,3,3-tetramethylaziridin-1-yl)isoindoline-1,3-dione (2as)

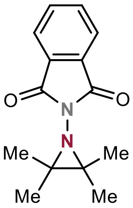

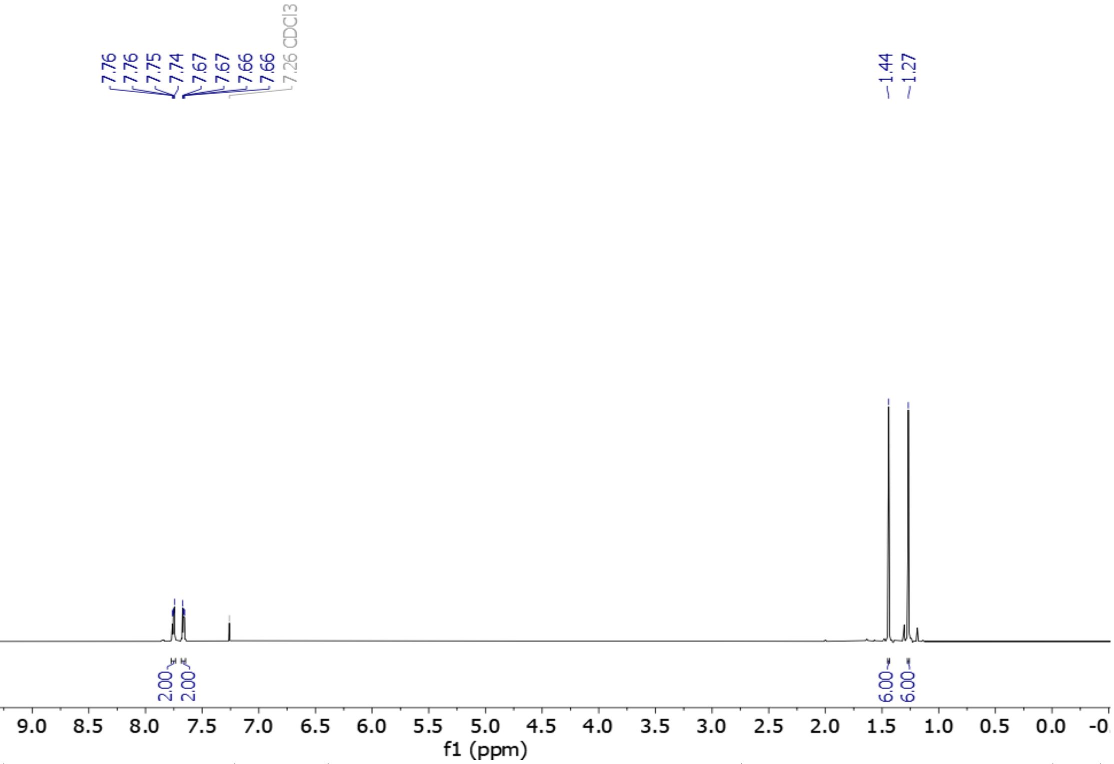

^{13}C NMR (126 MHz, $CDCl_3$) of 2-(2,2,3,3-tetramethylaziridin-1-yl)isoindoline-1,3-dione (2as)

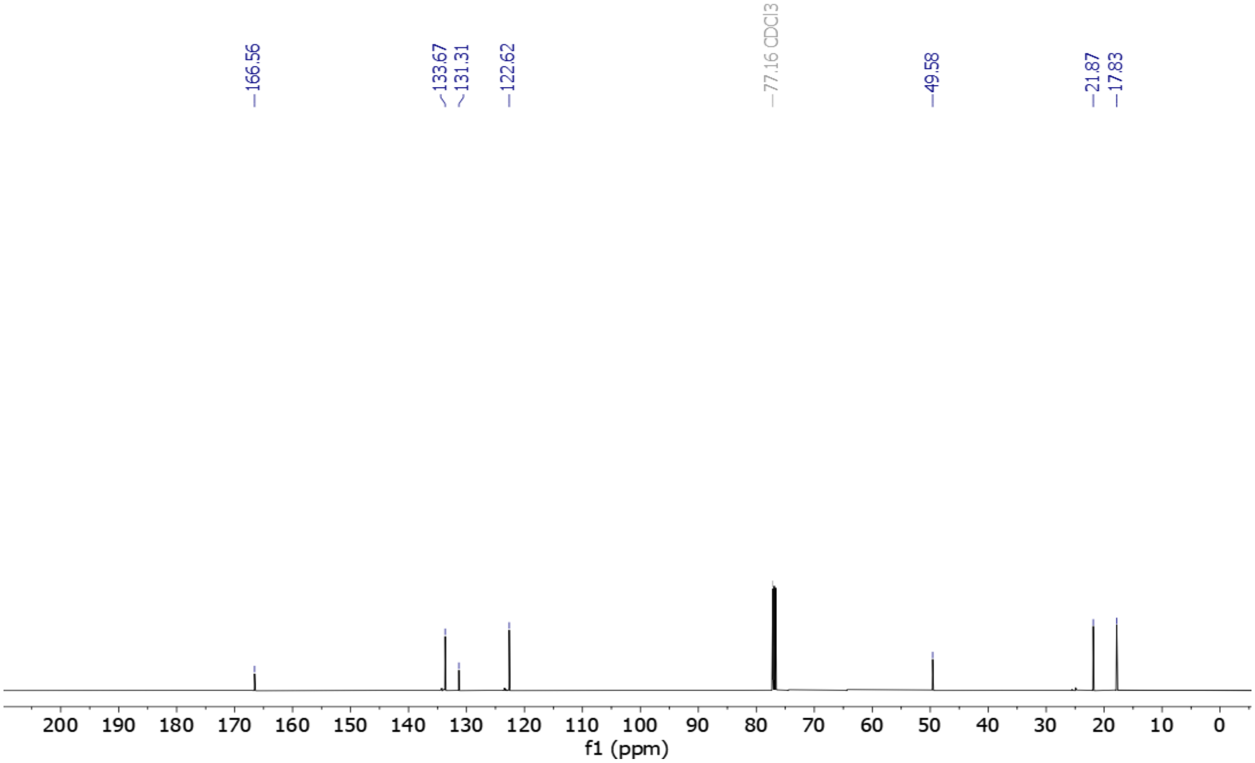

^{1}H NMR (400 MHz, $CDCl_3$) of 2-(7-azabicyclo[4.1.0]heptan-7-yl)isoindoline-1,3-dione (2at)

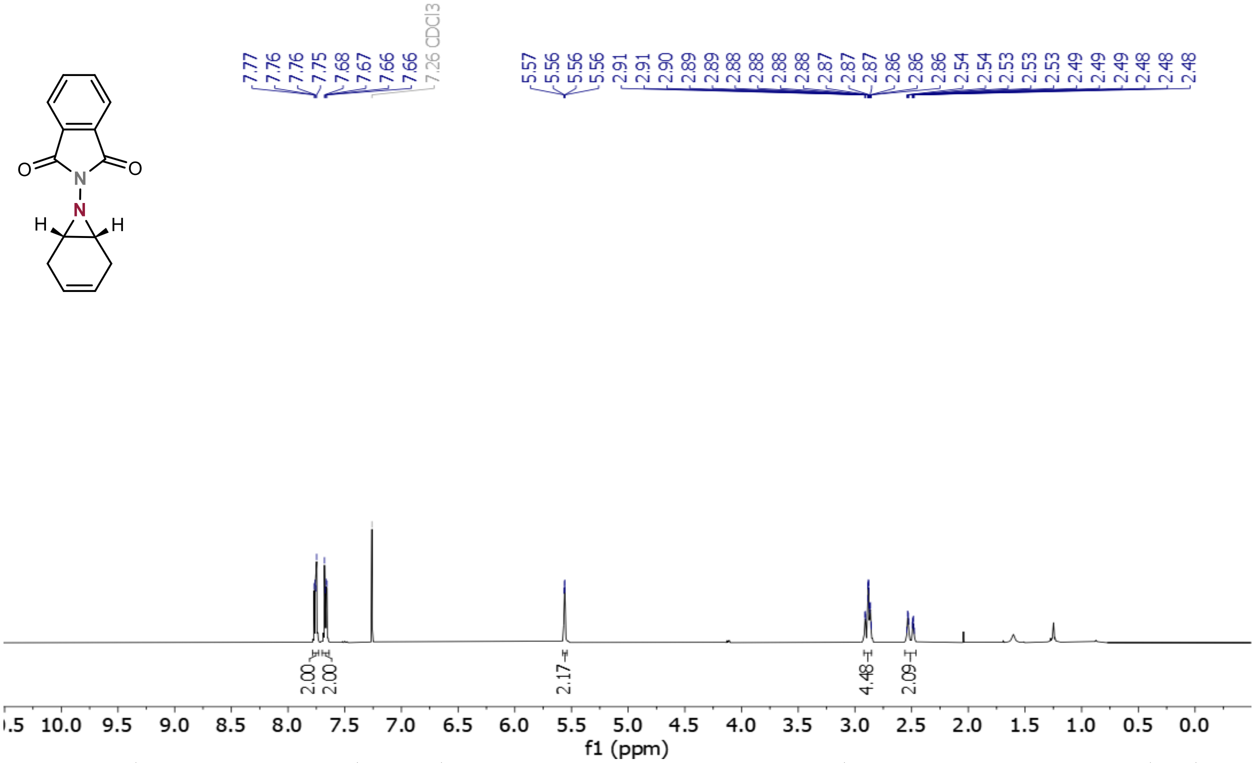

^{13}C NMR (101 MHz, $CDCl_3$) of 2-(7-azabicyclo[4.1.0]heptan-7-yl)isoindoline-1,3-dione (2at)

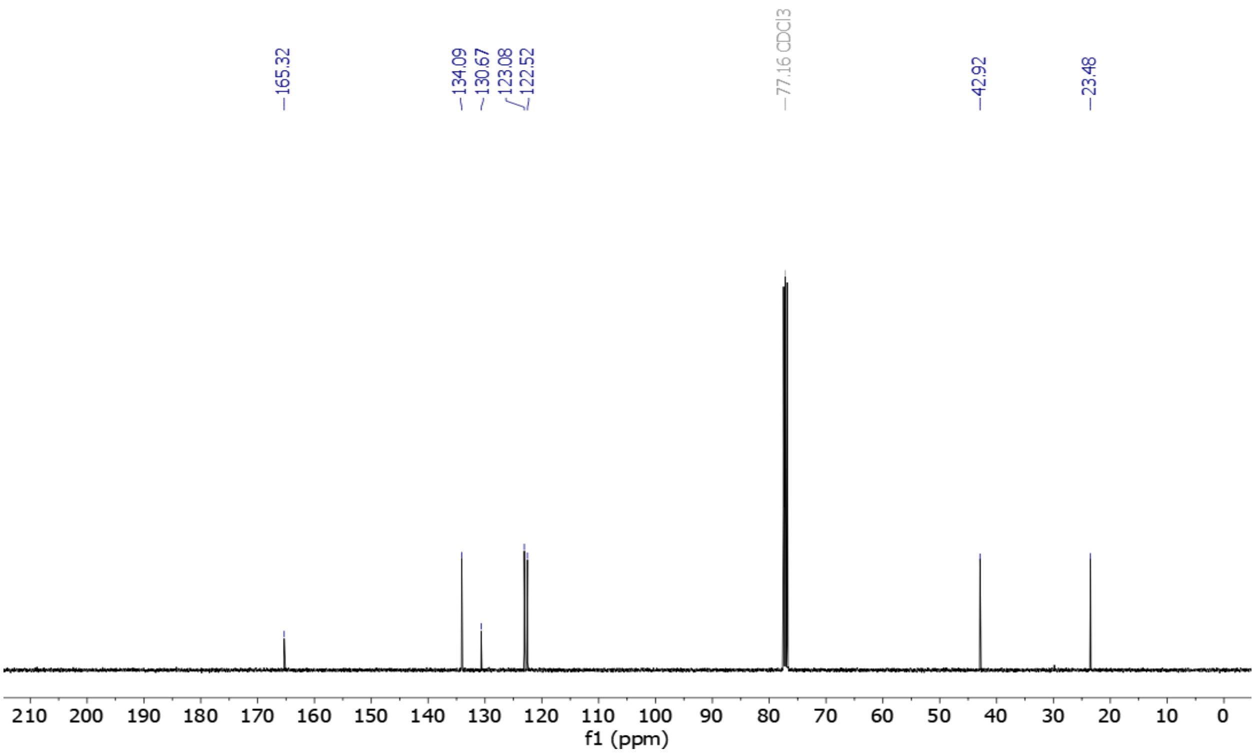

^{1}H NMR (500 MHz, $CDCl_3$) of *Cis* 2-(3-(prop-1-en-2-yl)-7-azabicyclo[4.1.0]heptan-7-yl)isoindoline-1,3-dione (2au)

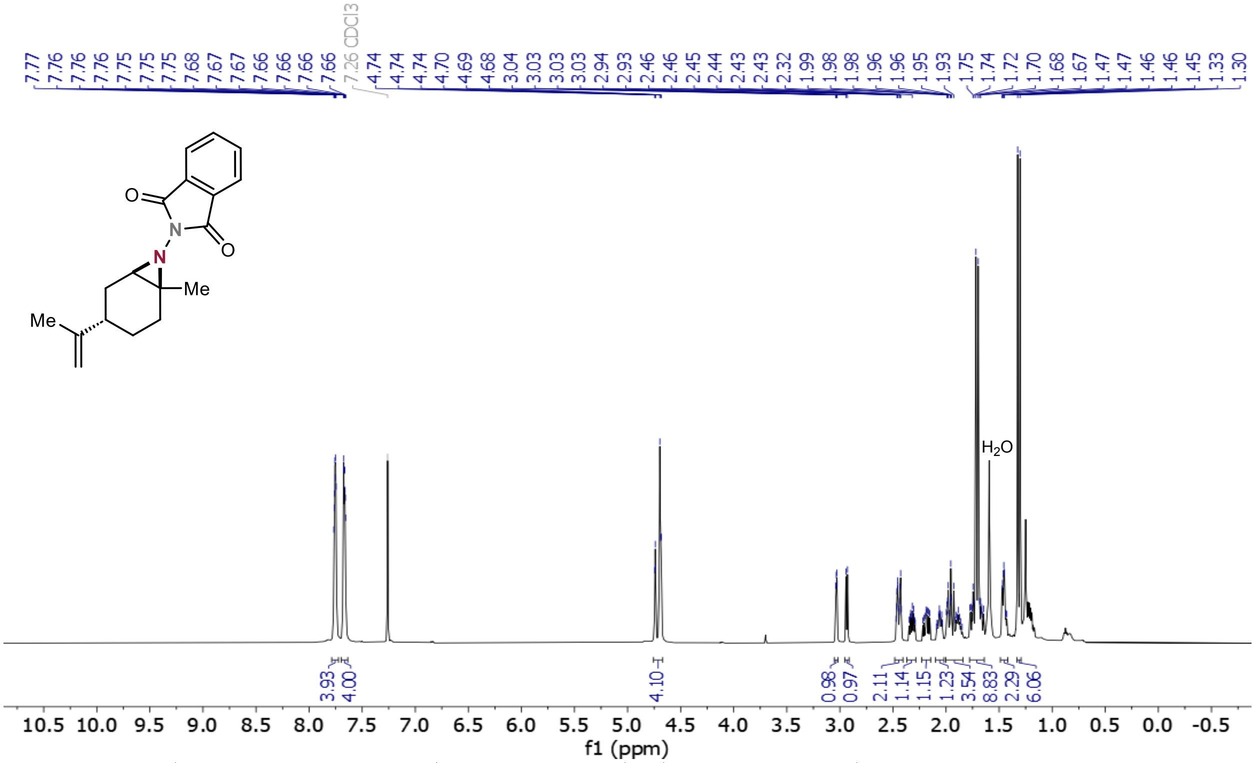

^{13}C NMR (126 MHz, $CDCl_3$) of *Cis* 2-(3-(prop-1-en-2-yl)-7-azabicyclo[4.1.0]heptan-7-yl)isoindoline-1,3-dione (2au)

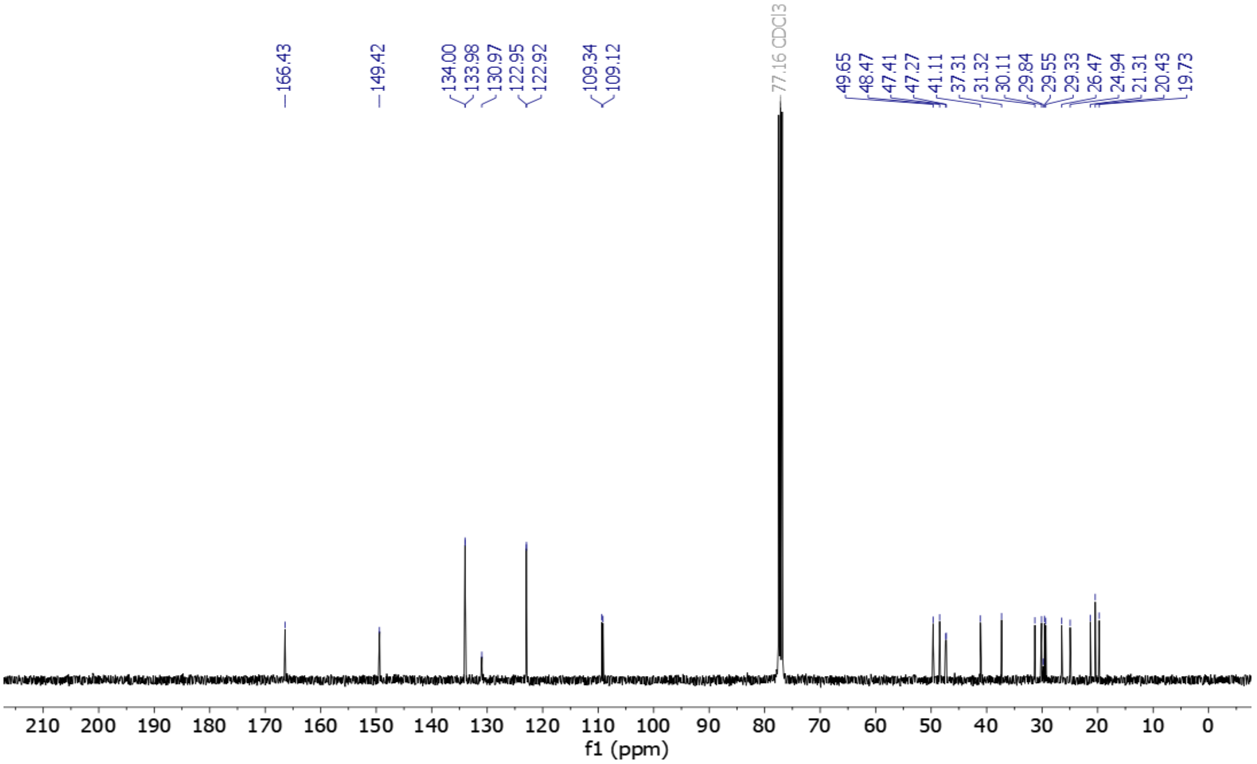

^{1}H NMR (400 MHz, $CDCl_3$) of 2-(4-isopropyl-1-methyl-7-azabicyclo[4.1.0]hept-3-en-7-yl)isoindoline-1,3-dione (2av)

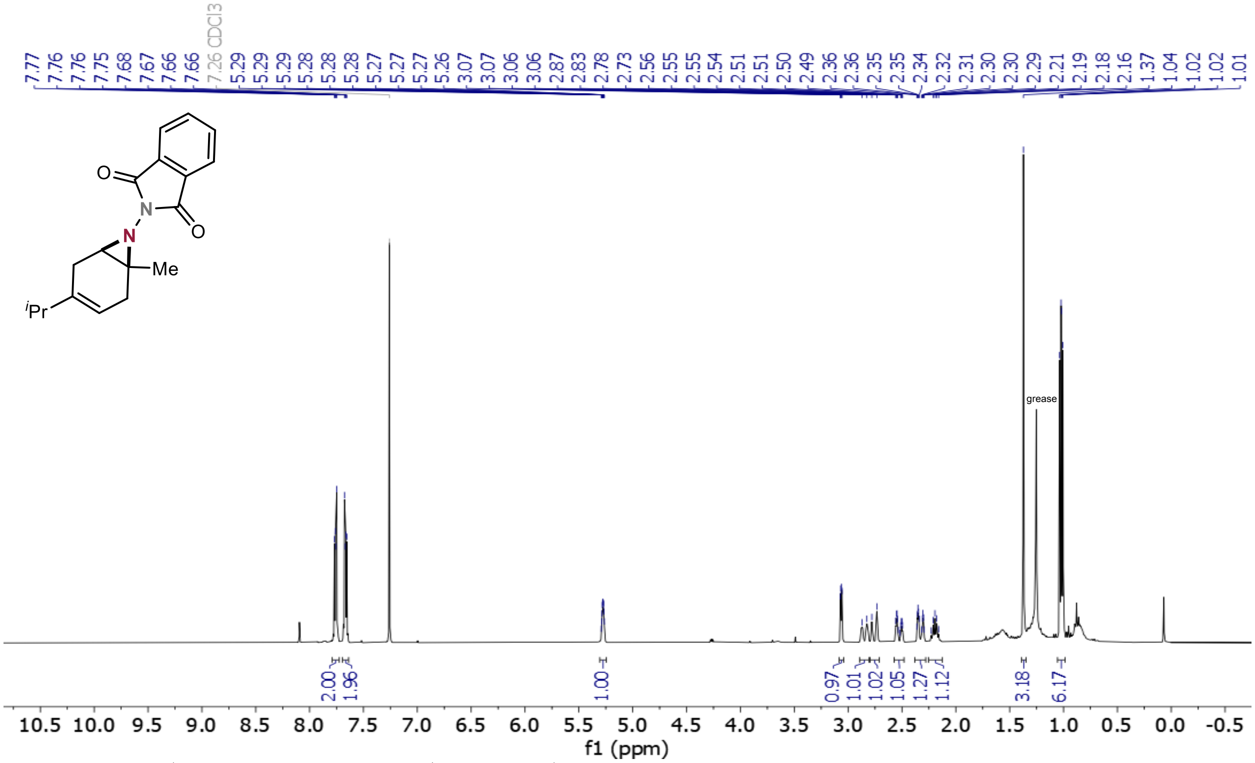

^{13}C NMR (101 MHz, $CDCl_3$) of 2-(4-isopropyl-1-methyl-7-azabicyclo[4.1.0]hept-3-en-7-yl)isoindoline-1,3-dione (2av)

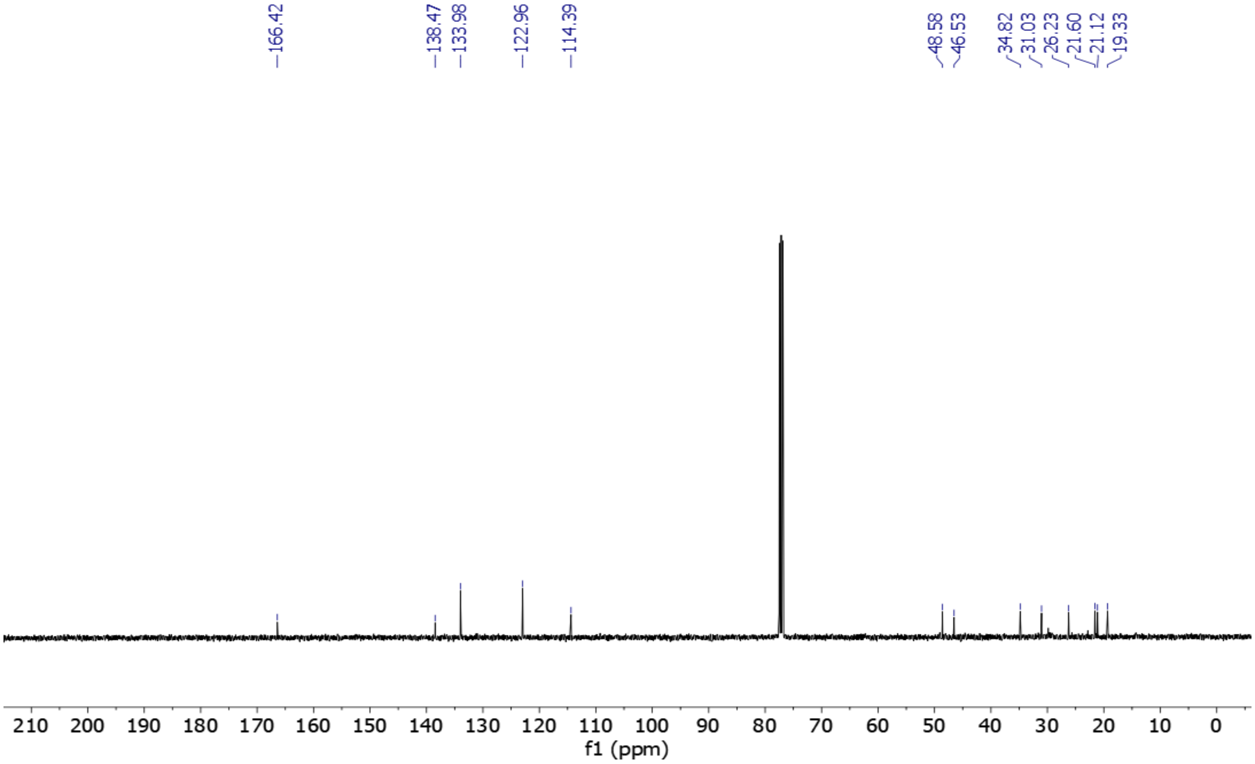

^{1}H NMR (400 MHz, $CDCl_3$) of *Trans* 2-(-2-acetyl-3-(2,6,6-trimethylcyclohex-2-en-1-yl)aziridin-1-yl)isoindoline-1,3dione(2aw)

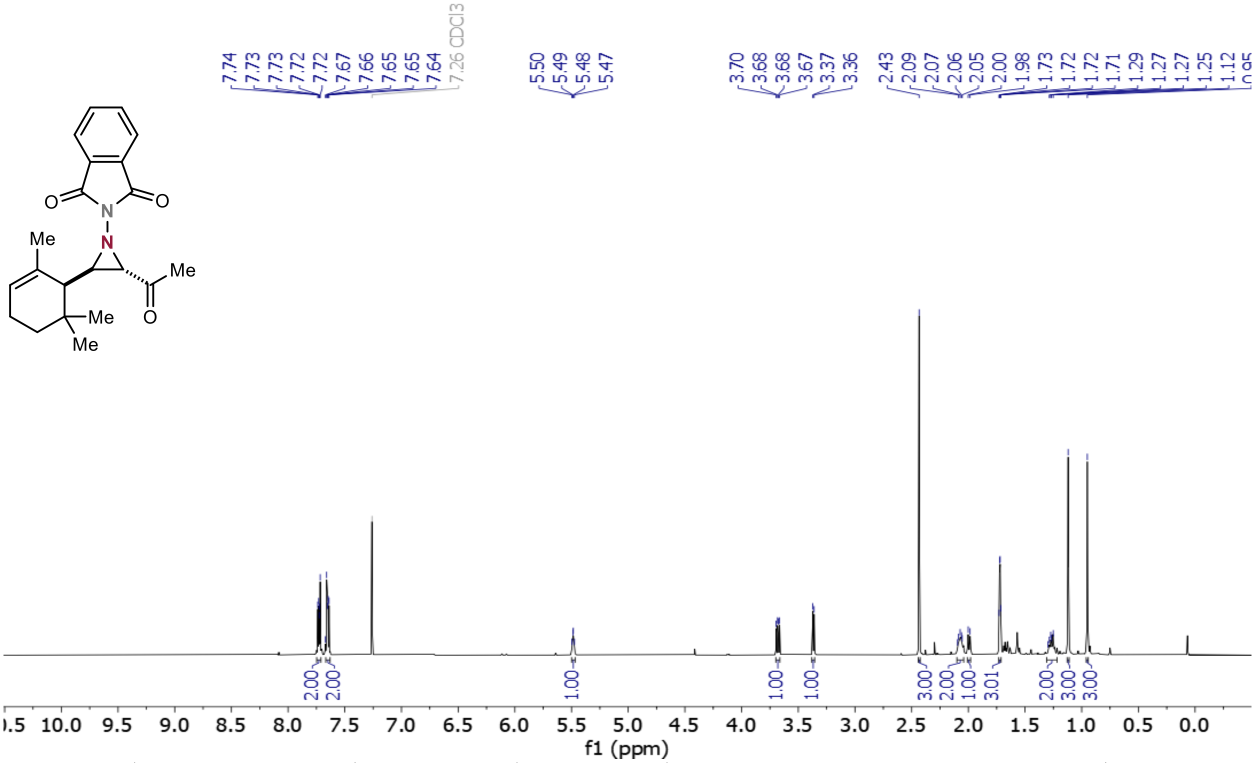

^{13}C NMR (101 MHz, $CDCl_3$) of *Trans* 2-(2-acetyl-3-(2,6,6-trimethylcyclohex-2-en-1-yl)aziridin-1-yl)isoindoline-1,3dione (2aw)

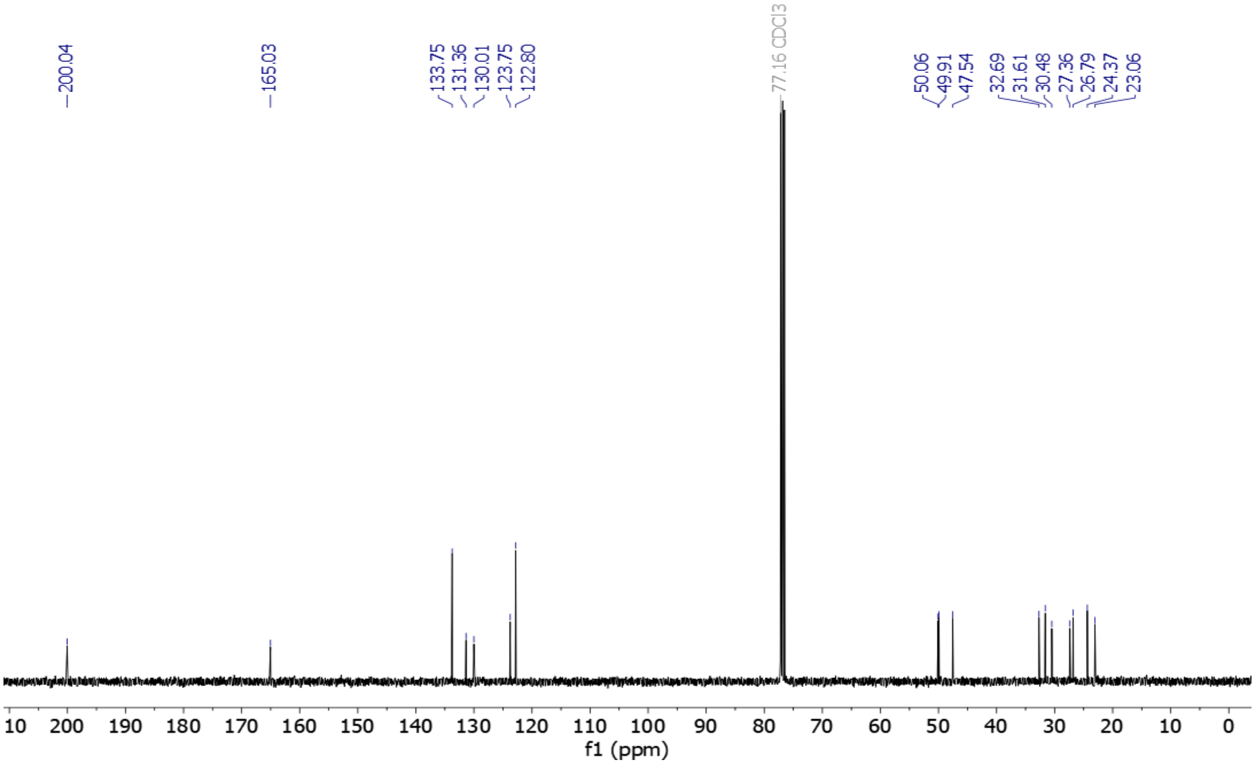

^{1}H NMR (500 MHz, $CDCl_3$) of 5-(1-(1,3-dioxoisoindolin-2-yl)-3,3-dimethylaziridin-2-yl)-3-methylpent-1-en-3-yl acetate (2ax)

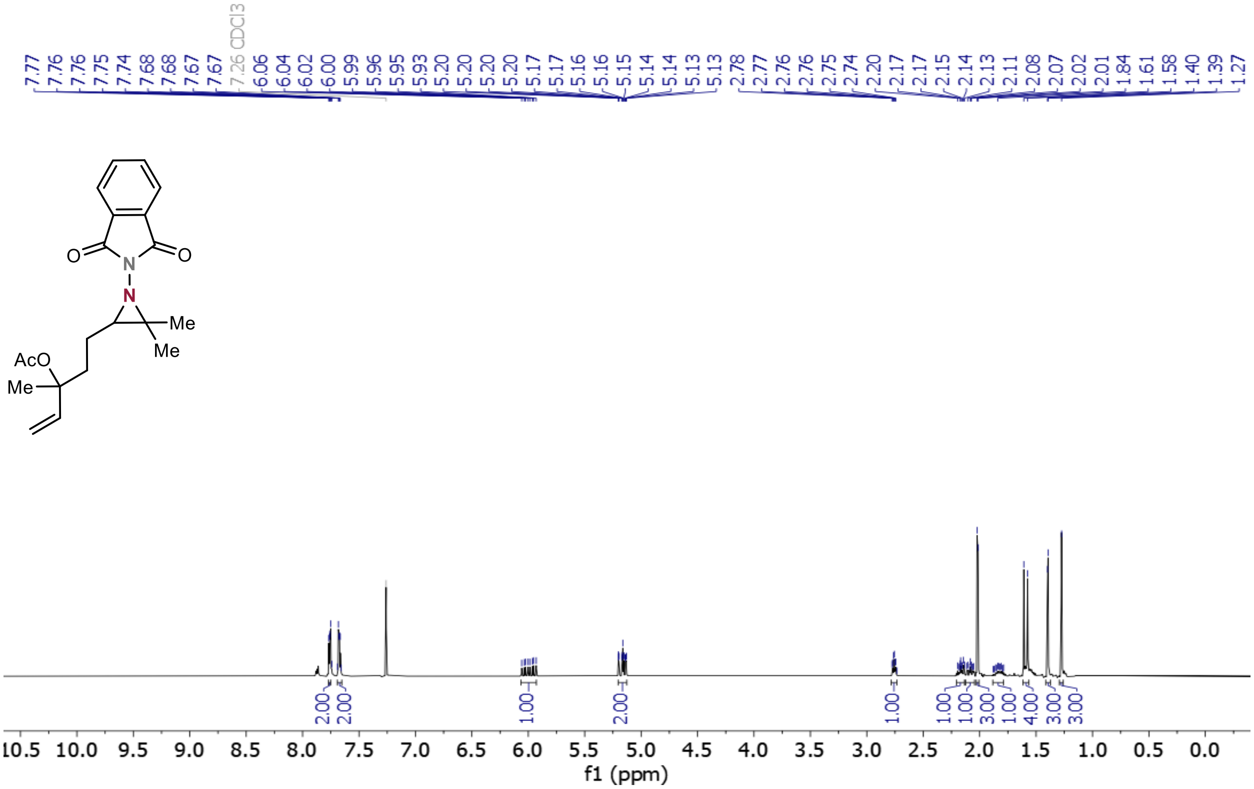

^{13}C NMR (101 MHz, $CDCl_3$) of 5-(1-(1,3-dioxoisoindolin-2-yl)-3,3-dimethylaziridin-2-yl)-3-methylpent-1-en-3-yl acetate (2ax)

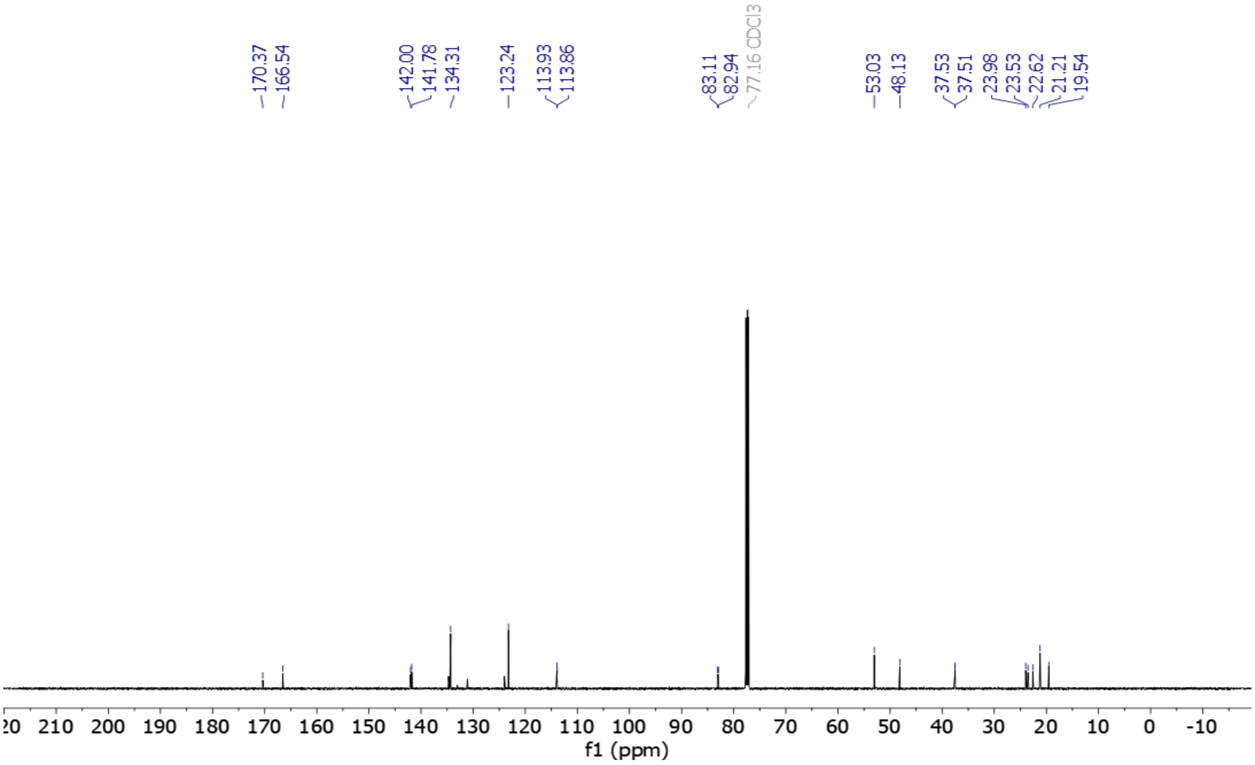

^{1}H NMR (500 MHz, CDCl$_3$) of methyl 8-(1-(1,3-dioxoisoindolin-2-yl)-3-octylaziridin-2-yl)octanoate (2ay)

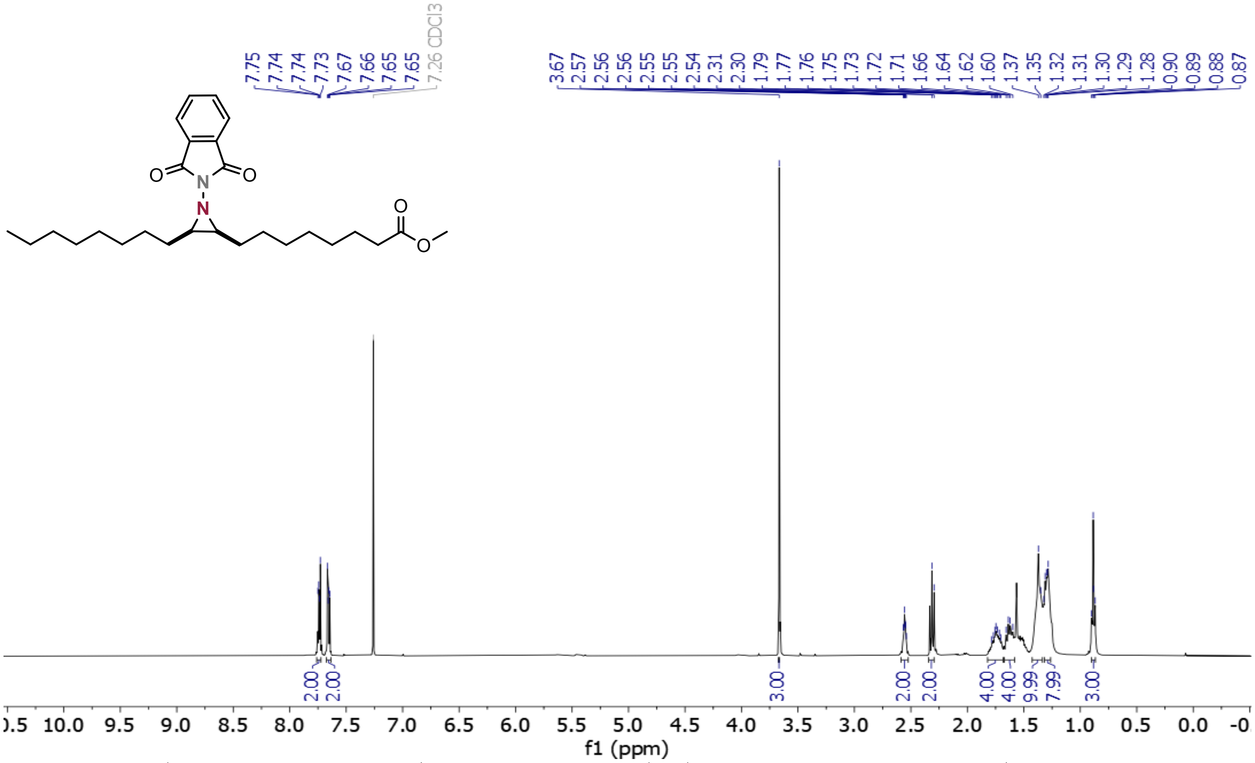

^{13}C NMR (126 MHz, CDCl$_3$) of methyl 8-(1-(1,3-dioxoisoindolin-2-yl)-3-octylaziridin-2-yl)octanoate (2ay)

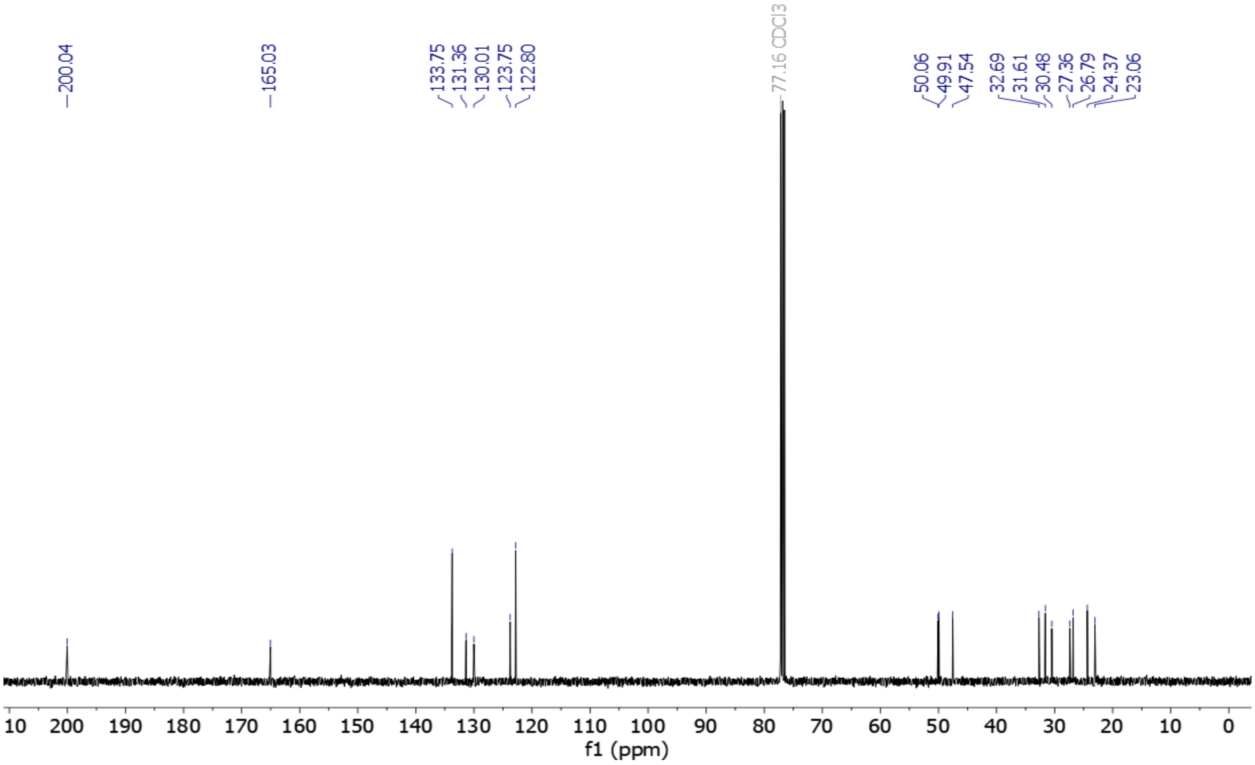

^{1}H NMR (500 MHz, $CDCl_3$) of *tert*-butyl 1-(1,3-dioxoisoindolin-2-yl)aziridine-2carboxylate (2az)

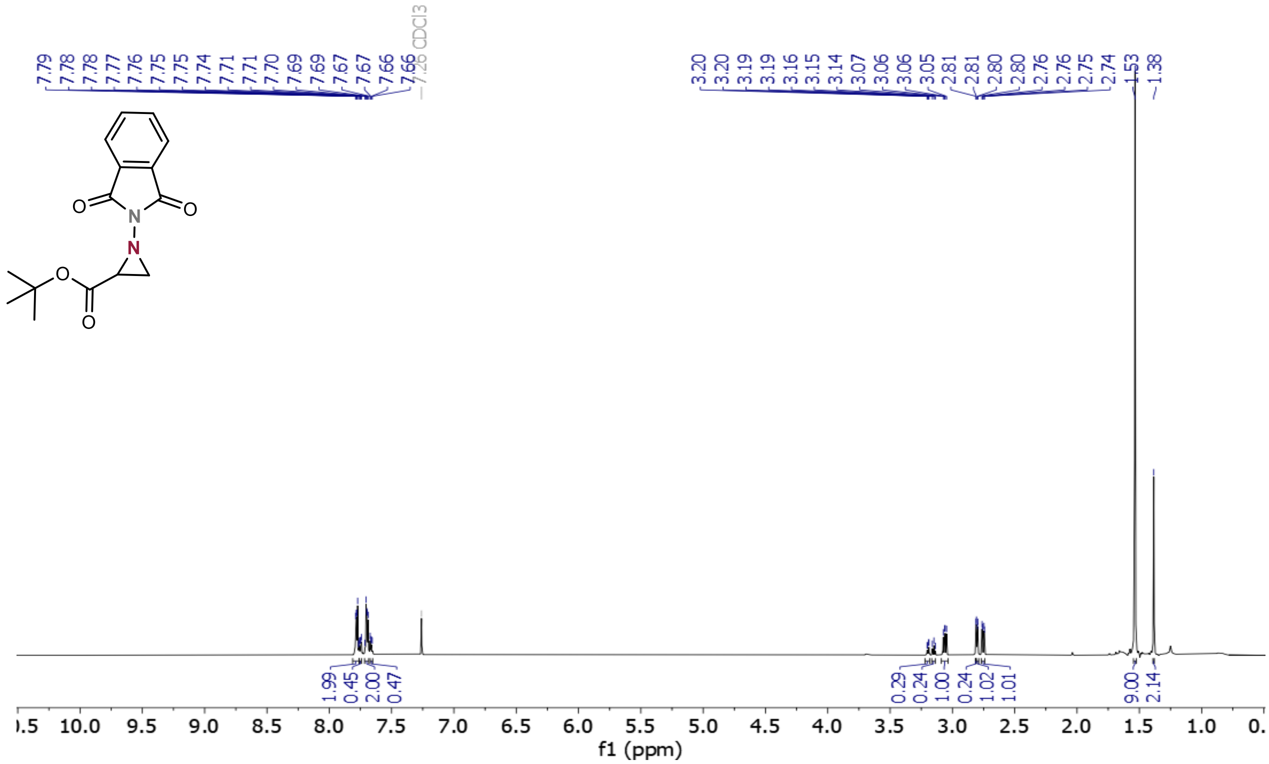

^{13}C NMR (126 MHz, $CDCl_3$) of *tert*-butyl 1-(1,3-dioxoisoindolin-2-yl)aziridine-2carboxylate (2az)

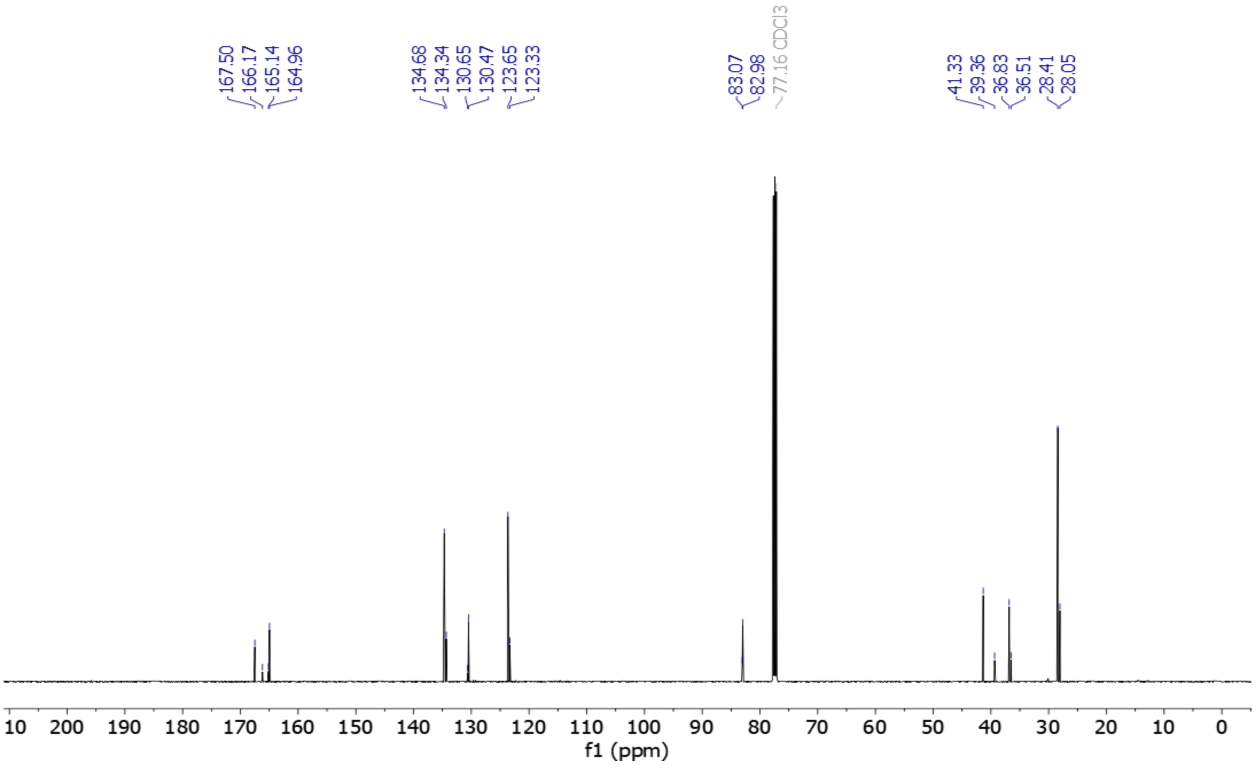

^{1}H NMR (400 MHz, $CDCl_3$) of 2-(2-(phenylsulfonyl)aziridin-1-yl)isoindoline-1,3-dione (2ba)

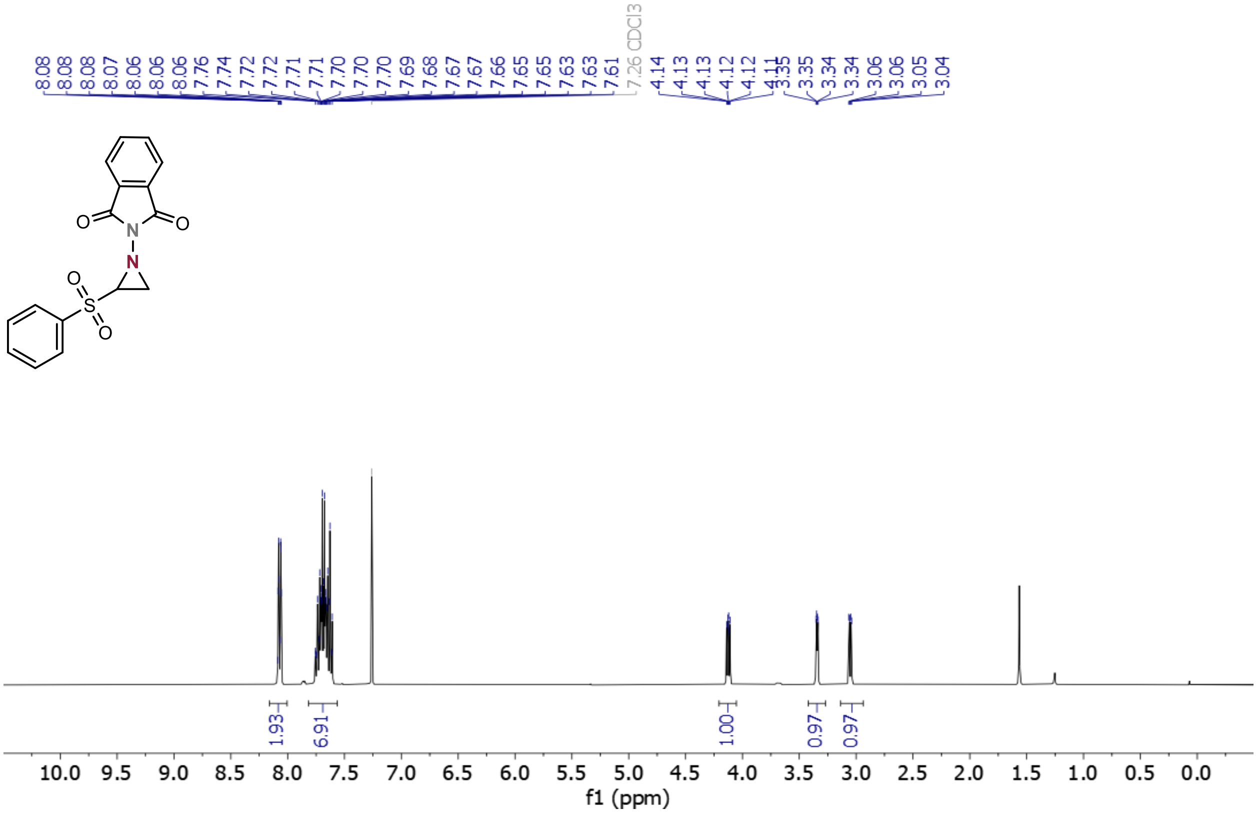

^{13}C NMR (101 MHz, $CDCl_3$) of 2-(2-(phenylsulfonyl)aziridin-1-yl)isoindoline-1,3-dione (2ba)

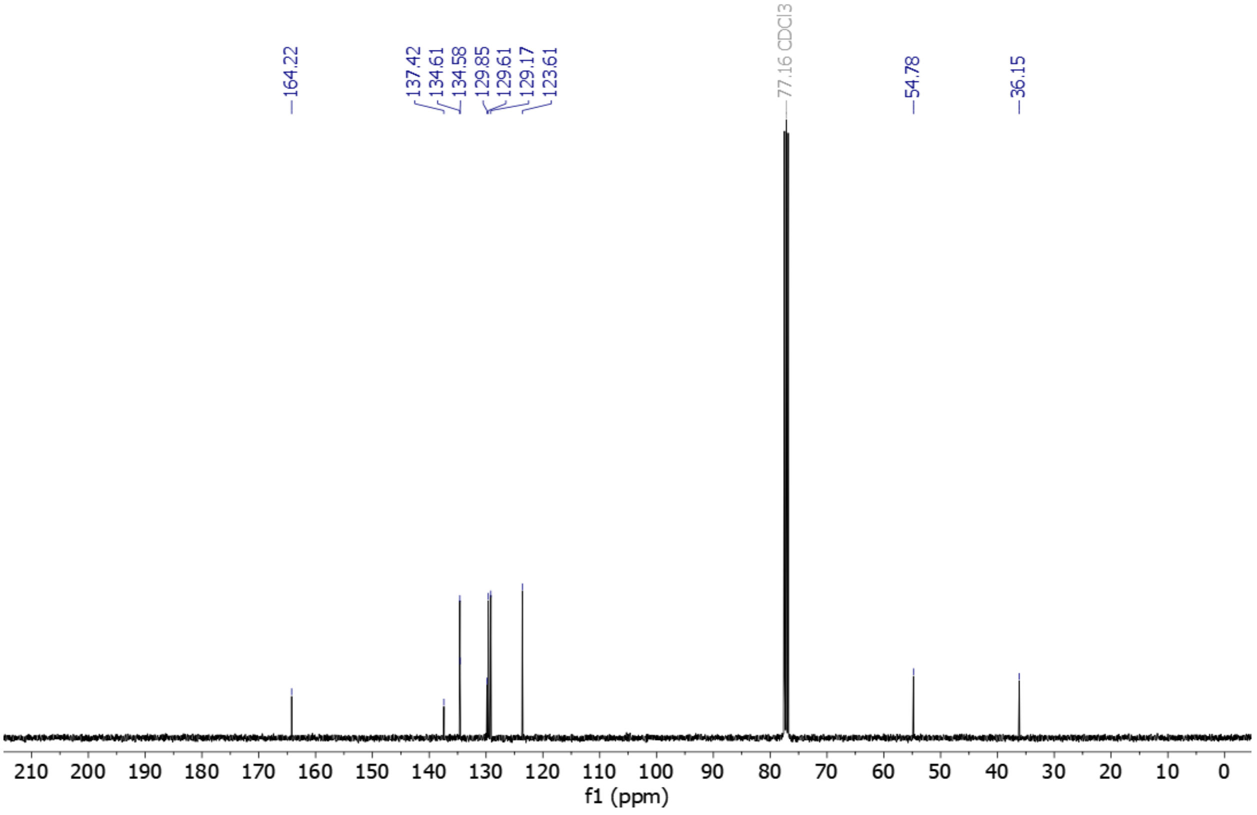

^{1}H NMR (500 MHz, CD_2Cl_2) of diethyl (1-(1,3-dioxoisoindolin-2-yl)aziridin-2-yl)phosphonate (2bb)

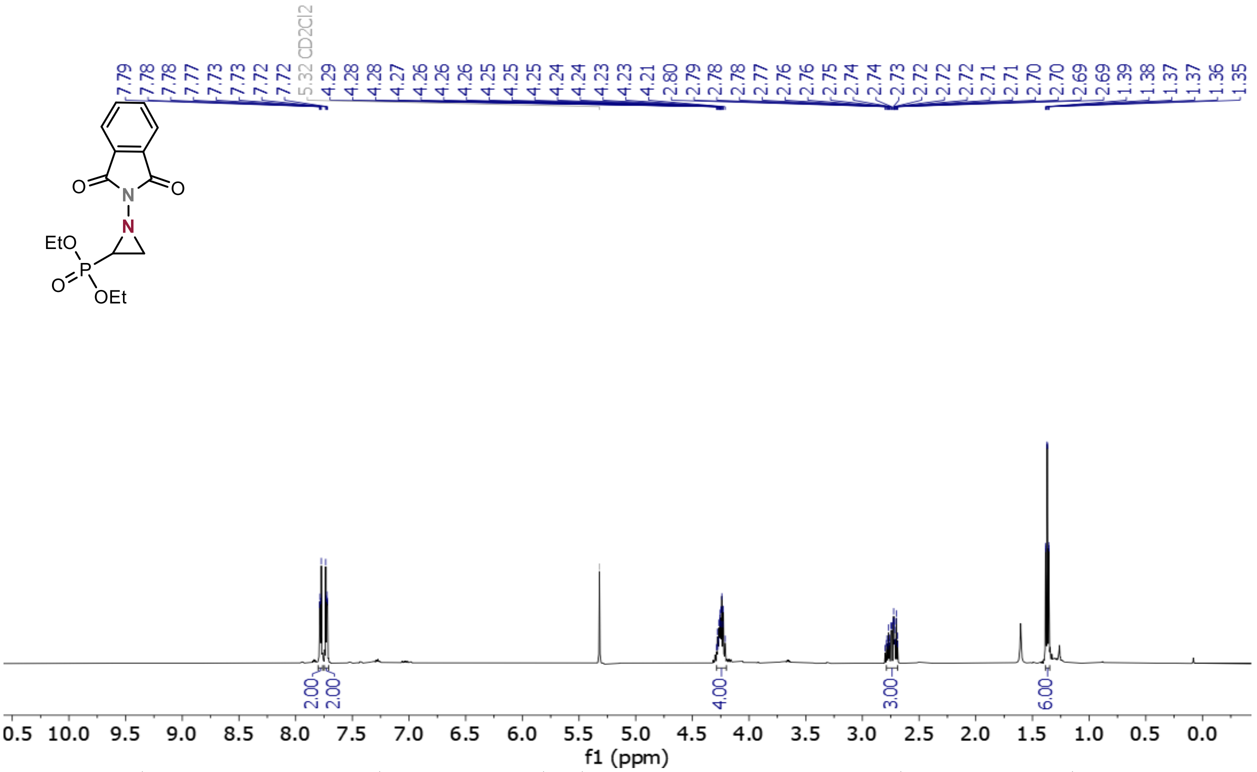

^{13}C NMR (126 MHz, CD_2Cl_2) of diethyl (1-(1,3-dioxoisoindolin-2-yl)aziridin-2-yl)phosphonate (2bb)

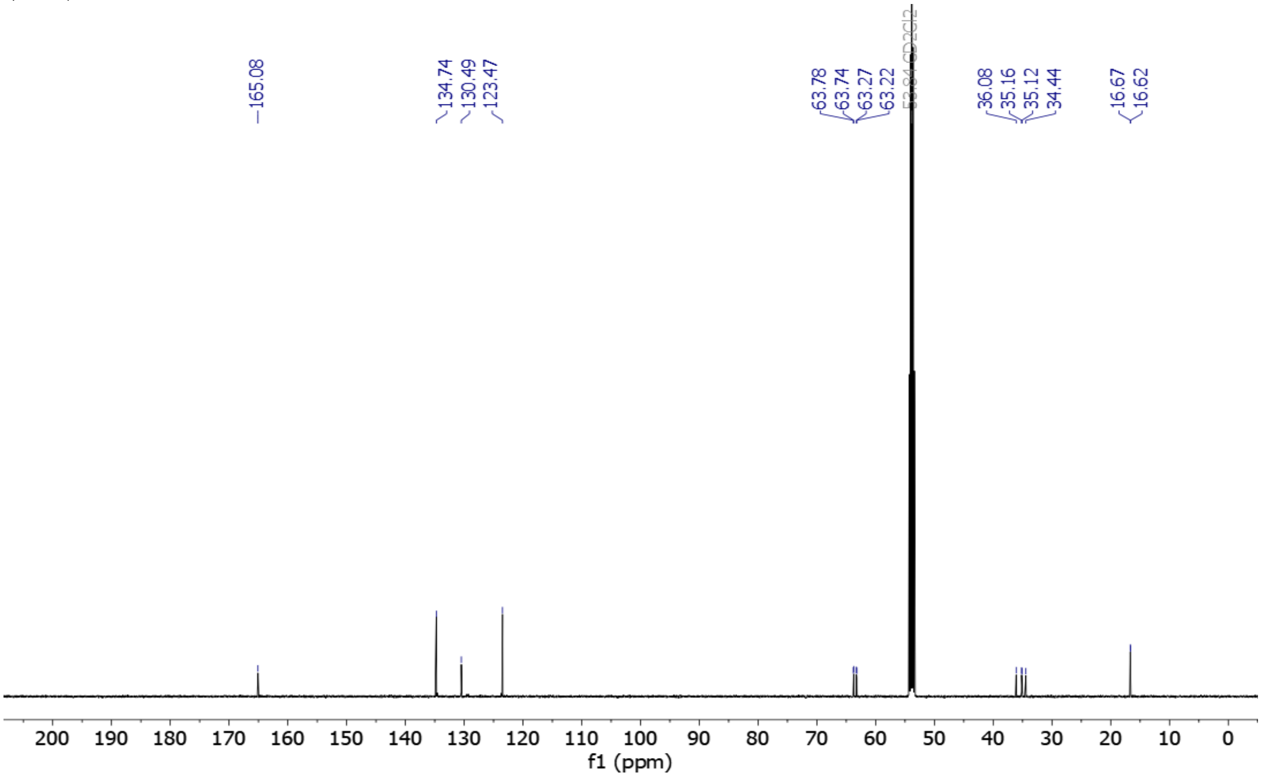

^{1}H NMR (400 MHz, CDCl$_3$) of 2-((3*S*,4a*R*,5a*S*,6a*S*,6b*S*,9*R*,9a*R*,11a*S*,11b*R*)-3-hydroxy-9a,11b-dimethyl-9-((*R*)-5-methylhexan-2-yl)hexadecahydro-5*H*-cyclopenta[1,2]phenanthro[8a,9-*b*]azirin-5-yl)isoindoline-1,3-dione (2bc)

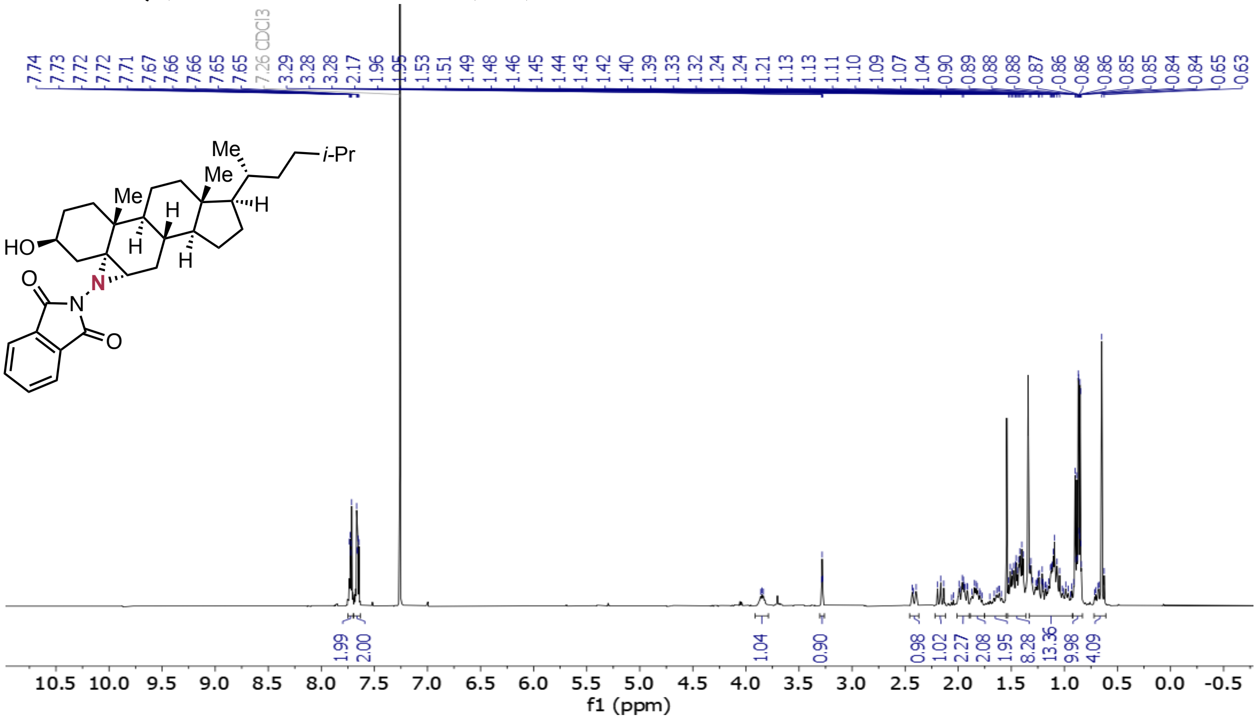

^{13}C NMR (101 MHz, CDCl$_3$) of 2-((3*S*,4a*R*,5a*S*,6a*S*,6b*S*,9*R*,9a*R*,11a*S*,11b*R*)-3-hydroxy-9a,11b-dimethyl-9-((*R*)-5-methylhexan-2-yl)hexadecahydro-5*H*-cyclopenta[1,2]phenanthro[8a,9-*b*]azirin-5-yl)isoindoline-1,3-dione (2bc)

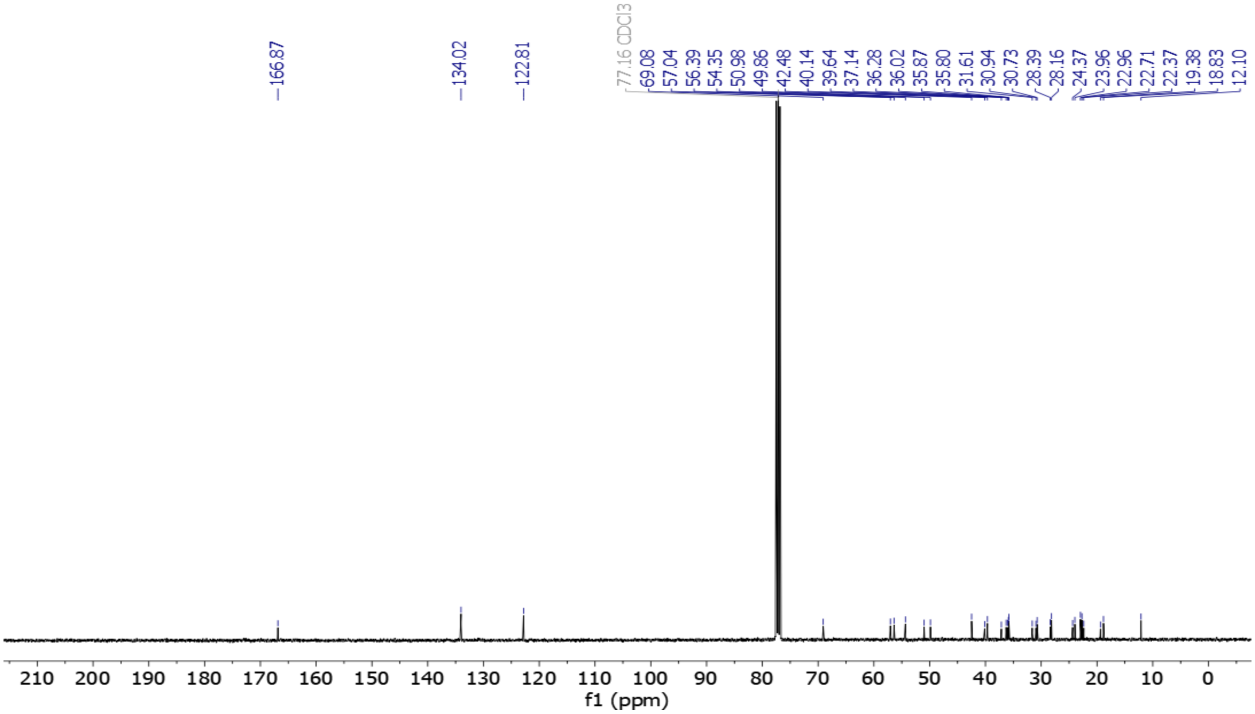

^{1}H NMR (500 MHz, $CDCl_3$) of 1-phenyl-2-phthalimidodiazene 1-oxide (AT1)

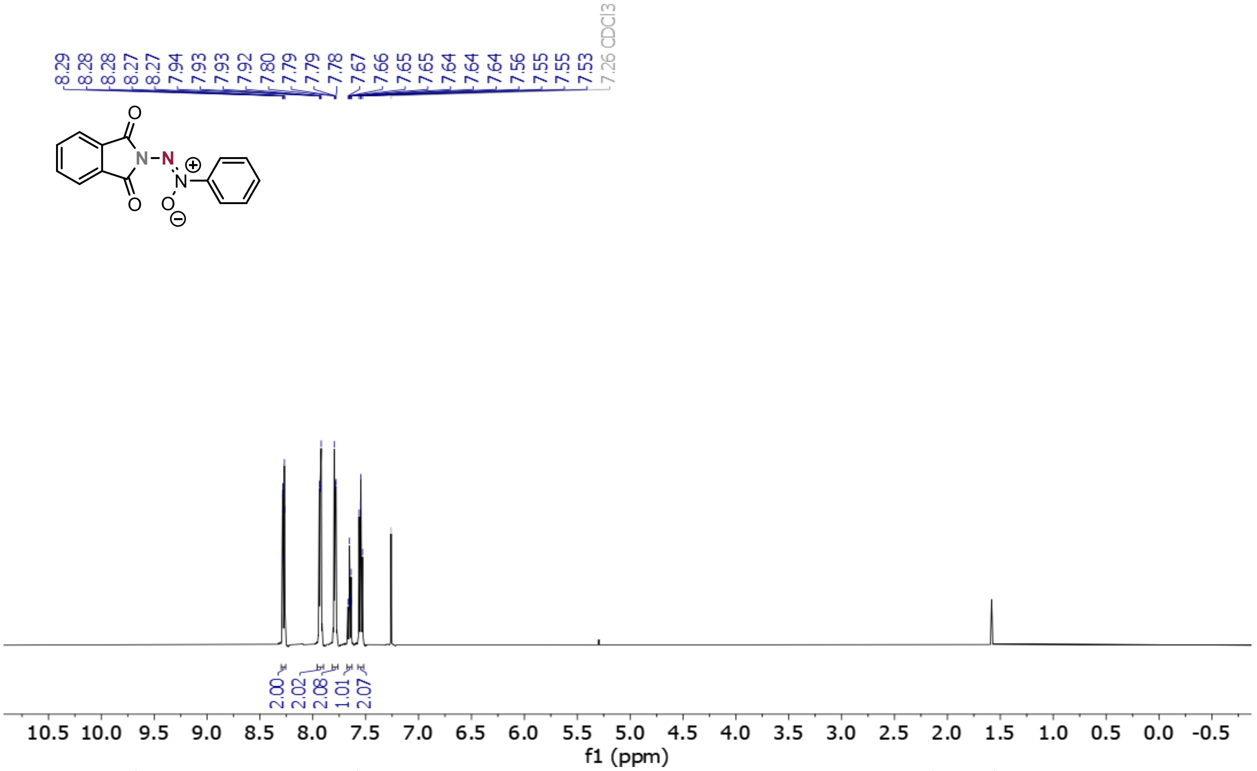

^{13}C NMR (126 MHz, $CDCl_3$) of 1-phenyl-2-phthalimidodiazene 1-oxide (AT1)

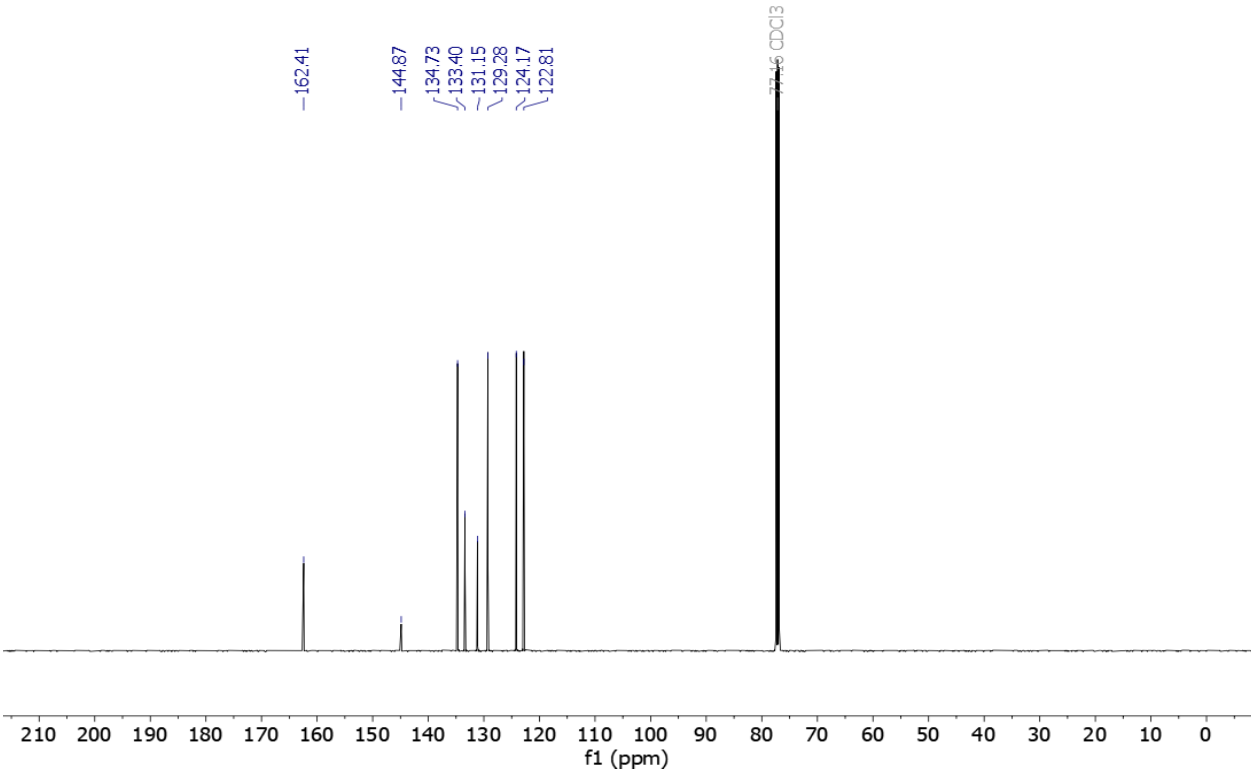

^{1}H NMR (500 MHz, $CDCl_3$) of 1-(4-cyanophenyl)-2-(1,3-dioxoisoindolin-2-yl)diazene 1-oxide (AT2)

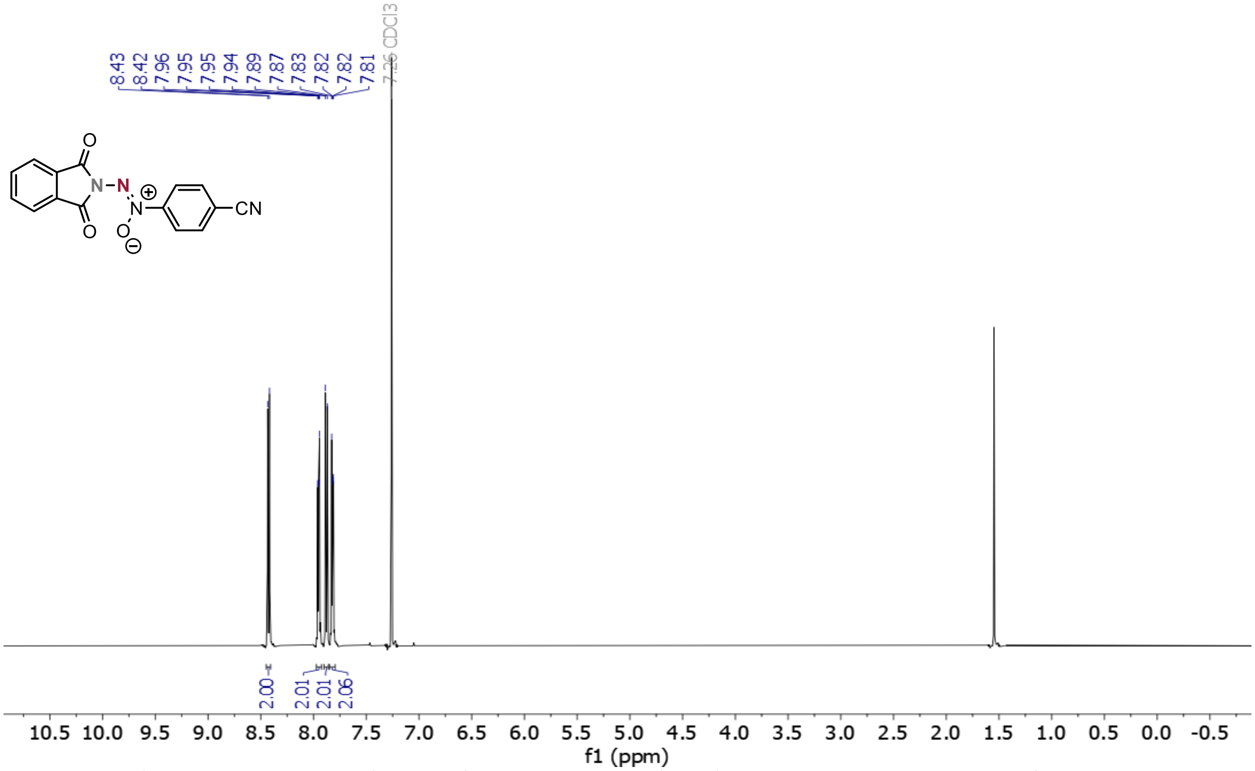

^{13}C NMR (126 MHz, $CDCl_3$) of 1-(4-cyanophenyl)-2-(1,3-dioxoisoindolin-2-yl)diazene 1-oxide (AT2)

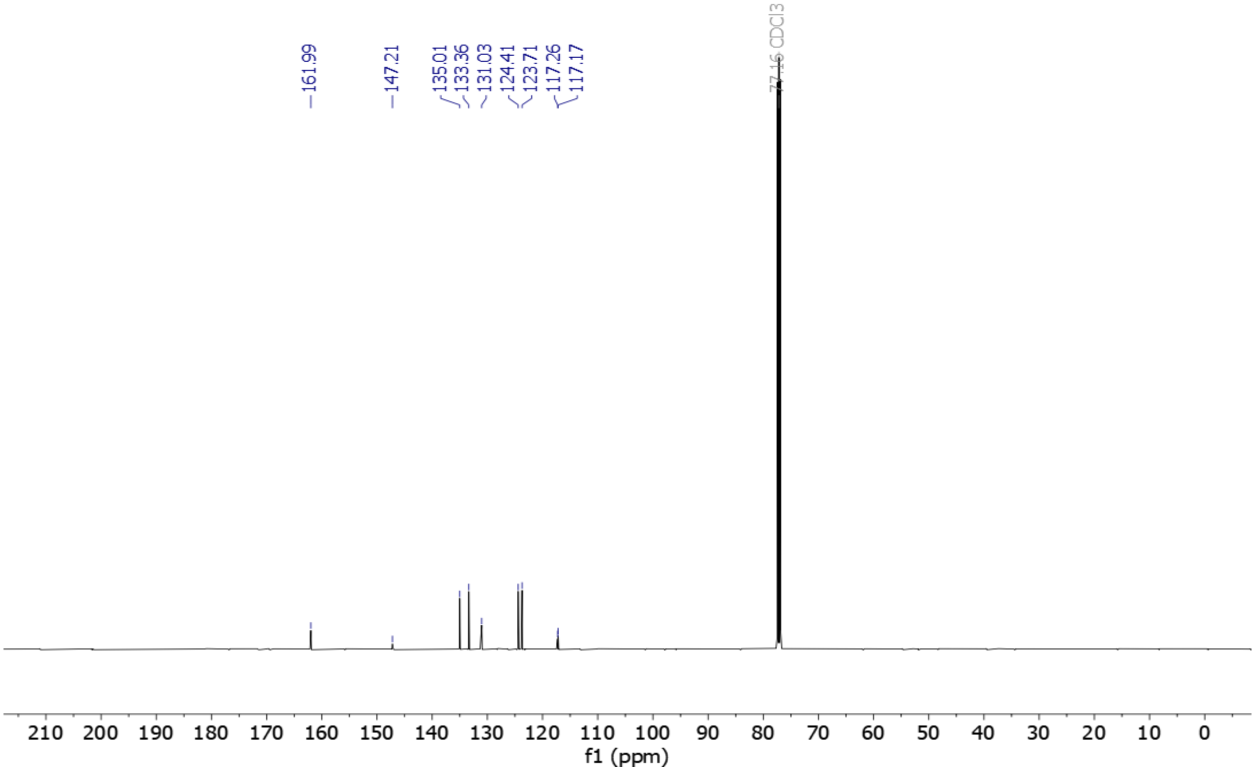

^{1}H NMR (500 MHz, $CDCl_3$) of 1-(4-(diethylamino)phenyl)-2-(1,3-dioxoisoindolin-2-yl)diazene 1-oxide (AT3)

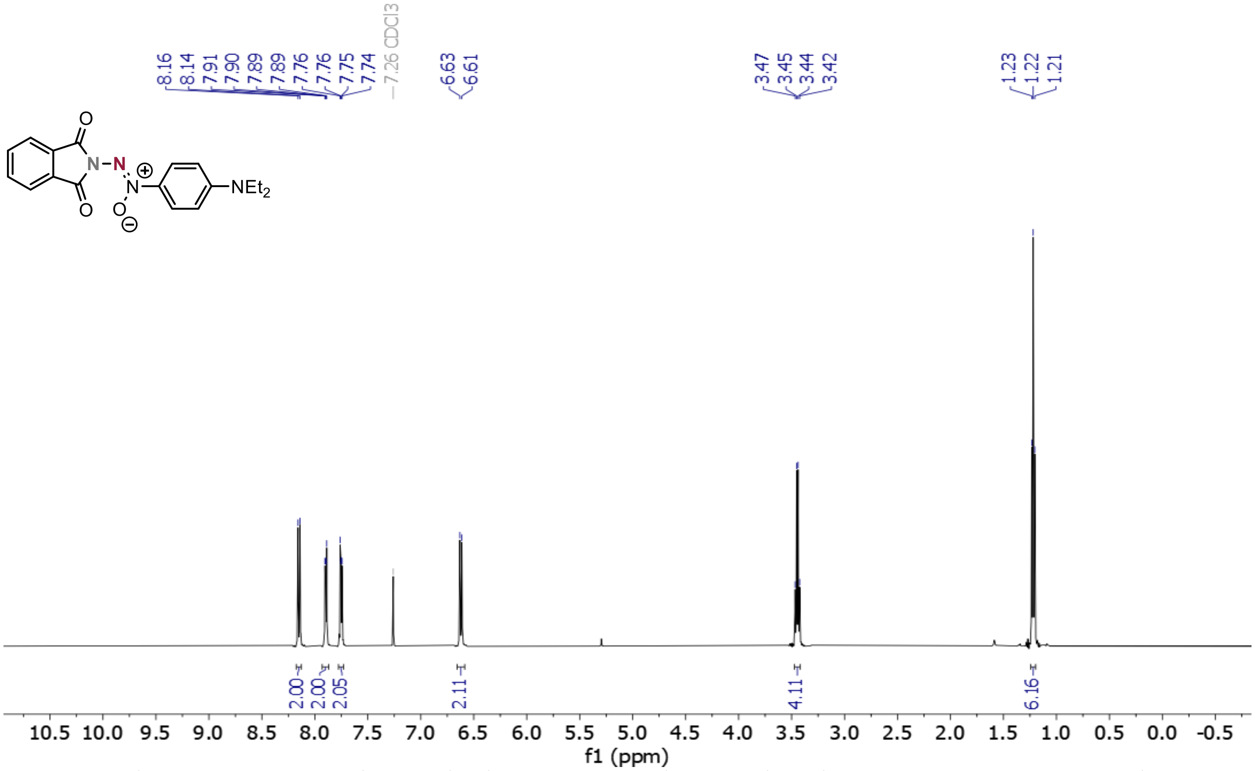

^{13}C NMR (126 MHz, $CDCl_3$) of 1-(4-(diethylamino)phenyl)-2-(1,3-dioxoisoindolin-2-yl)diazene 1-oxide (AT3)

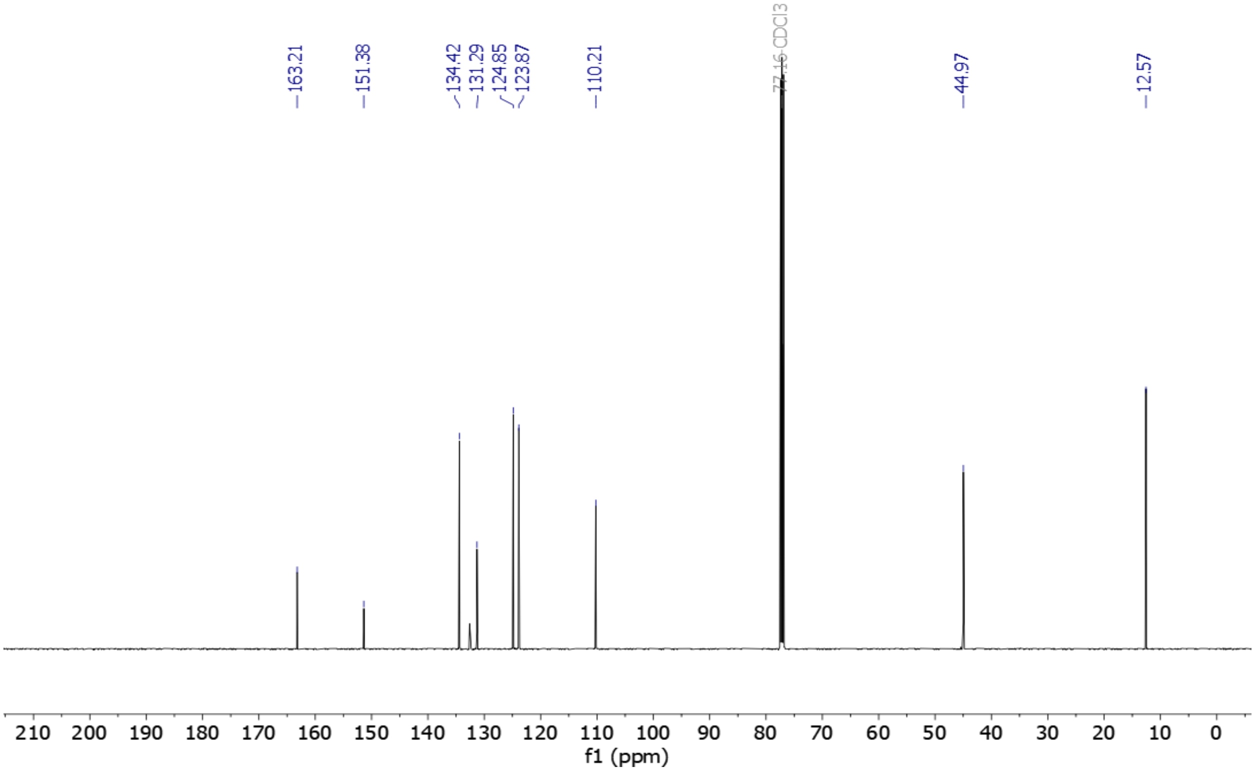

^{1}H NMR (500 MHz, $CDCl_3$) of 2-(1,3-dioxoisoindolin-2-yl)-1-(3 (trifluoromethyl)phenyl)diazene 1-oxide (AT4)

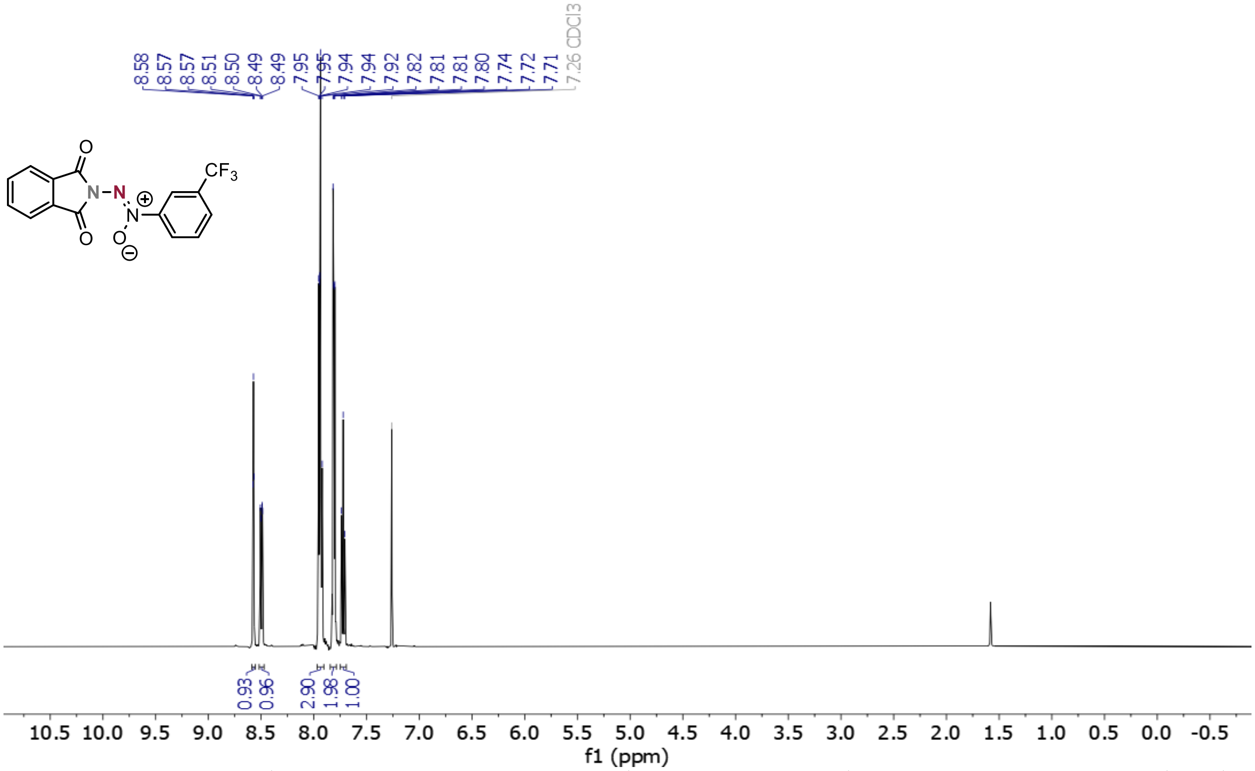

^{13}C NMR (126 MHz, $CDCl_3$) of 2-(1,3-dioxoisoindolin-2-yl)-1-(3-(trifluoromethyl)phenyl)diazene 1-oxide (AT4)

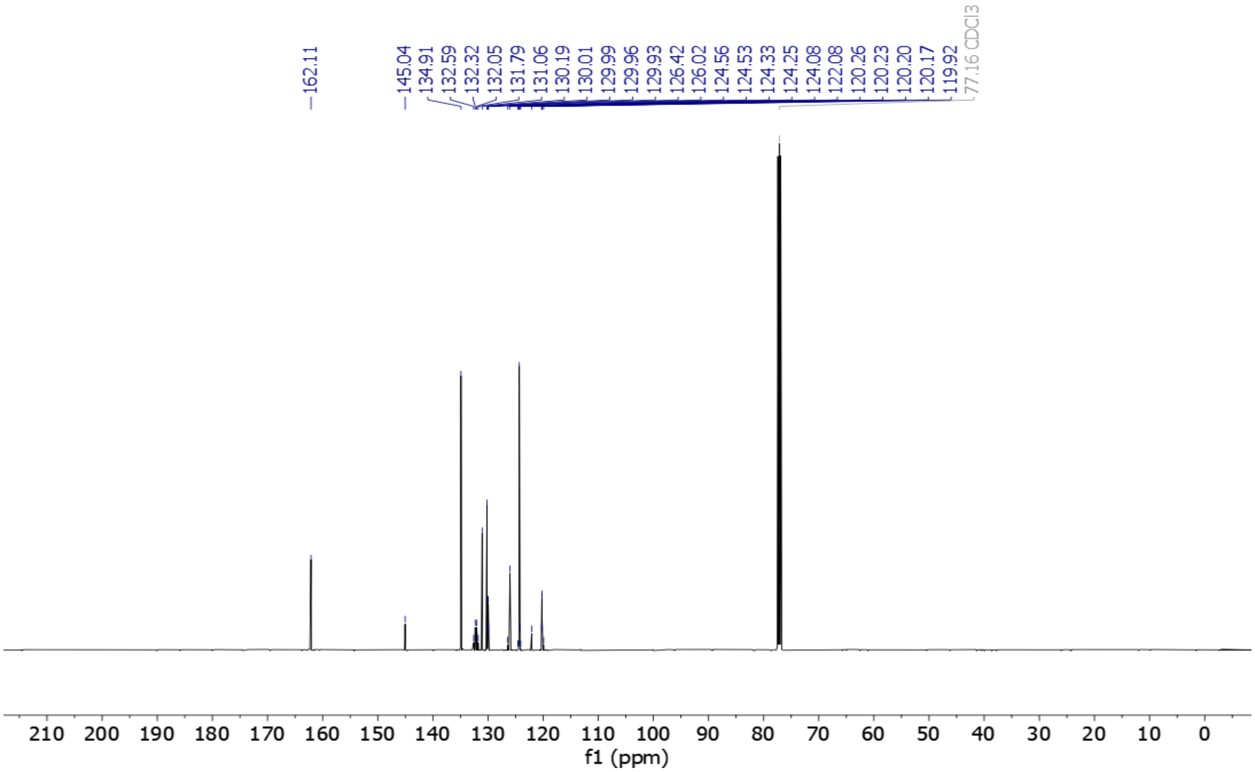

^{1}H NMR (500 MHz, $CDCl_3$) of 2-(1,3-dioxoisoindolin-2-yl)-1-(o-tolyl)diazene 1-oxide (AT5)

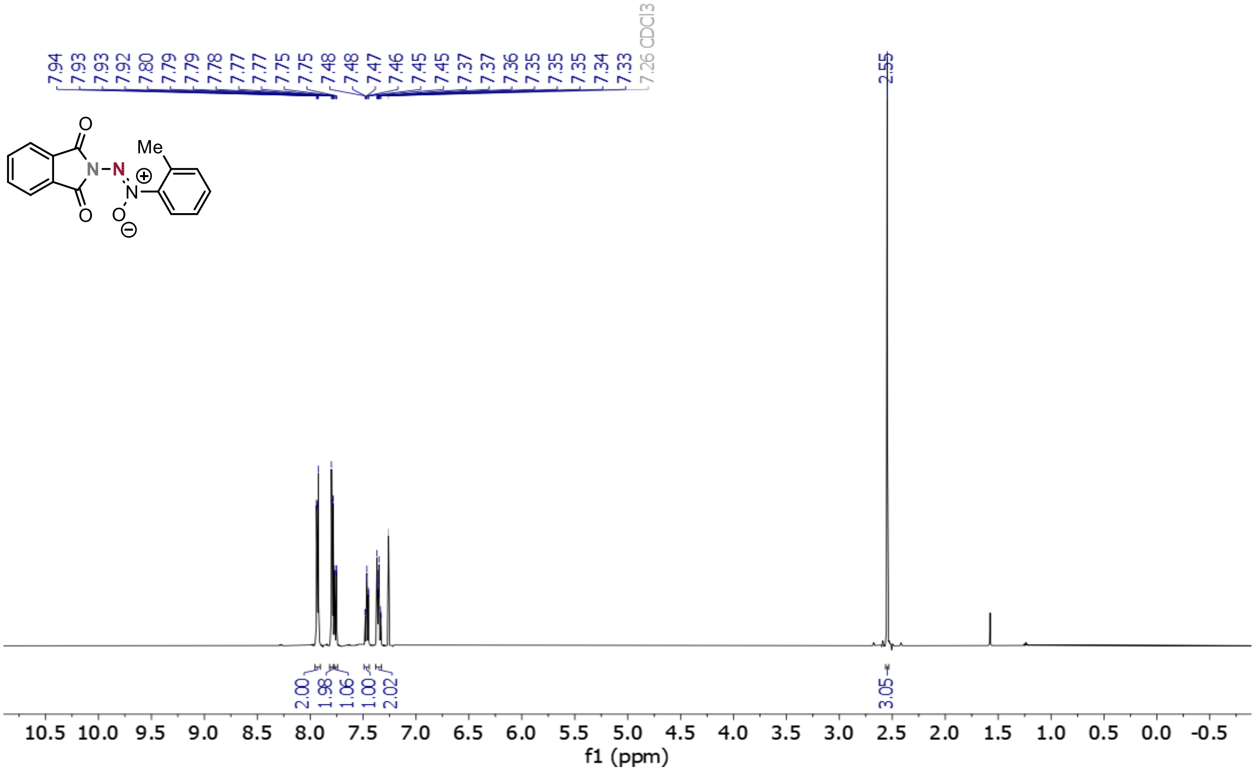

^{13}C NMR (126 MHz, $CDCl_3$) of 2-(1,3-dioxoisoindolin-2-yl)-1-(o-tolyl)diazene 1-oxide (AT5)

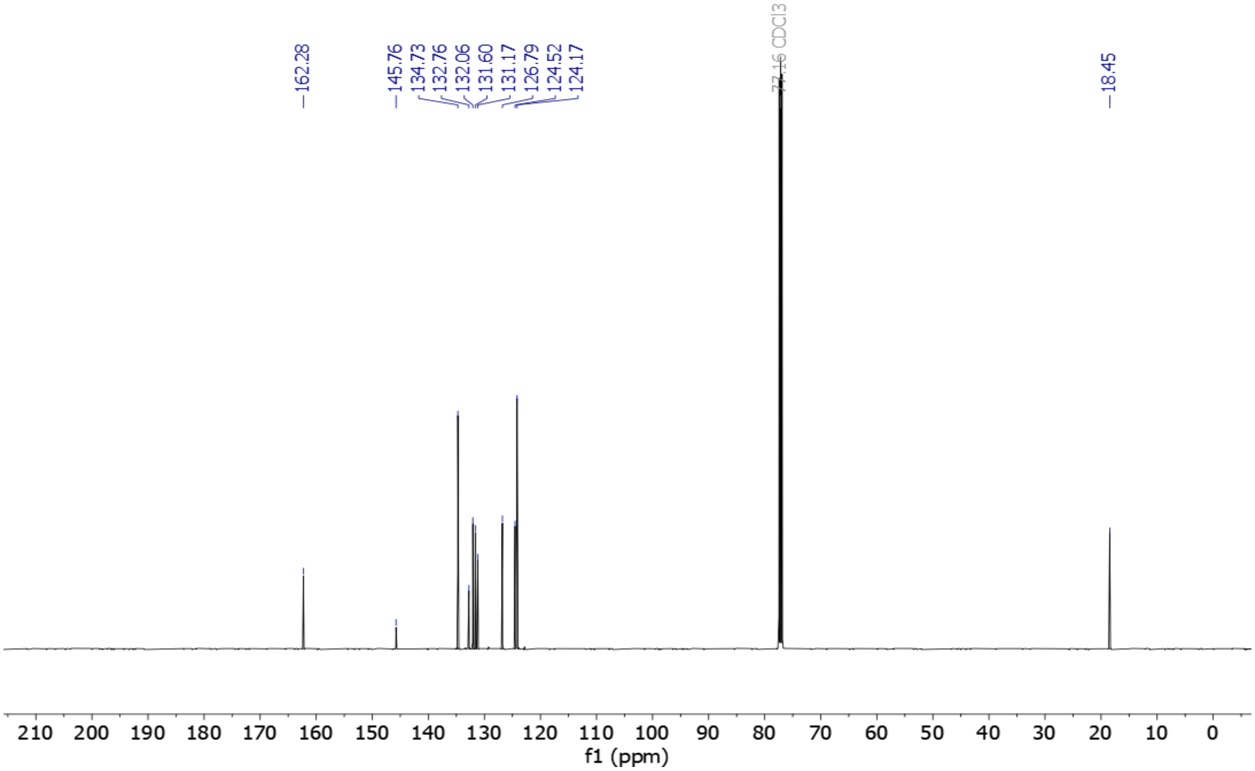

^{1}H NMR (600 MHz, $CDCl_3$) of *Cis* methyl 8-(3-octylaziridin-2-yl)octanoate (2ay1)

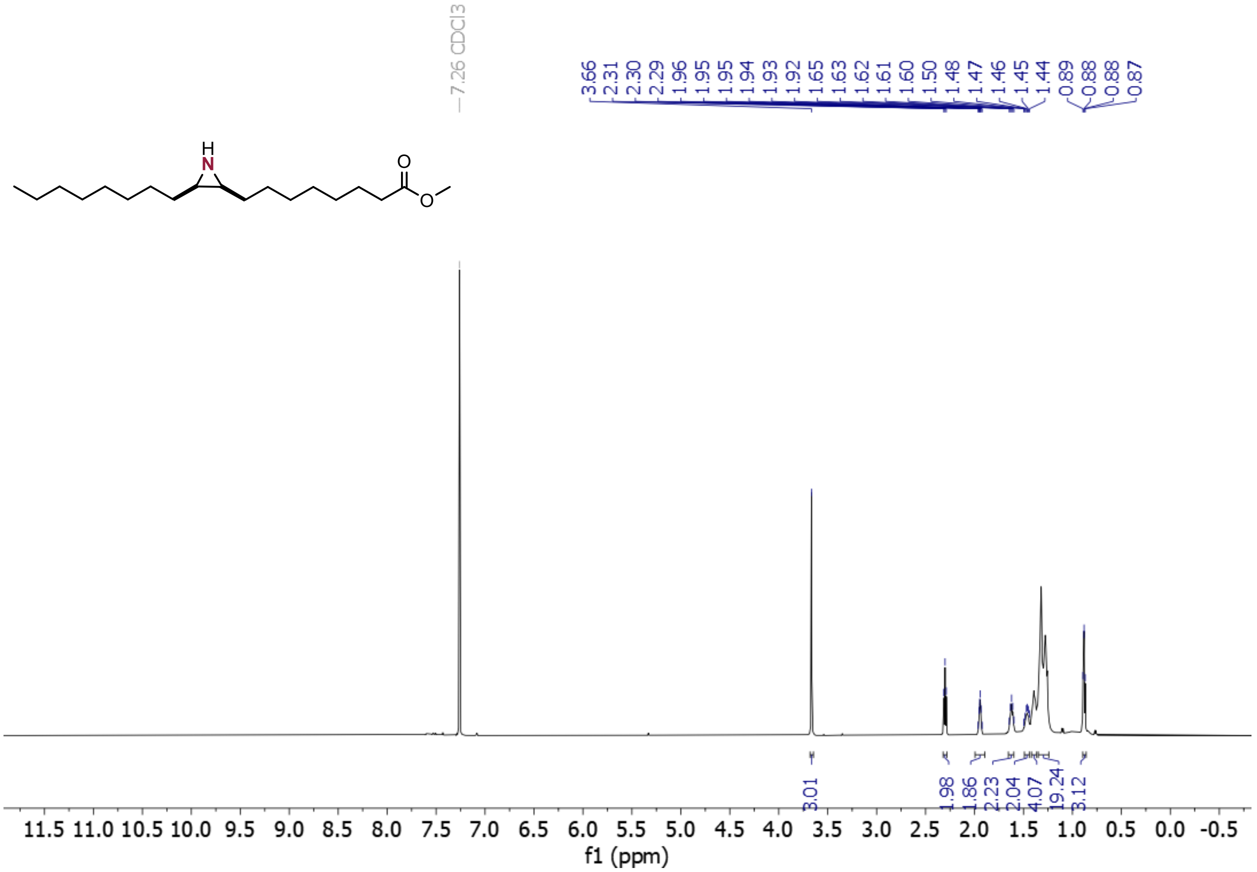

^{13}C NMR (151 MHz, $CDCl_3$) of *Cis* methyl 8-(3-octylaziridin-2-yl)octanoate (2ay1)

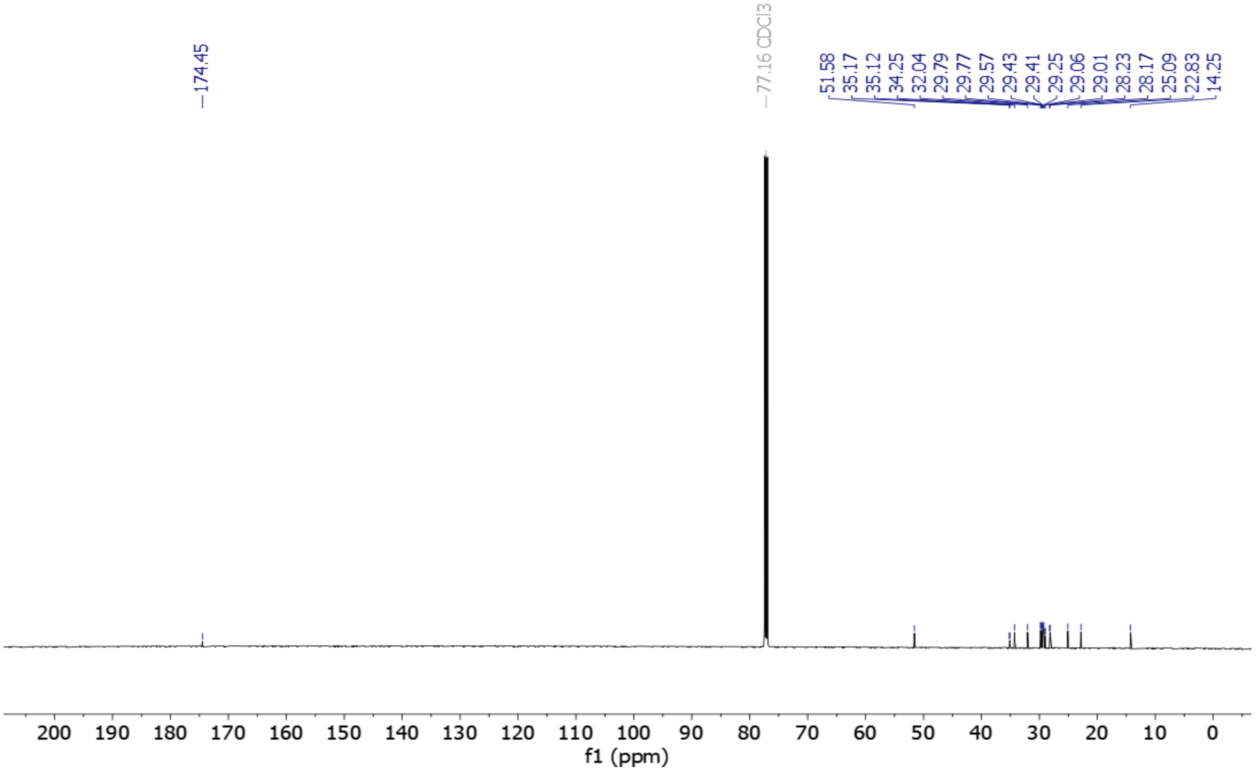

^{1}H NMR (500 MHz, $CDCl_3$) of *Cis* (9-azabicyclo[6.1.0]nonan-9-yl)(phenyl)methanone (2al2)

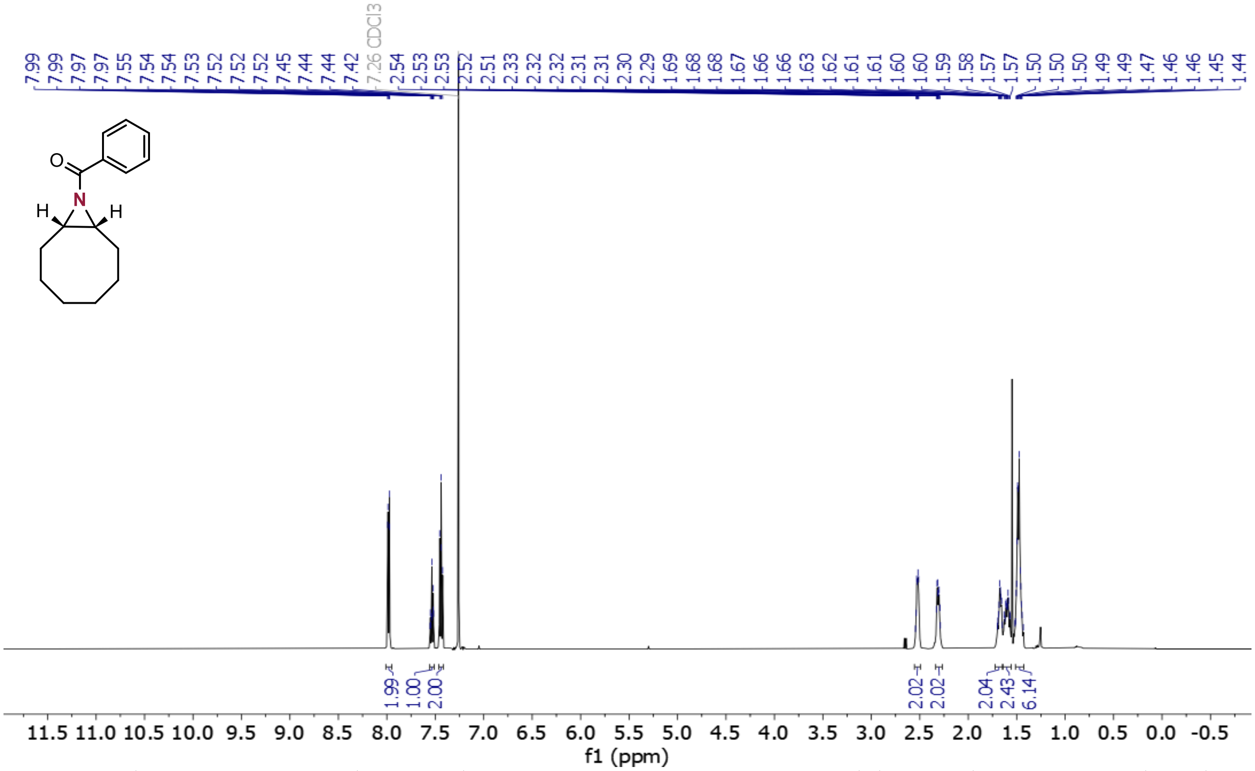

^{13}C NMR (126 MHz, $CDCl_3$) of Cis (9-azabicyclo[6.1.0]nonan-9-yl)(phenyl)methanone (2al2)

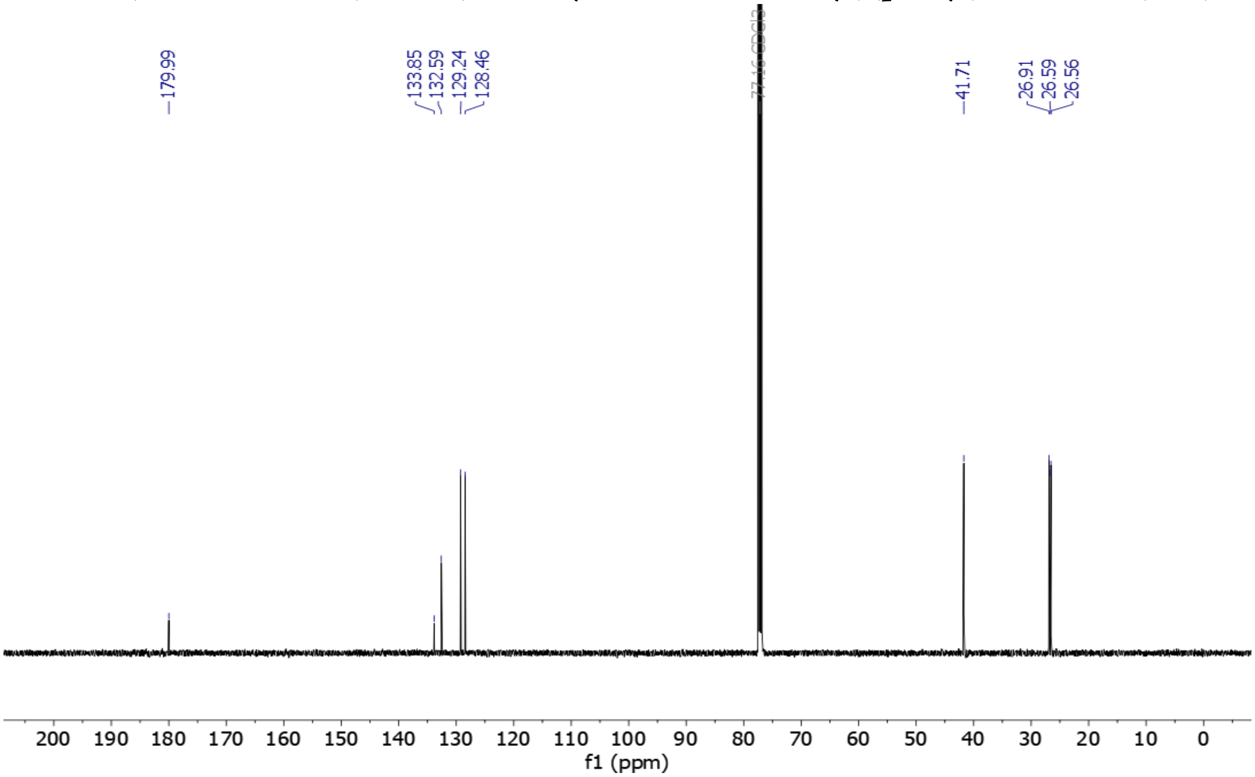

^{1}H NMR (500 MHz, $CDCl_3$) of *Cis* 2-methyl-3-phenylaziridin-1-amine (*Z*-6c1)

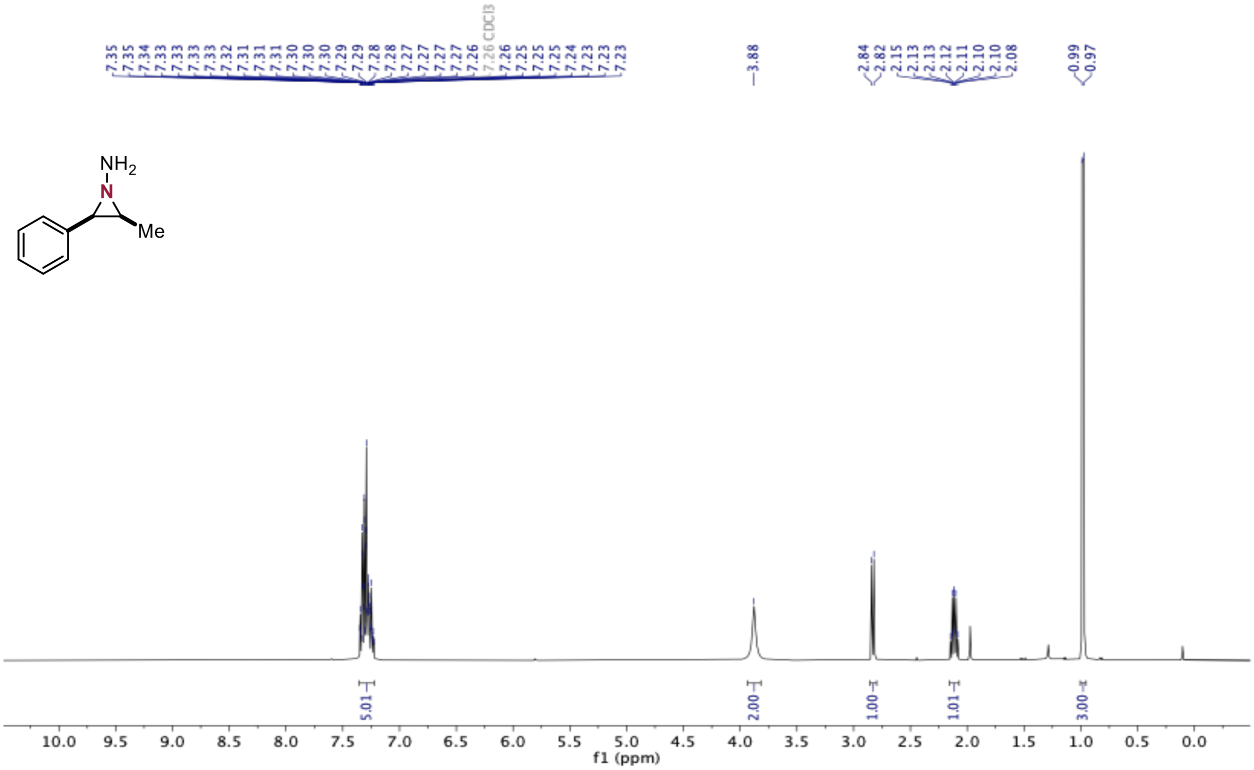

^{13}C NMR (126 MHz, $CDCl_3$) of *Cis* 2-methyl-3-phenylaziridin-1-amine (*Z*-6c1)

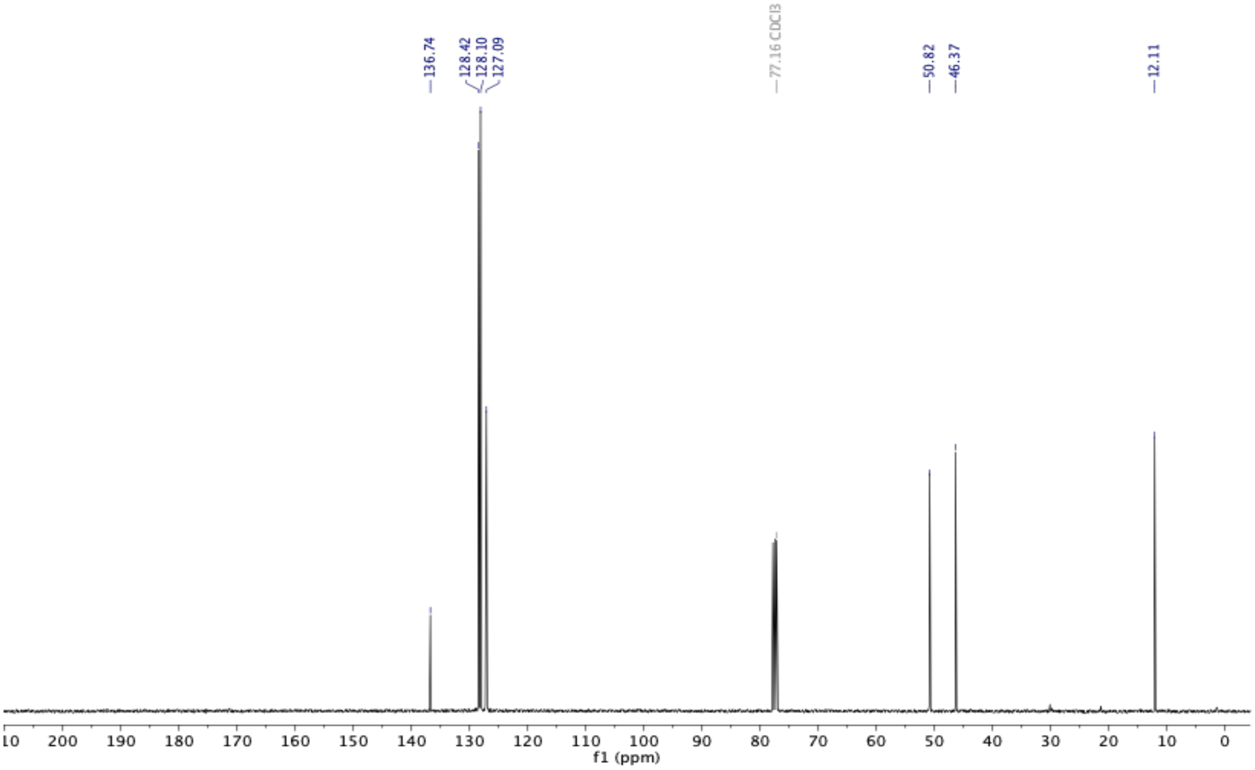

^{1}H NMR (400 MHz, CD_2Cl_2) of 2-((4-fluorophenethyl)amino)isoindoline-1,3-dione (2a1)

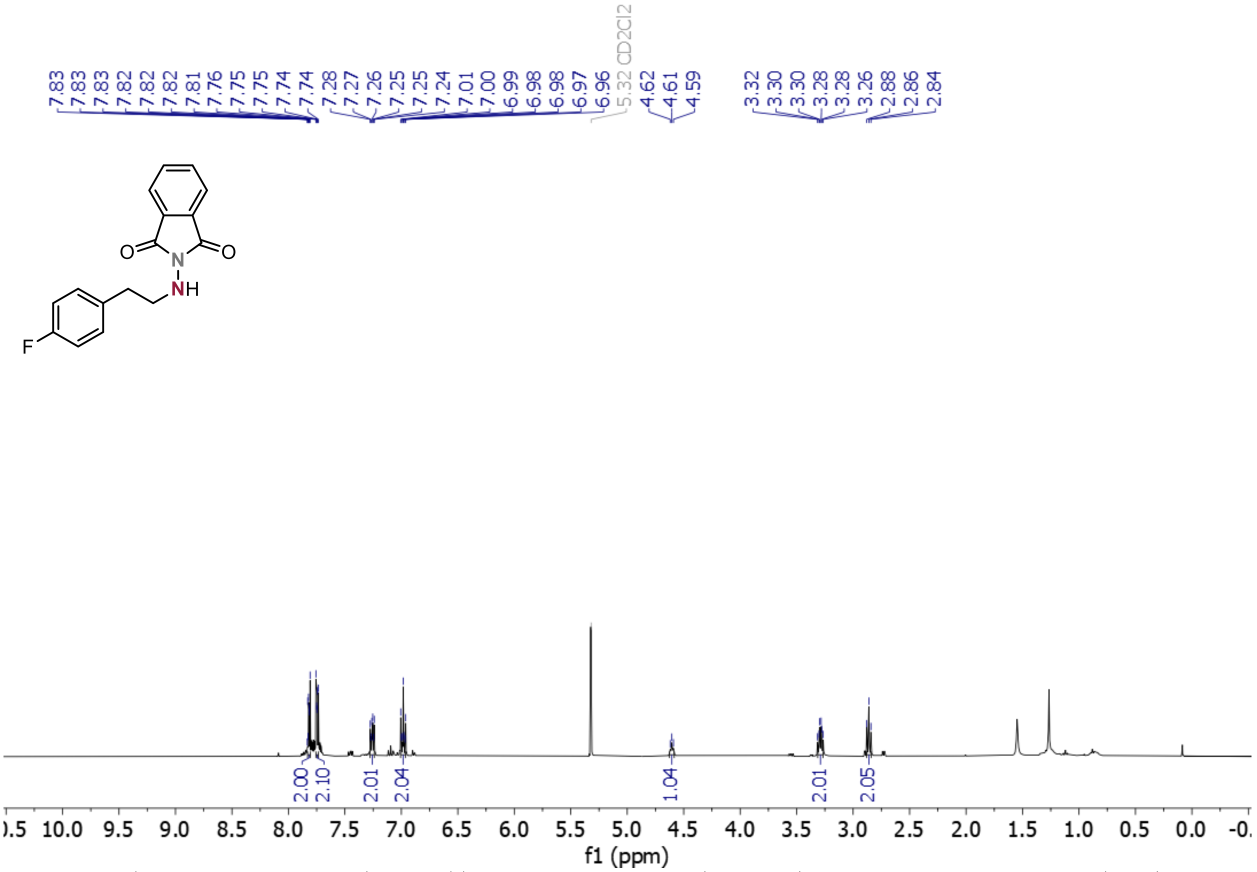

^{13}C NMR (101 MHz, CD_2Cl_2) of 2-((4-fluorophenethyl)amino)isoindoline-1,3-dione (2a1)

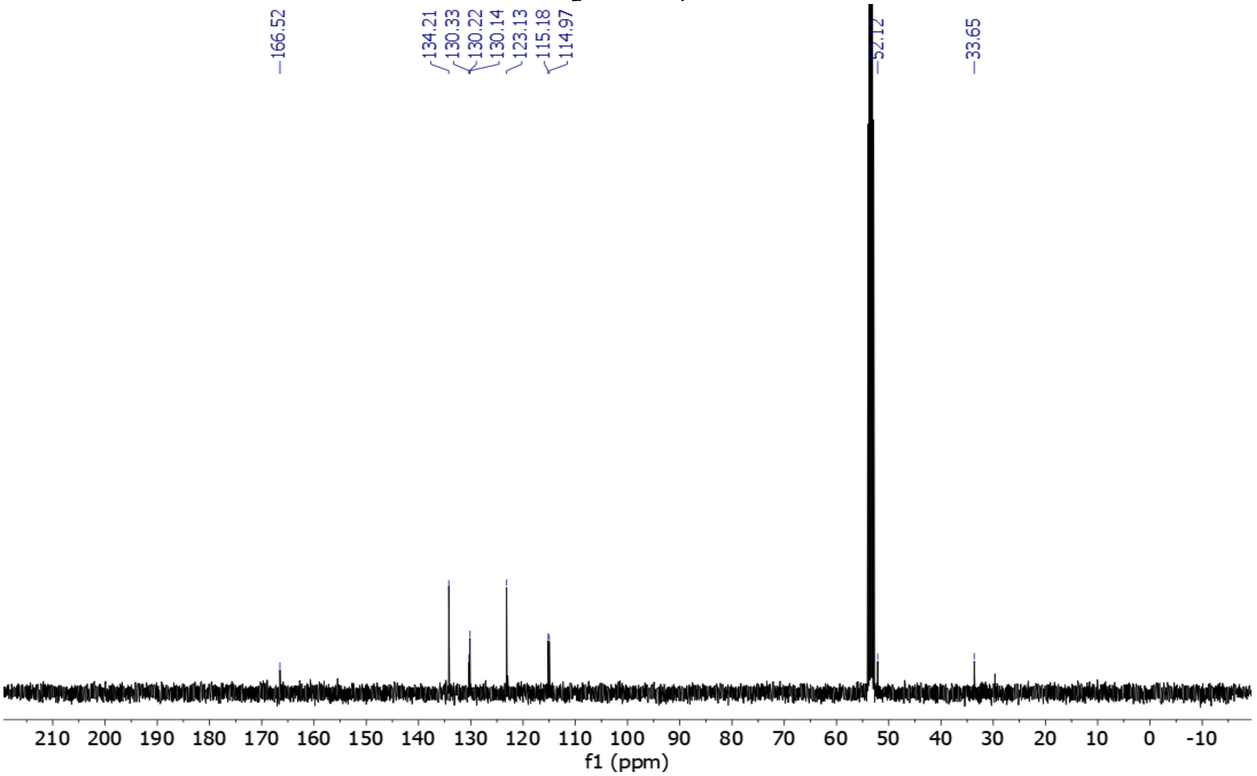

NMR Yields Spectra

^{1}H NMR (500 MHz, $CDCl_3$) of 2-(2-(4-hydroxyphenyl)aziridin-1-yl)isoindoline-1,3-dione (2i)

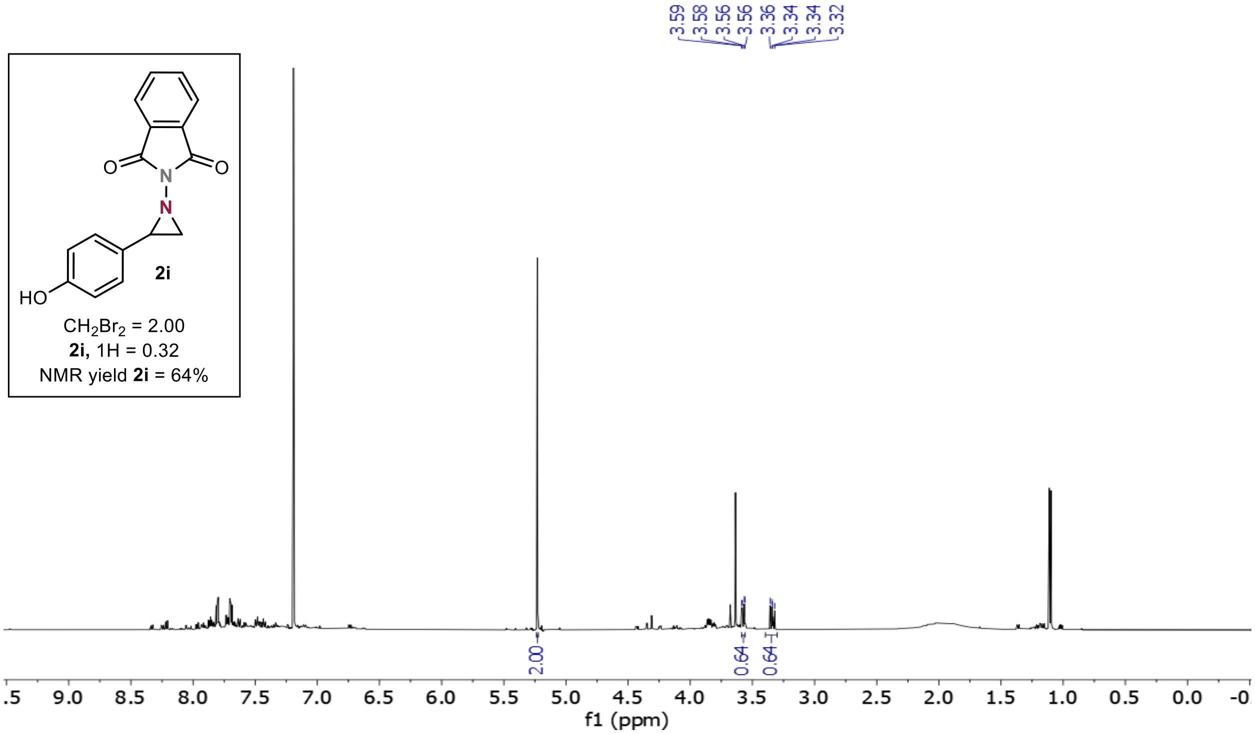

^{1}H NMR (500 MHz, $CDCl_3$) of 2-((2*R*,3*R*)-2-nitro-3-phenylaziridin-1-yl)isoindoline-1,3-dione (2bd)

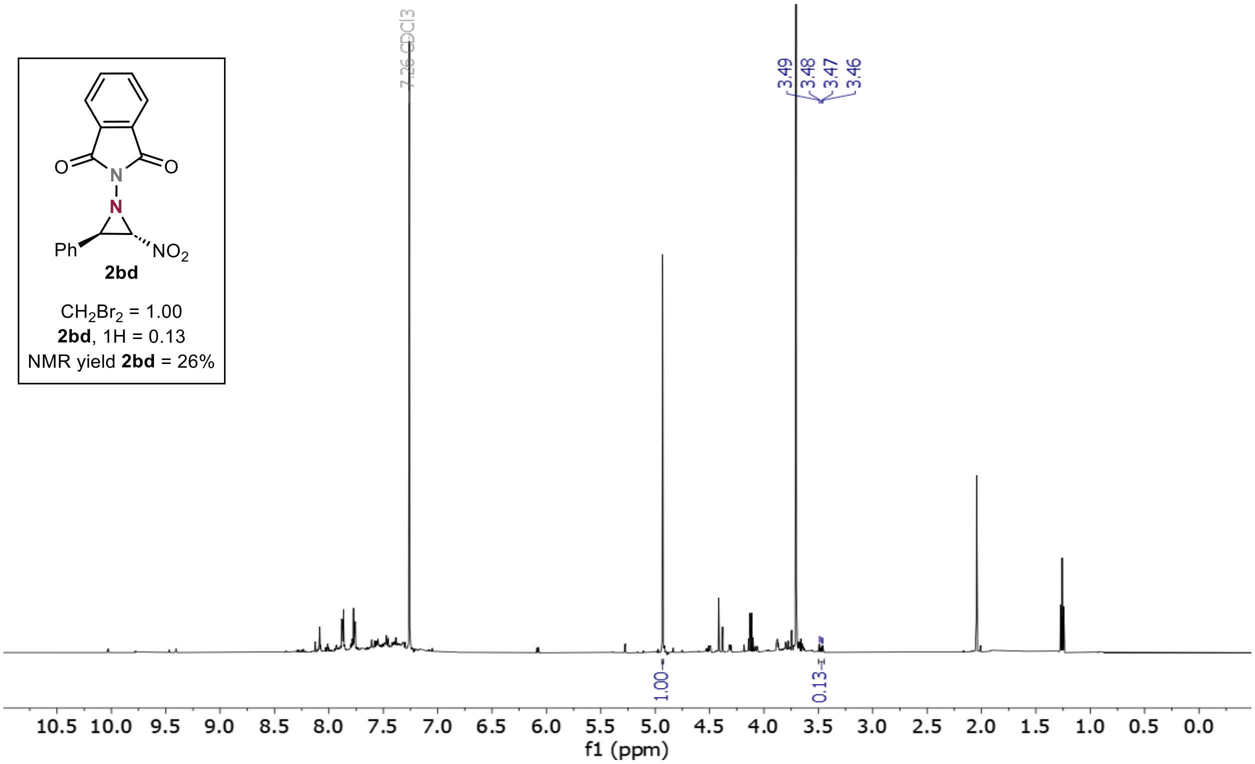

^{1}H NMR (500 MHz, $CDCl_3$) of (2*S*,3*R*)-1-(1,3-dioxoisoindolin-2-yl)-3-phenylaziridine-2-carbaldehyde (2be)

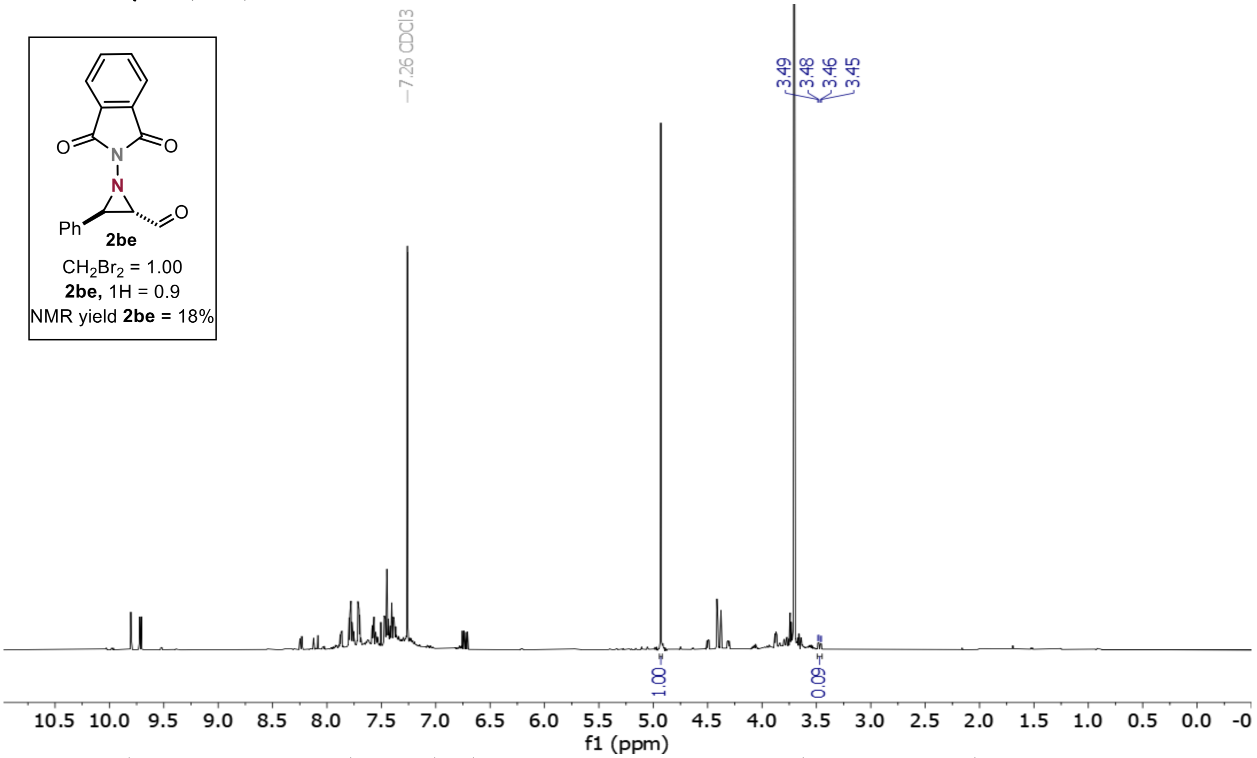

^{1}H NMR (400 MHz, $CDCl_3$) of 2-(2-(benzo[*d*][1,3]dioxol-5-yl)aziridin-1-yl)isoindoline-1,3-dione (2ah)

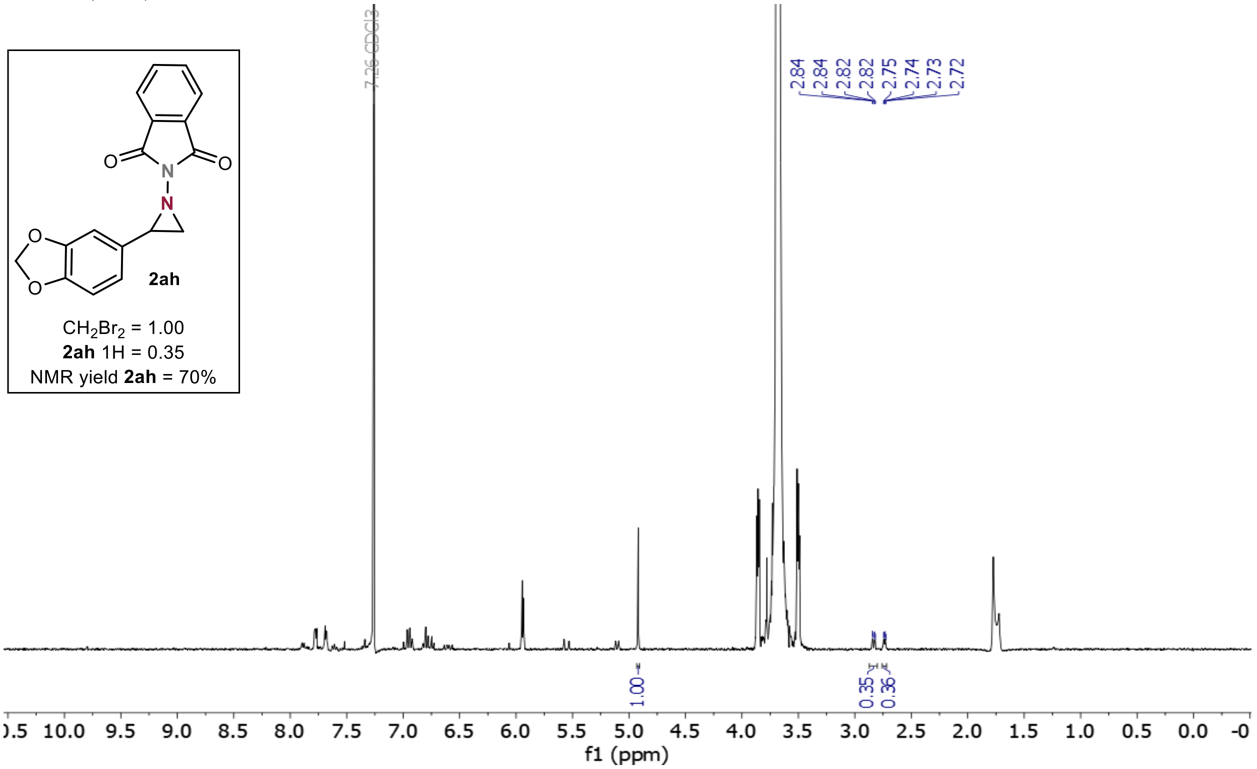

^{1}H NMR (500 MHz, $CDCl_3$) of 2-phenylaziridine (2b1)

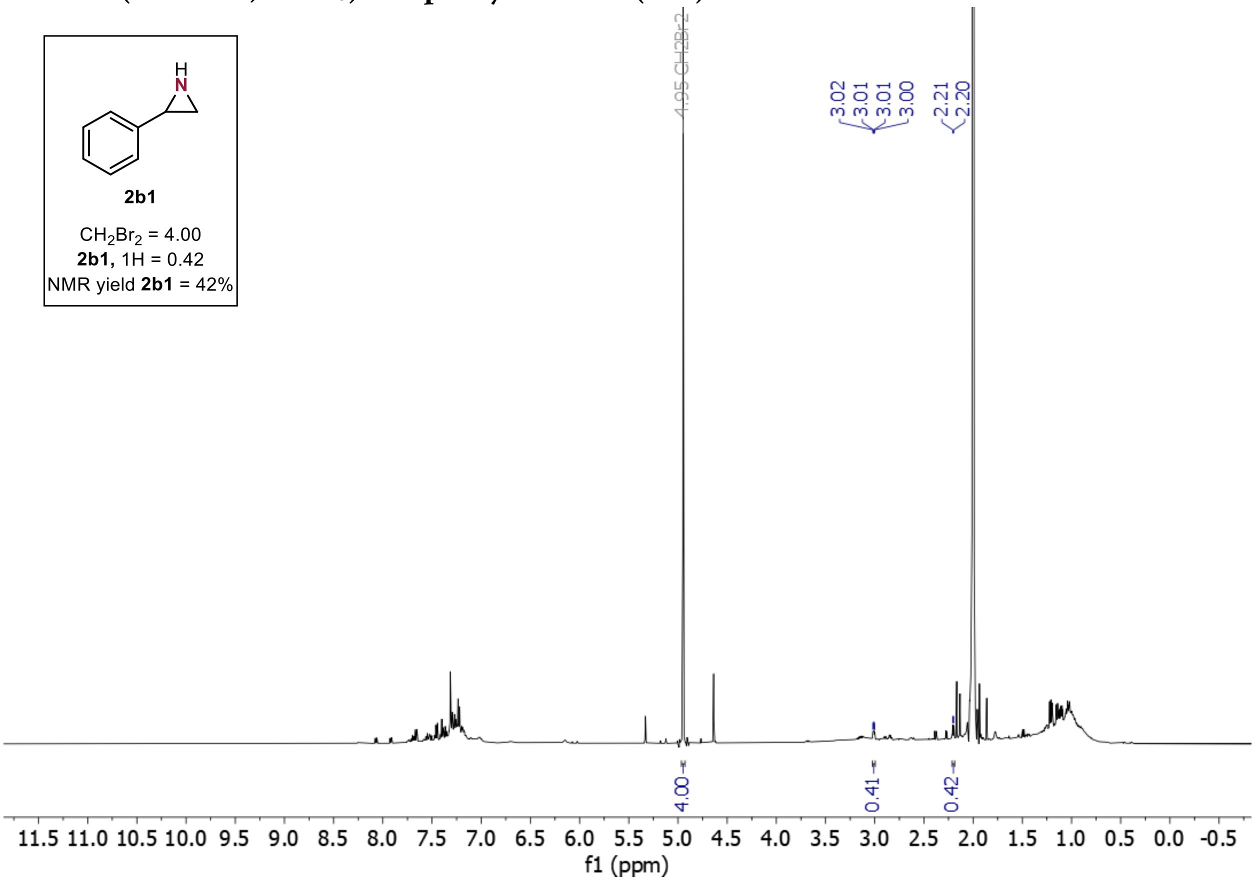

^{1}H NMR (500 MHz, $CDCl_3$) of *Cis* 9-azabicyclo[6.1.0]nonane (2al1)

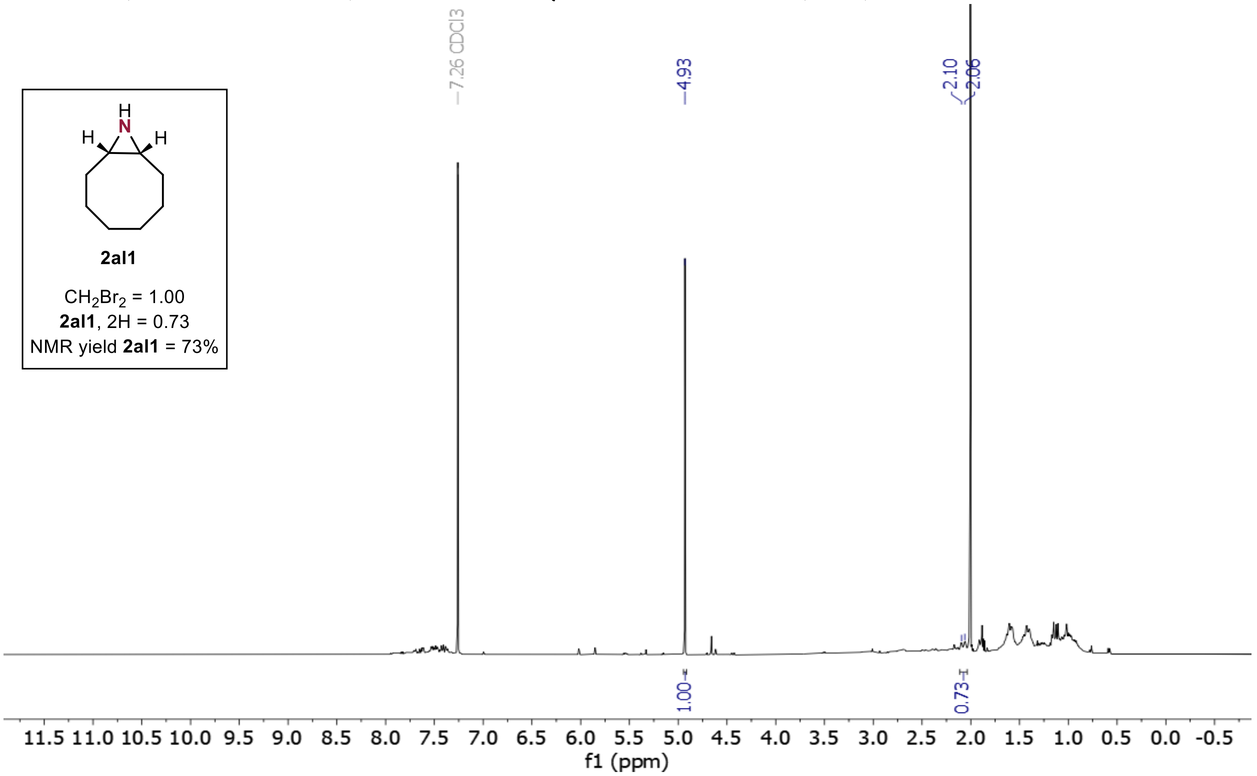

^{1}H NMR (400 MHz, $CDCl_3$) of ethyl 3-phenylaziridine-2-carboxylate (2x1)

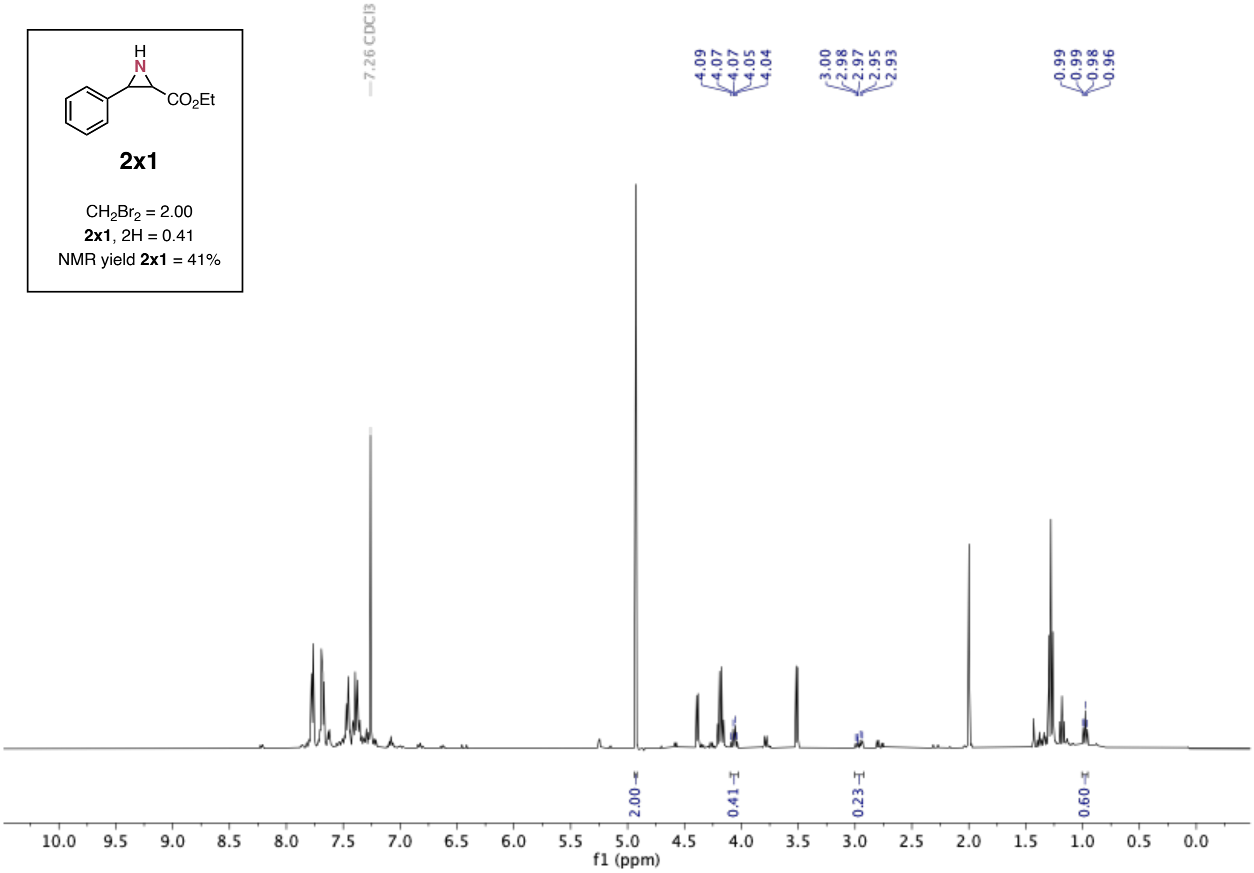

Continuous-Flow NMR Yield Spectra

^{1}H NMR (500 MHz, $CDCl_3$) of 2-(2-(pyridin-4-yl)aziridin-1-yl)isoindoline-1,3-dione (2ai)

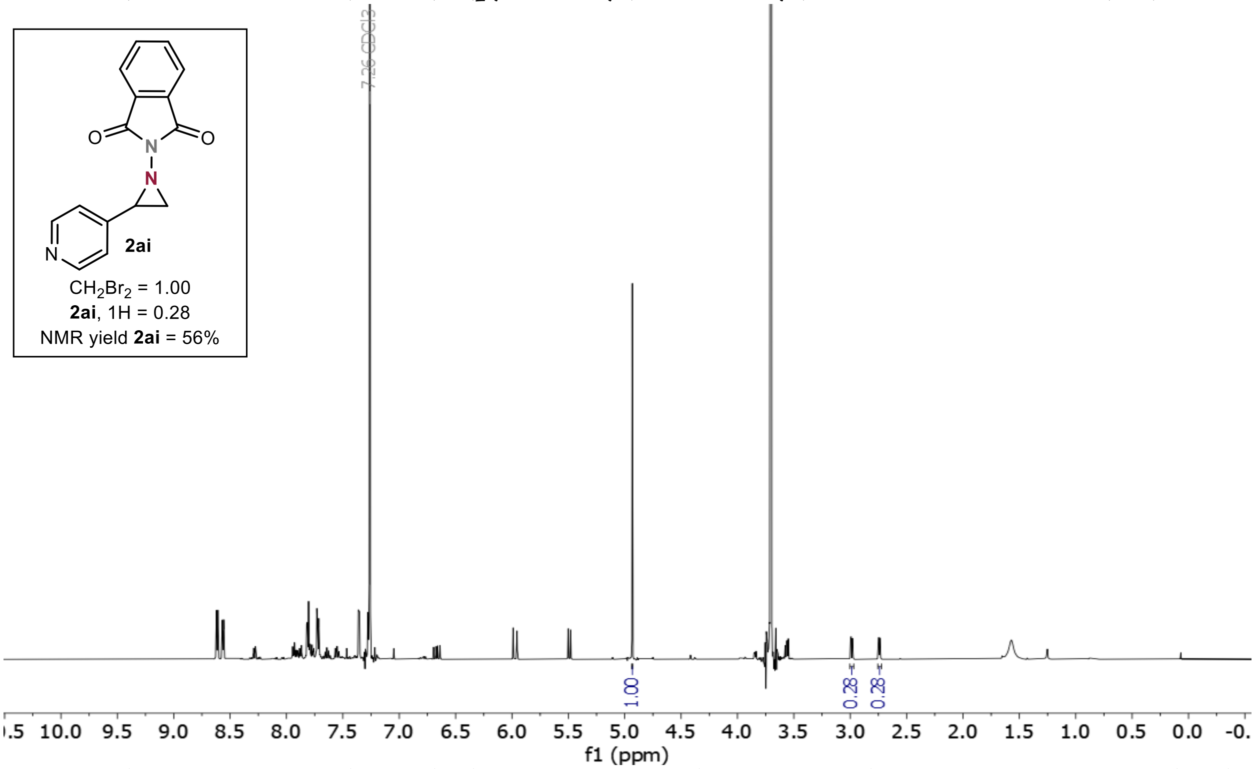

^{1}H NMR (500 MHz, $CDCl_3$) of 2-(2-(1*H*-imidazol-1-yl)aziridin-1-yl)isoindoline-1,3-dione (2ak)

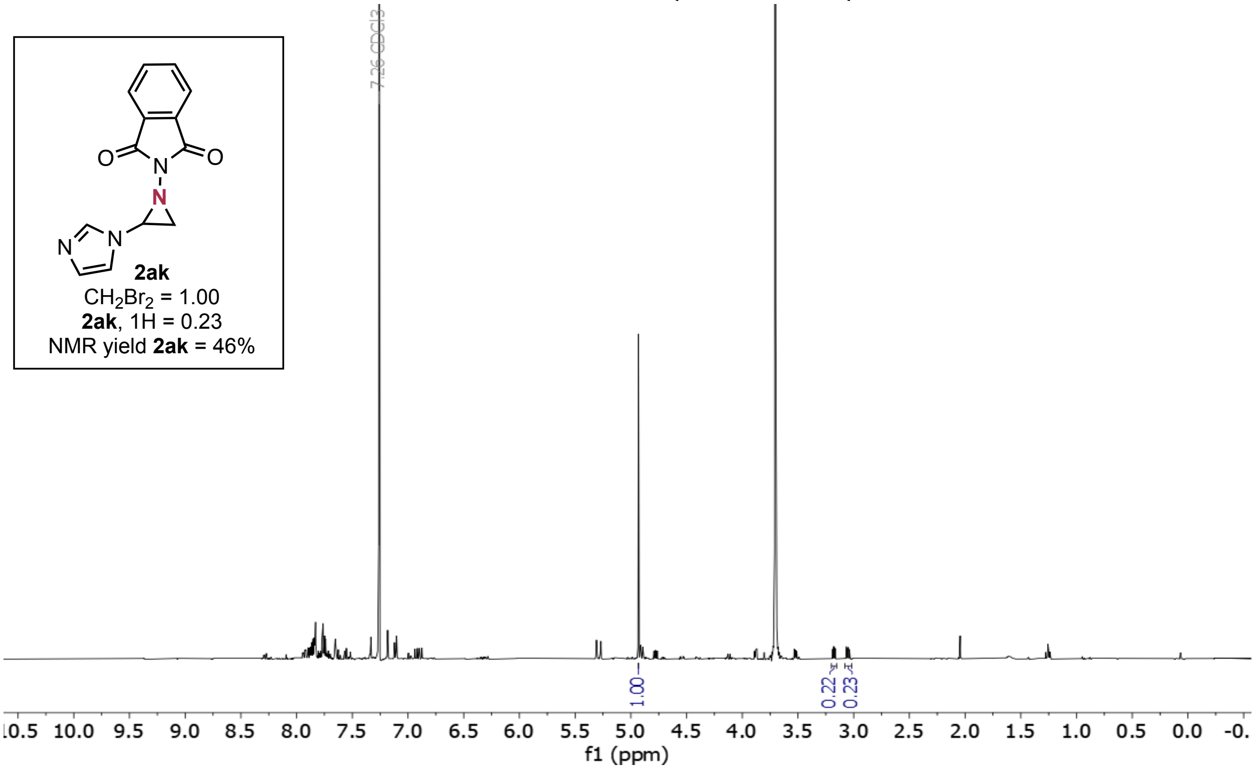

^{1}H NMR (500 MHz, CDCl$_3$) of 2-(2-(2-bromophenyl)aziridin-1-yl)isoindoline-1,3-dione (2m)

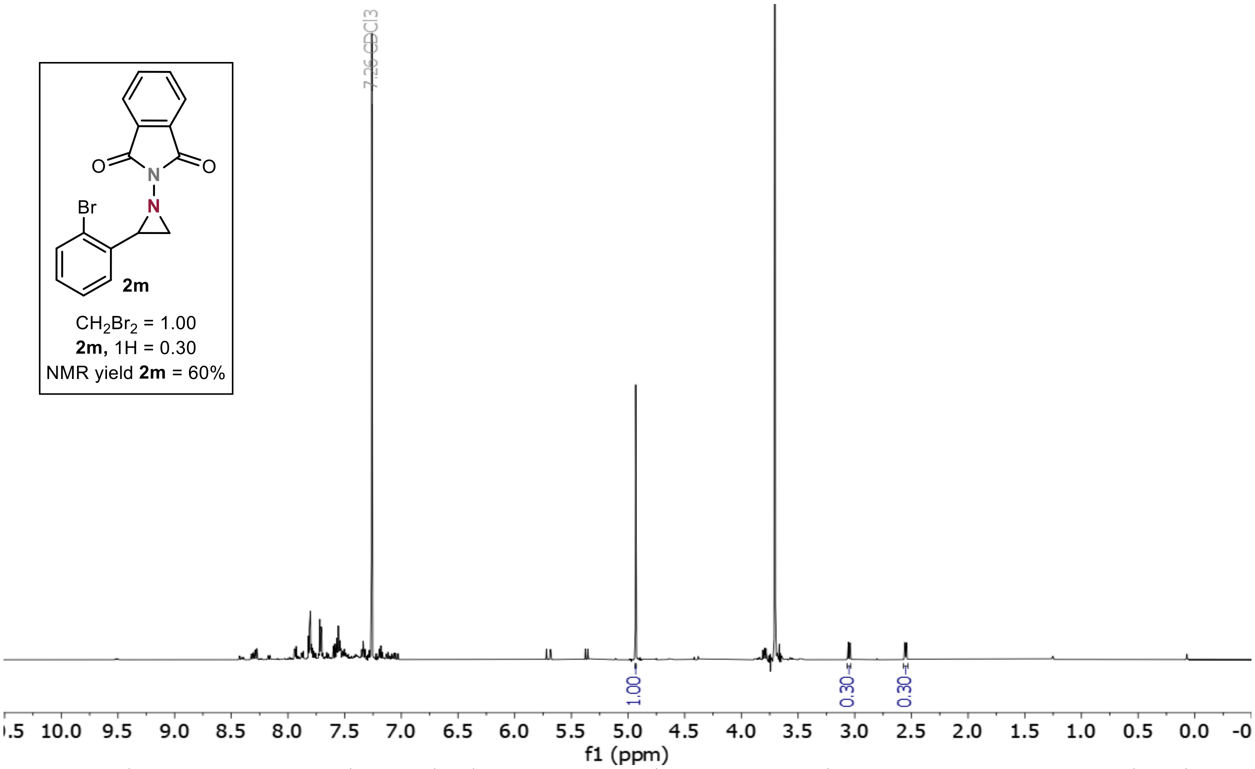

^{1}H NMR (500 MHz, CDCl$_3$) of 2-(2-(2-bromoethyl)aziridin-1-yl)isoindoline-1,3-dione (2ap)

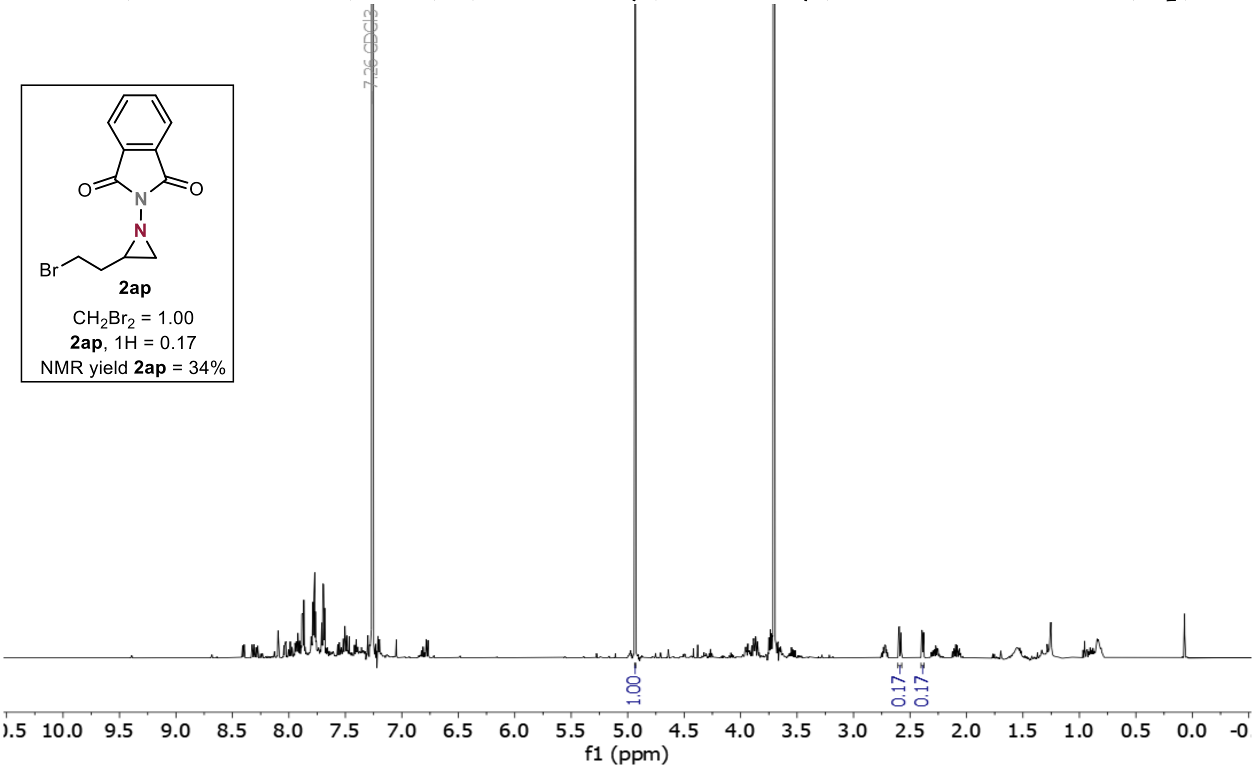

References

1. Hoesch, L.; Köppel, B. 1-Aryl- und 1-Alkyl-2-phthalimido-diazen-1-oxide, diacylierte Vertreter von trisubstituierten Triazen-1-oxiden: Bildung, Eigenschaften, Stereoisomerisierung und Fragmentierung. *Helv. Chim. Acta* **1981**, *64*, 864–889.
2. Zlotin, S. G.; Prokshits, O. V.; Karpenko, N. F., et al. Reaction of 1,1-Disubstituted Hydrazines with Dibromoisocyanurate in the Presence of Nitrosobenzene. *Russ. Chem. Bull.* **1990**, *39*, 1526–1528.
3. Moriarty, R. M.; Hopkins, T. E.; Prakash, I.; Vaid, B. K.; Vaid, R. K. Hypervalent Iodine Oxidation of Amines in the Presence of Nitroso Compounds: A Method for the Preparation of Unsymmetrically Substituted Azoxy Compounds. *Synth. Commun.* **1990**, *20*, 2353–2357.
4. Chen, J.; Yan, W.-Q.; Lam, C. M.; Zeng, C.-C.; Hu, L.-M.; Little, R. D. Electrocatalytic Aziridination of Alkenes Mediated by *n*-Bu_4NI: A Radical Pathway**.** *Org. Lett.* **2015**, *17*, 986–989.
5. Belov, V. N.; Kuznetsov, M. A. Reaction of Phthalimidonitrene with p-Methoxystyrene. *J. Org. Chem. USSR (Engl. Transl.)* **1988**, *24*, 1160–1163.
6. Li, J.; Chan, P. W. H.; Che, C.-M. Aryl Iodide Mediated Aziridination of Alkenes. *Org. Lett.* **2005**, *7*, 5801–5804.
7. Yoshimura, A.; Middleton, K. R.; Zhu, C.; Nemykin, V. N.; Zhdankin, V. V. Hypoiodite-Mediated Metal-Free Catalytic Aziridination of Alkenes. *Angew. Chem. Int. Ed.* **2012**, *51*, 8059-8062.
8. Anderson, D.; Horwell, D.; Atkinson, R. Substituent interactions in slow-inverting aziridines. *J. Chem. Soc. C: Org.* **1971**, 624-628.
9. Xue, W.; Zhu, Z.; Chen, S.; You, B.; Tang, C. Atomically Dispersed Co-N/C Catalyst for Divergent Synthesis of Nitrogen-Containing Compounds from Alkenes. *J. Am. Chem. Soc.* **2023**, *145*, 4142–4149.
10. Jat, J. L.; Paudyal, M. P.; Gao, H.; Xu, Q.-L.; Yousufuddin, M.; Devarajan, D.; Ess, D. H.; Kürti, L.; Falck, J. R. Direct Stereospecific Synthesis of Unprotected N–H and N–Me Aziridines from Olefins. *Science* **2014**, *343*, 61–65.
11. Hili, R.; Yudin, A. K. Amphoteric Amino Aldehydes Enable Rapid Assembly of Unprotected Amino Alcohols. *Angew. Chem. Int. Ed.* **2008**, *47*, 4188-4191
12. Wise, D. E.; Gogarnoiu, E. S.; Duke, A. D.; Paolillo, J. M.; Vacala, T. L.; Hussain, W. A.; Parasram, M. Photoinduced Oxygen Transfer Using Nitroarenes for the Anaerobic Cleavage of Alkenes. *J. Am. Chem. Soc.* **2022**, *144*, 15437–15442.
13. Mitchell, J. K.; Hussain, W. A.; Bansode, A. H.; O'Connor, R. M.; Wise, D. E.; Choe, M. H.; Parasram, M. Photoinduced Nitroarenes as Versatile Anaerobic Oxidants for Accessing Carbonyl and Imine Derivatives. *Org. Lett.* **2023**, *25*, 6517–6521.
14. Paolillo, J. M.; Duke, A. D.; Gogarnoiu, E. S.; Wise, D. E.; Parasram, M. Anaerobic Hydroxylation of C(sp3)-H Bonds Enabled by the Synergistic Nature of Photoexcited Nitroarenes. *J. Am. Chem. Soc.* **2023**, *145*, 2794–2799.
15. Maestre, L.; Sameera, W. M. C.; Díaz-Requejo, M. M.; Maseras, F.; Pérez, P. J. A General Mechanism for the Copper-and Silver-Catalyzed Olefin Aziridination Reactions: Concomitant Involvement of the Singlet and Triplet Pathways. *J. Am. Chem. Soc.* **2013**, *135*, 1338–1348.
16. Guo, Y.; Pei, C.; Empel, C.; Jana, S.; Koenigs, R. M. Photochemical Nitrene Transfer Reactions of Iminoiodinanes with Sulfides. *ChemPhotoChem.* **2022**, *6*, e202100293.
17. Atkinson, R. S.; Judkins, B. D.; Khan, N. 2,4-Dinitrobenzenesulphenylnitrene: addition to (Z)- and (E)-1-phenylpropene. *J. Chem. Soc., Perkin Trans. 1* **1982**, *1*, 2491–2497.

18. Siu, T.; Picard, C. J.; Yudin, A. K. Development of Electrochemical Processes for Nitrene Generation and Transfer. *J. Org. Chem.* **2005**, *70*, 932–937.
19. Deng, T.; Mazumdar, W.; Yoshinaga, Y.; Patel, P. B.; Malo, D.; Malo, T.; Wink, D. J.; Driver, T. G. Rh_2(II)-Catalyzed Intermolecular *N*-Aryl Aziridination of Olefins Using Nonactivated N Atom Precursors. *J. Am. Chem. Soc.* **2021**, 143, 19149–19159.
20. Li, Y.; He, J.; Khankhoje, V.; Herdtweck, E.; Köhler, K.; Storcheva, O.; Cokoja, M.; Kühn, F. E. Copper(II) Complexes Incorporating Poly/Perfluorinated Alkoxyaluminate-Type Weakly Coordinating Anions: Syntheses, Characterization, and Catalytic Application in Stereoselective Olefin Aziridination. *Dalton Trans.* **2011**, *2011*, 5746–5754.
21. Evans, D. A.; Bilodeau, M. T.; Faul, M. M. Development of the Copper-Catalyzed Olefin Aziridination Reaction. *J. Am. Chem. Soc.* **1994**, *116*, 2742–2753.
22. Mahy, J.-P.; Bedi, G.; Battioni, P.; Mansuy, D. Aziridination of Alkenes Catalyzed by Porphyrinirons: Selection of Catalyts for Optimal Efficiency and Stereospecificity. *J. Chem. Soc. Perkin Trans. II* **1988**, *1988*, 1517–1524.
23. Atkinson, R. S.; Kelly, B. J. Oxidation of N-Aminoquinarolones in the Presence of Alkenes: Evidence against Involvement of N-Nitrenes. *J. Chem. Soc. Chem. Comm.* **1987**, *18*, 1362–1363.
24. Li, J.; Liang, J.-L.; Chan, P. W. H.; Che, C.-M. Aziridination of alkenes with N-substituted hydrazines mediated by iodobenzene diacetate. Tetrahedron Lett. **2004**, *45*, 2685–2688.
25. McConaghy, J. S.; Lwowski, W. Singlet and triplet nitrenes. I. Carbethoxynitrene generated by α elimination. *J. Am. Chem. Soc.* **1967**, *89*, 2357–2364.
26. Atkinson, R. S.; Jones, D. W.; Kelly, B. Evidence for phthalimidonitrene as a common intermediate in several extrusion reactions. J. *Chem. Soc., Perkin Trans. 1.* **1991**, 1344–1346.
27. Richardson, R. D.; Desaize, M.; Wirth, T. Hypervalent Iodine-Mediated Aziridination of Alkenes: Mechanistic Insights and Requirements for Catalysis. *Chem. – Eur. J.* **2007**, *13*, 6745–6754.
28. Newcomb, M. Kinetics of Radical Reactions: Radical Clocks. In *Comprehensive Organic Synthesis II*; Eds. Trost, B. M., Fleming, I., Paquette, L. A.; Elsevier: Amsterdam, **2001**; pp 316–336.
29. Zhang, Z. Q.: Meng, X. Y.; Sheng, J.; Lan, Q.; Wang, X. S. Enantioselective copper-catalyzed 1, 5-cyanotrifluoromethylation of vinylcyclopropanes. *Org. Lett.* **2019**, *21*, 8256-8260.
30. Harada, S.; Matsuda, D.; Morikawa, T.; Nishida, A. Direct Synthesis of Enones by Visible-Light-Promoted Oxygenation of Trisubstituted Olefins Using Molecular Oxygen. *Synlett*, **2020**, *31*, 1372-1377.
